# Distinct repair outcomes from single and convergent replication fork collapse

**DOI:** 10.1038/s41594-026-01812-9

**Published:** 2026-05-27

**Authors:** Sara C. Conwell, Khushi V. N. Patel, Savannah J. Weeks-Pollenz, Steven N. Dahmen, Matthew T. Cranford, William G. Dunphy, David T. Long, David Cortez, James M. Dewar

**Affiliations:** 1 Department of Biochemistry, Vanderbilt University School of Medicine, Nashville, TN USA; 2 Division of Biology and Biological Engineering, California Institute of Technology, Pasadena, CA USA; 3 Department of Biochemistry and Molecular Biology, Medical University of South Carolina, Charleston, SC USA

**Keywords:** Stalled forks, Single-strand DNA breaks, Homologous recombination, Double-strand DNA breaks, DNA synthesis

## Abstract

Replication fork collapse at single-strand DNA breaks threatens genome stability but how such forks are repaired and resolved has remained unclear. Here we replicate site-specific nicks with single or converging replication forks in *Xenopus* *laevis* egg extracts. Collapse of a single fork generates a single-ended double-strand break (DSB) that undergoes homologous recombination to yield stable D-loops and end-to-end fusions, yet does not restart DNA synthesis. Single collapsed forks can also undergo extensive nucleolytic degradation, appearing to disassemble the sister fork through ‘secondary collapse’ events that resolve single-ended DSBs without engaging DSB repair. In contrast, semisynchronous convergent collapse generates a double-ended DSB that is primarily repaired through annealing-dependent DSB repair, completing DNA synthesis but generating precise deletions and templated insertions. These error-prone products are not detected following single-fork collapse. Our findings demonstrate that single and semisynchronous convergent collapsed forks elicit distinct repair outcomes.

## Abstract (web summary)

Here the authors show that collapse of single replication forks causes recombination, end fusions or degradation but does not restart DNA synthesis. In contrast, collapse of convergent forks completes DNA synthesis through error-prone double-strand break repair.

## Main

Tens of thousands of single-strand DNA breaks (SSBs) arise each day^1,2^. When a replication fork encounters an SSB, it generates a double-strand break (DSB)^3–8^. This process, termed ‘fork collapse’ (ref. ^7^), blocks DNA synthesis and occurs every cell cycle in humans^9^ in response to SSBs generated by topoisomerases^4^, transcription^10^, viral infection^11^, antiviral responses^12^, lagging-strand maturation^13^, abasic sites^14^, defective DNA repair^15^ and genome editing^16^. Unresolved collapsed forks are highly mutagenic and potentially oncogenic^5,17^. Fork collapse is central to many anticancer therapies^18^, including poly(ADP-ribose) polymerase (PARP) inhibitors^1,19,20^. Although multiple pathways can resolve collapsed forks^5,8,21–41^, how their usage is determined remains unclear. Addressing these questions is imperative given the profound implications of fork collapse for normal cellular functioning and human health.

**Extended Data Fig. 1:**
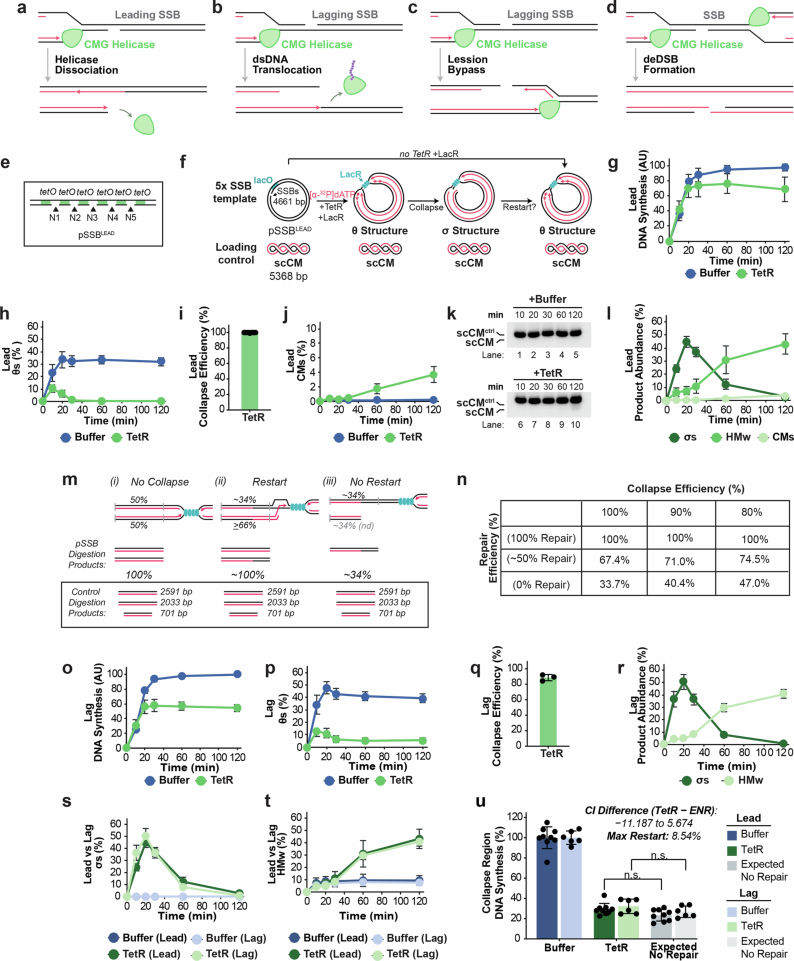
Analyses of leading and lagging collapse in *Xenopus* egg extracts. (**a**) Encounter with an SSB on the leading strand template, results in a blunt seDSB (leading collapse). (**b**) Encounter with an SSB on the lagging strand template, results in an seDSB with an overhang (lagging collapse). (**c)** Encounter with a lagging SSB can also result in continued unwinding and a deDSB. (**d)** Fork convergence at an SSB can also form a deDSB. (**e)** Template design with five tandem SSBs. To induce leading collapse, pSSB^LEAD^ contains 5x tandem Nb.BsmI nicking sites (‘N1’ to ‘N5’) each flanked by 1x *tetracycline* operator (*tetO*) sequence either side. To induce lagging collapse, pSSB^LAG^ contains the reverse complement of the *tetO*/SSB array from pSSB^LEAD^. (**f)** Pre-replicated control plasmid schematic and normalization procedure. Schematic depicting the DNA structures from Fig. 1a and the loading control, replicated prior to pSSB^LEAD^. (**g)** Quantification of total DNA synthesis from Fig. 1b normalized to the maximum signal across all time points and conditions. Total DNA synthesis remained unperturbed prior to collapse. Mean ± S.D., n = 9 independent experiments. (**h)** Quantification of θ structures from Fig. 1b. θ structures (converging fork intermediates) were abolished upon TetR addition. Mean ± S.D., n = 9 independent experiments. (**i)** Quantification of collapse efficiency from Fig. 1b. Collapse efficiency approached 100% (calculated as described in methods). Mean ± S.D., n = 9 independent experiments. (**j)** Quantification of nicked plus supercoiled circular monomers (CMs) from Fig. 1b. Mean ± S.D., n = 9 independent experiments. (**k)** Overexposure of Fig. 1b (bottom) showing that scCMs form after collapse (lane 10) but not without collapse (lane 5). (**l)** Quantification of σ structures (σs), high molecular weight products (HMw), and circular monomers (CMs) from Fig. 1b. Mean ± S.D., n = 9 independent experiments. (**m)** Schematic of expected XmnI-SacI digestion products from Fig. 1e. Control digestion products arise from digestion of the pre-replicated control plasmid. Fragment lengths (bp) and expected radioactive signal relative to the ‘no collapse’ control are indicated. (**n)** Table of expected DNA synthesis across the collapse region (%) for various collapse and repair efficiencies. See Supplementary Table 2 for a worked example. (**o)** Quantification of total DNA synthesis from Fig. 1i normalized to the maximum signal across all time points and conditions. Mean ± S.D., n = 3 independent experiments. (**p)** Quantification of θ structures from Fig. 1i. Mean ± S.D., n = 3 independent experiments. (**q)** Quantification of collapse efficiency (see methods) from Fig. 1i. Mean ± S.D., n = 3 independent experiments. (**r)** Quantification of σ structures (σs) and high molecular weight products (HMw) from Fig. 1i. Mean ± S.D., n = 3 independent experiments. (**s)** Side-by-side quantification of σ structures (σs) from Fig. 1b, i. Mean ± S.D., n = 9 independent experiments for leading collapse and n = 3 for lagging collapse. (**t)** Side-by-side quantification of high molecular weight products (HMw) from Fig. 1b, i. Mean ± S.D., n = 9 independent experiments for leading collapse and n = 3 for lagging collapse. (**u)** Side-by-side quantification of collapse region DNA synthesis following lead and lag collapse, as originally depicted in Fig. 1g, n. Mean ± S.D., n = 9 independent experiments for leading collapse and n = 6 for lagging collapse. One-way ANOVA with Dunnett’s test. The 95% confidence interval of the difference (TetR − Expected No Repair) was −11.187 to 5.674. Maximum undetectable restart, from the upper bound, was 8.54%. Source data

When a replication fork encounters an SSB, the fork can break to form a single-ended DSB (seDSB)^4–6,21,22,27^, leaving a sister DNA duplex with a gap that is rapidly filled and ligated^6^. In parallel, the replisome is lost through different mechanisms depending on which parental strand contains the SSB. A leading-strand template SSB (‘leading collapse’) causes rapid replisome dissociation^6^ from loss of the translocating strand (Extended Data Fig. 1a). A lagging-strand template SSB (‘lagging collapse’) causes either replisome unloading through the termination pathway (Extended Data Fig. 1b)^6^ or continued unwinding^22^ to form a double-ended DSB (deDSB) behind the fork (Extended Data Fig. 1c)^21–23^. deDSBs also form at leading SSBs^21–23,42,43^, likely through a converging fork^5,22,27^ (Extended Data Fig. 1d). Fork convergence at an SSB is reported to generate a deDSB^43,44^ but an seDSB intermediate has not been observed and extensive rereplication can occur^44,45^. Thus, we lack direct evidence that converging forks at an SSB generate an seDSB that converts to a deDSB.

DSB ‘resolution’ at collapsed forks (removal of the free DNA end) depends critically on homologous recombination (HR)^3,5,8,21–26^. Resolution likely involves RAD51-dependent D-loop formation^26^, regardless of whether an seDSB or deDSB forms^24^. In contrast, replication-independent DSBs undergo both HR and end joining^46–48^. End joining may occur following fork collapse^27,28,49–52^ but its contribution remains unclear. Once a D-loop forms at an seDSB, it is capable of extensive DNA synthesis through ‘break-induced replication’ (BIR)^53–55^, which is well established in eukaryotes^56^ and may function analogously to bacterial replication restart^57^. However, several observations call into question BIR as a major restart mechanism; BIR is detected through error-prone repair events^29,36^ that may not represent most outcomes, key bacterial restart proteins are not conserved in eukaryotes^21^, BIR is strongly negatively regulated^27^ and human BIR proteins^58,59^ are dispensable for resolving DNA ends following fork collapse^22^. Direct analysis of BIR is complicated because interfering with seDSB resolution results in deDSB formation by fork convergence^22^. Hence, it remains unclear how effectively collapsed forks complete DNA synthesis, with or without fork convergence to generate a deDSB.

To address these questions, we replicated ‘simple’ SSBs using *Xenopus* *laevis* egg extracts^60,61^, which mimic the nuclear proteome of human cells^62^, support many DNA repair pathways^63^ and recapitulate fork collapse mechanisms that function in human cells^6,22^. We found that either leading or lagging collapse induced seDSBs that were efficiently resolved by RAD51 to generate joint molecules arising from D-loops, as well as erroneous end-to-end fusions involving microhomology. Restart of DNA synthesis was not detected at single collapsed forks. seDSBs generated by leading but not lagging template SSBs also underwent extensive resection that disassembled the sister fork through ‘secondary collapse’. In contrast, semisynchronous convergent collapse generated deDSBs that efficiently completed DNA synthesis independently of RAD51 through annealing-dependent DSB repair, which generated precise deletions and templated insertions. We observed similar outcomes at SSBs generated by Cas9 nickase (nCas9; H840A). Overall, our data demonstrate that single and convergent collapsed forks can elicit distinct repair outcomes.

## Results

### Leading and lagging collapse elicit DSB resolution but not replication restart

**Fig. 1:**
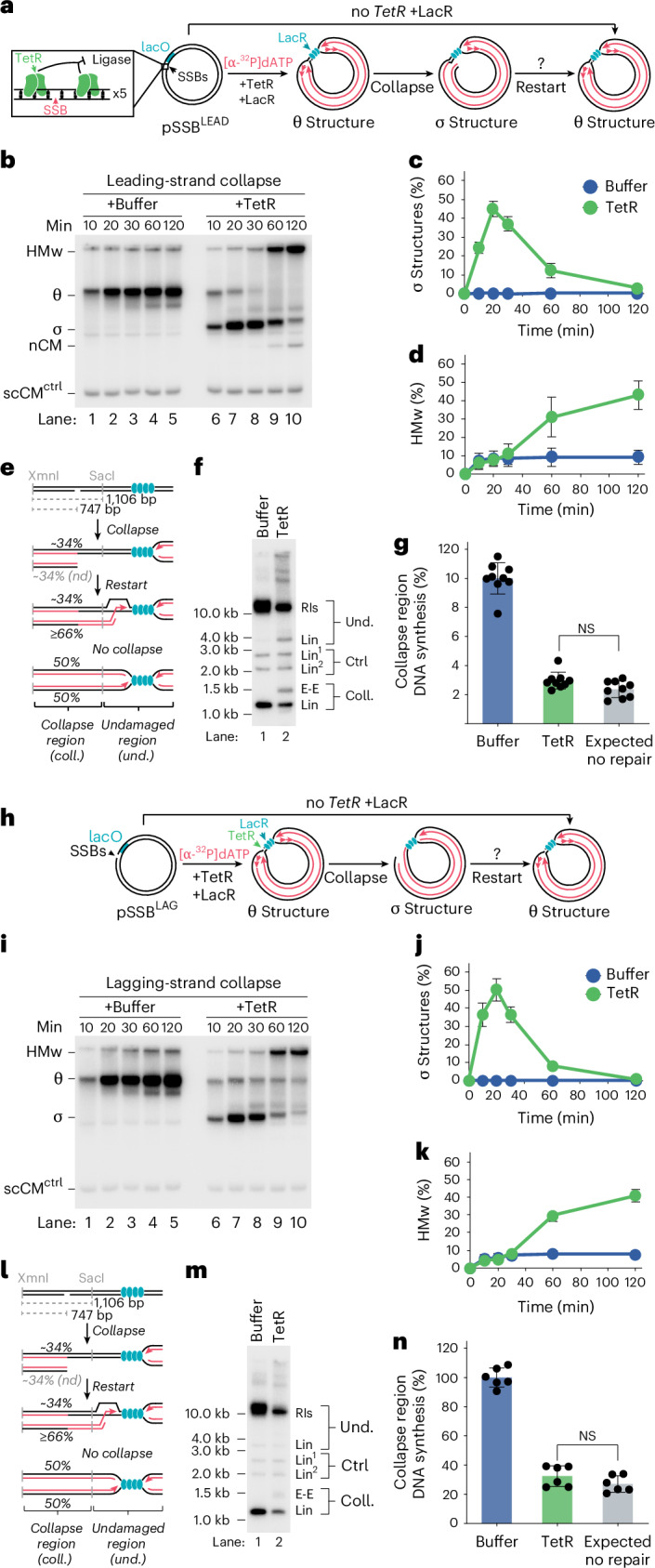
Leading and lagging seDSBs are resolved but do not restart DNA synthesis. **a**, pSSB^LEAD^ contains five tandem, site-specific and strand-specific SSBs generated by nicking endonucleases before enzyme removal. SSBs are flanked by *tetO* arrays bound by TetR to competitively inhibit religation without impairing replisome progression (Extended Data Fig. 1e). A downstream 1.6-kb *lacO* array bound by LacR blocks converging forks at a distance^60^ to isolate leading-strand collapse events. Plasmids harbor multiple sequence-nonspecific origins distributed throughout the backbone^89,90^. The *tetO* array is placed 320 bp from the LacR array, such that approximately 90% of events should involve a single fork encountering the SSB given the 2,684-bp backbone. pSSB^LEAD^ was replicated using *X*. *laevis* egg extracts derived from replicating nuclei^61^ in the presence of TetR (+TetR) to stabilize the SSBs. TetR was omitted in the buffer control (+buffer), which allowed religation of SSBs before replication and prevented collapse (as described previously^6^). LacR was included to impede converging replication forks and ensure strand-specific fork collapse at a single fork. Nascent strands were radiolabeled by inclusion of [α-^32^P]dATP. A prereplicated internal control plasmid (scCM^ctrl^) was included as a loading control for normalization of replication intermediates (Extended Data Fig. 1f). **b**, Samples from **a** were separated on an agarose gel and visualized by autoradiography (Extended Data Fig. 1g–l). **c**, Quantification of σ structures from **b**. Data are presented as the mean ± s.d. (*n* = 9 independent experiments). **d**, Quantification of HMw products from **b**. Data are presented as the mean ± s.d. (*n* = 9 independent experiments). **e**, Schematic depicting the assay for restart of DNA synthesis in the collapse region (Extended Data Fig. 1m,n). **f**, Purified DNA samples from *t* = 120 in **b** were digested with XmnI and SacI, then separated on an agarose gel and visualized by autoradiography. **g**, Quantification of collapse region DNA synthesis from **f** as in **e**. Signal was normalized to control fragment ‘Lin^1^’. Expected values for no repair account for replication efficiency and collapse efficiency (Extended Data Fig. 1n). Data presented are the mean ± s.d. (*n* = 9 independent experiments). Data were analyzed by one-way analysis of variance (ANOVA) and Dunnett’s multiple-comparison method. **h**, Schematic of pSSB^LAG^ as for pSSB^LEAD^ in **a**, but with SSBs positioned on the lagging-strand template. **i**, Samples from **h** were separated and visualized as in **b** (Extended Data Fig. 1o–r). **j**, Quantification of σ structures from **i** as in **c**. Data are presented as the mean ± s.d. (*n* = 3 independent experiments). **k**, Quantification of HMw products from **i** as in **d**. Data are presented as the mean ± s.d. (*n* = 3 independent experiments). **l**, Schematic of the assay for collapse region DNA synthesis as in **e**, but for lagging collapse. **m**, Purified DNA samples from *t* = 120 in **i** were digested and visualized as in **f**. **n**, Quantification of collapse region DNA synthesis from **m** as in **g**. Data are presented as the mean ± s.d. (*n* = 6 independent experiments). Data were analyzed by one-way ANOVA and Dunnett’s multiple-comparison method. Lin, linear; E–E, end–end joining; NS, not significant; nd, not determined. Source data

To examine leading-strand fork collapse, we replicated pSSB^LEAD^ (Fig. 1a and Extended Data Fig. 1e,f)^6,60,64^ in *X*. *laevis* egg extracts^61^. Replication without TetR permitted SSB religation^6^, yielding θ structures arising from converging forks stalled at the LacR barrier (Fig. 1b, lanes 1–5)^60^. TetR addition stabilized SSBs, converting θ intermediates into σ structures arising from fork collapse (Fig. 1b, lanes 6–8, and Fig. 1c)^6^, with collapse approaching 100% (Extended Data Fig. 1g–i). Total DNA synthesis was unaltered (Extended Data Fig. 1g), indicating normal replication before collapse.

Following collapse, σ structures declined (Fig. 1c) and two products emerged. The major product was high-molecular-weight (HMw) species remaining in the well (Fig. 1b, lanes 9 and 10, and Fig. 1d), which resembled DSB repair products^65,66^. We also observed nicked and supercoiled circular monomers (nCMs and scCMs; Fig. 1b, lanes 9 and 10, and Extended Data Fig. 1j,k) that were much less abundant than HMw species (Extended Data Fig. 1l). Because CMs were absent from the control (Fig. 1b, lanes 1–5), they were unlikely to arise from displacement of the LacR barrier. Their existence suggested a mechanism that converts collapsed molecules into full-length circular plasmids (Fig. 3). Thus, seDSBs from leading collapse were efficiently converted into at least three products.

**Extended Data Fig. 2:**
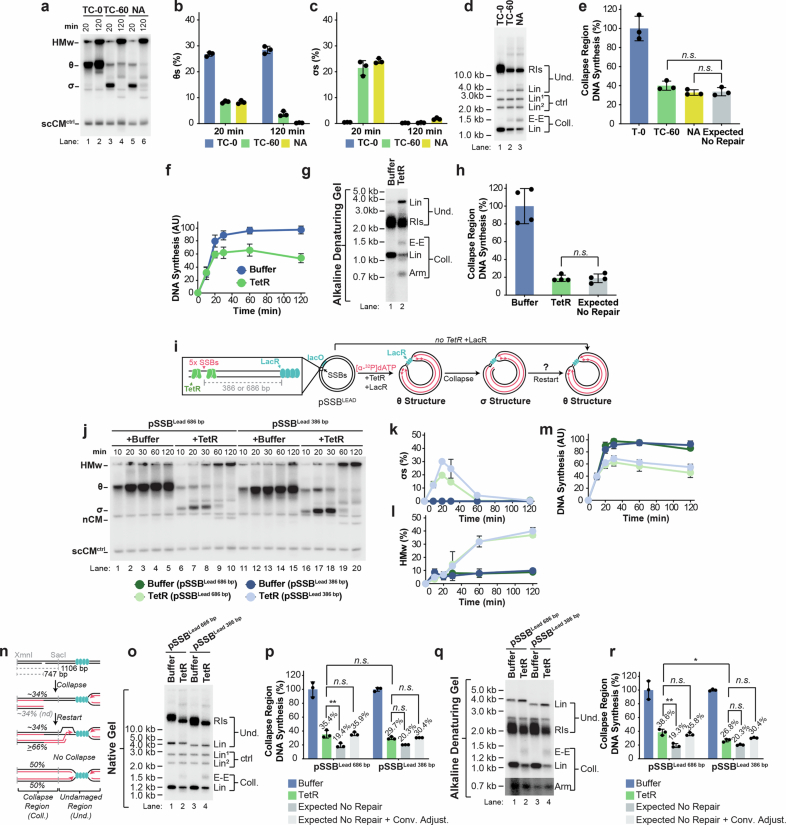
Lack of restart is not due to suppression by TetR or the LacR barrier. (**a)** To address whether TetR binding interferes with seDSB repair, pSSBLEAD was replicated as in Fig. 1b, lanes 6-10. Tetracycline (TC) was added at the onset of replication (TC-0), at 60 minutes (TC-60), or not added (NA control). Samples were separated on an agarose gel and analyzed by autoradiography. TC addition at the onset of replication blocked formation of σs (lanes 1, 3, 5), indicating efficient TetR displacement. (**b)** Quantification of θ structures from (a). Mean ± S.D., n = 3 independent experiments. (**c)** Quantification of σ structures (σs) from (a). Mean ± S.D., n = 3 independent experiments. TC-60 did not impact σ structure resolution, indicating residual TetR did not impair seDSB resolution. (**d)** Purified DNA samples from T = 120 in (a) were analyzed as in Fig. 1f. (**e)** DNA synthesis in the collapse region was quantified from (d) as in Fig. 1g. Mean ± S.D., n = 3 independent experiments. One-way ANOVA with Dunnett’s test. Post-collapse TC did not increase DNA synthesis, indicating TetR does not block restart. (**f)** To test whether complex intermediates masked detection of DNA synthesis in the collapse region (Fig. 1e-g), individual DNA strands were analyzed. DNA samples were generated as in Fig. 1f. Graph depicts total DNA normalized to the maximum signal across all time points and conditions. Mean ± S.D., n = 4 independent experiments. (**g)** Samples from (f) were then separated on an alkaline denaturing agarose gel to separate individual DNA strands then visualized by autoradiography. The broken arm was visible, demonstrating that DNA synthesis did not restart at the seDSB. (**h)** DNA synthesis in the collapsed region was quantified from (g) as in Fig. 1g. Control fragments could not be resolved in (g), so values were normalized to ‘Lin^1^’ from the corresponding native gels. Mean ± S.D., n = 4 independent experiments. One-way ANOVA with Dunnett’s test. Denaturing alkaline gel electrophoresis confirmed the absence of restart and revealed stable seDSB intermediates (arm) persisting for at least two hours. Expected no-repair synthesis was lower than in Fig. 1g due to reduced overall DNA synthesis in this experiment set (see f). (**i)** Plasmid DNAs containing leading SSBs were replicated in extract with LacR and either Tet Buffer or TetR, as in Fig. 1a. pSSB^LEAD^ (pSSB^LEAD-386 bp^) has the central nick site 386 bp from the *lacO* array. Plasmid pSSB^LEAD-686 bp^ has the nick site 686 bp away. (**j)** Samples from (i) were separated on an agarose gel and visualized by autoradiography. (**k)** Quantification of σ structures (σs) from (j). Mean ± S.D., n = 3 independent experiments. (**l)** Quantification of high molecular weight products (HMw) from (j). Mean ± S.D., n = 3 independent experiments. (**m)** Quantification of total DNA synthesis from (j) normalized to the maximum signal across all timepoints and conditions. Mean ± S.D., n = 3 independent experiments. (**n)** Schematic depicting the assay for restart of DNA synthesis in the collapse region. (**o)** Purified DNA samples from T = 120 in (j) were digested with XmnI and SacI, then separated on a native agarose gel and visualized by autoradiography. (**p)** Quantification of collapse region DNA synthesis from (o) as in (n). Signal was normalized to control fragment ‘Lin1’. ‘Expected No Repair’ values account for replication efficiency and collapse efficiency. In *Xenopus* egg extracts origins are delocalized and variable^2^, as in human cells^9^, and plasmids typically use multiple origins^3^. Thus a small fraction of plasmids is expected to undergo convergent collapse, which elicits efficient repair (see Figs. 4–5), and this fraction will be greater for pSSB^LEAD-686 bp^ where the *tetO* nick sites are further from the LacR array. To correct for origin firing between the *tetO* and *lacO* arrays ‘Expected No Repair + Conv. Adjust.’ accounts for the contribution of converging fork-dependent repair arising from stochastic origin firing between the TetR-bound SSBs and the LacR barrier. Mean ± S.D., n = 3 independent experiments. Two-tailed unpaired t-tests and two-way ANOVA with Šídák’s test. ** *P* = 0.0040 (**q)** Purified DNA samples from T = 120 in (j) were separated on an alkaline denaturing agarose gel and visualized by autoradiography. The persistent arm fragment confirmed the absence of detectable restart at the seDSB, and end-to-end fusion products (E-E) were also detected. (**r)** Quantification of collapse region DNA synthesis from (q) as in (n). Signal was normalized to control fragment ‘Lin1’ from the corresponding native gel. Expected values are as described in (p). Increasing the distance between the collapse site and the LacR array did not promote restart. A small increase in synthesis (p,r) was attributable to stochastic fork convergence, not restart, per convergence-adjusted analysis. Mean ± S.D., n = 3 independent experiments. Two-tailed unpaired t-tests and two-way ANOVA with Šídák’s test. *P = 0.0138 and ** *P* = 0.0020. Source data

seDSBs can restart replication^5,26–36^. To assess restart efficiency, we used restriction digests to measure nascent DNA synthesis around the leading SSB (collapse region DNA synthesis; Fig. 1e and Extended Data Fig. 1m,n). Collapse reduced synthesis ~4-fold in this region (Fig. 1f,g), matching levels expected if no restart had occurred (Fig. 1g). This reduction was not because of TetR blocking restart (Extended Data Fig. 2a–e) or interlinked repair intermediates (Extended Data Fig. 2f–h). Denaturing analysis confirmed the absence of restart and detected the broken end after 2 h (arm; Extended Data Fig. 2g), indicating a stable seDSB. Both native and denaturing analyses detected end-to-end fusions (Fig. 1f and Extended Data Fig. 2g). Increasing the distance between the collapse site and the LacR array did not promote restart (Extended Data Fig. 2i–r); a small increase in synthesis was attributable to fork convergence arising from multiple sequence-nonspecific origins (Extended Data Fig. 2q,r). Thus, seDSBs arising from leading collapse did not detectably restart DNA synthesis, regardless of distance from the LacR barrier.

We next examined lagging seDSBs using pSSB^LAG^ (Fig. 1h and Extended Data Fig. 1o). Lagging collapse also approached 100% efficiency (Extended Data Fig. 1p,q) and generated seDSBs (Fig. 1i,k) that were converted to HMw species (Fig. 1i,k and Extended Data Fig. 1r–t), as for leading collapse (Extended Data Fig. 1s,t). No DNA synthesis was detected in the collapse region (Fig. 1l–n) and apparent end-to-end fusions were detected (Fig. 1m), also as for leading collapse. However, several differences were noted; CMs were not detected (Fig. 1I), θ intermediates remained detectable, indicating less efficient collapse (Fig. 1i, lanes 6–10), and a more pronounced reduction in total DNA signal occurred (Extended Data Fig. 1o), suggesting increased degradation. By combining leading and lagging collapse data, we calculated that no more than 8.5% of collapse events led to restart (Extended Data Fig. 1u). Overall, both leading and lagging collapse generated seDSBs that were resolved to HMw species, with replication restart either rare (≤8.5%) or absent.

### Collapsed forks form D-loops and undergo end joining

**Fig. 2:**
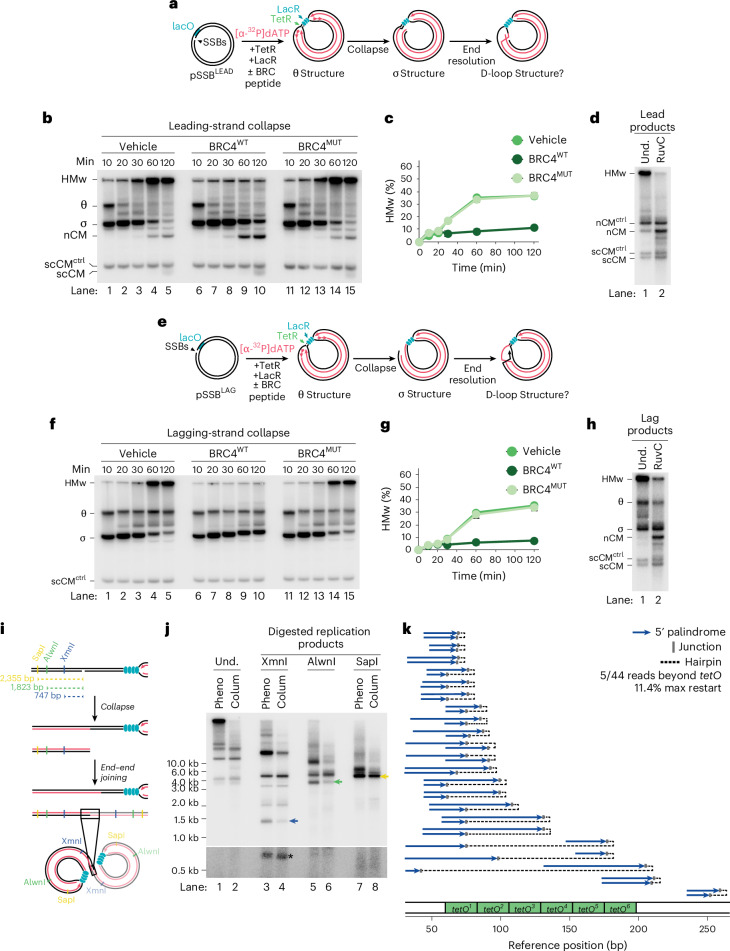
Collapsed forks undergo RAD51-dependent D-loop formation and end joining. **a**, Leading collapse was induced by replication of pSSB^LEAD^ in the presence of TetR and LacR, with vehicle, BRC4^WT^ peptide or BRC4^Mut^ peptide. BRC4^WT^ is a BRCA2-derived peptide that inhibits RAD51 binding to chromatin^67,68^. BRC4^Mut^ is a mutant peptide that does not disrupt RAD51 function and served as a negative control^67,68^. **b**, Samples from **a** were separated on an agarose gel and visualized by autoradiography. **c**, Quantification of HMw products from **b**. Data are presented as the mean ± s.d. (*n* = 3 independent experiments). **d**, Products of leading collapse were purified and digested with RuvC. Digested samples were then separated on an agarose gel and visualized by autoradiography (Extended Data Fig. 3a–e) (*n* = 5 independent experiments). **e**, Lagging collapse was induced by replication of pSSB^LAG^ as for pSSB^LEAD^ in **a**. **f**, Samples from **e** were separated and visualized as in **b**. **g**, Quantification of HMw products from **f** as in **c**. Data are presented as the mean ± s.d. (*n* = 3 independent experiments). **h**, Products of lagging collapse were purified and digested with RuvC as in **d** (Extended Data Fig. 3f–i) (*n* = 2 independent experiments). **i**, Schematic depicting expected products of end-to-end fusion events. Restriction enzymes located at different distances from the collapse site were used to map end-to-end fusion products. Digestion of end-to-end fused molecules was expected to generate linear fragments twice the length of the distance between the restriction site and the SSB. **j**, Products of leading collapse were purified with phenol–chloroform extraction to recover total DNA products or using column purification to remove HMw species (Extended Data Fig. 3j,k). Purified DNA samples were restriction-digested, separated on an agarose gel and visualized by autoradiography. Colored arrows indicate end-to-end linear fragments indicated in **i**. The asterisk indicates collapsed arm fragments (Extended Data Fig. 3j–m) (*n* = 4 independent experiments). **k**, Mapping of palindromic reads from end-to-end fusion products to the *tetO* array. Each horizontal bar represents an individual palindromic read, with the green portion indicating the 5′ palindrome sequence and dashed lines indicating hairpin regions. Black vertical lines mark the junction point of each fusion. In total, 89% of reads (39/44) mapped to the vicinity of the *tetO* array and did not extend beyond it, consistent with inefficient restart of DNA synthesis from collapsed ends (Extended Data Fig. 4). Source data

**Extended Data Fig. 3:**
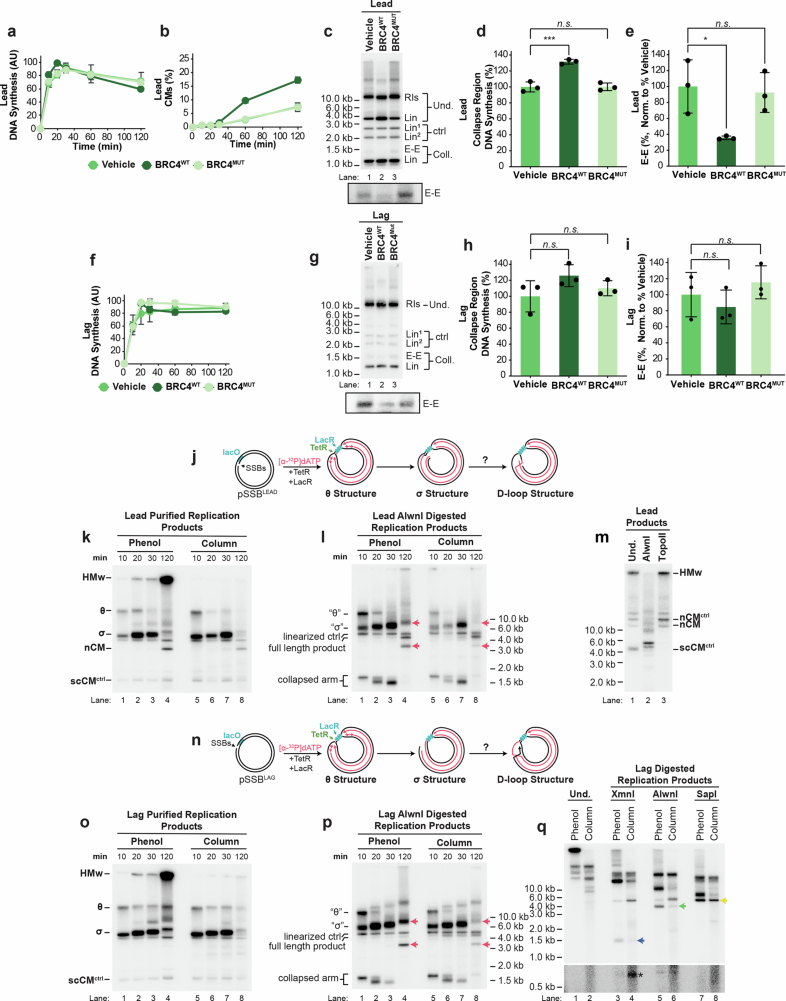
Analyses of RAD51 function during single fork collapse. (**a)** Quantification of DNA synthesis from Fig. 2b normalized to the maximum signal across all time points and conditions. Mean ± S.D., n = 3 independent experiments. (**b)** Quantification of nicked plus supercoiled circular monomers (CMs) from Fig. 2b. Mean ± S.D., n = 3 independent experiments. (**c)** Purified DNA from T = 120 in Fig. 2b was digested with XmnI and SacI to analyze collapse region synthesis. Digested samples were separated on an agarose gel and visualized by autoradiography. (**d)** Quantification of DNA synthesis in the collapse region from (c) normalized to control fragment ‘Lin^1^’. Mean ± S.D., n = 3 independent experiments. One-way ANOVA with Dunnett’s test. *** *P* = 0.0004 (**e)** Quantification of E-E fragments from (c), normalized to vehicle condition. Mean ± S.D., n = 3 independent experiments. One-way ANOVA with Dunnett’s test. * *P* = 0.0300 (**f)** Quantification of DNA synthesis from Fig. 2f normalized to the maximum signal across all time points and conditions. Mean ± S.D., n = 3 independent experiments. (**g)** Purified samples from T = 120 in Fig. 2f were digested with XmnI and SacI, then separated on an agarose gel and visualized by autoradiography. (**h)** Quantification of DNA synthesis in the collapse region from (g) normalized to control fragment ‘Lin^1^’. Mean ± S.D., n = 3 independent experiments. One-way ANOVA with Dunnett’s test. (**i)** Quantification of E-E fragments from (g), normalized to vehicle condition. Mean ± S.D., n = 3 independent experiments. One-way ANOVA with Dunnett’s test. (**j)** pSSB^Lead^ was replicated in *Xenopus* egg extract to induce leading collapse as in Fig. 1a. (**k)** Samples from (j) were purified by either phenol:chloroform extraction, to recover total DNA, or spin column purification to exclude high molecular weight DNA. Purified DNA samples were separated on an agarose gel and visualized by autoradiography. High molecular weight species were undetectable after column extraction but all other species were detected, confirming that column purification effectively excluded high molecular weight species. n = 3 independent experiments. (**l)** Samples from (j) were digested with AlwNI, then separated on an agarose gel, and visualized by autoradiography. Red arrows indicate fragments enriched in phenol:chloroform purified samples. n = 3 independent experiments. (**m)** Phenol:chloroform samples from (j) were treated with either AlwNI or Topo II then separated on an agarose gel and visualized by autoradiography. HMw species were resolved by AlwNI but not topo II, indicating they were not catenanes. n = 3 independent experiments. (**n)** pSSB^Lag^ was replicated to induce lagging collapse as in Fig. 1h. (**o)** Purified samples from (n), as in (k). (**p)** Samples from (n) digested with AlwNI, as in (l). n = 3 independent experiments. (**q)** Samples from (n) were restriction digested, separated on an agarose gel, and visualized by autoradiography. Colored arrows indicate end-to-end linear fragments from Fig. 2i. Asterisk indicates collapsed arm fragments. n = 3 independent experiments. Source data

To test the role of RAD51 in resolving leading seDSBs, we induced fork collapse in extracts containing a wild-type BRCA2-derived peptide that inhibits RAD51 binding to chromatin (BRC4^WT^) or a mutant control (BRC4^Mut^, Fig. 2a)^67,68^. RAD51 inhibition greatly reduced HMw species (Fig. 2b, lanes 6–10, Fig. 2c and Extended Data Fig. 3a) and stabilized collapsed forks (Fig. 2b, lanes 6–10), indicating that RAD51 converts collapsed forks to HMw species. CMs increased upon RAD51 inhibition (Fig. 2b, lanes 8–10, and Extended Data Fig. 3b), demonstrating that RAD51 typically suppresses these products. RAD51 inhibition did not reduce collapse region DNA synthesis (Extended Data Fig. 3c,d), supporting the conclusion that seDSB resolution does not restart replication. HMw species were resolved by RuvC treatment (Fig. 2d), indicating they contain joint molecules^69^. These junctions are consistent with D-loops, rather than reversed forks or Holliday junctions, because they arise from RAD51-dependent repair of an seDSB. Thus, following leading collapse, RAD51 converts seDSBs to joint molecules arising from D-loops and suppresses formation of full-length molecules.

RAD51 inhibition also reduced HMw species following lagging collapse (Fig. 2e–g and Extended Data Fig. 3f) and these were also RuvC sensitive (Fig. 2h). RAD51 inhibition did not reduce collapse region DNA synthesis (Extended Data Fig. 3g,h). Thus, lagging collapse also converts seDSBs to joint molecules arising from D-loops.

**Extended Data Fig. 4:**
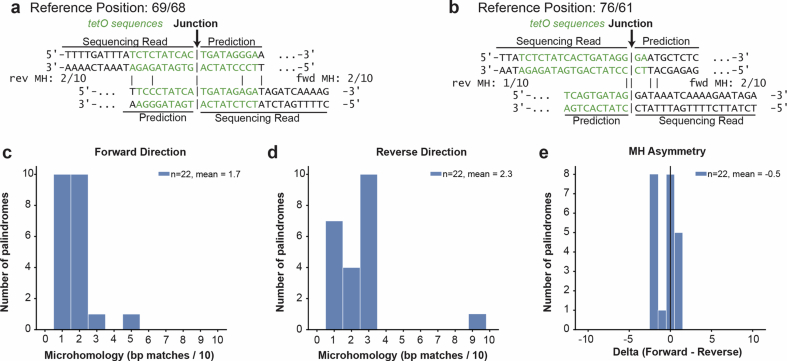
Analysis of microhomology at end-to-end fusion junctions following leading collapse. (**a)** Example of an end-to-end fusion junction detected by sequencing at reference position 69/68. The junction (indicated by the arrow) falls within a *tetO* sequence (indicated in green). Forward (fwd) and reverse (rev) microhomology (MH) scores are expressed as base pair matches per 10 bp window on each side of the junction. Sequencing reads are indicated alongside predicted junction sequences. (**b)** As in (a), a representative end-to-end fusion junction at reference position 76/61. (**c)** Distribution of forward microhomology scores across all palindromic junctions identified within the *tetO* array (n = 22, mean=1.7 bp matches/10). For visualization of junction positions refer to Fig. 2k. (**d)** Distribution of reverse microhomology scores across all palindromic junctions identified within the *tetO* array (n = 22, mean=2.3 bp matches/10). For visualization of junction positions refer to Fig. 2k. (**e)** Distribution of microhomology asymmetry across all palindromic junctions (n = 22, mean = -0.5). A central score demonstrates no significant directional bias in microhomology usage at end-to-end fusion junctions. For visualization of junction positions refer to Fig. 2k. Source data

End-to-end fusions followed leading collapse (Fig. 1f) and were suppressed by RAD51 inhibition (Extended Data Fig. 3c,e). End-to-end fusions also formed following lagging collapse but were not suppressed by RAD51 inhibition (Extended Data Fig. 3g,i). To confirm that HMw species contained end-to-end fusions, we used column purification to remove HMw products (Extended Data Fig. 3j–p). Restriction digests with enzymes located at different distances from the collapse site (Fig. 2i) produced fragments consistent with end-to-end fusions (Fig. 2j, lanes 1, 3, 5 and 7, and Extended Data Fig. 3q) that were greatly reduced upon HMw removal (Fig. 2j, lanes 2, 4, 6 and 8). These products appeared late (Extended Data Fig. 3l) and were insensitive to topoisomerase II (Extended Data Fig. 3m). Illumina sequencing of palindromic reads mapping to the vicinity of the *tetO* array (Fig. 2k) revealed approximately 2 bp of microhomology at each junction (Extended Data Fig. 4), indicating microhomology-mediated end joining (MMEJ). Only 11% of reads mapped downstream of the *tetO* array (Fig. 2k), supporting our assessment that restart following fork collapse was rare or absent. Overall, seDSBs from both leading and lagging collapse formed RAD51-dependent joint molecules and underwent end-to-end fusions involving MMEJ, with RAD51 promoting these fusions only for leading collapse.

### Leading SSBs cause secondary replication fork collapse

**Fig. 3:**
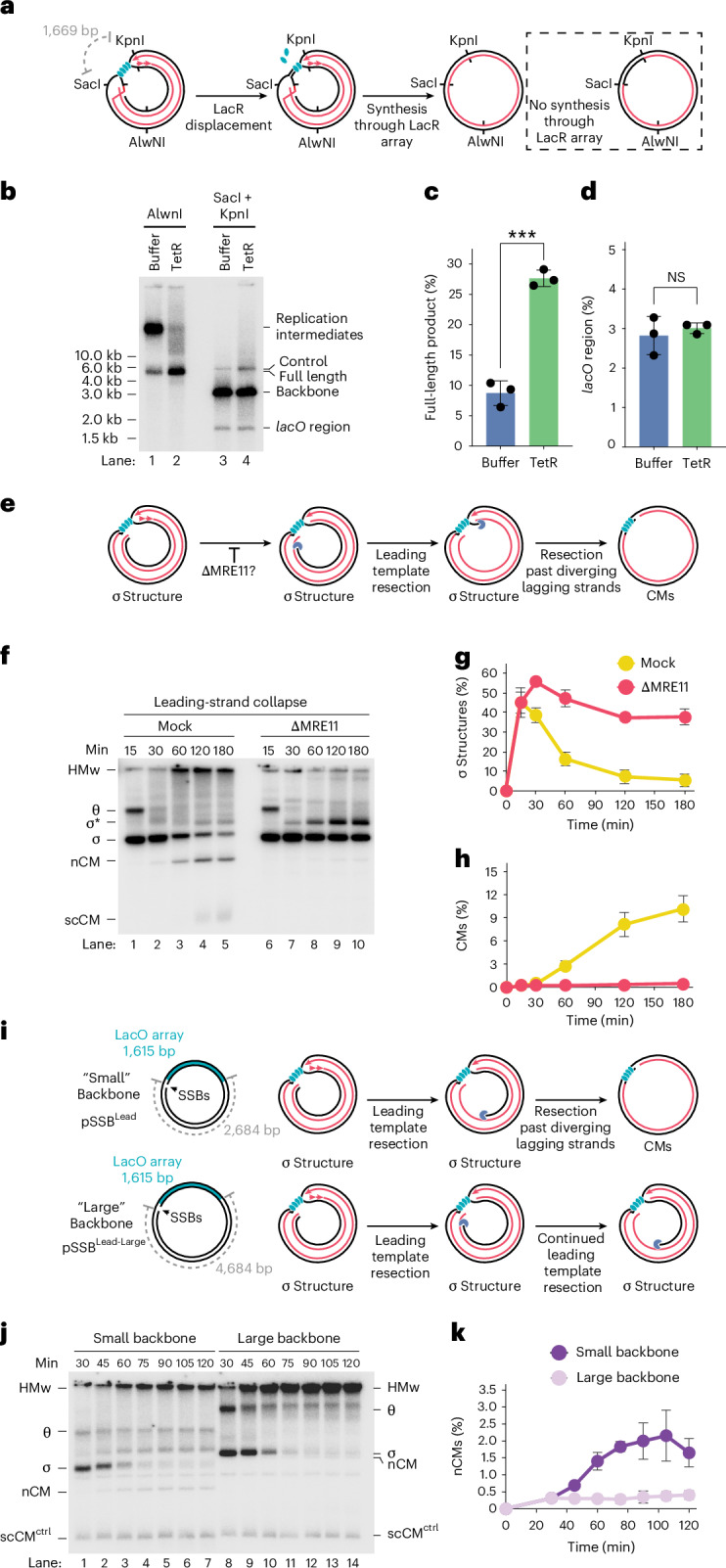
Leading seDSBs can be resolved by extensive nuclease digestion. **a**, Schematic depicting formation of full-length molecules by synthesis through the LacR array or independent of LacR array displacement. **b**, Leading collapse was induced as depicted in Fig. 1a. Samples from *t* = 120 min were then purified and analyzed by restriction digest using either AlwNI alone or SacI and KpnI combined. Digested samples were separated on an agarose gel and visualized by autoradiography. **c**, Quantification of full-length linear products arising from AlwNI digestion in **b**. Data are the mean ± s.d. (*n* = 3 independent experiments). Data were analyzed by unpaired two-sided *t*-test. ***P = 0.0002. **d**, Quantification of linearized *lacO* array fragments arising from SacI–KpnI digestion in **b**. Data are presented as the mean ± s.d. (*n* = 3 independent experiments). Data were analyzed by unpaired two-sided *t*-test. **e**, Leading collapse was induced as depicted in Fig. 1a in mock or MRE11-immunodepleted extracts. Schematic depicts generation of CMs by extensive resection of seDSBs, which would be blocked by MRE11 immunodepletion. **f**, Samples from **e** were separated on an agarose gel and visualized by autoradiography. σ* is a topoisomer of σ because the two species are converted to a single product by restriction digest (Extended Data Fig. 5b). σ species migrated more slowly over time in mock but not MRE11-immunodepleted conditions, indicating the slowed migration was because of resection—presumably because of secondary structures formed by exposed single-stranded DNA. **g**, Quantification of σ structures from **f**. Data are presented as the mean ± s.d. (*n* = 3 independent experiments). **h**, Quantification of nCMs plus scCMs from **f**. Data are presented as the mean ± s.d. (*n* = 3 independent experiments). **i**, Schematic comparing the predicted outcomes of secondary collapse for small (pSSB^LEAD^, 2,684-bp backbone) and large (pSSB^LEAD-Large^, 4684-bp backbone) plasmids. Increasing the backbone size positions the sister fork further from the collapse site, which is predicted to prevent resection from reaching the sister fork and, thus, abolish CM formation. **j**, Leading collapse was induced for pSSB^LEAD^ and pSSB^LEAD-Large^ as in Fig. 1a. Samples were separated on an agarose gel and visualized by autoradiography. **k**, Quantification of nCMs from **j**. Data are presented as the mean ± s.d. (*n* = 3 independent experiments). Source data

Leading collapse yielded full-length CMs (Fig. 1b) that were suppressed by RAD51 (Fig. 2b) and absent in lagging collapse (Fig. 1i). We initially hypothesized that these arose from enhanced progression through the LacR barrier. However, restriction digests revealed that leading collapse did not induce detectable DNA synthesis within the *lacO* array (Fig. 3a,b,d) yet produced a ~3-fold increase in full-length molecules (Fig. 3b, lanes 1 and 2, and Fig. 3c). Thus, leading collapse produced full-length molecules without synthesis through the LacR barrier.

**Extended Data Fig. 5:**
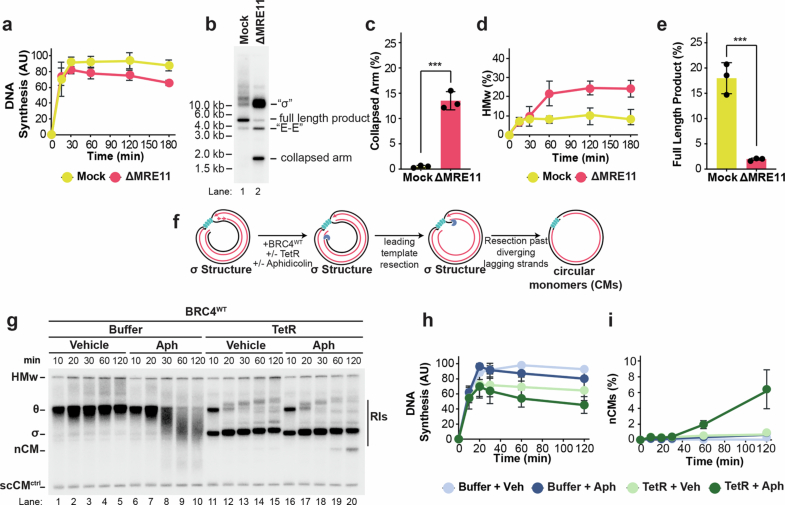
Resolution of leading, but not lagging, collapse by extensive resection. (**a)** Quantification of DNA synthesis from Fig. 3f normalized to the maximum signal across all time points and conditions. Mean ± S.D., n = 3 independent experiments. (**b)** Samples from T = 120 in Fig. 3f were purified and restriction digested with AlwNI. Digested samples were then separated on an agarose gel and visualized by autoradiography. The collapsed arm fragment was readily detectable following MRE11 depletion, indicating that degradation of the collapsed arm was blocked. (**c)** Quantification of collapsed arm products from (b). Mean ± S.D., n = 3 independent experiments. Data were analyzed by unpaired two-sided t-test. *** *P* = 0.0002 (**d)** Quantification of high molecular weight products (HMw) from Fig. 3f. Mean ± S.D., n = 3 independent experiments. (**e)** Quantification of full length products from (b). Mean ± S.D., n = 3 independent experiments. Data were analyzed by unpaired two-sided t-test. *** *P* = 0.0008 (**f)** Leading strand collapse was induced as in Fig. 1a, except extracts were supplemented with BRC4^WT^ at T = 0 and with either vehicle or aphidicolin (Aph) at T = 22.5 min. Addition of BRC4^WT^ blocks D-loop formation such that it does not compete with the secondary collapse pathway. Later addition of aphidicolin was used to specifically identify the effects of inhibited resynthesis following seDSB formation. Schematic depicts the predicted effect of aphidicolin on secondary collapse where the inhibition of resynthesis is predicted to shift the balance toward resection, promoting the enhanced formation of circular monomers (CMs). (**g)** Samples from (f) were separated on an agarose gel and visualized by autoradiography. (**h)** Quantification of total DNA synthesis from (g) normalized to the maximum signal across all timepoints and conditions. Reduced DNA synthesis in aphidicolin-treated conditions confirms effective inhibition of DNA polymerase activity, while the overall low level of nCM formation in the vehicle control compared to Fig. 1b indicates nonspecific inhibition of nCM formation by the vehicle (DMSO). Mean ± S.D., n = 3 independent experiments. (**i)** Quantification of nicked circular monomers (nCMs) from (g). Mean ± S.D., n = 3 independent experiments. Source data

We next hypothesized that extensive 5′–3′ resection removed the lagging-strand template from the sister fork, causing it to collapse^6^. This secondary collapse model predicts that blocking resection should abolish full-length molecules (Fig. 3e). MRE11 immunodepletion inhibited resection, stabilized collapsed forks (Fig. 3f,g and Extended Data Fig. 5a–c) and abolished formation of both HMw species and full-length molecules (CMs; Fig. 3f,h and Extended Data Fig. 5d,e). We also tested whether DNA polymerase activity counteracted resection during secondary collapse. Addition of aphidicolin following collapse caused a substantial increase in nCMs (Extended Data Fig. 5f–i), consistent with DNA polymerase activity guarding against resection. Increasing the size of the plasmid backbone to move the sister fork further away essentially eliminated nCMs (Fig. 3i–k). This result strongly supported the conclusion that CMs arise from resection past the sister fork. Thus, leading SSBs can trigger secondary collapse through extensive resection. Because these observations were made in *X*. *laevis* egg extracts on plasmid substrates, the frequency with which this process occurs in cells with linear chromosomes remains to be established (Discussion).

### Efficient end resolution and completion of DNA synthesis following convergent collapse

**Fig. 4:**
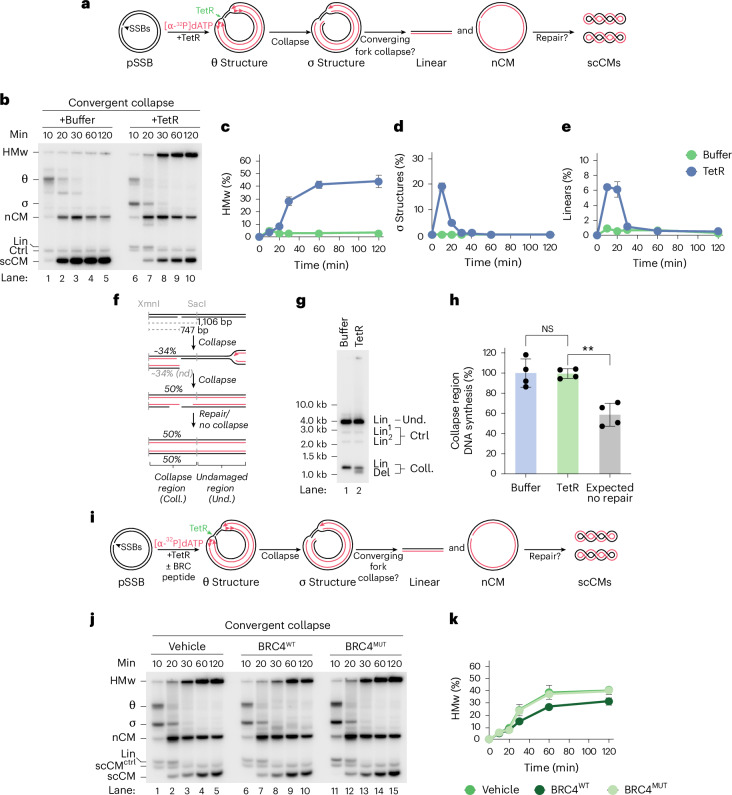
deDSBs arising from fork collapse readily complete DNA synthesis. **a**, pSSB was replicated using *X*. *laevis* egg extract in the presence of TetR (+TetR) to stabilize the SSBs. TetR was omitted in the buffer control (+buffer), which allowed religation of SSBs before replication. LacR was omitted from the reaction so that forks could converge upon the SSBs to elicit convergent collapse. Nascent strands were radiolabeled by inclusion of [α-^32^P]dATP. Fully replicated plasmid DNA (scCM^ctrl^) served as a loading control. **b**, Samples from **a** were separated on an agarose gel and visualized by autoradiography (Extended Data Fig. 6a–c). **c**, Quantification of HMw products from **b**. Data are presented as the mean ± s.d. (*n* = 4 independent experiments). **d**, Quantification of σ structures from **b**. Data are presented as the mean ± s.d. (*n* = 4 independent experiments). **e**, Quantification of linears from **b**. Data are presented as the mean ± s.d. (*n* = 4 independent experiments). **f**, Schematic depicting the assay for completion of DNA synthesis in the collapse region. **g**, Purified DNA samples from *t* = 120 min in **b** were digested with XmnI and SacI, then separated on an agarose gel and visualized by autoradiography. **h**, Quantification of collapse region DNA synthesis from **g** as in **f**. Signal was normalized to control fragment Lin^1^. Expected values for no repair account for replication efficiency and collapse efficiency. Data are presented as the mean ± s.d. (*n* = 4 independent experiments). Data were analyzed by one-way ANOVA and Tukey’s multiple-comparison method. **P = 0.0012. **i**, Convergent collapse was induced by replication of pSSB in the presence of TetR and vehicle, BRC4^WT^ or BRC4^Mut^ as a negative control. **j**, Samples from **i** were separated on an agarose gel and visualized by autoradiography. **k**, Quantification of HMw products from **j**. Data are presented as the mean ± s.d. (*n* = 3 independent experiments). Source data

**Extended Data Fig. 6:**
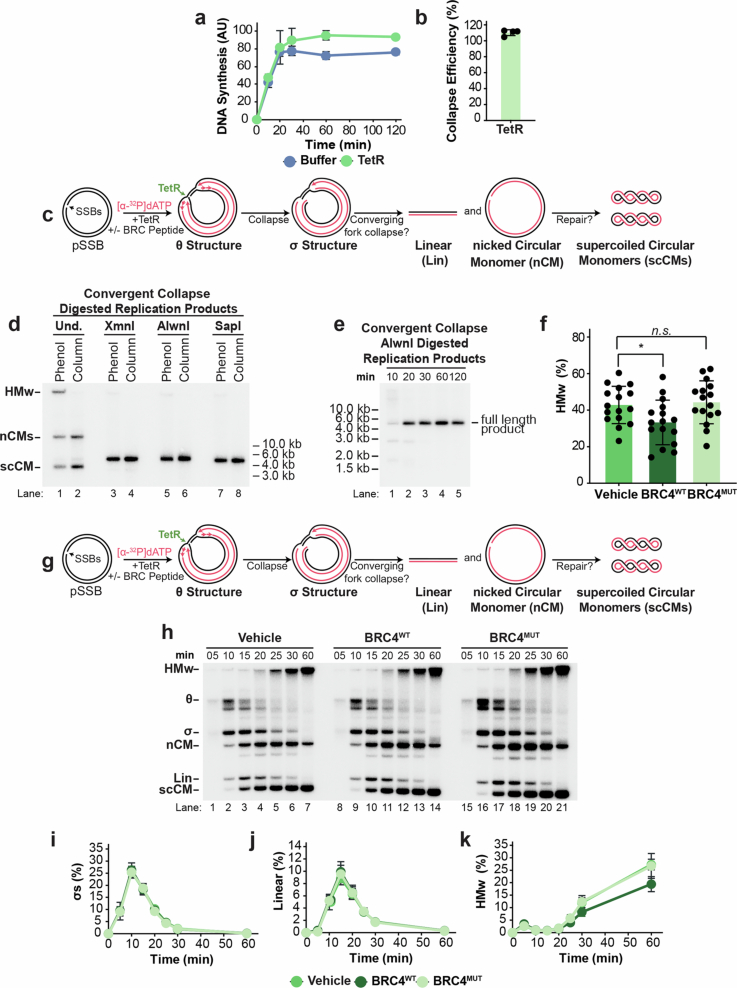
Analyses of convergent collapse in *Xenopus* egg extracts. (**a**) Quantification of DNA synthesis from Fig. 4b normalized to the maximum signal across all time points and conditions. Mean ± S.D., n = 4 independent experiments. (**b)** Quantification of collapse efficiency from Fig. 4b. Mean ± S.D., n = 4 independent experiments. Collapse efficiency was calculated as described in methods. (**c)** Convergent fork collapse was induced by replication of pSSB as in Fig. 4a. (**d)** DNA samples from T = 120 minutes in (c) were purified by either phenol:chloroform extraction, to recover total DNA, or by spin column purification to preferentially exclude high molecular weight DNA. To investigate whether erroneous end-to-end fusions were formed, purified DNA was restriction digested with XmnI, AlwNI, or SapI, which should cut pSSB only once. Products of digestion were then separated on an agarose gel. Erroneous end-to-end fusions should have resulted in products greater than and smaller than the length of full length products (4661 bp), similar to Fig. 2i-j. However, exclusively linear products were formed in all conditions and few other species were generated, indicating that end-to-end fusions were absent. n = 6 independent experiments. (**e)** pSSB was replicated as in Fig. 4b lanes 6-10. DNA intermediates were purified, digested with AlwNI, then separated on an agarose gel. Replication and collapse intermediates were readily converted to full length linear products, indicating that the ends were efficiently repaired. n = 3 independent experiments (**f)** Quantification of HMw products at T = 120 min across multiple independent experiments in Vehicle, BRC4^WT^-, and BRC4^MUT^-treated conditions. BRC4^WT^ treatment resulted in a modest but significant reduction in HMw compared to vehicle, while BRC4^MUT^ treatment did not significantly differ from vehicle. Mean ± S.D., n = 16 independent experiments. Data were analyzed by one-way ANOVA and Dunnett’s multiple-comparison method. * *P* = 0.0414 (**g)** Convergent fork collapse was induced as in Fig. 4i. A tight time course was performed to analyze the effects of BRC peptide treatment on collapsed σs and linear (Lin) products. (**h)** Convergent collapse was induced as in (g) in the presence of Vehicle, BRC4^WT^, or BRC4^MUT^. Samples were collected at the indicated timepoints, separated on an agarose gel, and visualized by autoradiography. (**i)** Quantification of σ structures from (h). Mean ± S.D., n = 3 independent experiments. (**j)** Quantification of linear products (Lin) from (h). Mean ± S.D., n = 3 independent experiments. (**k)** Quantification of high molecular weight products (HMw) from (h). Mean ± S.D., n = 3 independent experiments. Source data

We next asked whether fork convergence at an SSB (‘convergent collapse’) could trigger deDSB formation and completion of DNA synthesis. We replicated pSSB^LEAD^ without LacR to allow fork convergence (pSSB; Fig. 4a). Without TetR, SSBs were repaired and expected CM replication products formed (Fig. 4b)^6,64^. With TetR, DNA synthesis increased slightly (Extended Data Fig. 6a), possibly because of degradation and resynthesis of the parental strand. We detected linear molecules corresponding to deDSBs (Fig. 4b, lanes 6 and 7, and Fig. 4e), collapse approached 100% (Extended Data Fig. 6b) and HMw species formed as for single-fork collapse (Fig. 4c).

During convergent collapse, we also detected seDSBs (Fig. 4b, lanes 6 and 7) that peaked at 10 min and rapidly declined by 20 min (Fig. 4b,d), while deDSBs persisted from 10–20 min before declining (Fig. 4b,e). Thus, seDSBs resolved before deDSBs. seDSBs were ~2.5-fold lower during convergent collapse than during single-fork collapse (~20% in Fig. 4d versus ~50% in Fig. 1c,j), indicating rapid conversion to deDSBs by the converging fork. deDSBs were also ~3-fold less abundant than seDSBs (~6% in Fig. 4e versus ~20% in Fig. 4d), indicating that deDSBs were more readily resolved. Accordingly, deDSBs resolved by 30 min (Fig. 4e) while seDSBs from single-fork collapse did not resolve until after 60 min (Fig. 1c,j). These data provide direct evidence that convergent collapse initially forms an seDSB that the converging fork converts to a more rapidly resolved deDSB (Fig. 4a).

Collapse region DNA synthesis was indistinguishable from the control (Fig. 4f–h) and HMw products contained only full-length molecules (Extended Data Fig. 6c–e). Thus, convergent collapse enabled efficient completion of DNA synthesis through DSB repair. Inspection of the collapse region revealed small deletion products (Fig. 4g) specific to convergent collapse; end-to-end fusions arising from single-fork collapse (Figs. 1f and 2i–k) were not detected (Fig. 4g).

**Extended Data Fig. 7:**
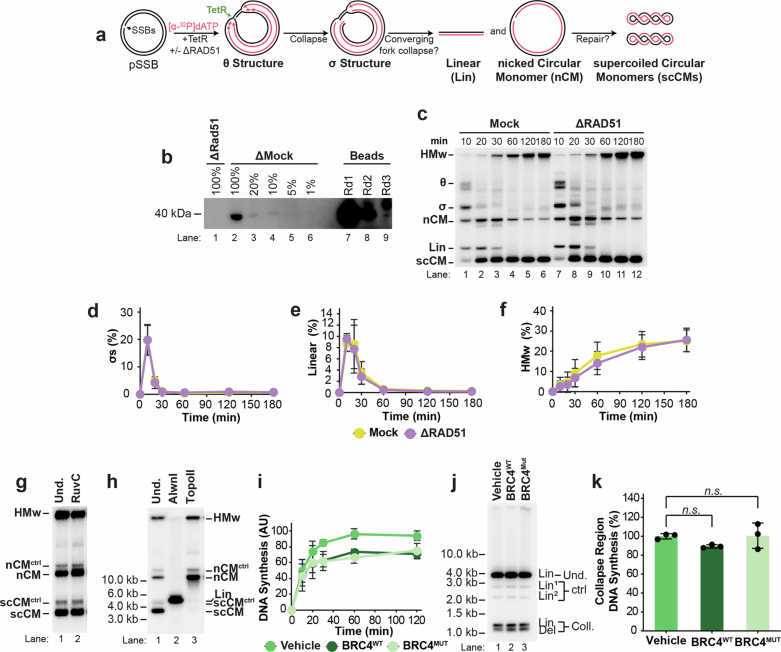
Analyses of RAD51-requirement following convergent fork collapse. (**a**) Convergent fork collapse was induced as depicted in Fig. 4a except in either mock- or RAD51-immunodepleted extracts. (**b)** Western blot confirming immunodepletion of RAD51 from Xenopus egg extract. Rd1, Rd2, and Rd3 indicate sequential rounds of depletion beads. (**c)** Convergent collapse was induced as in (a) in mock- or RAD51-immunodepleted extracts. Samples were collected at the indicated timepoints, separated on an agarose gel, and visualized by autoradiography. (**d)** Quantification of σ structures from (c). Mean ± S.D., n = 3 independent experiments. (**e)** Quantification of linear products (Lin) from (c). Mean ± S.D., n = 3 independent experiments. (**f)** Quantification of high molecular weight products (HMw) from (c). Loss of RAD51 does not impair HMw formation following convergent collapse, demonstrating that resolution of collapsed deDSBs occurs independently of RAD51. Mean ± S.D., n = 3 independent experiments. (**g)** pSSB was replicated as in Fig. 4b, lane 10, then treated with RuvC or left untreated. RuvC treatment did not resolve high molecular weight species following convergent collapse (lanes 1-2), in contrast to leading and lagging collapse in Fig. 2d, h. n = 3 independent experiments. (**h)** DNA samples were prepared and purified as in (g) then treated with either AlwNI or topo II. High molecular weight products were resolved by AlwNI but not topo II, indicating that they were not composed of catenanes. n = 3 independent experiments. (**i)** Quantification of DNA synthesis from Fig. 4j normalized to the maximum signal across all time points and conditions. Mean ± S.D., n = 3 independent experiments. (**j)** Purified samples from T = 120 of Fig. 4j were digested with XmnI and SacI to analyze DNA synthesis in the collapse region (as in Fig. 4f-h). Digested samples were separated on an agarose gel then visualized by autoradiography. (**k)** Quantification of DNA synthesis in the collapsed region from (j) normalized to control fragment ‘Lin^1^’. Mean ± S.D., n = 3 independent experiments. Data were analyzed by one-way analysis of variance (ANOVA) and Dunnett’s multiple-comparison method. No detectable effect on collapse region DNA synthesis was observed, indicating that completion of DNA synthesis occurred independently of RAD51. Source data

RAD51 inhibition decreased HMw species slightly but consistently (Fig. 4i,j, lanes 6–10, Fig. 4k and Extended Data Fig. 6f), suggesting a limited requirement for HR. It was possible that some seDSBs were rapidly resolved by RAD51 before fork convergence. To address this, we performed a fine time course (Extended Data Fig. 6g–k). We observed no difference in the rate of formation or resolution of seDSBs (Extended Data Fig. 6i), arguing against rapid HR-mediated resolution before fork convergence. The limited role for RAD51 was unlikely to reflect inefficient BRC peptide inhibition, as RAD51 immunodepletion produced the same result (Extended Data Fig. 7a–f). HMw products were not resolved by RuvC or topoisomerase treatment but were fully resolved by restriction digest (Extended Data Fig. 7g,h), indicating that they were concatemers formed by end ligation rather than D-loops. RAD51 inhibition did not affect collapse region DNA synthesis or deletion events (Extended Data Fig. 7i–k). Thus, convergent collapse led to efficient completion of DNA synthesis largely independently of HR, in contrast to other reports^3,5,8,21–26^ but consistent with the ability of HR-independent repair pathways to act at deDSBs^27,28,49–52^.

### Single and convergent forks elicit distinct outcomes following nCas9-induced collapse

**Fig. 5:**
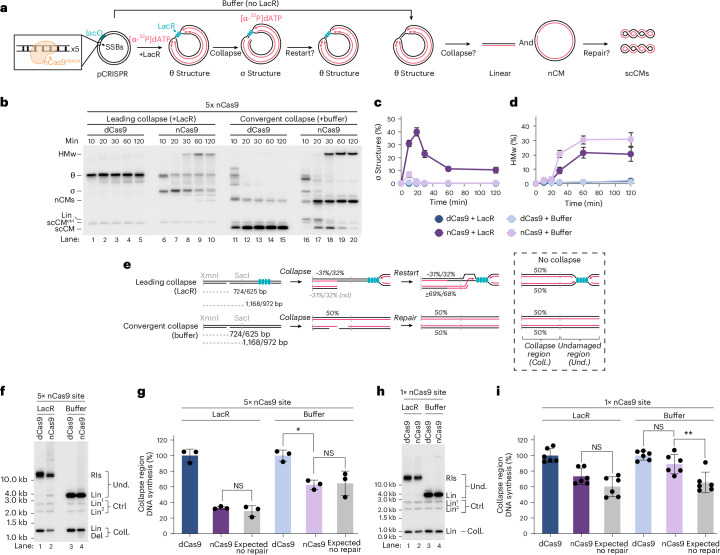
Analysis of nCas9-induced fork collapse. **a**, pCRISPR was replicated using *X*. *laevis* egg extract in the presence of nCas9 to generate SSBs. Nuclease-dead Cas9 (dCas9) did not generate SSBs and was used as a control. LacR was included to induce leading collapse or omitted to induce convergent collapse. Nascent strands were radiolabeled by inclusion of [α-^32^P]dATP. Fully replicated plasmid DNA (scCM^ctrl^) served as a loading control. **b**, Samples from **a** were separated on an agarose gel and visualized by autoradiography. **c**, Quantification of σ structures in **b**. Data are presented as the mean ± s.d. (*n* = 3 independent experiments). **d**, Quantification of HMw products in **b**. Data are presented as the mean ± s.d. (*n* = 3 independent experiments). **e**, Schematic depicting the assay for restart or completion of DNA synthesis in the collapse region. Numbers indicate the expected DNA signal for pCRISPR (used above) followed by pCRISPR^1X^ (used below). **f**, Purified DNA samples from *t* = 120 min in **b** were digested with XmnI and SacI, then separated on an agarose gel and visualized by autoradiography. **g**, Quantification of collapse region DNA synthesis from **f** as in **e**. Signal was normalized to control fragment Lin^1^. Expected values for no repair account for replication efficiency and collapse efficiency. Data are presented as the mean ± s.d. (*n* = 3 independent experiments). Data were analyzed by one-way ANOVA and Tukey’s multiple-comparison method. *P = 0.0112. **h**, Fork collapse was induced as in **a** using pCRISPR^1X^ that contained only a single copy of the nCas9 target sequence. Purified DNA samples were digested with XmnI and SacI, then separated on an agarose gel and visualized by autoradiography (Extended Data Fig. 8t). **i**, Quantification of collapse region DNA synthesis from **h** as in **e**. Signal was normalized to control fragment Lin^2^. Expected values for no repair account for replication efficiency and collapse efficiency. Data are presented as the mean ± s.d. (*n* = 6 independent experiments). Data were analyzed by one-way ANOVA and Tukey’s multiple-comparison method. **P = 0.0057. Source data

We next tested whether our findings apply to CRISPR–Cas9 SSBs using H840A Cas9 nickase (nCas9), which primarily generates seDSBs^22^. We replicated a plasmid harboring multiple nCas9 target sites adjacent to a *lacO* array (pCRISPR; Fig. 5a), with or without LacR to directly compare leading and convergent collapse.

**Extended Data Fig. 8:**
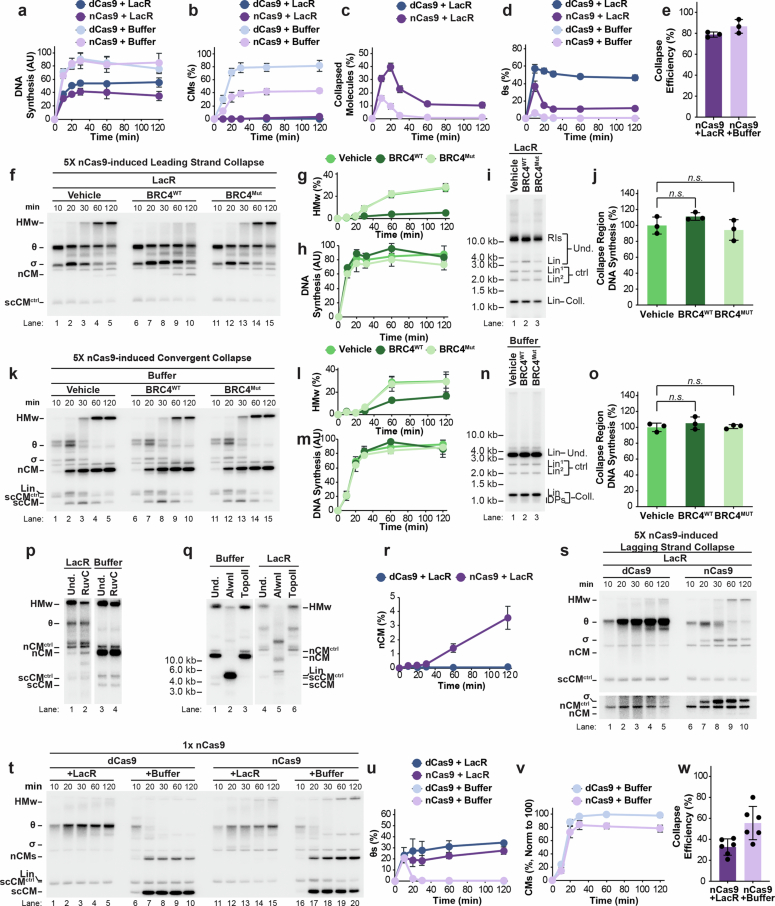
Analyses of nCas9 induced collapse in *Xenopus* egg extracts. (**a**) Quantification of DNA synthesis from Fig. 5b. Mean ± S.D., n = 3 independent experiments. (**b)** Quantification of nicked plus supercoiled circular monomers (CMs) from Fig. 5b. Mean ± S.D., n = 3 independent experiments. (**c)** Quantification of σ structures plus linear products (collapsed molecules) from Fig. 5b. Mean ± S.D., n = 3 independent experiments. (**d)** Quantification of θ structures from Fig. 5b. Mean ± S.D., n = 3 independent experiments. (**e)** Quantification of collapse efficiency (see methods) from Fig. 5b. Mean ± S.D., n = 3 independent experiments. (**f)** Leading collapse was induced using nCas9 as in Fig. 5b lanes 6-10 with vehicle, BRC4^WT^, or BRC4^Mut^ peptide. DNA samples were separated on an agarose gel and visualized by autoradiography. (**g)** Quantification of high molecular weight products (HMw) from (f). Mean ± S.D., n = 3 independent experiments. (**h)** Quantification of DNA synthesis from (f) normalized to the maximum signal across all time points and conditions. Mean ± S.D., n = 3 independent experiments. (**i)** Purified DNA samples from T = 120 in (f) were analyzed as in Fig. 5f to monitor synthesis in the collapse region. (**j)** Synthesis in the collapse region was quantified from (i) as in Fig. 5g. Mean ± S.D., n = 3 independent experiments. One-way ANOVA with Dunnett’s test. (**k)** Convergent collapse was induced using nCas9 as in Fig. 5b lanes 16-20 with vehicle, BRC4^WT^, or BRC4^Mut^ peptide. DNA samples were separated on an agarose gel and visualized by autoradiography. (**l)** Quantification of high molecular weight products (HMw) from (k). Mean ± S.D., n = 3 independent experiments. (**m)** Quantification of DNA synthesis from (k) normalized to the maximum signal across all time points and conditions. Mean ± S.D., n = 3 independent experiments. (**n)** Purified DNA samples from T = 120 in (k) were analyzed as in Fig. 5f to monitor synthesis in the collapse region. (**o)** Synthesis in the collapse region was quantified from (n) as in Fig. 5g. Mean ± S.D., n = 3 independent experiments. One-way ANOVA with Dunnett’s test. (**p)** Leading collapse and convergent collapse were induced by replicating pCRISPR. Purified DNA products (Fig. 5b, lanes 10, 20) were treated with RuvC then separated on an agarose gel and visualized by autoradiography. HMw products are mostly sensitive to RuvC for leading collapse (lanes 1-2) but mostly insensitive for convergent collapse (lanes 3-4) as observed for collapse at stabilized SSBs (Fig. 2d,h and Extended Data Fig. 7g). n = 3 independent experiments. (**q)** Purified DNA was obtained as in (p), treated with AlwNI or TopoII, separated on an agarose gel and visualized by autoradiography. HMw species were resolved by AlwNI but not topo II, indicating they were not composed of catenanes, as observed for collapse at stabilized SSBs (Extended Data Fig. 3m). n = 3 independent experiments. (**r)** Quantification of nicked circular monomers (nCM) from Fig. 5b, lanes 1-10. Mean ± S.D., n = 3 independent experiments. (**s)** Lagging collapse was induced by replication of pCRISPR as depicted in Fig. 5a but with guides targeting the lagging strand template. Replication samples were separated on an agarose gel and visualized by autoradiography. (**t)** Leading collapse was induced using nCas9^H840A^ as in Fig. 5a but using pCRISPR^1X^ containing only a single target sequence. Replication samples were separated on an agarose gel and visualized by autoradiography. (**u)** Quantification of θ structures from (t). Mean ± S.D., n = 6 independent experiments. (**v)** Quantification of nicked plus supercoiled circular monomers from (t). Mean ± S.D., n = 6 independent experiments. (**w)** Quantification of collapse efficiency (see methods) from (t). Mean ± S.D., n = 6 independent experiments. Source data

nCas9 induction of leading or convergent collapse generated seDSBs (Fig. 5b, lanes 6–10 and 16–20, and Fig. 5c) with ~80% collapse efficiency (Extended Data Fig. 8a–e), slightly lower than for TetR-stabilized SSBs (~90–100%; Extended Data Fig. 1i,q). The DNA structures closely resembled those formed at simple SSBs; leading collapse produced seDSBs converted to RAD51-dependent, RuvC-sensitive HMw species (Fig. 5b, lanes 1–10, Fig. 5c,d and Extended Data Fig. 8f–j,p,q), whereas convergent collapse produced fewer seDSBs that were converted to deDSBs and then HMw species (Fig. 5b, lanes 16–20, and Fig. 5c,d) that were only minimally affected by RAD51 inhibition (Extended Data Fig. 8k–p). nCas9 reduced CM mobility (Fig. 5b, lanes 17–20) by preventing the compensatory supercoiling that otherwise accompanies nucleosome removal during deproteinization^70^. Full-length products were generated following leading collapse (Fig. 5b, lanes 6–10) but not in the nuclease-dead control (Extended Data Fig. 8r) or following nCas9-induced lagging collapse (Extended Data Fig. 8s), indicating that secondary collapse occurred. As for collapse at simple SSBs, the frequency at which secondary collapse occurs in cells with linear chromosomes remains to be established (Discussion). Thus, the DNA structures generated by nCas9 collapse closely resembled those formed at simple SSBs.

We next monitored DNA synthesis following nCas9-induced collapse (Fig. 5e). Leading collapse did not result in detectable collapse region synthesis (Fig. 5f, lanes 1 and 2, and Fig. 5g), as expected. However, nCas9-induced convergent collapse also did not result in detectable synthesis (Fig. 5f, lanes 3 and 4, and Fig. 5g), in contrast to convergent collapse at simple SSBs (Fig. 4f–h), despite efficient DSB resolution. Because Cas9 remains associated with DSBs^71^ and inhibits their repair^72^, the multiple nCas9 target sites in pCRISPR could have interfered with completion of DNA synthesis. In support of this interpretation, seDSBs persisted longer when induced by nCas9 (Fig. 5c) than in response to simple SSBs (Fig. 1c). Therefore, we induced leading and convergent collapse at a single nCas9 target site (Fig. 5h,i). Under these conditions, leading and convergent collapse was inefficient but detectable (Extended Data Fig. 8t–w). Collapse region DNA synthesis was detected for convergent collapse but not leading collapse (Fig. 5i, buffer versus LacR). These experiments were highly variable (Fig. 5i), suggesting complex and stochastic repair events. These results support the conclusion that convergent collapsed forks can efficiently complete DNA synthesis while single collapsed forks do not.

### Convergent collapse leads to resection-dependent DSB repair

**Fig. 6:**
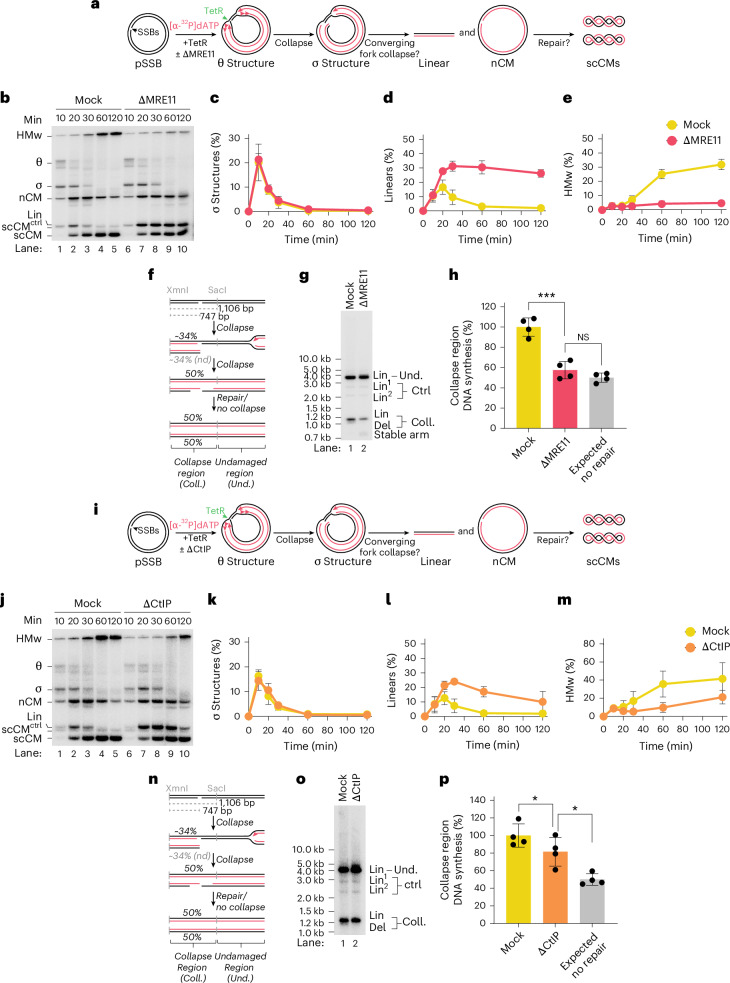
Convergent collapse requires resection for DSB repair. **a**, Schematic of convergent collapse in mock or MRE11-immunodepleted extracts. pSSB was replicated with TetR and [α-^32^P]dATP to allow fork convergence at stabilized SSBs. **b**, Samples from **a** were separated on an agarose gel and visualized by autoradiography. **c**, Quantification of σ structures from **b**. Data are presented as the mean ± s.d. (*n* = 4 independent experiments). **d**, Quantification of linear molecules (deDSBs) from **b**. Data are presented as the mean ± s.d. (*n* = 4 independent experiments). **e**, Quantification of HMw products from **b**. Data are presented as the mean ± s.d. (*n* = 4 independent experiments). **f**, Schematic of the assay for collapse region DNA synthesis following convergent collapse in mock or MRE11-immunodepleted extracts, as in Fig. 4f. **g**, Purified DNA samples from *t* = 120 min in **b** were digested with XmnI and SacI, separated on an agarose gel and visualized by autoradiography. **h**, Quantification of collapse region DNA synthesis from **g** as in **f**. Data are presented as the mean ± s.d. (*n* = 4 independent experiments). Data were analyzed by paired two-sided *t*-test. ***P = 0.0004. **i**, Schematic of convergent collapse in mock or CtIP-immunodepleted extracts as in **a**. **j**, Samples from **i** were separated on an agarose gel and visualized by autoradiography. **k**, Quantification of σ structures from **j**. Data are presented as the mean ± s.d. (*n* = 4 independent experiments). **l**, Quantification of linear molecules (deDSBs) from **j**. Data are presented as the mean ± s.d. (*n* = 4 independent experiments). **m**, Quantification of HMw products from **j**. Data are presented as the mean ± s.d. (*n* = 4 independent experiments). **n**, Schematic of the assay for collapse region DNA synthesis following convergent collapse in mock or CtIP-immunodepleted extracts as in **f**. **o**, Purified DNA samples from *t* = 120 min in **j** were digested and visualized as in **g**. **p**, Quantification of collapse region DNA synthesis from **o** as in **h**. Data are presented as the mean ± s.d. (*n* = 4 independent experiments). Data were analyzed by paired two-sided *t*-test (Extended Data Fig. 9). *P = 0.0150 and 0.0101. Source data

**Extended Data Fig. 9:**
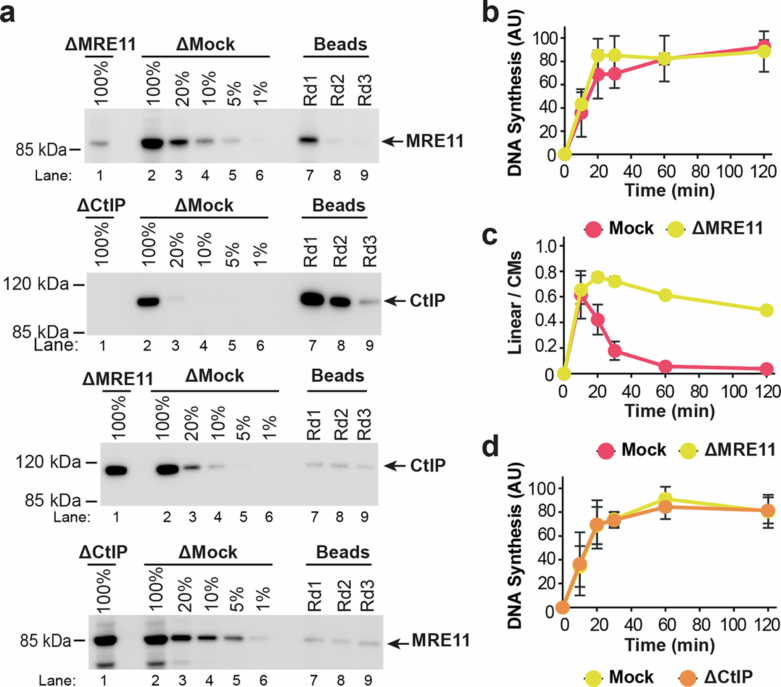
Analyses of MRE11 and CtIP immunodepletion. (**a**) Western blots confirming independent immunodepletion of MRE11 and CtIP from Xenopus egg extract. Rd1, Rd2, and Rd3 indicate sequential rounds of depletion beads. Reciprocal Western blotting for MRE11 and CtIP revealed that the proteins were not co-depleted. (**b)** Quantification of DNA synthesis from Fig. 6b. Mean ± S.D., n = 4 independent experiments. (**c)** Quantification of the ratio of linear products ‘Lin’ to nicked plus supercoiled circular monomers (CMs). (**d)** Quantification of DNA synthesis from Fig. 6j. Mean ± S.D., n = 4 independent experiments. Source data

We next tested whether resection was required for repair following convergent collapse. We examined fork collapse in mock and MRE11-immunodepleted extracts (Fig. 6a and Extended Data Fig. 9a,b). Resolution of seDSBs was unaffected by MRE11 depletion (Fig. 6b,c), consistent with conversion to deDSBs by fork convergence. In contrast, deDSBs persisted for essentially the entire time course (Fig. 6b,d) and HMw products were abolished (Fig. 6b,e). Persistence of deDSBs but unaltered seDSB resolution strongly supported our conclusion that most seDSBs are resolved by fork convergence rather than HR. The ratio of deDSBs to fully replicated molecules approached 1:1 (Extended Data Fig. 9c), as expected if collapse generated one deDSB and one fully replicated molecule that were both stable. When we directly examined repair, there was no detectable repair in MRE11-immunodepleted extracts (Fig. 6f–h). Thus, MRE11 immunodepletion abolished detectable repair following convergent collapse.

To test whether this reflected a specific role for the early stages of resection, we immunodepleted CtIP (Fig. 6i and Extended Data Fig. 9a,d). The resolution of seDSBs was unaffected (Fig. 6j,k), as for MRE11 depletion. deDSBs persisted (Fig. 6j,l) and HMw products were reduced (Fig. 6j,m). However, in contrast to MRE11 depletion, deDSBs were gradually resolved (60–120 min; Fig. 6j,l), HMw products were eventually produced (60–120 min; Fig. 6j,m) and repair was only modestly diminished (Fig. 6n–p). Codepletion controls confirmed that MRE11 and CtIP immunodepletions targeted distinct components of the initial resection machinery (Extended Data Fig. 9a). Thus, the initial resection machinery is important for deDSB repair following convergent collapse, with MRE11 having a more crucial role than CtIP.

### Convergent collapse generates error-prone repair products distinct from single-fork collapse

**Fig. 7:**
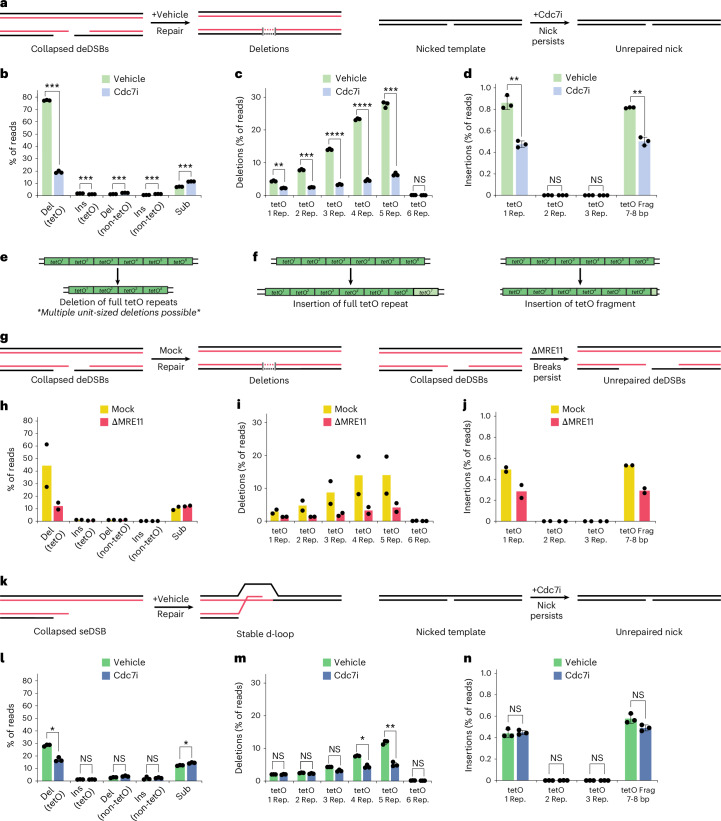
Convergent collapse generates error-prone repair products distinct from single-fork collapse. **a**, Schematic of convergent collapse repair products. Replication of pSSB in the presence of TetR (vehicle) generates deDSBs that undergo repair, producing accurate products and deletions. In the no-replication control (Cdc7i), SSBs remain as unrepaired nicks. **b**, Frequency of mutation classes following convergent collapse (vehicle) compared to the no-replication control (Cdc7i), determined by Illumina sequencing. Ins, insertions; Del, deletions; Sub, substitutions. Data are presented as the mean ± s.d. (*n* = 3 independent experiments). Data were analyzed by two-sided Welch’s *t*-test with Benjamini–Hochberg multiple-comparison correction. ***P = 2.47 × 10^−5^, 3.68 × 10^−4^, 5.91 × 10^−5^, 2.14 × 10^−5^ and 3.50 × 10^−5^. **c**, Frequency of precise *tetO* repeat deletions (1–5 repeats (Rep.)) from **b**. Data are presented as the mean ± s.d. (*n* = 3 independent experiments). Data were analyzed by two-sided Welch’s *t*-test with Benjamini–Hochberg multiple-comparison correction. **P = 5.38 × 10^−4^, ***P = 8.30 × 10^−5^ and 4.59 × 10^−5^ and ****P = 6.38 × 10^−7^ and 3.44 × 10^−7^. **d**, Frequency of templated insertions (full *tetO* repeat, 2–3 repeats and 7–8-bp *tetO* fragment) from **b**. Data are presented as the mean ± s.d. (*n* = 3 independent experiments). Data were analyzed by two-sided Welch’s *t*-test with Benjamini–Hochberg multiple-comparison correction (Extended Data Fig. 10). **P = 3.04 × 10^−3^ and 4.31 × 10^−3^. **e**, Schematic of deletion events involving removal of one or more full *tetO* repeats from the array. **f**, Schematics of insertion events: insertion of a full *tetO* repeat (left) and insertion of a *tetO* fragment (right). **g**, Schematic of convergent collapse in mock or MRE11-immunodepleted extracts. MRE11 depletion is predicted to block resection and prevent repair, stabilizing unrepaired deDSBs. **h**, Frequency of mutation classes following convergent collapse in mock or MRE11-immunodepleted extracts as in **b** (*n* = 2 independent experiments). **i**, Frequency of precise *tetO* repeat deletions from **h** as in **c** (*n* = 2 independent experiments). **j**, Frequency of templated insertions from **h** as in **d** (*n* = 2 independent experiments). **k**, Schematic of leading collapse repair products. Replication of pSSB^LEAD^ in the presence of TetR and LacR (vehicle) generates seDSBs that form stable D-loops. In the no-replication control (Cdc7i), SSBs remain as unrepaired nicks. **l**, Frequency of mutation classes following leading collapse compared to the no-replication control, as in **b**. Data are the mean ± s.d. (*n* = 3 independent experiments). Data were analyzed by two-sided Welch’s *t*-test with Benjamini–Hochberg multiple-comparison correction. *P = 2.28 × 10^−3^ and 4.99 × 10^−3^. **m**, Frequency of precise *tetO* repeat deletions following leading collapse from **l** as in **c**. Data are presented as the mean ± s.d. (*n* = 3 independent experiments). Data were analyzed by two-sided Welch’s *t*-test with Benjamini–Hochberg multiple-comparison correction. *P = 4.59 × 10^−3^ and **P = 2.36 × 10^−4^. **n**, Frequency of templated insertions following leading collapse from **l** as in **d**. Data are presented as the mean ± s.d. (*n* = 3 independent experiments). Data were analyzed by two-sided Welch’s *t*-test with Benjamini–Hochberg multiple-comparison correction. In all panels, *P* values are listed in order of appearance, from left to right. Source data

**Extended Data Fig. 10:**
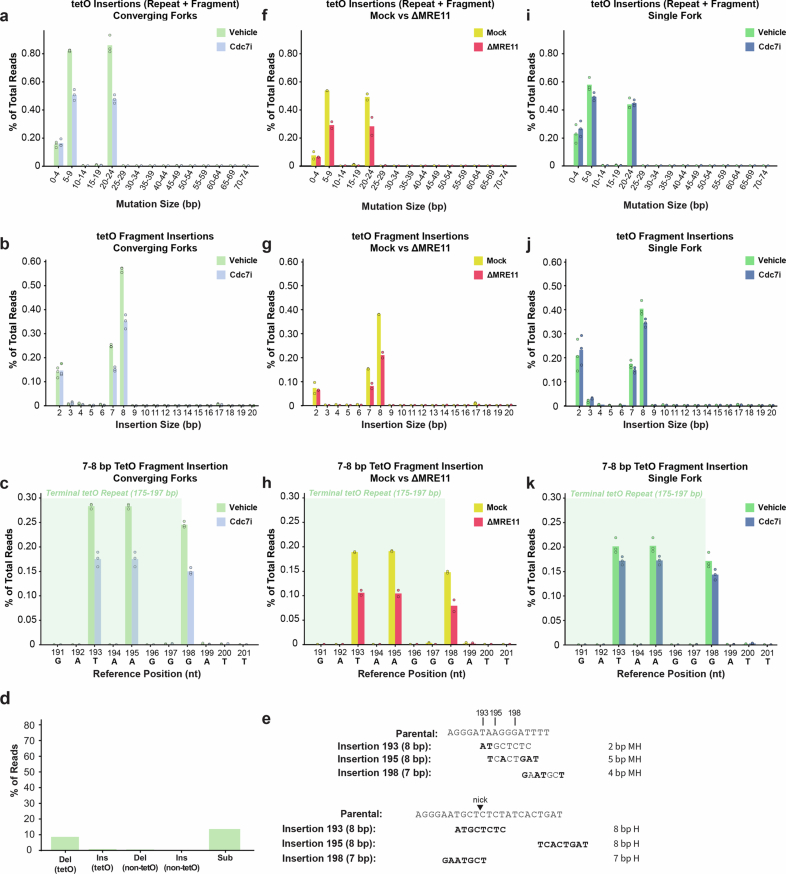
*tetO* and *tetO* fragment insertions arising from fork collapse. (**a)** Size distribution of *tetO* insertions (repeat + fragment) at repair junctions following convergent fork collapse in vehicle- or Cdc7i-treated conditions, expressed as a percentage of total sequencing reads. Mean ± S.D., n = 3 independent experiments. (**b)** Size distribution of *tetO* fragment insertions at repair junctions following convergent fork collapse in vehicle- or Cdc7i-treated conditions. Insertions of 7-8 bp are the predominant fragment size across conditions. Mean ± S.D., n = 3 independent experiments. (**c)** Mapping of 7-8 bp *tetO* fragment insertions to the terminal *tetO* repeat (highlighted in green) at repair junctions following convergent fork collapse in vehicle- or Cdc7i-treated conditions. Insertions cluster predominantly at reference positions 193, 195, and 198, consistent with microhomology-mediated templating from the terminal tetO repeat. Mean ± S.D., n = 3 independent experiments. (**d)** Frequency of mutation classes for the parental plasmid used in Fig. 7. Abbreviations: Ins, insertions; Del, deletions; Sub, substitutions. (**e)** Examples of *tetO* fragment insertions detected by amplicon sequencing at repair junctions following convergent fork collapse. Three insertion events (at reference positions 193, 195, and 198) are shown mapped to two distinct parental sequence contexts: the non-nick region (top) and the nick-proximal region (bottom). Microhomology (MH) and homology (H) associated with each insertion event is indicated. Bold nucleotides indicate the homologous bases. (**f)** Size distribution of total *tetO* insertions (repeat + fragment) at repair junctions following convergent fork collapse in mock- or MRE11-immunodepleted extracts. Mean ± S.D., n = 2 independent experiments. (**g)** Size distribution of *tetO* fragment insertions at repair junctions following convergent fork collapse in mock- or MRE11-immunodepleted extracts. Mean ± S.D., n = 2 independent experiments. (**h)** As in (c), for repair junctions following convergent fork collapse in mock- or MRE11-immunodepleted extracts. Mean ± S.D., n = 2 independent experiments. (**i)** Size distribution of total *tetO* insertions (repeat + fragment) at repair junctions following single fork collapse in vehicle- or Cdc7i-treated conditions. Mean ± S.D., n = 3 independent experiments. (**j)** Size distribution of *tetO* fragment insertions at repair junctions following single fork collapse in vehicle- or Cdc7i-treated conditions. Mean ± S.D., n = 3 independent experiments. (**k)** As in (c), for repair junctions following single fork collapse in vehicle- or Cdc7i-treated conditions. Mean ± S.D., n = 3 independent experiments. Source data

To characterize repair products, we sequenced the outcome of convergent collapse (Fig. 7a–d and Extended Data Fig. 10a–c) compared to a no-replication control (Cdc7i). The major replication-dependent product involved precise deletions corresponding to removal of 1–5 *tetO* repeats (Fig. 7b,c,e). Furthermore, 77.3% of reads contained deletions, which exceeded the ~50% maximum expected if all repair events produced mutations. The excess deletions arose from replication-independent deletions (19.2% for Cdc7i; Fig. 7b), including preexisting deletions in the parental plasmid (8.7%; Extended Data Fig. 10d). Importantly, the 58.1% frequency of replication-dependent deletions was similar to the expected ~50% maximum. We also observed ~10% substitutions but these increased upon Cdc7i treatment (Fig. 7b), indicating that they were replication independent. Deletions were skewed toward larger sizes; we never observed deletion of all six repeats, indicating that the process was homology dependent (Fig. 7c). The precise nature of these junctions, involving exact removal of the 23-bp repeating unit, meant that we could not determine whether repair used the full 23 bp of homology or a shorter sequence within each repeat.

Templated insertions were also detected at low frequency, involving either one complete *tetO* repeat or a 7–8-bp *tetO* fragment at sites of 2–5-bp microhomology (Fig. 7d,f and Extended Data Fig. 10a–c,e). These insertions are characteristic of MMEJ^73^. Both deletions and insertions were diminished by MRE11 immunodepletion (Fig. 7g–j and Extended Data Fig. 10f–h). Combined with the resection dependence and RAD51 independence of convergent collapse repair (Figs. 4–6), these data indicate that repair proceeds through annealing-dependent DSB repair (single-strand annealing or alternative end joining)^74^.

We next sequenced products from leading collapse (Fig. 7k–n) to test whether convergent collapse products were also detected in this setting. Leading collapse produced replication-dependent *tetO* deletions at greatly reduced frequency (~11.4%; Fig. 7l). Smaller deletions (1–3 repeats) were not detected and larger deletions (4–5 repeats) were diminished compared to convergent collapse (Fig. 7c,m). Templated insertions were not detected (Fig. 7n and Extended Data Fig. 10i–k). The lack of insertions and deletions was not because of these mechanisms forming palindromes, which represented only 0.023% ± 0.006% (mean ± s.d.) of total reads. This difference in mutation profiles demonstrates that semisynchronous convergent collapse produces repair outcomes distinct from single collapsed forks.

## Discussion

**Fig. 8:**
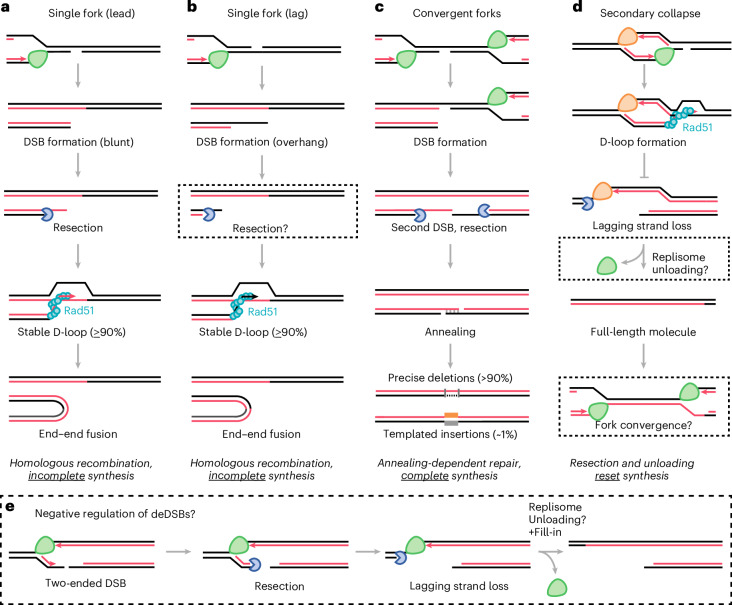
Models for repair outcomes following replication fork collapse at SSBs in *X*. *laevis* egg extracts. **a**, Leading collapse (single fork). An SSB on the leading-strand template generates a blunt seDSB upon fork encounter. Resection enables RAD51-dependent strand invasion to form a stable D-loop that does not support restart of DNA synthesis. RAD51 promotes end-to-end fusions at leading collapse (‘Discussion’). End-to-end fusions involving microhomology are an alternative, erroneous outcome. Both pathways result in HR-dependent DSB resolution without restart of DNA synthesis. **b**, Lagging collapse (single fork). An SSB on the lagging-strand template generates an seDSB with a 3′ overhang. The seDSB is similarly converted to a stable D-loop, although whether resection is required was not directly addressed in this study (dashed box). End-to-end fusions are also formed, possibly because RAD51 binding to the preresected 3′ overhang does not inhibit resection of the opposing strand as effectively as RAD51 binding to blunt ends. **c**, Convergent forks. When a converging fork arrives at an SSB following initial seDSB formation, a second DSB is generated to produce a deDSB. The deDSB undergoes resection and is repaired largely independently of RAD51 through end joining, enabling efficient completion of DNA synthesis. Templated insertions and precise deletions are error-prone outcomes of this repair process. **d,** Secondary collapse. In the context depicted here, one replisome has stalled near the sister fork (as in Fig. 3). Following leading collapse, extensive 5′–3′ resection of the seDSB extends past the sister fork, removing the lagging-strand template and triggering replisome unloading to generate a full-length molecule. Fork convergence from adjacent origins may then allow replication of the collapse region. D-loop formation at the seDSB inhibits secondary collapse by blocking resection. Whether replisome unloading occurs and whether fork convergence completes replication of the collapse region were not directly addressed in this study (dashed boxes). **e**, Negative regulation of deDSBs. The secondary collapse mechanism may explain why certain types of lagging-strand nicks (for example, nCas9 (H840A) in Fig. 5 and a previous study^22^ or Tet-nick in Fig. 1) do not generate deDSBs. Following deDSB formation at a lagging-strand nick, 5′–3′ resection could degrade the 5′ flap generated by unwinding past the nick, converting the deDSB into an seDSB. Continued resection past the sister fork triggers secondary collapse, leading to replisome unloading and fill-in synthesis to produce a full-length molecule. Whether replisome unloading occurs in this context was not directly addressed in this study (dashed box).

We interrogated replication fork collapse in *X*. *laevis* egg extracts using both simple SSBs and nCas9 (H840A)-generated SSBs. DSBs were efficiently resolved regardless of whether a single fork collapsed at a leading-strand or lagging-strand SSB or whether convergent forks collapsed (Fig. 8a–c). We provide direct evidence that fork convergence at an SSB involves formation of an seDSB that is converted to a deDSB (Figs. 4 and 5), consistent with previous results^21–23,27^. Single collapsed forks formed stable joint molecules arising from D-loops without detectable restart of DNA synthesis (Fig. 8a,b), while semisynchronous convergent collapse led to efficient completion of DNA synthesis independently of RAD51, generating precise deletions and templated insertions consistent with MMEJ (Fig. 8c). These findings parallel recent work^75^, where fork collapse in bacteria engages HR to form D-loops that cannot efficiently restart DNA synthesis alone without replicative helicase reloading.

Our data support the conclusion that the dominant outcome of single-fork collapse is a stable D-loop that does not restart DNA synthesis (Fig. 8a,b). Our inability to detect restart is unlikely to reflect synchrony or density, as high-density fork collapse in human cells does not noticeably alter the response^22^. Across DNA synthesis measurements (~9%; Extended Data Fig. 1u) and sequencing analysis (~11%; Fig. 2k), our data support an upper bound of ~10% restart likely because of low levels of convergent collapse (Extended Data Fig. 2n–r). Our inability to detect BIR is consistent with evidence that most fork collapse events involve deDSB formation^21–23,42,43^ and that BIR proteins do not appear to be major determinants of PARP inhibitor cytotoxicity^76^. BIR may be more critical when fork convergence is not possible, such as during mitotic DNA synthesis or alternative lengthening of telomeres^37–41^. However, we cannot exclude unique properties of our in vitro system.

seDSBs formed RAD51-dependent joint molecules that persisted for hours (Figs. 1, 2 and 8a,b), while deDSBs arising from semisynchronous convergent collapse were repaired largely independently of RAD51 (Figs. 5 and 8c and Extended Data Fig. 7). Our in vitro findings differ from cellular findings that collapse repair depends critically on HR^21,23,42^. *X*. *laevis* egg extracts are proficient for HR (Fig. 1a,b)^68^ but induce convergent collapse with relatively high synchrony between the two collapse events (Fig. 4). Our favored explanation is that the synchrony with which convergent forks collapse has a role, with more synchronous collapse favoring HR-independent pathways. We consistently observed an approximately 10% reduction in HMw species following convergent collapse in the presence of BRC peptide (Extended Data Fig. 6f), indicating that HR can operate but is limited to a minor role. In fission yeast, the balance between BIR and end joining following fork collapse is shifted toward BIR when arrival of the converging fork is delayed^27^. Additionally, end joining is well documented following fork collapse at a trapped TOP1 complex^28,50–52^, where TOP1 negatively regulates collapse of single forks and favors synchronous collapse of converging forks^43^. Lastly, enzymatic deDSBs, involving synchronous formation of two DNA ends, can be repaired by end joining throughout the S phase^46–48^. It will be important to determine whether the timing of fork convergence influences DSB repair pathway choice.

Convergent collapse produced two classes of error-prone products (Fig. 8c). Templated insertions (~1%; Fig. 7d) are characteristic of MMEJ^73^. The precise deletions (~99%; Fig. 7b) could arise from either MMEJ^77^ or SSA (single-strand annealing)^78^. SSA is generally thought to act on longer homologies but MMEJ can readily generate deletions exceeding 100 bp when canonical nonhomologous end joining (c-NHEJ) is unable to operate^79^. This condition applies in our system, where lagging collapse generates an extended 3′ overhang that should inhibit c-NHEJ. Therefore, we are unable to determine whether the deletions arose through MMEJ, SSA or both. However, SSA and MMEJ are increasingly recognized as a single mechanistic class distinguished primarily by homology length^74,80–82^; therefore, we conclude that the repair we observe is exclusively annealing-dependent DSB repair^74^.

We are not aware of other examples of precise deletions arising from annealing-dependent DSB repair following fork collapse (Fig. 7). Similar deletions can arise following fork collapse in bacteria through a recombination-dependent mechanism^83^ and in human cells following enzymatic DSB induction^35,84^, demonstrating that collapse-driven deletions can occur in vivo. This annealing-dependent pathway could support programmed deletions in a chromosomal context and reduce faithful repair that otherwise limits DSB-based genome editing^85^.

End-to-end fusions arising from single-fork collapse occurred at sites of microhomology (Fig. 2k and Extended Data Fig. 4) and were diminished by RAD51 inhibition for leading collapse (Extended Data Fig. 3e and Fig. 8a) but not for lagging collapse (Extended Data Fig. 3i and Fig. 8b). RAD51 can suppress MMEJ through its nonenzymatic DNA binding^86^. We speculate that RAD51 promotes these fusions through its dsDNA-binding activity, which may limit resection and facilitate end joining at the blunt DNA end generated by leading collapse. For lagging collapse, RAD51 would not be expected to influence end joining because the native 3′ overhang generated at the moment of collapse can directly anneal and undergo MMEJ without further exonucleolytic processing.

Our data also reveal that leading collapse can trigger secondary collapse through extensive resection that disassembles the sister fork (Fig. 8d). We propose that 5′–3′ resection removes the lagging-strand template of the sister fork, triggering replisome removal through the termination pathway^6^. Secondary collapse is, thus, specific to leading collapse; lagging collapse 5′–3′ resection targets the nascent rather than parental strands. Secondary collapse disassembles the replication bubble, allowing converging forks from adjacent origins to replicate the region. Secondary collapse was abolished when the sister fork was moved further away (Fig. 3i–k) and enhanced by RAD51 inhibition (Fig. 2b), suggesting that it could operate when the sister fork stalls after origin firing (Fig. 3) and be promoted by HR deficiency^22,87^. This mechanism could also convert deDSBs arising from lagging collapse (as described previously^21–23^) into seDSBs (Fig. 8e) and may explain why deDSBs are not detected for certain types of lagging-strand nicks (for example, nCas9 (H840A) in Fig. 5 and a previous study^22^ or Tet-nick in Fig. 1). Importantly, our results demonstrate secondary collapse in *X*. *laevis* egg extracts but not in cells. A mechanistically similar process was recently described in bacteria^88^, supporting the idea that secondary collapse can also occur in cells. However, the frequency with which it operates in eukaryotic cells with linear chromosomes and multiple origins remains to be established.

Overall, our findings establish that strand specificity of SSBs and fork convergence can determine which repair pathways operate, with implications for understanding genome instability downstream of replication stress, PARP inhibitor therapy and genome engineering.

## Methods

### Statistics and reproducibility

Experimental repeats and statistical analyses are provided in figure legends. Where not specified, experiments were performed independently two times. Sequencing data were analyzed using SciPy (version 1.17.0) and statsmodels (version 0.14.6) in Python (version 3.12.10); all other analyses used GraphPad Prism (version 10). Significance is indicated in figures (NS, not significant). No statistical method was used to predetermine sample size. No data were excluded from the analyses. Experiments were not randomized. The investigators were not blinded to allocation during experiments and outcome assessment.

### *X*. *laevis* egg extracts

*X*. *laevis* egg extracts (‘*Xenopus* egg extracts’) were prepared from WT *X*. *laevis* male and female frogs, as described previously^91^. Animal protocols were approved by the Vanderbilt Division of Animal Care and the Institutional Animal Care and Use Committee.

### Plasmid construction

Commercially available plasmid pET-28b(+) (Novagen), referred to as ‘ctrl’ (pJD1), served as an internal loading control in indicated experiments. Plasmids pSSB^LEAD^, pSSB^LAG^, pCRISPR, pSSB^LEAD 686 bp^ and pSSB^LEAD-Large^ are modified derivatives of pJD161. Briefly, pJD161 contains 50× tandem repeats of the *lacO* sequence that collectively form a ~1,600-bp *lacO* array. Details on pJD161 construction were published previously^60^. To create pSSB^LEAD^ and pSSB^LAG^, blunt-ended DNA duplex JDD27 (Supplementary Table 1) was cloned into pJD161 that was linearized with PsiI (New England Biolabs (NEB)). JDD27 contained 5× tandem Nb.BsmI sites flanked by a single *tetO* sequence on either side. Blunt-end ligation allowed insertion of JDD27 in both forward and reverse orientations. Ligation products were transformed into DH5α cells and constructs were selected such that digestion with Nb.BsmI would nick either the top (pSSB^LAG^) or bottom (pSSB^LEAD^) strand.

Plasmids pSSB^LEAD 686 bp^ and pSSB^LEAD-Large^ were constructed from pSSB^LEAD^. Plasmid pSSB^LEAD-Large^ differs from pSSB^LEAD^ by the addition of a 2.0-kb insert ‘IS1’ (Supplementary Table 1) placed 330 bp downstream of the *lacO* array, on the opposite side of the *tetO* array. pSSB^LEAD 686 bp^ differs from pSSB^LEAD^ by the addition of a 300 bp insert ‘IS2’ (Supplementary Table 1) placed 304 bp downstream of the *tetO* array, placing the location of this insert between the *tetO* and *lacO* arrays. Both plasmids pSSB^LEAD 686 bp^ and pSSB^LEAD-Large^ were constructed by GenScript.

To create pCRISPR, DNA oligonucleotides JDO143 and JDO144 (Supplementary Table 1) were annealed and cloned into pJD161 that was linearized using PsiI (NEB). Constructs were chosen that contained JDO144 in the top strand and JDO143 in the bottom strand in a 5′–3′ orientation. pCRISPR contained 5× tandem repeats of the target sequences (5′-GGTTGAGTGTTGTTCCAGTT-3′) and (5′-AGATAGGGTTGAGTGTTGTT-3′), targeted by nCas9 (H840A) to generate SSBs in the leading and lagging strand, respectively. Plasmids and antibodies generated in this study are available from the corresponding author upon reasonable request.

### Protein purification

TetR and LacR were expressed in T7 Express *Escherichia coli* (NEB). His-tagged TetR was purified by Ni-NTA affinity chromatography and dialyzed. Biotinylated LacR was extracted from the insoluble cell fraction, subjected to polymin P precipitation and ammonium sulfate fractionation, purified on SoftLink monomeric avidin resin (Promega) and dialyzed, as described previously^64^.

### Preparation of damaged plasmid templates

To generate SSB-containing plasmid (for pSSB^LEAD^, pSSB^LAG^, pSSB^LEAD 686 bp^ and pSSB^Lead-Large^), 30 μg of plasmid was digested with 75 U of Nb.BsmI (NEB) in 1× NEBuffer r3.1 (NEB) for 1 h 45 min at 37 °C. Nicked DNA was then resolved on a 0.9% agarose gel at 5 V cm^−1^ and stained with SYBR gold (Invitrogen). The nicked DNA band was excised using a blue-light transilluminator and purified by electroelution. Electroelution was performed by placing the excised gel slice in SnakeSkin dialysis tubing (3.5-kDa molecular weight cutoff (MWCO), 35-mm diameter, Thermo Scientific) containing 1 ml of 1× TBE supplemented with BSA (0.3 mg ml^−1^ final). The slice was electrophoresed at 5 V cm^−1^ for 1.5 h. The slice was discarded and purified DNA was dialyzed overnight at 4 °C in 1 L of 10 mM Tris pH 8.0. Dialyzed DNA was concentrated using Amicon centrifugal concentrators (100-kDa MWCO, 0.5 ml). DNA concentration was estimated by resolving purified SSB-containing plasmids alongside a DNA standard purified by extraction with phenol, chloroform and isoamyl alcohol (25:24:1) followed by ethanol precipitation (70% (v/v) final) in the presence of sodium acetate (270 mM final) and glycogen (1% final). SSB-containing plasmids were adjusted to 225 ng μl^−1^ and stored at −20 °C.

To generate 1× or 5× SSBs in DNA using nCas9 (H840A), pCRISPR^1X^ (pJD161) or pCRISPR was incubated during licensing with assembled CRISPR–Cas9 RNP, as described below.

### Assembly of CRISPR–Cas9 ribonucleoprotein (RNP) complex

The CRISPR–Cas9 RNP complex was assembled by incubation of guide RNA (gRNA) with either Alt-R S.p. dCas9 protein V3 (Integrated DNA Technologies (IDT)) or Alt-R S.p. Cas9 H840A nickase V3 (IDT). gRNA was prepared by mixing Alt-R CRISPR–Cas9 *trans*-activating CRISPR RNA (tracrRNA) and ATTO550 (IDT) with a tenfold molar excess of Alt-R CRISPR–Cas9 crRNA in nuclease-free duplex buffer (IDT). crRNAs scRNA1 (5′-GGUUGAGUGUUGUUCCAGUU-3′) and scRNA2 (5′-AACAACACUCAACCCUAUCU-3′) targeted leading and lagging strands, respectively. crRNA–tracrRNA mixtures were heated to 95 °C for 5 min and then cooled to room temperature for 1 h. gRNA was stored at −20 °C. Immediately before experiments, RNP was formed by incubating 25 μM Cas9 with 2 μM gRNA in 1× Cas9 dilution buffer (15 mM KCl and 3 mM HEPES, pH 7.5) in 2 μl for ≥ 20 min.

### DNA replication in *Xenopus* extracts

Control plasmid (pJD1) was fully replicated in extract before fork collapse. Activated high-speed supernatant (HSS) was prepared by incubating HSS with an ATP-regenerating system (ARS; 20 mM phosphocreatine, 2 mM ATP and 5 ng μl^−1^ creatine phosphokinase) and nocodazole (3 ng μl^−1^) for 5 min at room temperature. pJD161 (0.1 volumes, 100 ng μl^−1^) was licensed in 0.9 volumes of activated HSS for 20 min at room temperature. Activated nucleoplasmic extract (NPE) was prepared by supplementing NPE with ARS, dithiothreitol (DTT; 2 mM final) and [α-^32^P]dATP (350 nM final). The NPE mix was diluted with 1× egg lysis buffer (ELB; 250 mM sucrose, 2.5 mM MgCl_2_ 50 mM KCl and 10 mM HEPES, pH 7.7) to 45% NPE (v/v). Replication was initiated by adding 0.1 volume of licensing mix to 0.9 volumes of 45% NPE mix. After 40 min, 0.05 volumes of the reaction was added to 0.95 volumes of fresh, activated NPE mix (65% in 1× ELB). This control NPE mix was used in collapse experiments. Rereplication of pJD1 did not occur because NPE contains a high concentration of factors that inhibit origin licensing^61^.

Fork collapse was induced by replicating SSB-containing plasmids in *Xenopus* egg extract. For experiments that used pSSB^LEAD^, pSSB^LAG^, pSSB^LEAD 686 bp^ or pSSB^LEAD-Large^ as template, 0.29 volumes of pSSB (225 ng μl^−1^) was incubated with 0.36 volumes of either TetR (100 μM) or TetR buffer and 0.36 volumes of either LacR (32 μM) or LacR buffer for 1 h at room temperature. These steps were performed immediately before licensing in activated HSS. Repressor-bound plasmid (0.2 volumes) was licensed in 0.8 volumes activated HSS for a final DNA concentration of ~13 ng μl^−1^ and then incubated for 20 min at room temperature. Replication of pSSB was initiated by addition of one volume of licensing mix to two volumes of control NPE mix. For TetR-stabilized reactions, 0.05 volumes of TetR (100 μM) was added to 0.95 volumes of control NPE mix before replication. Where indicated, BRC peptide^92^ (18 μM final) or Cdc7 inhibitor PHA-767491 (300 μM; Sigma-Aldrich) was added. Where indicated, aphidicolin (300 μM; Sigma-Aldrich) was added 22.5 min into reactions and tetracycline (100 μM final; Sigma-Aldrich) was added at 0 and 60 min.

When pCRISPR or pJD161 was used, 0.57 volumes of plasmid (115 ng μl^−1^) was bound with 0.43 volumes of either LacR (32 μM) or LacR buffer for 1 h at room temperature. Assembled RNP was diluted 1.75-fold in H_2_O and 0.57 volumes of RNP was incubated with 0.43 volumes of LacR (32 μM) or LacR buffer for 1 h at room temperature. Repressor-bound plasmid (0.1 volumes) was licensed with 0.8 volumes of activated HSS for 20 min at room temperature. After 20 min, 0.1 volumes of RNP was added and licensing continued for another 10 min (~30 min in total). Replication was initiated by adding one of volume licensing mix to two of volumes control NPE mix.

At indicated time points, reactions were sampled into either 20 volumes of replication stop solution (8 mM EDTA, 0.13% phosphoric acid, 10% Ficoll, 5% SDS, 0.2% bromophenol blue and 80 mM Tris pH 8.0), which stops replication reactions and serves as a DNA loading dye, or extraction stop solution (1% SDS, 25 mM EDTA and 50 mM Tris-HCl pH 7.5), which also stops reactions but is compatible with downstream processing (that is, DNA purification). Stopped reactions were treated with RNase A (182 ng μl^−1^ final) and then proteinase K (1.6 mg ml^−1^ final). Replication stop samples were analyzed by agarose gel electrophoresis at 5 V cm^−1^. Extraction stop samples were purified by either column (Monarch PCR and DNA cleanup kit, NEB) to remove HMw DNA species (as described previously^93^) or extraction with phenol and chloroform followed by ethanol precipitation (70% (v/v) final) in the presence of sodium acetate (270 mM final) and glycogen (1% final) to purify total DNA. Purified DNA samples were resuspended in 6 μl of 10 mM Tris-HCl (pH 8.0).

Experiments conducted in Figs. 1–4, 6 and 7 and Extended Data Figs. 1–7, 9 and 10 used either pSSB^LEAD^ or pSSB^LAG^ as the template for replication. Experiments shown in Fig. 3i–k additionally used pSSB^LEAD-Large^ as a template while the experiments shown in Extended Data Fig. 2i–r used pSSB^LEAD 686 bp^ as a template. Experiments conducted in Fig. 5 and Extended Data Fig. 8 used pCRISPR or pJD161, alternatively called pCRISPR^1X^, as a template. Control plasmid was included as a loading control in all experiments with the following exceptions, for which it was omitted: Fig. 3e–h and Extended Data Figs. 3q, 5a–e, 6g–k and 7a–f.

### Antibodies and peptides

Antibodies targeting *Xenopus* MRE11 and RAD51 were raised by New England Peptide by immunizing rabbits with Ac-CDPFKKSGPSRRGRR-OH for MRE11 and polypeptide PP1 (Supplementary Table 1) for RAD51. For targeting *Xenopus* CtIP, commercial anti-CtIP antibody MAEB1072 (Sigma-Aldrich) was used. The BRC4 peptide comprises residues 1517–1551 of BRCA2 (ref. ^68^). Bacterial cultures carrying pGEX-4T-3 (WT or mutant*) were grown at 37 °C to an optical density at 600 nm of 0.5 and then protein expression was induced with IPTG for 3 h at 37 °C. Following cell lysis and clarification, the GST-tagged peptide was purified using glutathione affinity chromatography and recovered by batch elution. Lastly, the eluate was concentrated and subjected to buffer exchange using a centrifugal filter unit.

### Western blotting and immunodepletion

Protein-A-coupled magnetic beads (Dynabeads protein A, 30 μg μl^−1^) were bound with 0.5 μg of control IgGs or anti-MRE11, anti-RAD51 or anti-CtIP antibody per 1 μl of magnetic beads. For each immunodepletion round, 1.29 volumes of antibody-bound beads were incubated with two volumes of HSS or one volume of NPE for 20 min at room temperature with end-over-end rotation. This was repeated twice for HSS and NPE, for a total of three rounds, as described previously^94^. Depleted extracts were isolated and used for either DNA replication or western blotting. For western analysis, the secondary antibody was horseradish peroxidase (HRP)-conjugated goat anti-rabbit antibody (Jackson Immunoresearch, 111-035-003) for MRE11 and RAD51 and goat anti-mouse for CtIP (Jackson Immunoresearch, 315-035-00). The following antibody dilutions were used for western blotting: MRE11, RAD51 and CtIP (1:5000) and HRP goat anti-rabbit or mouse (1:30,000). Images were acquired using Amersham Imager 600 (GE Healthcare).

### Analysis of replication and repair intermediates

To monitor replication, samples that had been collected into replication stop were separated on a 1% agarose gel at 5 V cm^−1^. Radiolabeled DNA was detected by phosphorimaging, which measured the incorporation of radiolabeled nucleotides, and DNA signal was measured using ImageJ. To measure DNA synthesis, individual whole-lane signals were normalized to the loading control in each lane. Normalized signals were expressed relative to the maximum whole-lane signal across all time points and conditions. Because of nicking of the internal loading control by nCas9 (H840A), DNA synthesis was not normalized to the loading control for CRISPR–Cas9 experiments (Fig. 5 and Extended Data Fig. 8). DNA synthesis was also not normalized to loading control for the MRE11 depletion experiment in Fig. 3 because of omission of the loading control to more clearly visualize CMs. Additionally, DNA synthesis was not normalized to the control for depletion experiments in Fig. 6 because of the overlap of the control with linear molecules.

The abundance of individual DNA species was expressed as a percentage of whole-lane signal (%). Collapse efficiency in single-fork conditions was calculated as 1 − (θ% in collapse conditions/θ% in control conditions) × 100 for each time point. The principle behind this formula is that replication fork structures are reduced in a manner that is directly proportional to the frequency of collapse because of conversion of θ to σ. For converging fork experiments in which plasmids pSSB^LEAD^, pSSB^LAG^ or pCRISPR were used as a template, collapse efficiency was calculated as (((((nCM% + scCM%)/Lin%) − 1)/2) + 1)^−1^ × 100%. The principle behind this formula is that, for converging forks, each convergent collapse event should generate an nCM or scCM and a linear molecule while completion of DNA synthesis without collapse should generate two nCMs or scCMs. For converging fork experiments in which plasmid pJD161 (that is, pCRISPR^1X^) was used as a template, the aforementioned formula was not used to calculate collapse efficiency, as the linear band generated from collapse migrated at the same position as the supercoiled control plasmid. Collapse efficiency was instead calculated using a less direct approach. First, CMs were normalized to the control using CMnorm = (nCM% + scCM% in collapse conditions)/(maximum nCM% + scCM% in control conditions). Second, CMnorm was used to calculate collapse efficiency across all time points by applying the following formula: [1 − ((CMnorm − 0.5)/0.5))] × 100%. The principle behind this formula is that each collapse event should lead to loss of one CM of the two that would be formed if replication proceeded without collapse.

### Analysis of DNA synthesis in the collapse region

To monitor synthesis in the collapse region, purified DNA samples were digested with 0.4 U per μl XmnI and 0.4 U per μl SacI in 1× rCutSmartBuffer (NEB) for 1 h at 37 °C. Digested products were then separated under either native or denaturing agarose gel conditions. For native separation, 6× native loading buffer (Ficoll, EDTA, SDS, bromophenol blue and Tris-HCl (1 M, pH 8)) was added to samples at 1× concentration before separation on a 1% agarose gel at 5 V cm^−1^. For separation under denaturing conditions, digests were stopped by addition of EDTA to a final concentration of 30 mM before the addition of 6× alkaline loading buffer (Ficoll, EDTA, xylene cyanol, bromocresol green and NaOH (10 N)) to a final concentration of 1×. Digests were then separated on a 1.5% alkaline gel at 1.5 V cm^−1^. The gel was then neutralized with gentle agitation in 7% trichloroacetic acid. Dried gels were imaged on the Amersham Typhoon Scanner (GE Healthcare).

Synthesis in the collapse region was calculated using the formula (((*a*/*b*) in each sample condition/(average (*a*/*b*) in control—Tet buffer or vehicle conditions)) × 100%, where *a* is the signal of the collapsed fragment and b is the signal of pJD1 control fragment 1. For single-fork collapse experiments that used pSSB^LEAD^, pSSB^LAG^ or pSSB^LEAD 686 bp^ as a template, ‘expected no repair’ was calculated as (((747/2,212) × fraction of molecules that collapsed) + fraction of molecules that did not collapse) × DNA synthesis percentage within the collapse region in control conditions. In the above formula, the value (747/2,212) corresponds to the amount of nascent synthesis within the collapsed region, assuming no repair. To adjust the expected no repair for differences in replication efficiency, the calculated value was then multiplied by DNA synthesis at *t* = 120 in collapsed conditions/DNA synthesis at *t* = 120 in control conditions). For nCas9-induced single-fork collapse experiments that used template plasmids pCRISPR or pJD161, alternatively termed pCRISPR^1X^, the same calculations as above were used except that 724/2,336 and 625/1,944 were used, respectively, as the expected nascent synthesis within the collapsed region, assuming no repair of collapsed ends. For collapse under converging fork conditions, the same calculations were applied, except that 0.5 was used as the expected signal assuming no repair because the parental strand lacking a SSB was fully replicated. The expected no repair + conv. values given in Extended Data Fig. 2q,r were calculated by adding an additional 12.7% (for pSSB^LEAD 386 bp^) and 20.5% (for pSSB^LEAD 686 bp^) repair signal to expected no repair values. These percentages represent the signal beyond the expected no repair, corresponding to the repair signal that would be expected from forks converging on the SSB as the SSB is moved further away from the *lacO* array. For Extended Data Fig. 1u, the maximum undetectable restart was calculated by expressing the upper bound of the 95% confidence interval of the unpaired difference (TetR − expected no repair) as a percentage of the remaining synthesis capacity (100% − ENR mean). Supplementary Table 2 shows a worked example of how DNA synthesis in the collapse region was calculated.

### Enzymatic analysis of replication and repair intermediates

Where indicated, purified DNA was subjected to the following enzymatic digestions: digestion with 0.8 U per μl AlwNI, XmnI or SapI in 1× rCutSmartBuffer (NEB) for 1 h at 37 °C, double digestion with 0.4 U per μl SacI and 0.4 U per μl KpnI in 1× rCutSmartBuffer (NEB) for 1 h at 37 °C, single digestion with 0.12 U per μl hTOP2α in 1× topoisomerase II assay buffer (Topogen) for 15 min at 37 °C or single digestion with 0.5 nM to 1.5 nM RuvC in 1× NEBuffer 2.1(NEB) supplemented with DTT (1 mM final) and Tris pH 8.0 (40 mM final) for 1 h at 37 °C. hTOP2α digestion was stopped by the addition of 0.5 volumes of TopSTOP solution (3% SDS and 2 mg ml^−1^ proteinase K). RuvC digestion was stopped by the addition of 0.2 volumes of RuvCSTOP solution (3% SDS, 240 mM EDTA and 3 mg ml^−1^ proteinase K). Radiolabeled digestion products were separated on a 1% agarose gel at 5 V cm^−1^ and detected by phosphorimaging.

### Amplicon sequencing of collapsed products

Samples from Fig. 7 experiments were quenched at 120 min, purified as above and processed for amplicon sequencing. Purified DNA (~2.8 ng or ~10 ng) was amplified in a 20-μl reaction using the NEB Phusion high-fidelity PCR kit (final: 1× Phusion HF buffer, 200 μM dNTPs, 500 μM each of SCP1 (5′-GACGTTGGAGTCCACGTTCTTTAATAGTG-3′) and SCP2 (5′-AATGGCGAATGGAAATTGTAAGCGTT-3′) primers and 0.4 U of Phusion DNA polymerase). Amplification followed a touchdown PCR program using the following settings: denaturation at 98 °C for 30 s, followed by ten cycles of denaturation at 98 °C for 10 s, annealing at 72 °C for 15 s (decreasing by 0.5 °C per cycle) and extension at 72 °C for 10 s. Subsequently, seven cycles were performed with denaturation at 98 °C for 10 s, annealing at 67 °C for 15 s and extension at 72 °C for 10 s, followed by a final extension at 72 °C for 7 min. PCR products were purified using the NEB Monarch PCR and DNA cleanup kit according to the manufacturer’s instructions, quantified on a 1% agarose 1× TBE gel stained with SYBR gold and sequenced by Genewiz Amplicon-EZ next-generation sequencing. Furthermore, unreplicated and unnicked pSSB^LEAD^ plasmid (~10 ng) was amplified and sequenced under identical conditions. This was performed to establish a ‘mutation event’ baseline. Sequencing results from this plasmid were subtracted from the experimental sequencing data (that is, background subtraction of pSSB^LEAD^ was performed).

### Sequencing analysis of collapsed products

To analyze sequencing reads, agentic coding (Claude Opus 4.6, Anthropic) was used to generate and implement a custom analysis pipeline. The pipeline performs paired-end read merging, semiglobal alignment to the 276-bp reference template, mutation calling (insertions, deletions and substitutions), classification of mutations relative to the *tetO* repeat array and detection of palindromic reads containing inversion junctions. Reads were assigned to 20 prespecified mutation bins. Condition means were compared using Welch’s *t*-test with Benjamini–Hochberg correction applied independently across all 20 bins for each comparison. The complete pipeline source code, configuration parameters, reference sequences and intermediate outputs are provided on Zenodo (10.5281/zenodo.19687676)^95^.

Pipeline validation used two independent strategies. First, we performed orthogonal reimplementation; all merged reads were realigned using EMBOSS needle^96^ and independently reclassified using minimal regex-based descriptors derived without access to the original pipeline code, following N-version programming principles^97–100^. Second, we performed round-trip verification; classification labels were stripped from pipeline outputs and an independent analysis recovered the classification scheme from the binned reads alone, analogous to round-trip correctness testing for code generated using a large language model^101,102^. Minimal classifiers (regex patterns and length constraints) were derived for each mutation category from the recovered bin characterizations and applied as a cross-check^103^. Validation inputs and outputs are provided on Zenodo (10.5281/zenodo.19687676)^95^.

### End labeling of DNA

Digested DNA products were run alongside a radiolabeled DNA ladder. Radiolabeled ladder was generated by treating a 2-μg DNA ladder (NEB) with 10 U of T4 PNK (NEB) in 1× PNK reaction buffer (NEB) supplemented with 1.33 mM [γ-^32^P]ATP in a 20-μl volume. The reaction proceeded for 45 min at 37 °C and then purified using the Monarch PCR and DNA cleanup kit (NEB) according to the manufacturer’s instructions.

### Materials availability

Plasmids and antibodies generated in this study are available from the corresponding author upon request.

### Reporting summary

Further information on research design is available in the Nature Portfolio Reporting Summary linked to this article.

## Online content

Any methods, additional references, Nature Portfolio reporting summaries, source data, extended data, supplementary information, acknowledgements, peer review information; details of author contributions and competing interests; and statements of data and code availability are available at 10.1038/s41594-026-01812-9.

## Supplementary information

Supplementary Information Supplementary Tables 1 and 2.

Reporting Summary

Peer Review File

## Source data

Source Data Fig. 1 Statistical source data.

Source Data Fig. 1 Unprocessed gels and western blots.

Source Data Fig. 2 Statistical source data.

Source Data Fig. 2 Unprocessed gels and western blots.

Source Data Fig. 3 Statistical source data.

Source Data Fig. 3 Unprocessed gels and western blots.

Source Data Fig. 4 Statistical source data.

Source Data Fig. 4 Unprocessed gels and western blots.

Source Data Fig. 5 Statistical source data.

Source Data Fig. 5 Unprocessed gels and western blots.

Source Data Fig. 6 Statistical source data.

Source Data Fig. 6 Unprocessed gels and western blots.

Source Data Fig. 7 Statistical source data.

Source Data Extended Data Fig. 1 Statistical source data.

Source Data Extended Data Fig. 1 Unprocessed gels and western blots.

Source Data Extended Data Fig. 2 Statistical source data.

Source Data Extended Data Fig. 2 Unprocessed gels and western blots.

Source Data Extended Data Fig. 3 Statistical source data.

Source Data Extended Data Fig. 3 Unprocessed gels and western blots.

Source Data Extended Data Fig. 4 Statistical source data.

Source Data Extended Data Fig. 5 Statistical source data.

Source Data Extended Data Fig. 5 Unprocessed gels and western blots.

Source Data Extended Data Fig. 6 Statistical source data.

Source Data Extended Data Fig. 6 Unprocessed gels and western blots.

Source Data Extended Data Fig. 7 Statistical source data.

Source Data Extended Data Fig. 7 Unprocessed gels and western blots.

Source Data Extended Data Fig. 8 Statistical source data.

Source Data Extended Data Fig. 8 Unprocessed gels and western blots.

Source Data Extended Data Fig. 9 Statistical source data.

Source Data Extended Data Fig. 9 Unprocessed gels and western blots.

Source Data Extended Data Fig. 10 Statistical source data.

## Data Availability

Raw sequencing data generated in this study were deposited to the National Center for Biotechnology Information Sequence Read Archive under BioProject PRJNA1454974. Processed data, intermediate analysis files and all other datasets supporting the findings of this study were deposited to Zenodo (10.5281/zenodo.19687676)^95^. Any additional information is available from the corresponding author on request. Source data are provided with this paper.

## Code availability

All custom code used for sequencing analysis (paired-end read merging, semiglobal alignment, mutation calling and classification and palindromic read detection) and for pipeline validation (orthogonal reimplementation using EMBOSS needle, round-trip verification and minimal regex-based classifiers) was deposited to Zenodo alongside the associated data (10.5281/zenodo.19687676)^95^ under an open-source license approved by the Open Source Initiative.
