# Supplementary material for: Distinct repair outcomes from single and convergent replication fork collapse
Source: Nat Struct Mol Biol. 2026 May 27;33(6):939–52. doi: 10.1038/s41594-026-01812-9 (PMC13275508; doi:10.1038/s41594-026-01812-9)
Supplement: Supplementary file 1 — Supplementary Tables 1 and 2. [file 41594_2026_1812_MOESM1_ESM.pdf]

nature structural & molecular biology

Supplementary information https://doi.org/10.1038/s41594-026-01812-9

Distinct repair outcomes from single and convergent replication fork collapse

In the format provided by the authors and unedited

Table of Contents

Supplementary Table 1. 2
Supplementary Table 2 4

Supplementary Table 1. Table of DNA and Protein Sequences

ID	Sequence (5’-3’)
JDD27	5’-TCTCTATCACTGATAGGGAATGCTCTCTATCACTGATAGGGAATGCTCTCTA<br>TCACTGATAGGGAATGCTCTCTATCACTGATAGGGAATGCTCTCTATCACTGAT<br>AGGGAATGCTCTCTATCACTGATAGGGA-3’
IS1	5’-CGCCCCGACAACTGCAAACCCCAACTTATTTAGATAACATGGTTAGCCGAA<br>GTTGCAGGGGGTGCCGACCGTGGAGTCCTCCCCTGGTGTGGCTCCTTCATC<br>TGACAACATGCAACCGCTACCACCATCGATTGATTCAGCGGACGGTGTTGTT<br>GTCATAGATTCGGCACATTTCCCTTGTAGGTGTGAAATCACTTAGCTTCGCGC<br>CGTAGTCTTATGGCAAAACCGATGGACTATGTTTCGGGTAGCACCAGGAGTCT<br>GTAGCACGTCCATCTCAACGTGGCGTGCGTACACCTTAATCACCGCTTCATC<br>CTAAGGATCTGGCTCCATGCTATGTTGATACGCCTAGACTGCTCGAAGAAAAT<br>ATACGAAGCGGGCGGCCTGGCCGGAGCGCTACCGCATCGACCCGTATTCGT<br>TTACTGTTAATTGCTGACACATGAGCAATATTGTAGACCGTCAATTTCAGCCCT<br>CTTATCCTCGGTGTTGTGTGTCAAATGGCGTAGATCTGGATTGACTCTATGAC<br>GGTATCTGCTGATCGGTAGGGACACCGAGAATCTATCGGGCTATGTCACTAAA<br>ACTTTCCAAACAGCCCGTGTCGATACTGAACGAATCGATGCACACTCCCTTCC<br>TTGAAAACGCACAATCATACAAGTGGGCACATGATGGGTACGCCCATCTAATA<br>CATCCAACTCTCTACGCCCTCTTCAAGAGCTGGAAGGGCACCCTGCACTTGG<br>ATAAGTGATAACCTCGTAAGGCAAGCTCGTACCGTCATTCATGCGGAAGAGTT<br>AAGACCATTGGAAGTAGGGATAGTTTCGAACCTCGGTTACTAGTCCCAATAAG<br>GGAACCCTGTCTGAAGGATGAGTGTCAGCCAGTGTAACCCGATGACGTACCC<br>AGAAGTCGAACTGGGCCAGACAACCCGGCGCTAACGCACTCAAACCCGTGG<br>CCCGACGCGACATATCAGCTAAGAGTAGGCCGGGAGTGTAGACCTTTGGGG<br>TTGAATAAATCTATTGTACTAATCGGCTTCAACGAGCCGTACAGGTGGCACCT<br>CAGGAGGGGCCCGCAGGGAGGAAGTAAACTGCTATTCGTCGCCGTTGGTGG<br>TAACTAATTGTGTTCCTTGCCACTACAATTGTATCTAAGCCGTGTAATGAGAAC<br>AACCACACCATAGCGAATTGATGCGCCGCTTCGGAATACCGTTTTGGCTACCC<br>GTTACTAAGCCGATCGCGATTTTCAGGTATCGTGCACGTTGGGTTGGACCGC<br>ACGCATGTCAAACTGCTGGCGAAGTGCGATTCCACGACCGGTGTACGATTTA<br>ACTACGCCGACGTGACGAGTTTCCTGTTAATGCTTCGCCCGCCGGACCGCC<br>GTCGTGATGGGGTAGCTGCGCATGAGCTTATGACAAATAACGAGAGTGTACTT<br>GTTTAATCATCTCACGGTGAAAGTCGGGAGAACAGCAGCCGCTACACACATT<br>TTACCGCAACTACACCTAGCTGAGATATTTCCATAGATGACTACGCATCCCTCT<br>AGGCCTTACATAACCGGATACAGTGACTTTGACAGGTTTGTGGGGTACAGCA<br>ATGACTTGCATAGCTGCGTATGGAGGAAGGAACTCTTGCGTGTTAGTATGTTG<br>ACCCCTGTATTACGGATGCGGGTAGAAGATGTGGGCAGAGACACCCAGGTCA<br>AGTTCTCGACCTTCTCGTAGGAGGTGTTCCAGTTCACCATACGACCATACCAT<br>TCGAGCATGGCACTATGTACGCTGTCCCCATTCTGGTAGTCATCATCCCTATC<br>ACGGTTTCGAGTGAGTGGTGACGGATATTCCCCACGAATGGACATCTTATTCA<br>CAGTCGGTCACATTGGGGTACTCCTTGGCTTTTCCGCTTGGCCCGGTCTGTT<br>AGGCCCCCGTAGCGTGAGTTTCGGCCCTGTGCTGCCCAGTGTCGGCCATTC<br>TCATTGGGGCCTCACTTCTGGATACCCCGACCTATTTTGACGGCACCATTGG<br>CGGAAGTTGT-3’

IS2	5’-TACGGGCTGCCACCGAGGACTCAACGCGCGTGTCGCTCCTTCATCTGACA ATATGCAGCCGCTACCACCATCGTTTAATACAACGAACGGTGATGTTGTCATA GATTCGGCACATTTCCCTTGTAGGTGTGAAATCACTTAGCTTCGCGCCGAAGT CTTATGGCAAAACCGATGGACTATGTTTCGGGTAGCACCAGAAGTCATTAGCA GATGATCCCAACGTGGCGTGCGTACACCTTAATCACCGCTTCTTGCTAATGTC CTGGCTGTGCTATGTTGATACGCCTGCTCTGCTCTGCA-3’
JDO143	5’-CCAAACTGGAACAACACTCAACCCTATCTCGGGTGACATACGAGTCTTACC AAACTGGAACAACACTCAACCCTATCTCGGCCAGACAGTGGACTCTGCCAAA CTGGAACAACACTCAACCCTATCTCGGAGGCAGAATCGCCCGTACCAAACTG GAACAACACTCAACCCTATCTCGGTTACATTCTCACCGTCGTTA-3’
JDO144	5’-TAACGACGGTGAGAATGTAACCGAGATAGGGTTGAGTGTTGTTCCAGTTTG GTACGGGCGATTCTGCCTCCGAGATAGGGTTGAGTGTTGTTCCAGTTTGGCA GAGTCCACTGTCTGGCCGAGATAGGGTTGAGTGTTGTTCCAGTTTGGTAAGA CTCGTATGTCACCCGAGATAGGGTTGAGTGTTGTTCCAGTTTGG-3’
PP1	TTATEFHQRRSEIIQIGTGSKELDKLLQGGIETGSITEMFGEFRTGKTQLCHTLAV TCQLPIDRGGGEGKAMYIDTEGTFRPERLLAVAERYGLSGSDVLDNVAYARAFN TDHQTQLLYQASAMMAESRYALLIVDSATALYRTDYSGRGELSARQMHLARFLR MLLRLADEFGVAVVITNQVVAQVDGAAMFAADPKKPIGGNIIAHASTTRLYLRKG RGETRICKIYDSPCLPEAEAMFAINADGVGDAKD

Supplementary Table 1. Sequences of DNA inserts, oligonucleotides, and polypeptides used in this study. DNA sequences (5′–3′) of inserts and oligonucleotides were used to construct template plasmids. DNA duplex JDD27 was used to construct plasmids pSSB^{LEAD} and pSSB^{LAG}. Insert IS1 was used to construct pSSB$^{LEAD\text{-}Large}$ while IS2 was used to generate pSSB$^{LEAD\ 686\ bp}$. Oligonucleotides JDO134 and JDO144 were used to generate pCRISPR. Lastly, PP1 is the polypeptide sequence used to immunize rabbits for generation of anti-*Xenopus* RAD51 antibody See Methods for full details.

Supplementary Table 2. Worked Example of Calculating DNA Synthesis and Expected No Repair Synthesis

Condition	Collapsed (AU) "a"	Control (AU) "b"	DNA Synthesis (AU) at T=120	θs (% Whole Lane)
Buffer Ctrl 1	11,000	100	100	35%
Buffer Ctrl 2	9,000	100	100	30%
TetR 1	5,500	100	70	3.5%
TetR 2	4,500	100	80	0%

Note: Collapse (AU) and Control (AU) are measured from digest gels, e.g., Fig 1F or 1M. Whole Lane (AU) and Thetas (%) are measured from replication gels, e.g., Fig. 1B or 1I.

Calculation of DNA Synthesis

Formula: [((a / b) in each sample condition/ (average (a / b) in control i.e. Tet buffer or vehicle conditions)] x 100%; where a= signal of the collapsed fragment and b=signal of pJD1 control fragment 1

Step 1: Normalize collapsed fragment to control fragment, i.e. "(a/b) in the sample condition"

Buffer Ctrl 1: 11,000 / 100 = 110 AU
Buffer Ctrl 2: 9,000 / 100 = 90 AU
TetR 1: 5,500 / 100 = 55 AU
TetR 2: 4,500 / 100 = 45 AU

Step 2: Calculate Average (AU) for control conditions, i.e., "average (a/b) in control conditions"

Average Buffer Ctrl: (110 AU + 90 AU) / 2 = 100 AU

Step 3: Calculate Synthesis (%) within collapse region, i.e., divide Step 1 values by Step 2 value then multiply by 100

Buffer Ctrl 1: (110 AU / 100 AU) x 100 = 110%
Buffer Ctrl 2: (90 AU / 100 AU) x 100 = 90%
TetR 1: (55 AU / 100 AU) x 100 = 55%
TetR 2: (45 AU / 100 AU) x 100 = 45 %

Calculation of Adjusted Expected No Repair

Formula: ([(747/2212) x fraction of molecules that collapsed] + fraction of molecules that did not collapse) x DNA synthesis (%) within the collapse region of control conditions

Step 1: Calculate Collapse Efficiency, i.e., "fraction of molecules that collapsed"

Formula: [1- (θs% in collapse conditions / θs% in control conditions)] x 100
TetR 1: [1-3.5/35)] x 100 = 90% (i.e., 0.9 of molecules collapsed, 0.1 of molecules did not collapse
TetR 2: [1-0/30)] x 100 = 100%

Step 2: Calculate Replication Efficiency

Formula: DNA Syntheses (AU) of Collapsed Conditions/ DNA Synthesis of Control Conditions at T=120
TetR 1: (70 / 100) = 0.7
TetR 2: (80 / 100) = 0.80

Step 3: Calculate Expected No Repair AND adjust by Replication Efficiency

TetR 1: ([(747/2212) x 0.9] + 0.1) x 110% = 40.4% Adjusted: 40.4% x 0.70 = 28.3%
TetR 2: ([(747/2212) x 1]) x 90% = 30.4% Adjusted: 30.4% x 80 = 24.3%

Supplementary Table 2. Worked example of calculating DNA synthesis and expected no repair. Representative values illustrating the quantification of DNA synthesis within the collapse region and the adjusted expected no repair signal. Collapsed (AU) and Control (AU) values are measured from restriction digest gels. Whole lane (AU) and theta (θ) percentages are measured from replication gels. See Methods for full details.
