# Supplementary material for: Distinct repair outcomes from single and convergent replication fork collapse
Source: Nat Struct Mol Biol. 2026 May 27;33(6):939–52. doi: 10.1038/s41594-026-01812-9 (PMC13275508; doi:10.1038/s41594-026-01812-9)
Supplement: Supplementary file 2 — Reporting Summary [file 41594_2026_1812_MOESM2_ESM.pdf]

nature portfolio

Corresponding author(s): James M Dewar

Last updated by author(s): Feb 22, 2026

Reporting Summary

Nature Portfolio wishes to improve the reproducibility of the work that we publish. This form provides structure for consistency and transparency in reporting. For further information on Nature Portfolio policies, see our Editorial Policies and the Editorial Policy Checklist.

Statistics

For all statistical analyses, confirm that the following items are present in the figure legend, table legend, main text, or Methods section.

n/a	Confirmed	
☐	☒	The exact sample size (n) for each experimental group/condition, given as a discrete number and unit of measurement
☐	☒	A statement on whether measurements were taken from distinct samples or whether the same sample was measured repeatedly
☐	☒	The statistical test(s) used AND whether they are one- or two-sided<br>*Only common tests should be described solely by name; describe more complex techniques in the Methods section.*
☒	☐	A description of all covariates tested
☐	☒	A description of any assumptions or corrections, such as tests of normality and adjustment for multiple comparisons
☒	☐	A full description of the statistical parameters including central tendency (e.g. means) or other basic estimates (e.g. regression coefficient) AND variation (e.g. standard deviation) or associated estimates of uncertainty (e.g. confidence intervals)
☐	☒	For null hypothesis testing, the test statistic (e.g. F, t, r) with confidence intervals, effect sizes, degrees of freedom and P value noted<br>*Give P values as exact values whenever suitable.*
☒	☐	For Bayesian analysis, information on the choice of priors and Markov chain Monte Carlo settings
☒	☐	For hierarchical and complex designs, identification of the appropriate level for tests and full reporting of outcomes
☒	☐	Estimates of effect sizes (e.g. Cohen's d, Pearson's r), indicating how they were calculated

Our web collection on statistics for biologists contains articles on many of the points above.

Software and code

Policy information about availability of computer code

Data collection	Autoradiographs - Amersham Typhoon Scanner (GE Healthcare) software version 2.0.0.6; Western blots - Amersham Imager 600 (GE Healthcare) software version 1.2.0
Data analysis	Autoradiographs - ImageJ version 1.53i; Bar graphs and statistical tests - Prism (GraphPad) v10; Line graphs - Microsoft Excel; Sequencing analysis - Custom Python 3 scripts using NumPy, SciPy, pandas, statsmodels, Biopython (v1.83; PairwiseAligner), and matplotlib; orthogonal validation used EMBOSS needle, R/ggplot2, and MUSCLE (v5.3). All custom code used for sequencing analysis and for pipeline validation is deposited alongside the associated data at Zenodo (DOI: 10.5281/zenodo.19687677) under an OSI-approved open-source license.

For manuscripts utilizing custom algorithms or software that are central to the research but not yet described in published literature, software must be made available to editors and reviewers. We strongly encourage code deposition in a community repository (e.g. GitHub). See the Nature Portfolio guidelines for submitting code & software for further information.

Data

Policy information about availability of data

All manuscripts must include a data availability statement. This statement should provide the following information, where applicable:

- Accession codes, unique identifiers, or web links for publicly available datasets
- A description of any restrictions on data availability
- For clinical datasets or third party data, please ensure that the statement adheres to our policy

Raw sequencing data generated in this study have been deposited at the NCBI Sequence Read Archive under BioProject accession PRJNA1454974. Source Data files accompany this article and provide the numerical values underlying all graphed measurements, together with uncropped scans of all blots and gels. Processed data, intermediate analysis files, and all other datasets supporting the findings of this study are deposited at Zenodo103 (DOI: 10.5281/zenodo.19687677). Any additional information is available from the corresponding author on request.

Research involving human participants, their data, or biological material

Policy information about studies with human participants or human data. See also policy information about sex, gender (identity/presentation), and sexual orientation and race, ethnicity and racism.

Reporting on sex and gender	*Use the terms sex (biological attribute) and gender (shaped by social and cultural circumstances) carefully in order to avoid confusing both terms. Indicate if findings apply to only one sex or gender; describe whether sex and gender were considered in study design; whether sex and/or gender was determined based on self-reporting or assigned and methods used. Provide in the source data disaggregated sex and gender data, where this information has been collected, and if consent has been obtained for sharing of individual-level data; provide overall numbers in this Reporting Summary. Please state if this information has not been collected. Report sex- and gender-based analyses where performed, justify reasons for lack of sex- and gender-based analysis.*
Reporting on race, ethnicity, or other socially relevant groupings	*Please specify the socially constructed or socially relevant categorization variable(s) used in your manuscript and explain why they were used. Please note that such variables should not be used as proxies for other socially constructed/relevant variables (for example, race or ethnicity should not be used as a proxy for socioeconomic status). Provide clear definitions of the relevant terms used, how they were provided (by the participants/respondents, the researchers, or third parties), and the method(s) used to classify people into the different categories (e.g. self-report, census or administrative data, social media data, etc.) Please provide details about how you controlled for confounding variables in your analyses.*
Population characteristics	*Describe the covariate-relevant population characteristics of the human research participants (e.g. age, genotypic information, past and current diagnosis and treatment categories). If you filled out the behavioural & social sciences study design questions and have nothing to add here, write "See above."*
Recruitment	*Describe how participants were recruited. Outline any potential self-selection bias or other biases that may be present and how these are likely to impact results.*
Ethics oversight	*Identify the organization(s) that approved the study protocol.*

Note that full information on the approval of the study protocol must also be provided in the manuscript.

Field-specific reporting

Please select the one below that is the best fit for your research. If you are not sure, read the appropriate sections before making your selection.

☒ Life sciences ☐ Behavioural & social sciences ☐ Ecological, evolutionary & environmental sciences

For a reference copy of the document with all sections, see nature.com/documents/nr-reporting-summary-flat.pdf

Life sciences study design

All studies must disclose on these points even when the disclosure is negative.

Sample size	No sample size calculation was performed. Biochemical experiments were performed at least twice with a representative result shown and, where relevant, quantification of multiple repeats. This is consistent with standard biochemical practice.
Data exclusions	No data were excluded from the analysis
Replication	All experiments were replicated at least twice. All results reproduced.
Randomization	Randomization is not relevant to this study. Xenopus egg extracts and cells were split evenly between different experimental conditions to ensure that essentially identical starting material was used in call cases.
Blinding	For biochemical experiments samples could not be blinded because it was necessary to present individual samples in the appropriate order

Blinding	for visual analysis. Blinding also would not matter because these are bulk assays where the entire sample is measured using densitometry which is highly objective.

Reporting for specific materials, systems and methods

We require information from authors about some types of materials, experimental systems and methods used in many studies. Here, indicate whether each material, system or method listed is relevant to your study. If you are not sure if a list item applies to your research, read the appropriate section before selecting a response.

Materials & experimental systems

n/a	Involved in the study
☐	☒ Antibodies
☒	☐ Eukaryotic cell lines
☒	☐ Palaeontology and archaeology
☐	☒ Animals and other organisms
☒	☐ Clinical data
☒	☐ Dual use research of concern
☒	☐ Plants

Methods

n/a	Involved in the study
☒	☐ ChIP-seq
☒	☐ Flow cytometry
☒	☐ MRI-based neuroimaging

Antibodies

Antibodies used	Antibody targeting Xenopus MRE11 was raised by immunizing rabbits with Ac-CDPFKKSGPSRRGRR-OH and is available from New England Peptide (NEP3875). Antibody targeting Xenopus RAD51 was raised by immunizing rabbits with "PP1" (sequence given in Supplemental Table 1) and is available from New England Peptide (NEP5205). Anti-CtIP, clone 11-1 antibody was used for targeting Xenopus CtIP (Supplier Sigma, Cat # MABE1072, Lot # 4115705).
Validation	The specificity of the antibody for the target peptide was confirmed by the manufacturer. The antibody was then tested in Xenopus egg extracts and found to immunoprecipitate the target proteins. For all antibodies used in the study, the efficiency of immunoprecipitation is provided within Extended Data Figures 7 and 9. For immunodepletion: 0.5 mg antibody per 1 mg Protein A Dynabeads, with 1.29 volumes antibody-bound beads per 0.5 volumes HSS or 1 volume NPE, three rounds. For western blotting: 1:5000 primary target antibody and 1:30000 secondary goat-anti rabbit (Jackson Immunoresearch, 111-035-003) or goat-anti mouse (Jackson Immunoresearch, 315-035-00).

Animals and other research organisms

Policy information about studies involving animals; ARRIVE guidelines recommended for reporting animal research, and Sex and Gender in Research

Laboratory animals	Xenopus Laevis, approximately 18 months and older
Wild animals	Study did not involve wild animals
Reporting on sex	N/A. Xenopus egg extracts are used as an experimental system for this study. These extracts are derived from frog eggs, prior to sex determination.
Field-collected samples	The study did not involve animals collected from the field
Ethics oversight	Animal protocols were approved by Vanderbilt Division of Animal Care (DAC) and Institutional Animal Care and Use committee (IACUC).

Note that full information on the approval of the study protocol must also be provided in the manuscript.

Plants

Seed stocks	*Report on the source of all seed stocks or other plant material used. If applicable, state the seed stock centre and catalogue number. If plant specimens were collected from the field, describe the collection location, date and sampling procedures.*
Novel plant genotypes	*Describe the methods by which all novel plant genotypes were produced. This includes those generated by transgenic approaches, gene editing, chemical/radiation-based mutagenesis and hybridization. For transgenic lines, describe the transformation method, the number of independent lines analyzed and the generation upon which experiments were performed. For gene-edited lines, describe the editor used, the endogenous sequence targeted for editing, the targeting guide RNA sequence (if applicable) and how the editor was applied.*
Authentication	*Describe any authentication procedures for each seed stock used or novel genotype generated. Describe any experiments used to assess the effect of a mutation and, where applicable, how potential secondary effects (e.g. second site T-DNA insertions, mosiacism, off-target gene editing) were examined.*
