# Supplementary figures and images for: Distinct repair outcomes from single and convergent replication fork collapse
Source: Nat Struct Mol Biol. 2026 May 27;33(6):939–52. doi: 10.1038/s41594-026-01812-9 (PMC13275508; doi:10.1038/s41594-026-01812-9)

Figure 1B

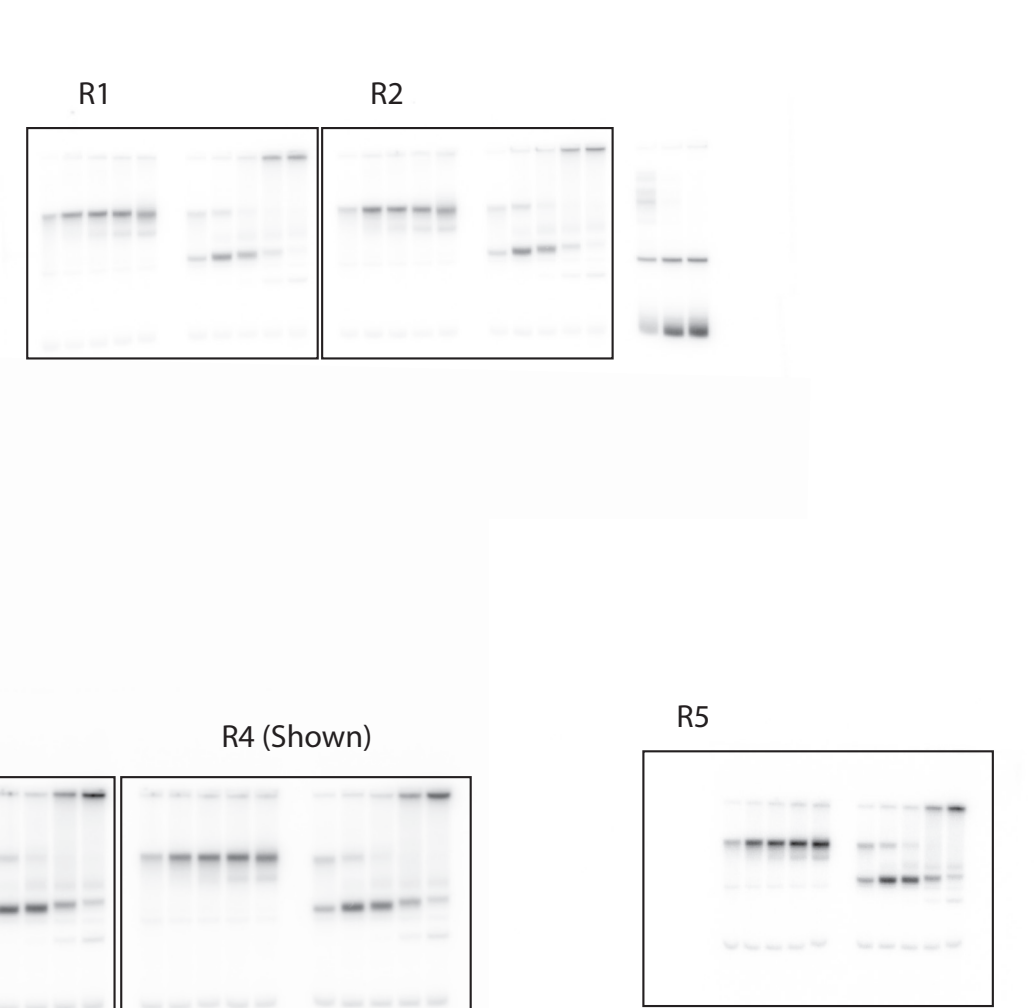

Figure 1B

R6

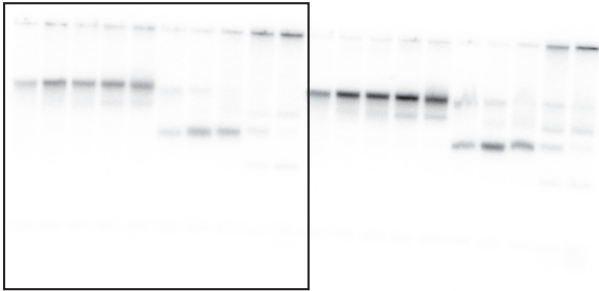

R7

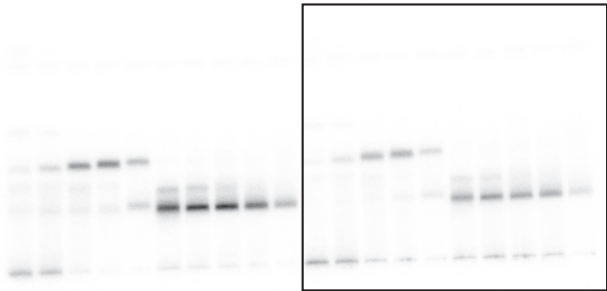

R8

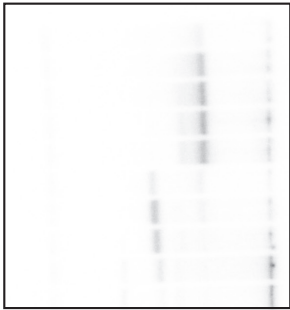

R9

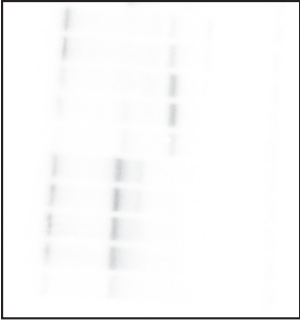

Figure 1F

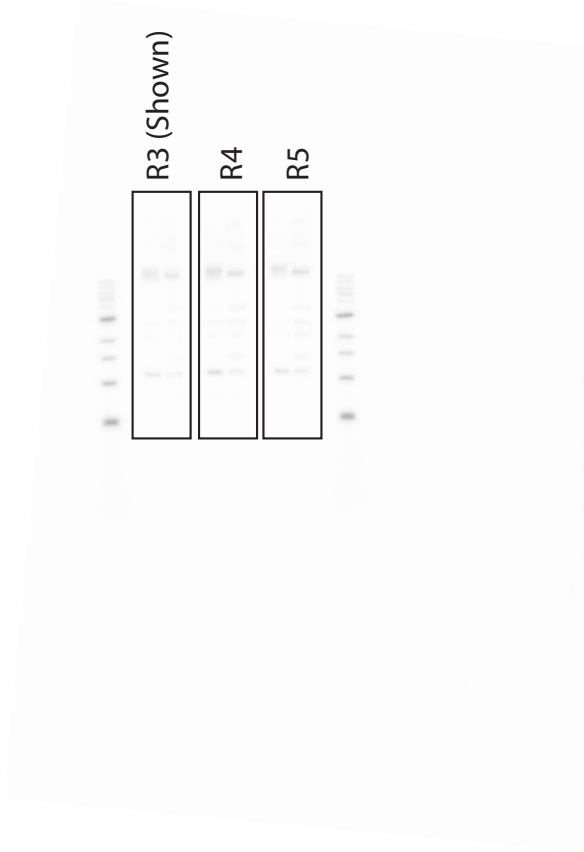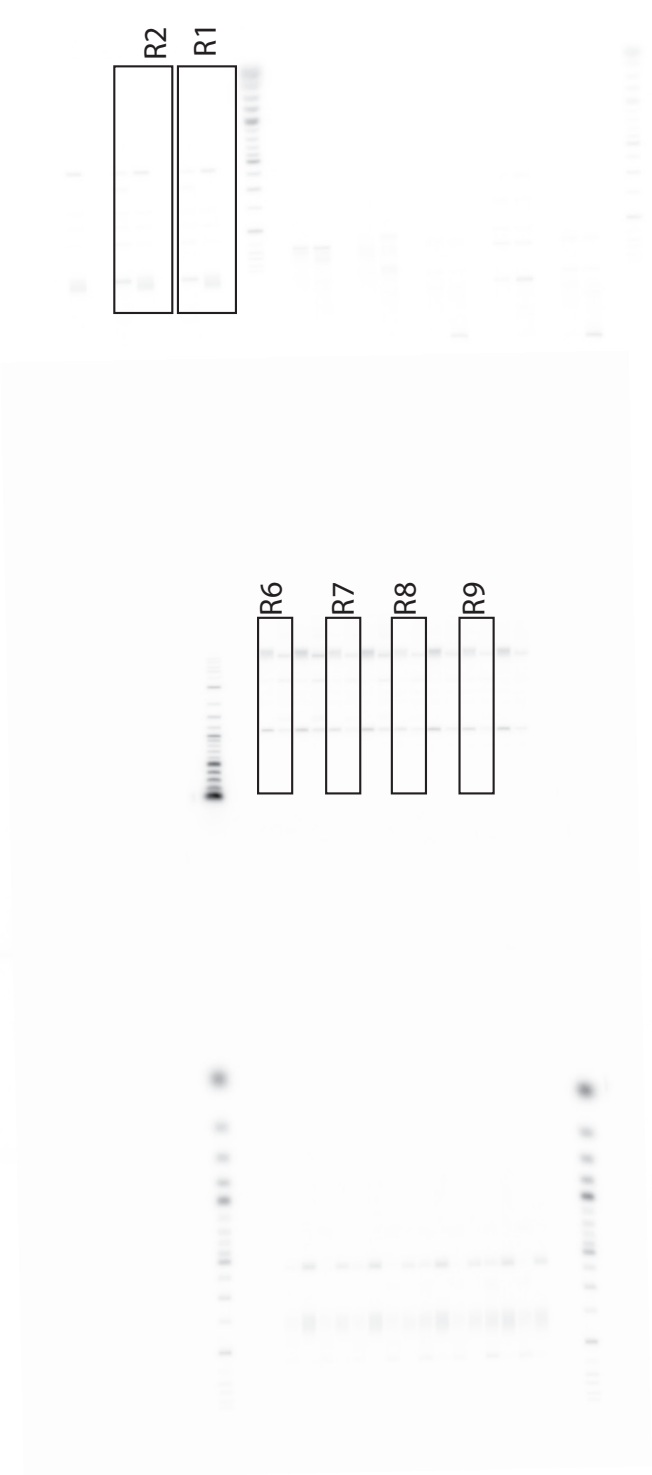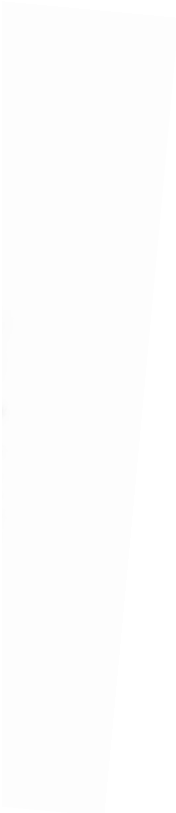

Figure 11

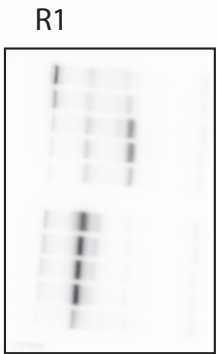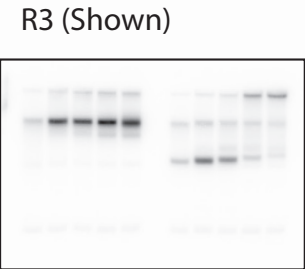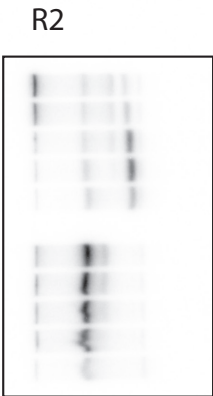

Figure 1M

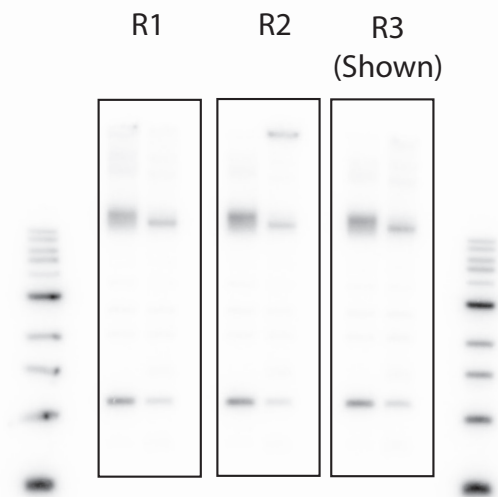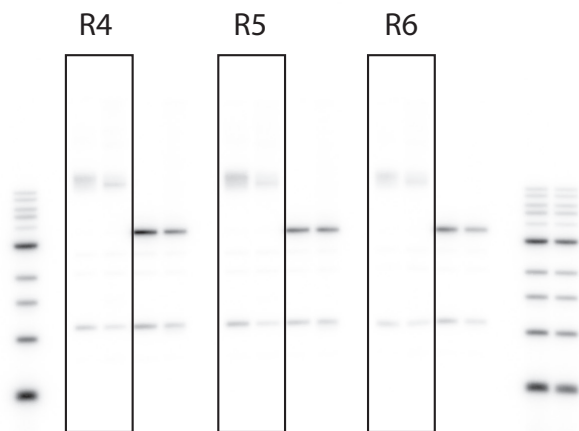

Supplement: Supplementary file 5 — Unprocessed gels and western blots. [file 41594_2026_1812_MOESM5_ESM.pdf]

Figure 2B

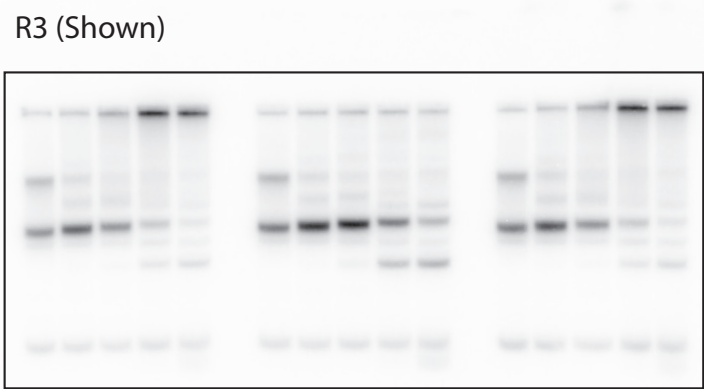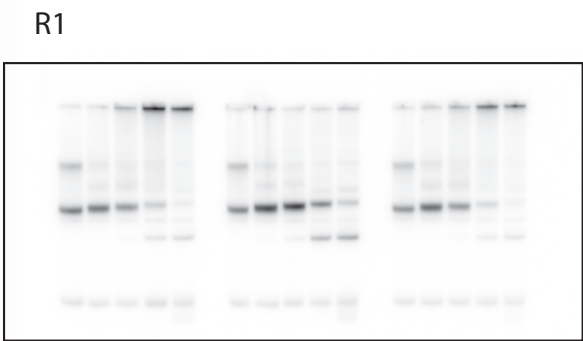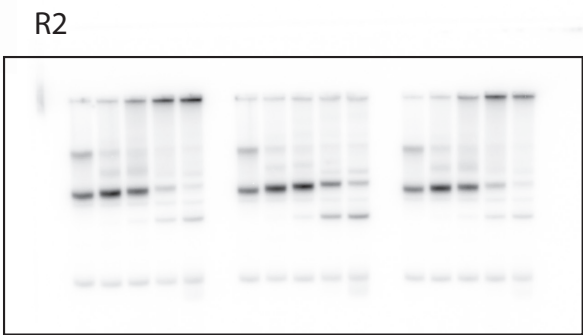

Figure 2D

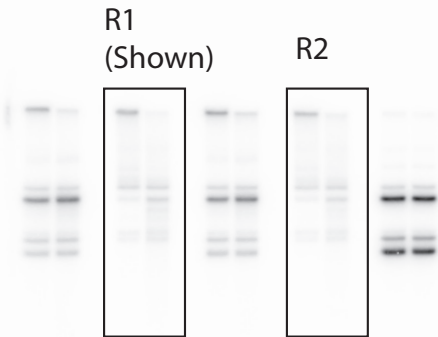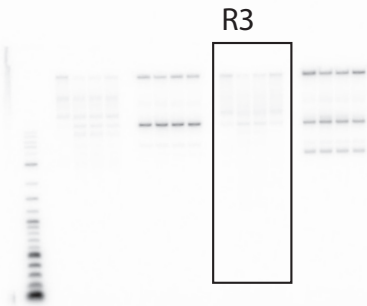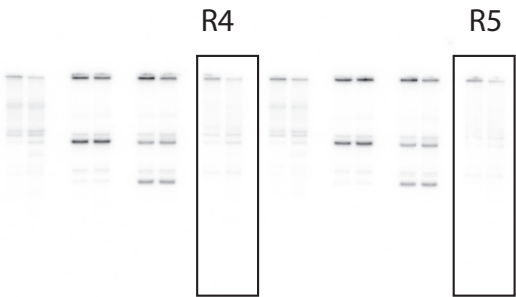

Figure 2F

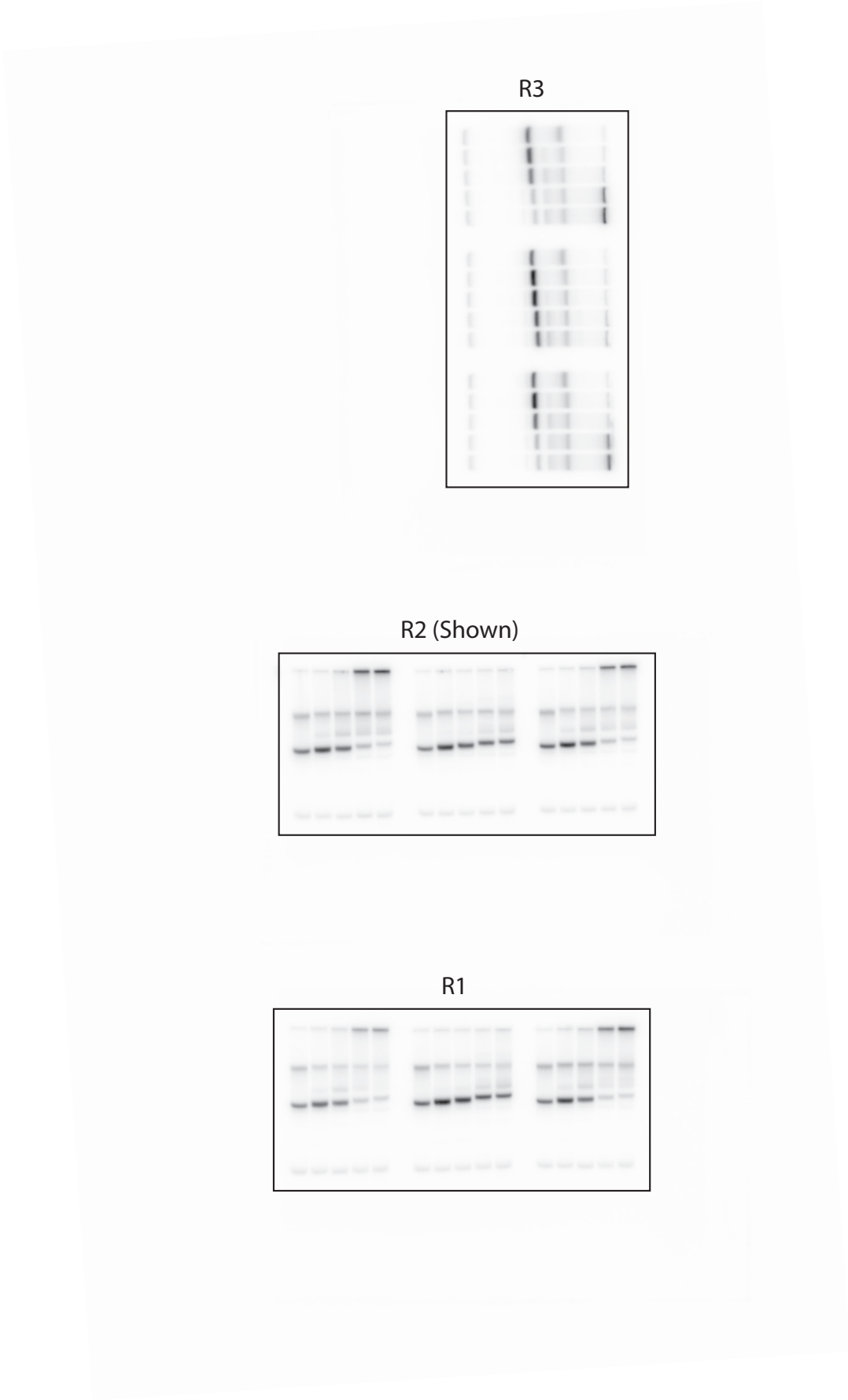

Figure 2H

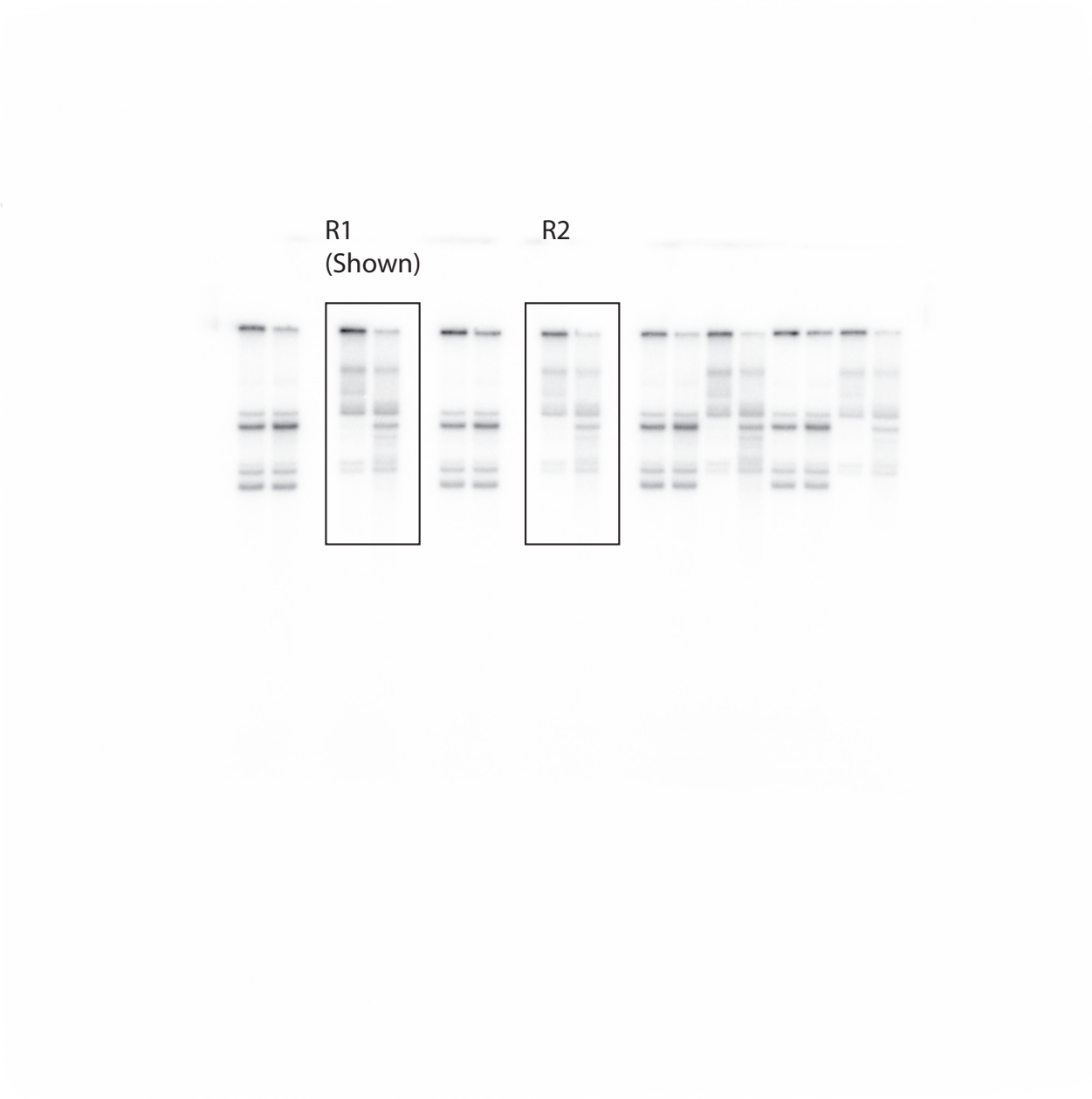

Figure 2J

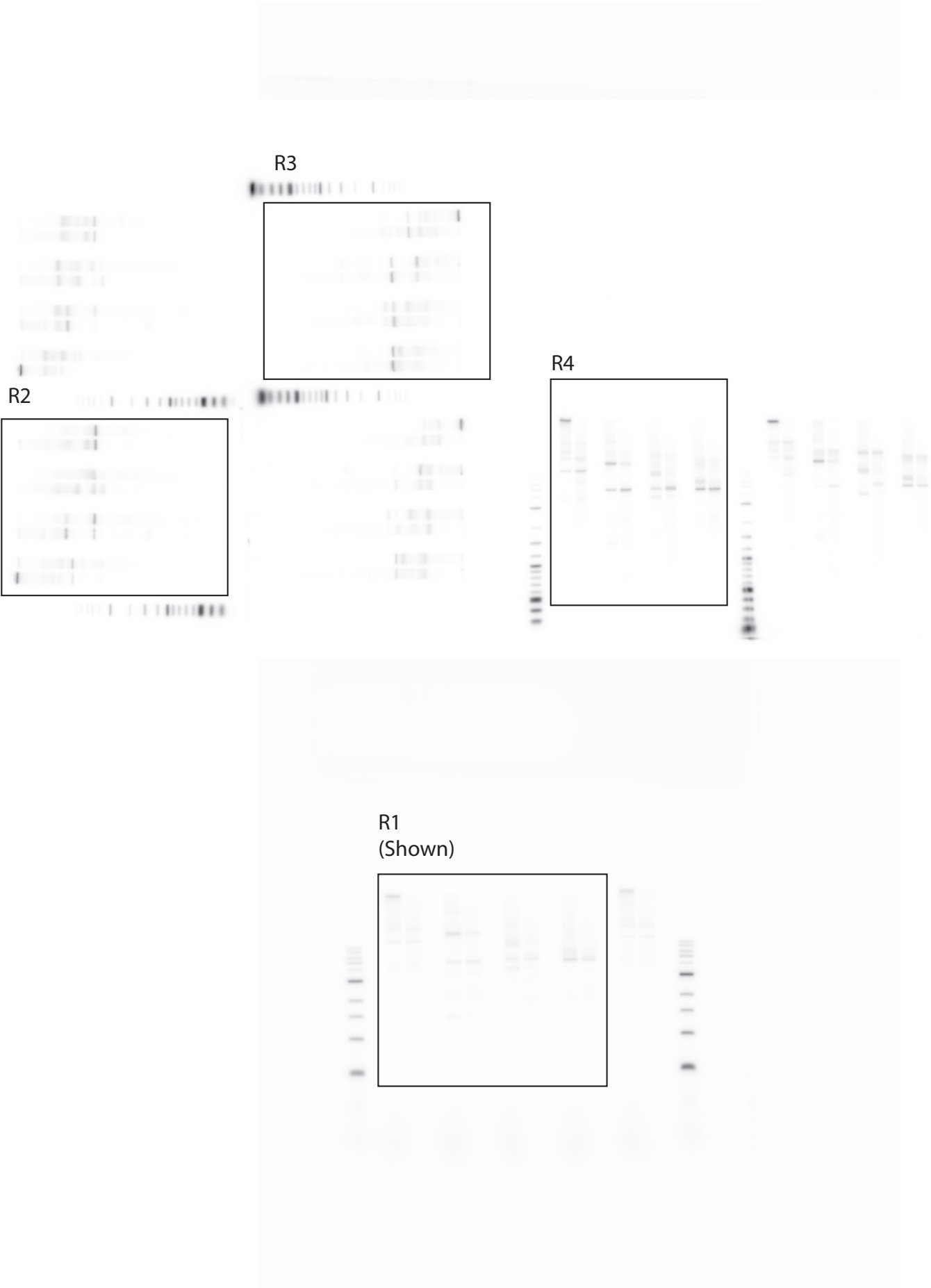

Supplement: Supplementary file 7 — Unprocessed gels and western blots. [file 41594_2026_1812_MOESM7_ESM.pdf]

Figure 3B

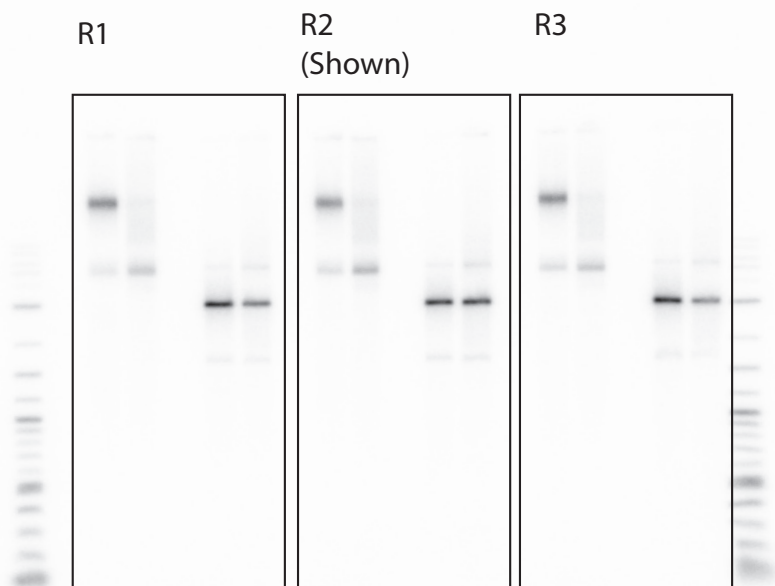

Figure 3F

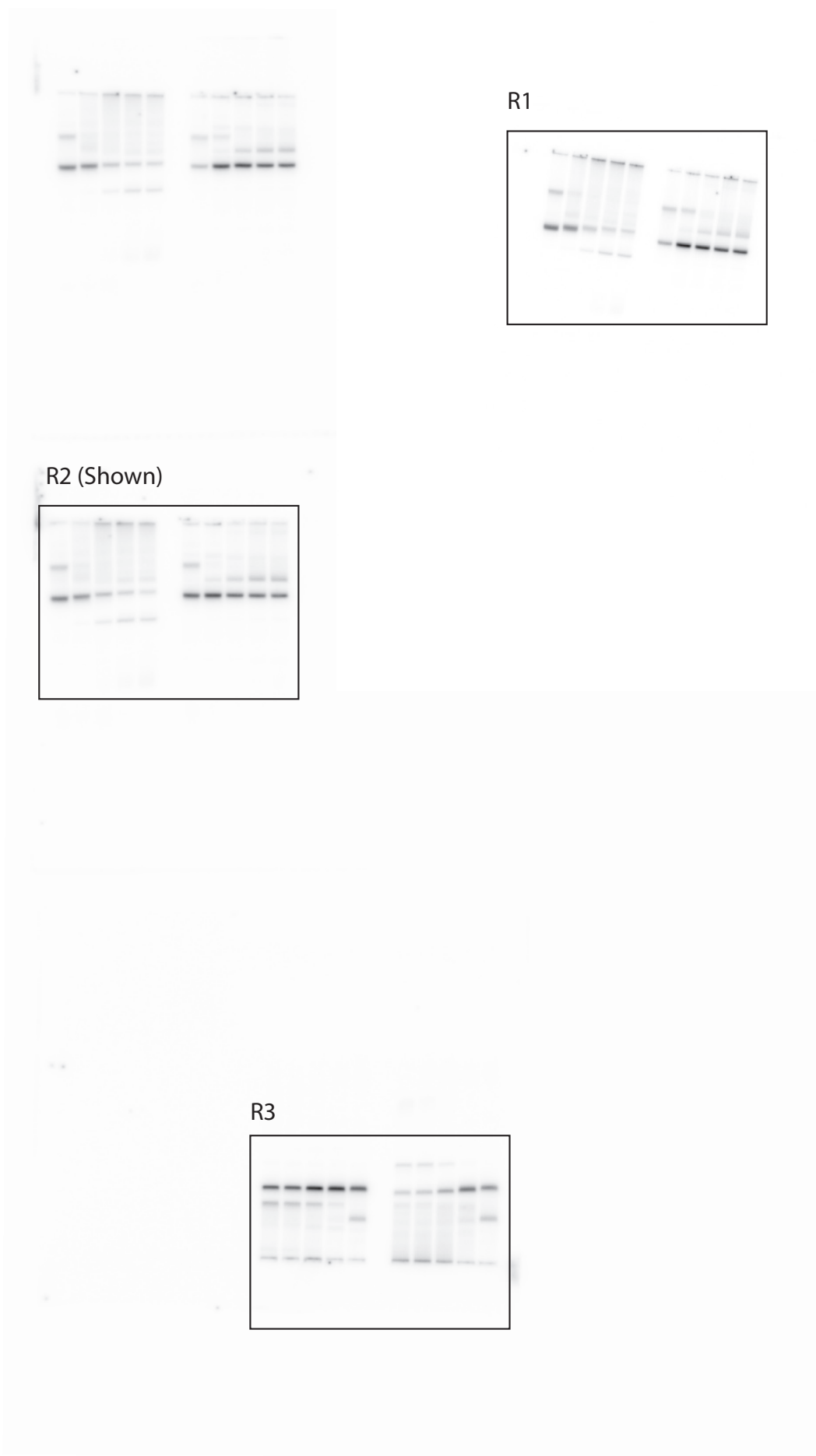

Figure 3J

R1 (Shown)

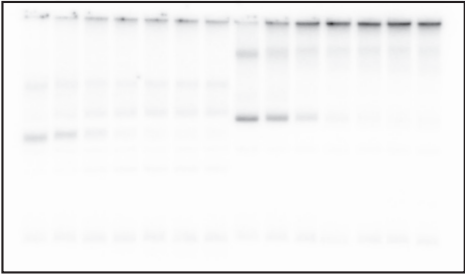

R2

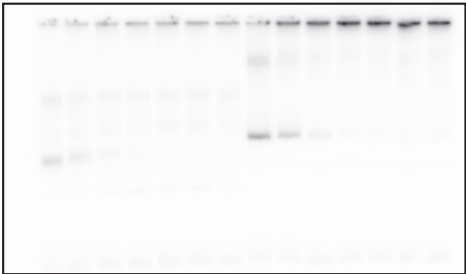

R3

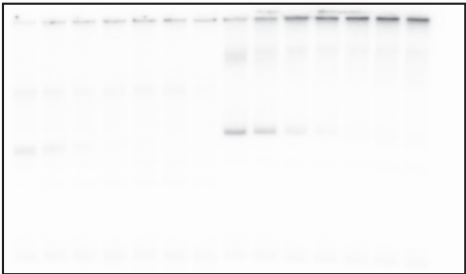

Supplement: Supplementary file 9 — Unprocessed gels and western blots. [file 41594_2026_1812_MOESM9_ESM.pdf]

Figure 4B

R1

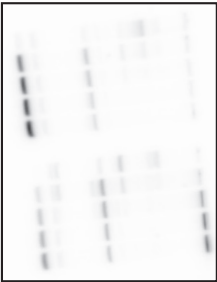

R2 (Shown)

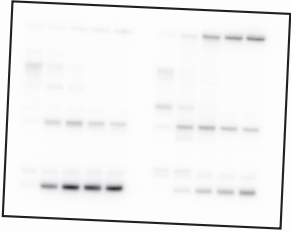

R3

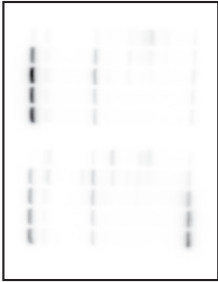

R4

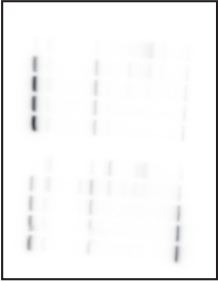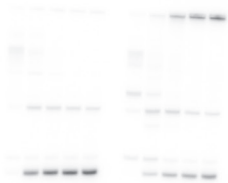

Figure 4G

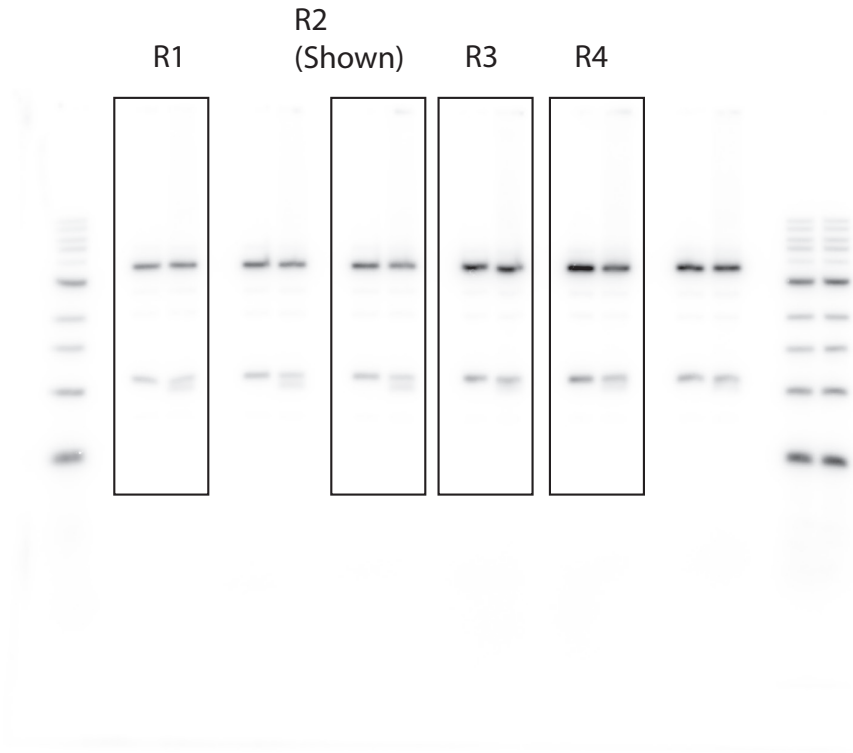

Figure 4J

R1

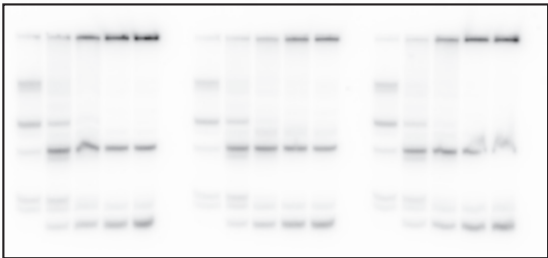

R2 (Shown)

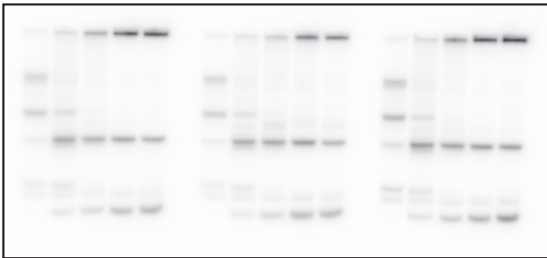

R3

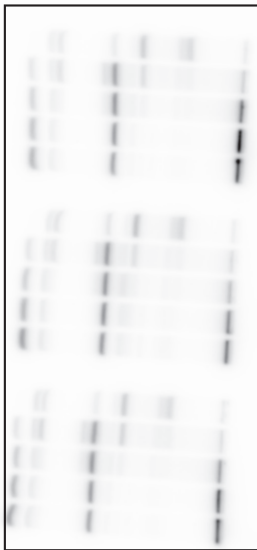

Supplement: Supplementary file 11 — Unprocessed gels and western blots. [file 41594_2026_1812_MOESM11_ESM.pdf]

Figure 5B

R3 (Shown)

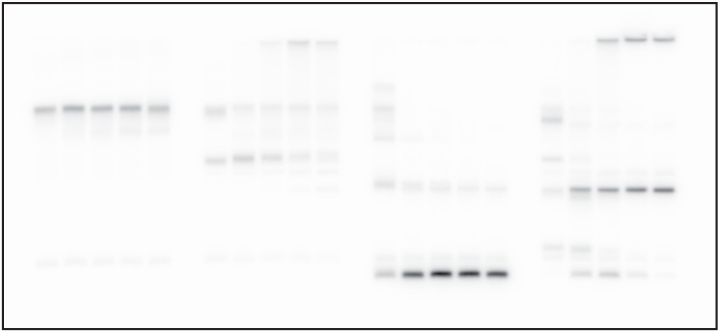

R2

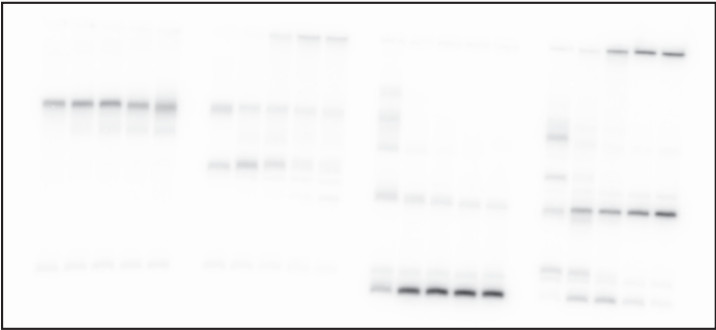

R1

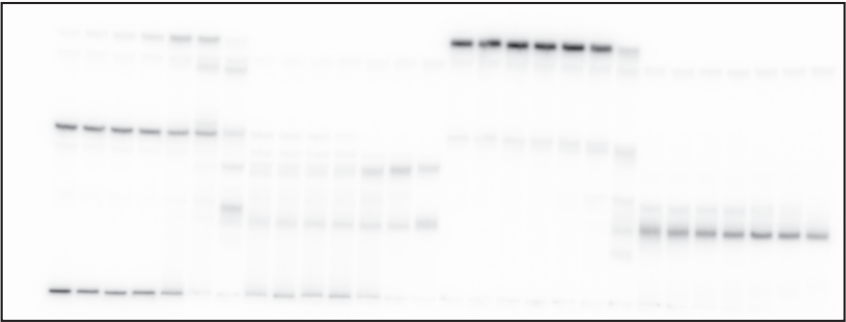

Figure 5F

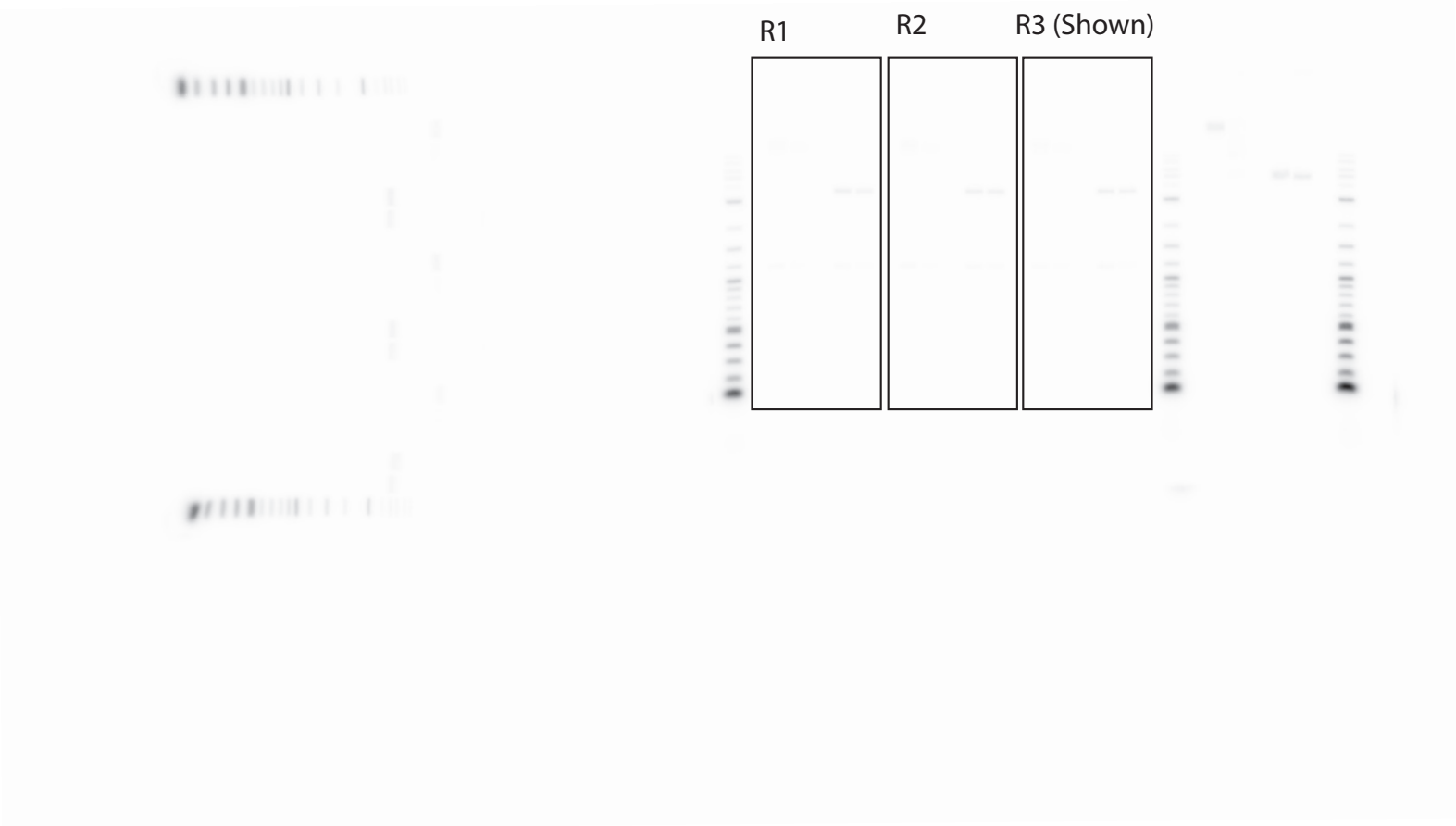

Figure 5H

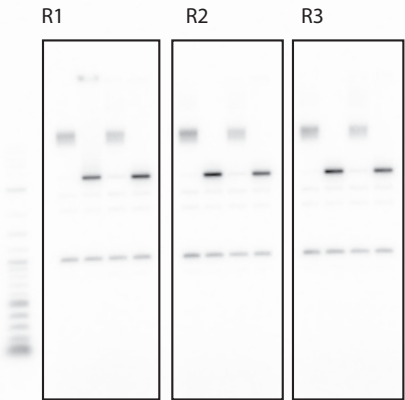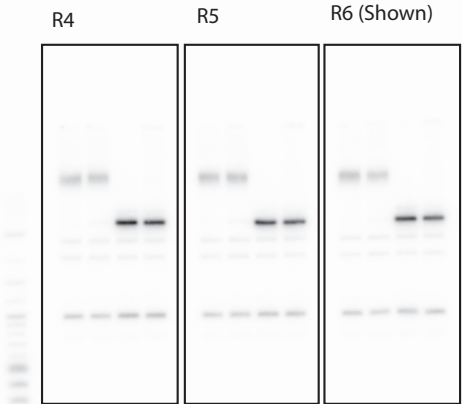

Supplement: Supplementary file 13 — Unprocessed gels and western blots. [file 41594_2026_1812_MOESM13_ESM.pdf]

Figure 6B

R1

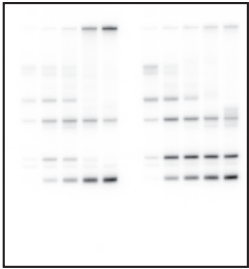

R2 (Shown)

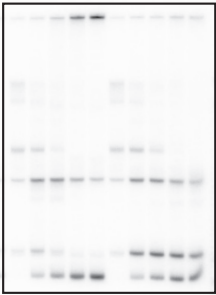

R3

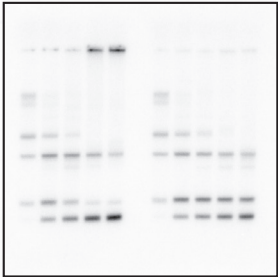

R4

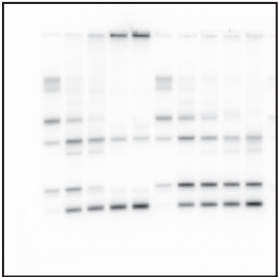

Figure 6G

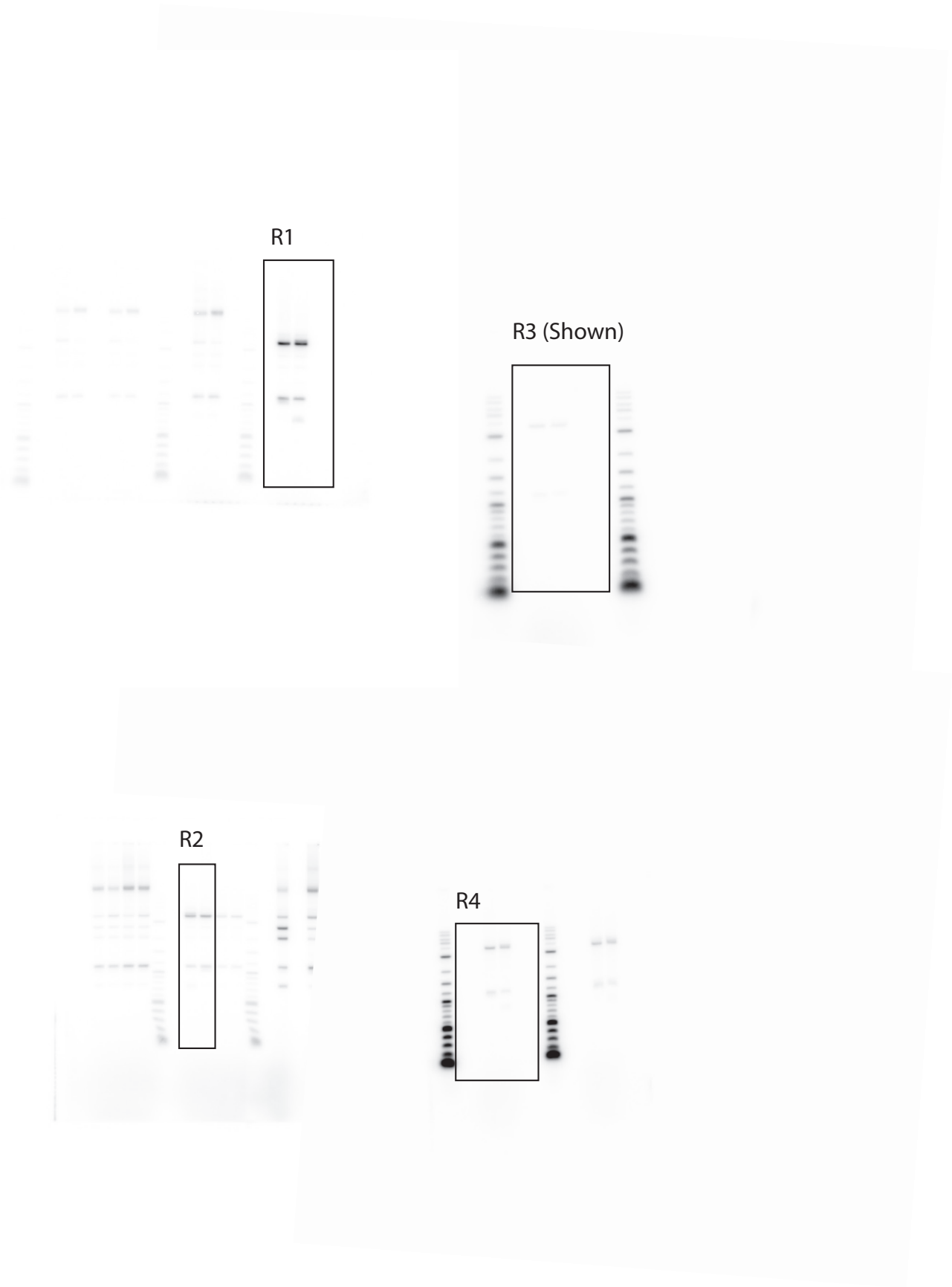

Figure 6J

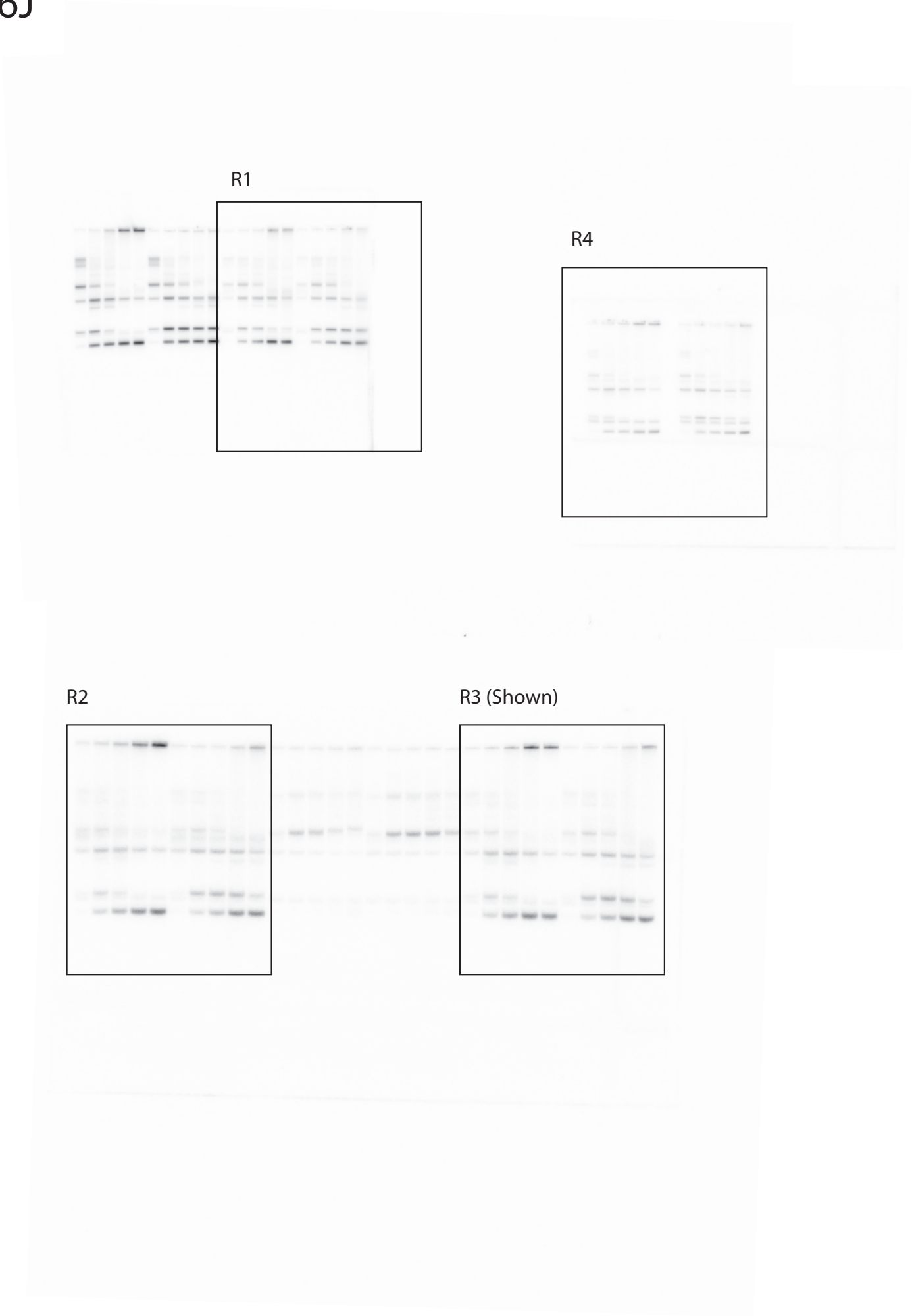

Figure 60

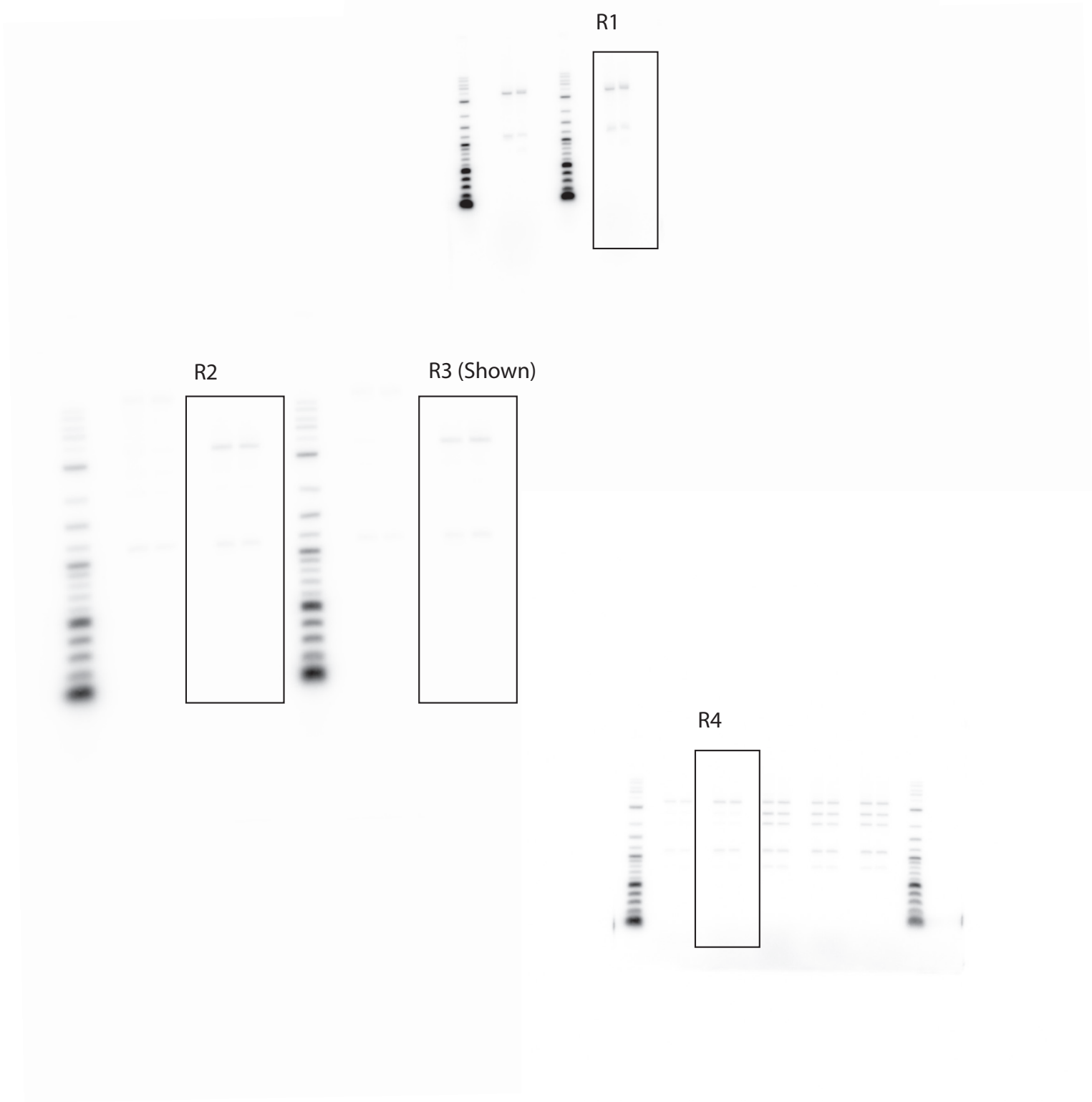

Supplement: Supplementary file 15 — Unprocessed gels and western blots. [file 41594_2026_1812_MOESM15_ESM.pdf]

ED Fig 1K

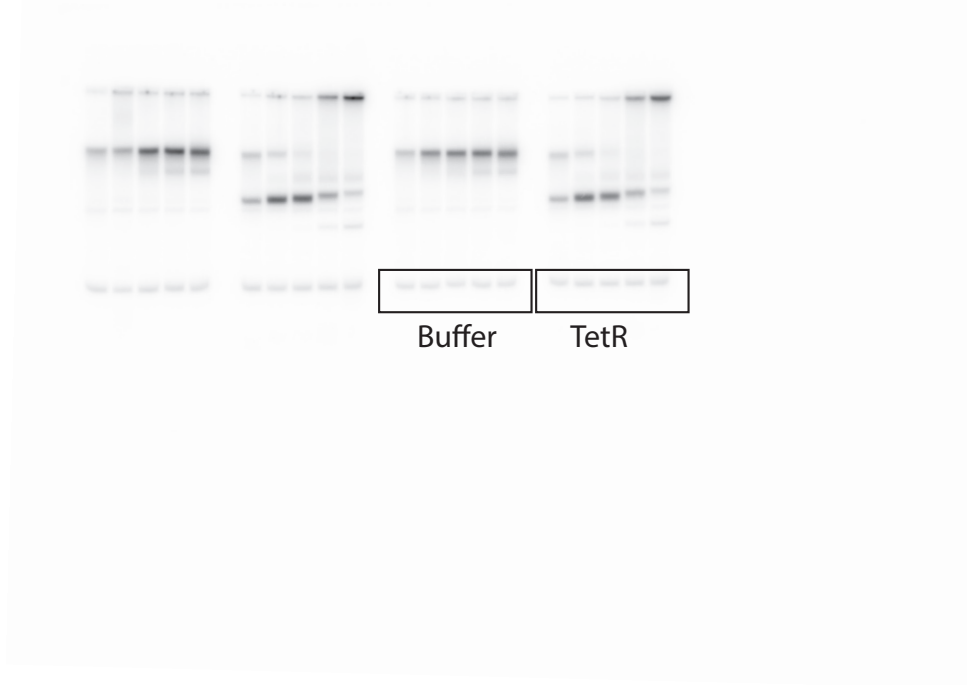

Supplement: Supplementary file 18 — Unprocessed gels and western blots. [file 41594_2026_1812_MOESM18_ESM.pdf]

# ED Figure 2A

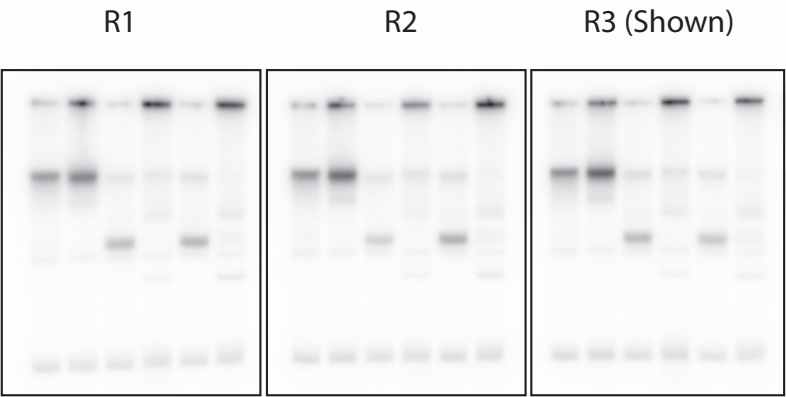

ED Figure 2D

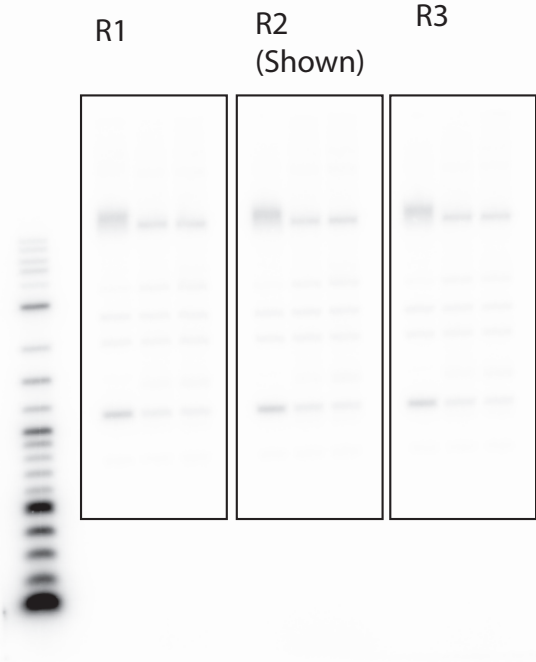

ED Figure 2G

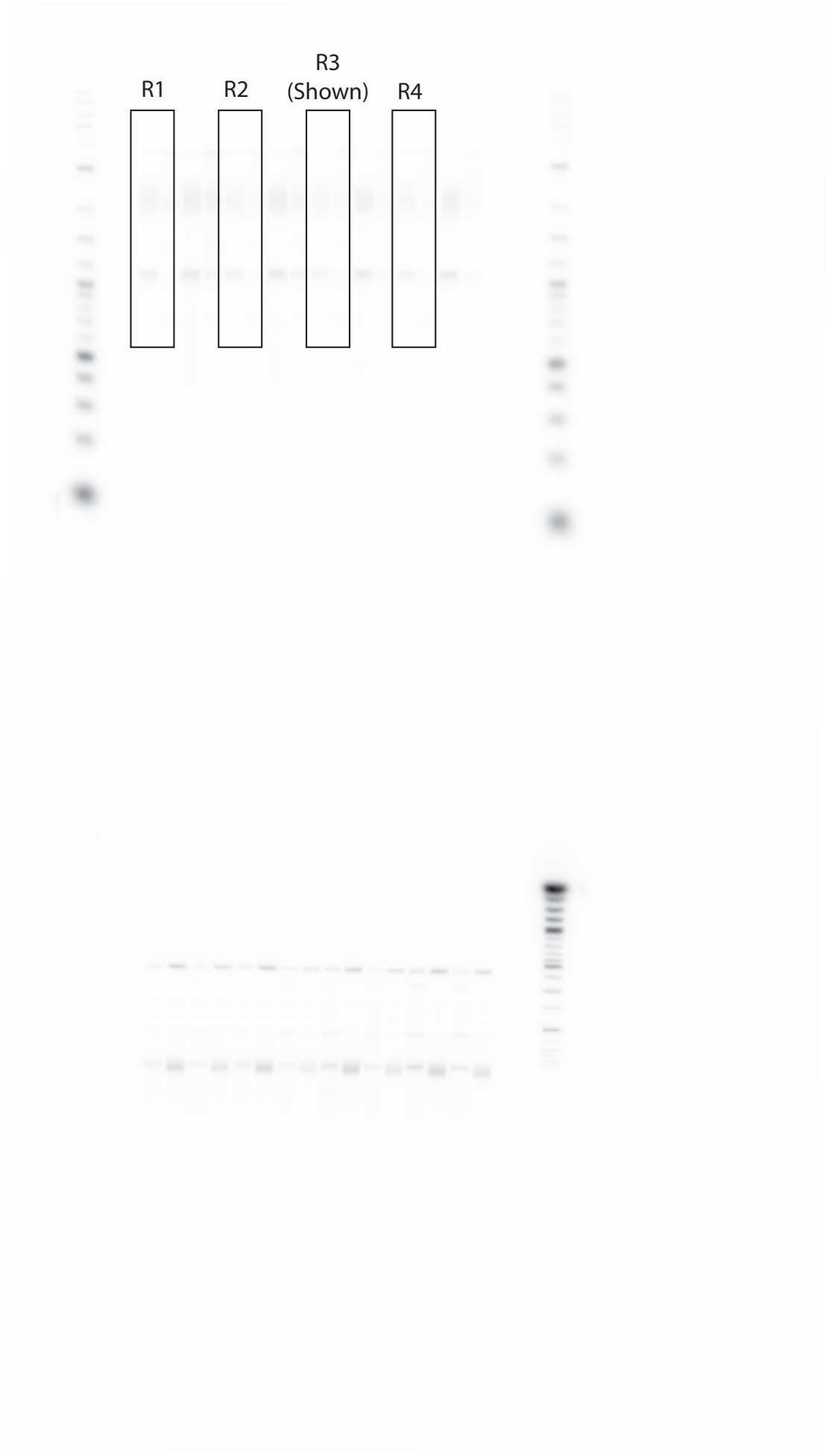

ED Figure 2J

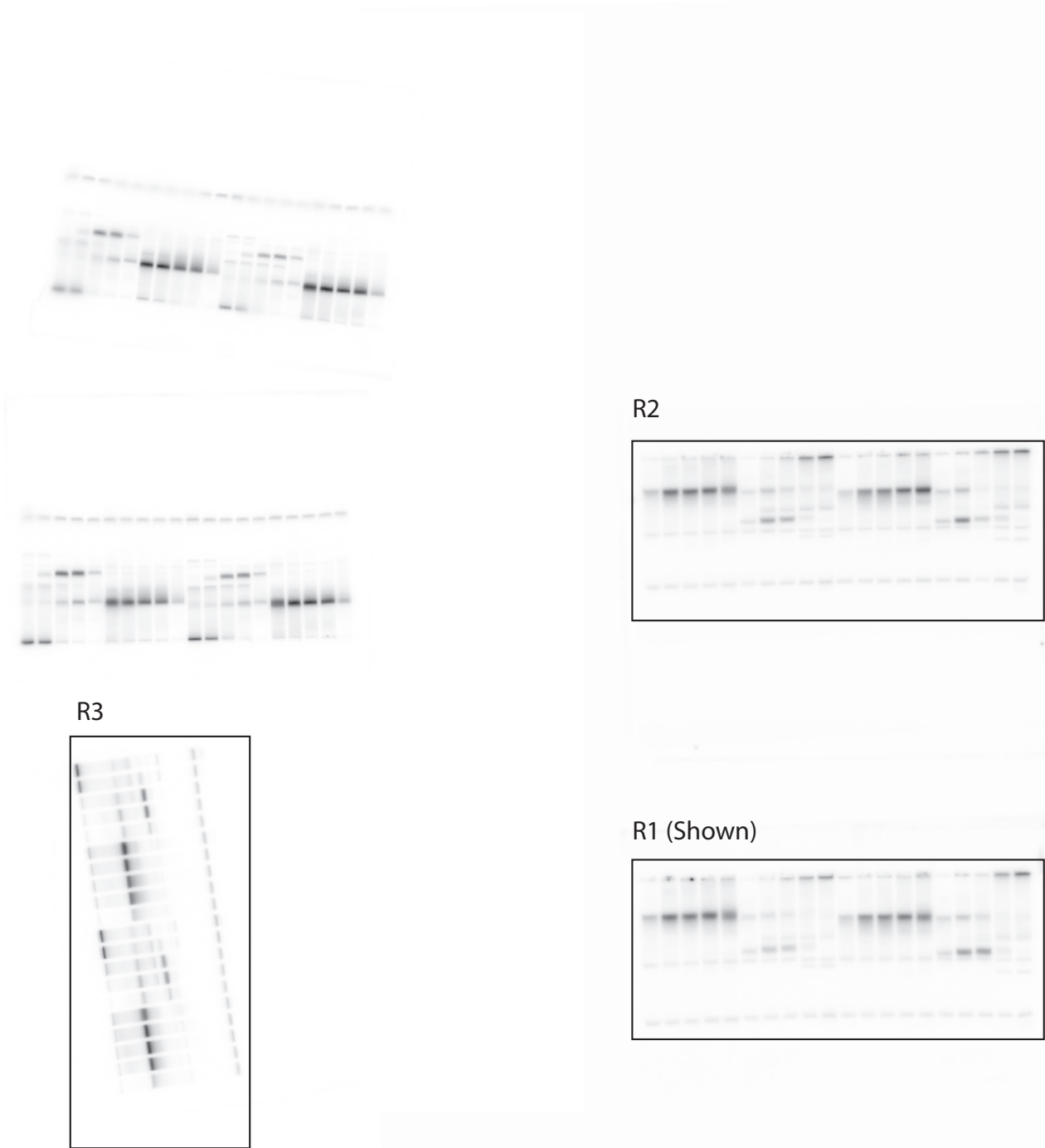

ED Figure 20

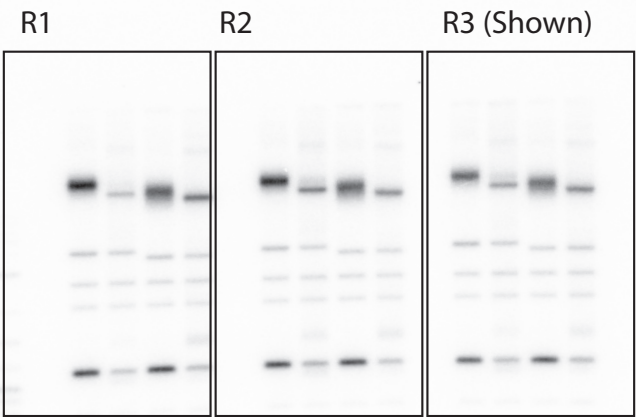

ED Figure 2Q

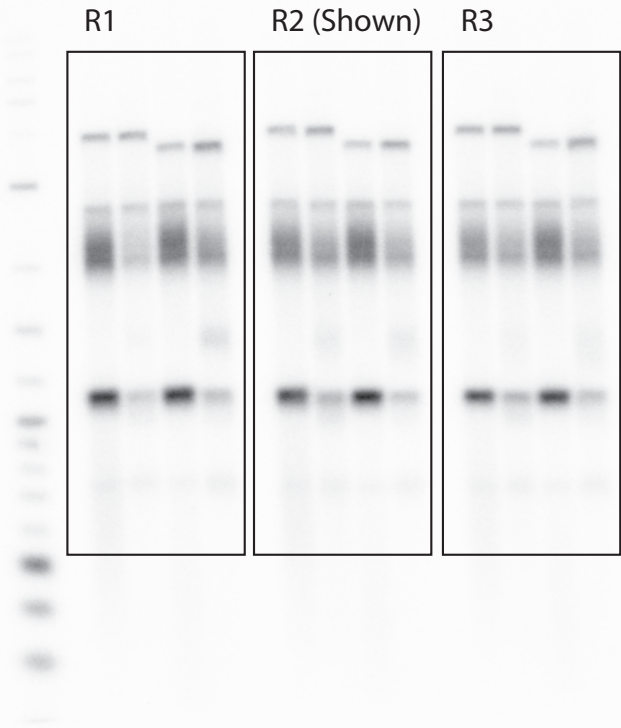

Supplement: Supplementary file 20 — Unprocessed gels and western blots. [file 41594_2026_1812_MOESM20_ESM.pdf]

ED Figure 3C

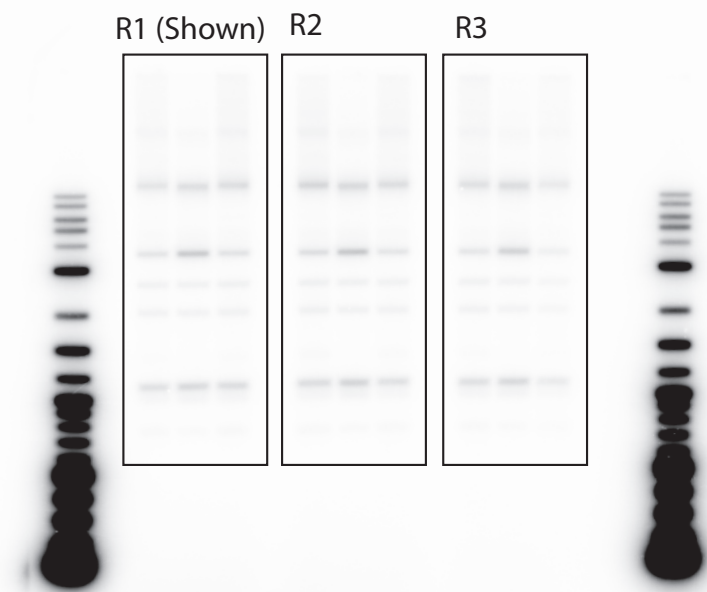

ED Figure 3G

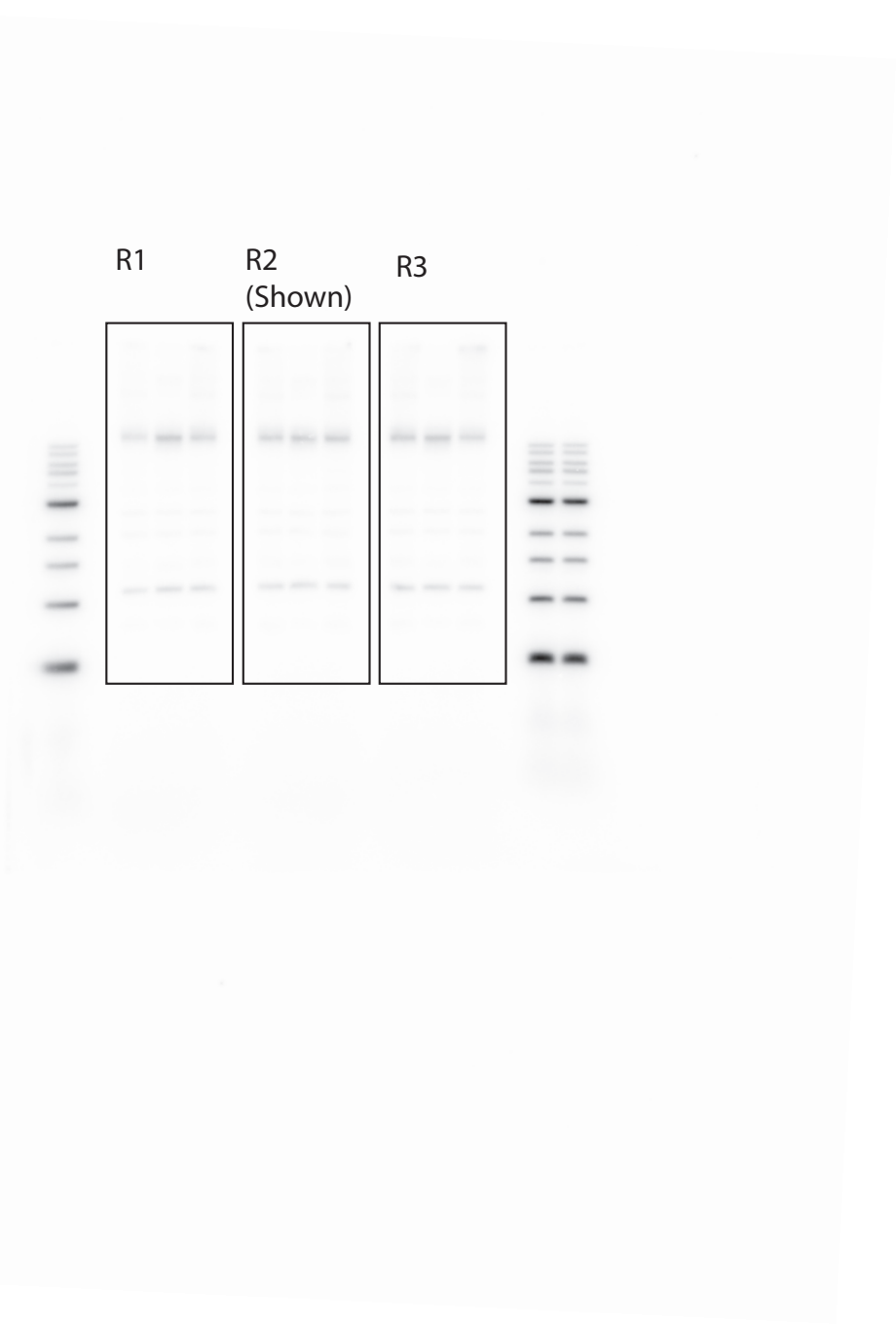

ED Figure 3K

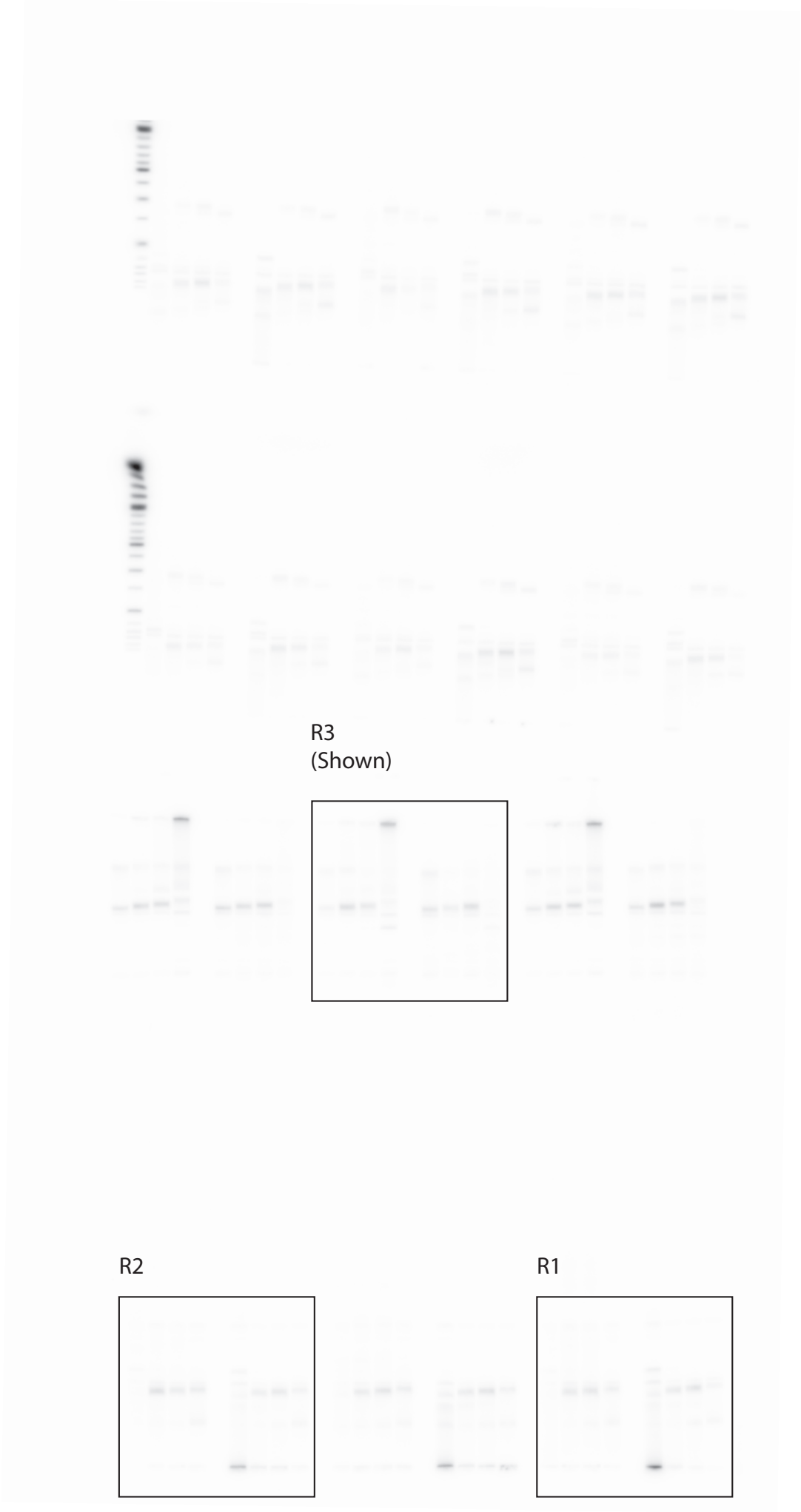

ED Figure 3L

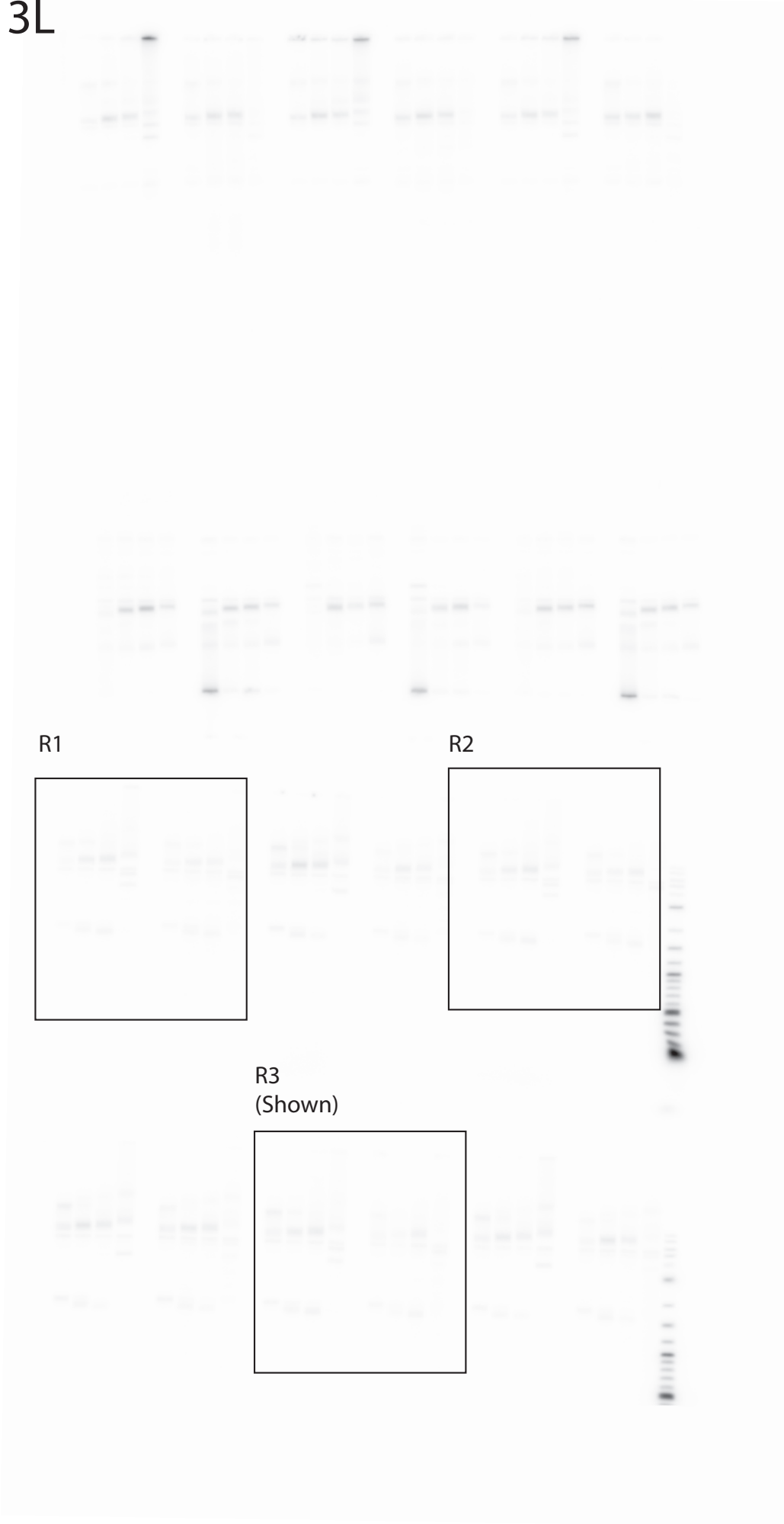

ED Figure 3M

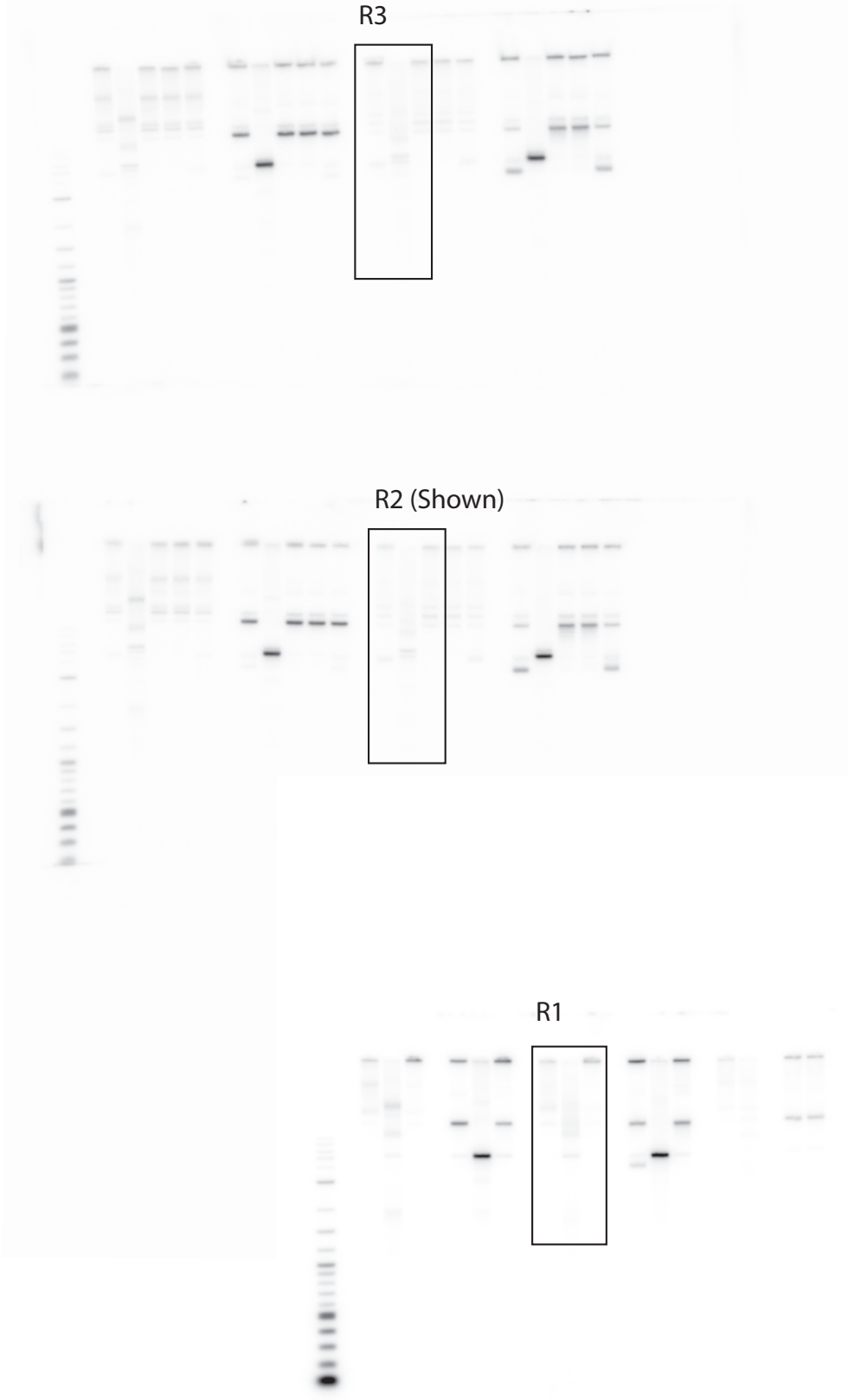

ED Figure 30

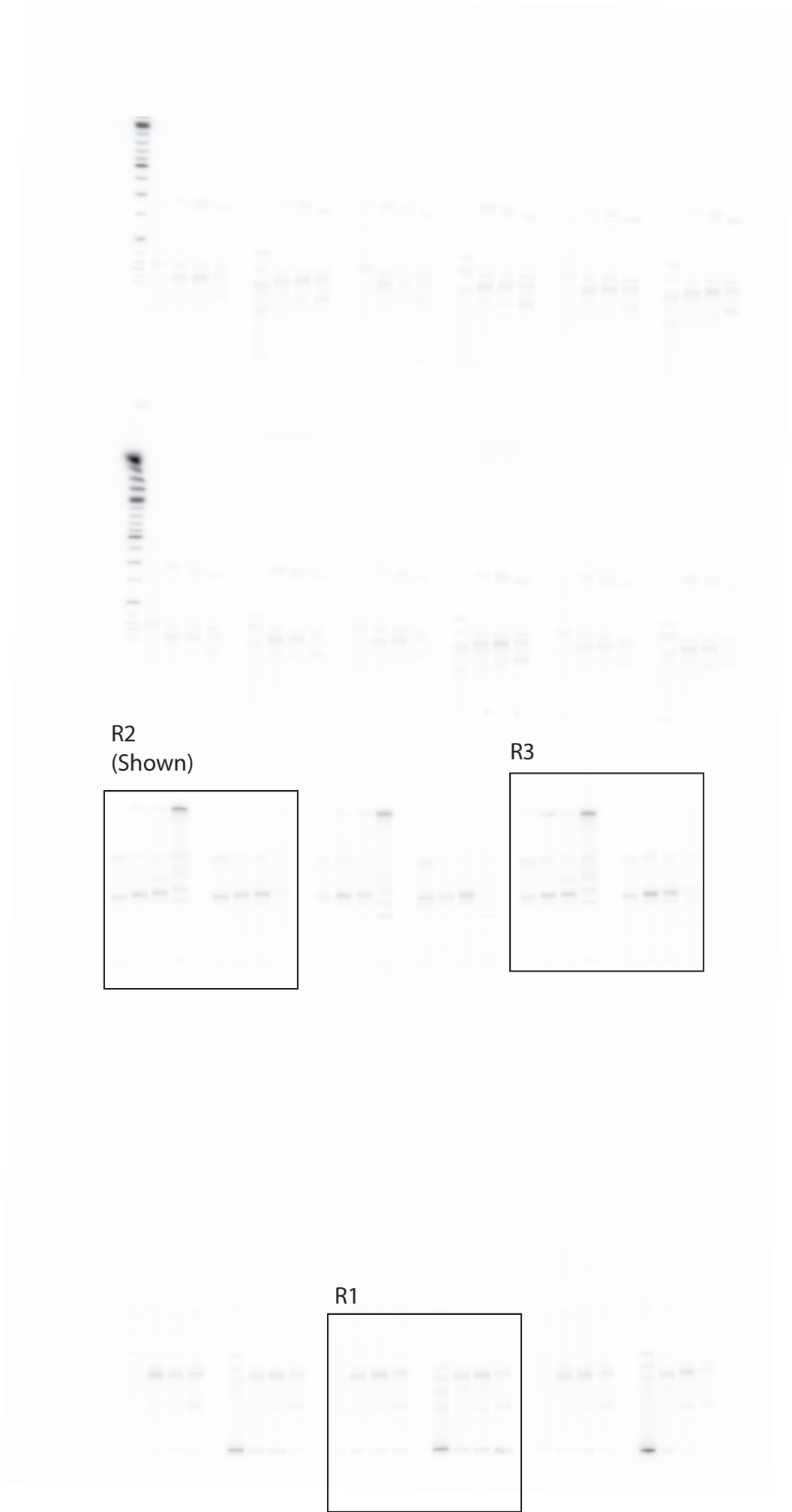

ED Figure 3P

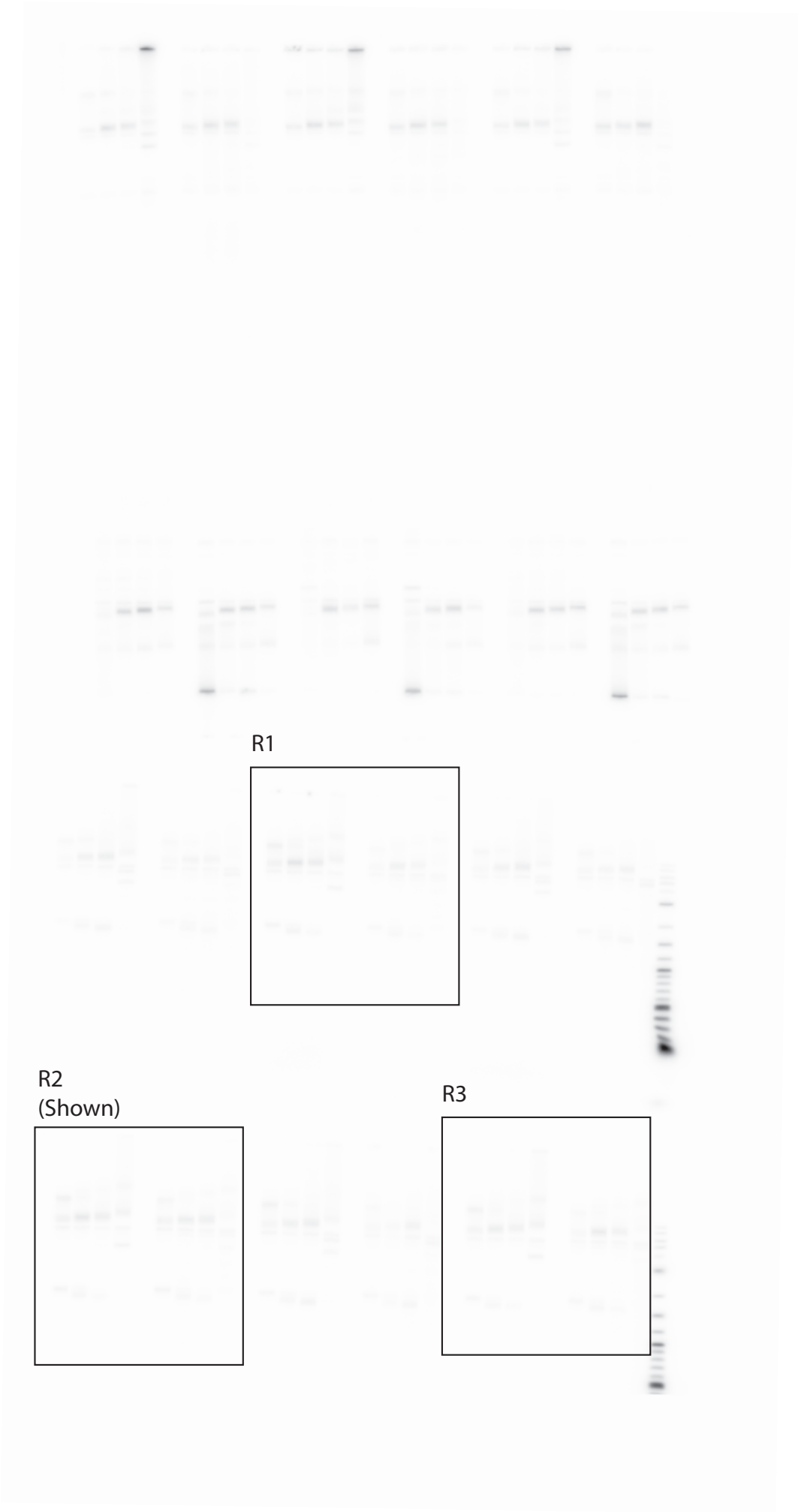

# ED Figure 3Q

R1

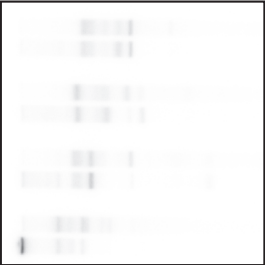

R2

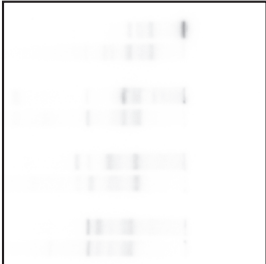

R3 (Shown)

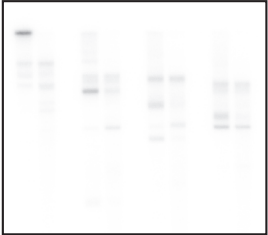

Supplement: Supplementary file 22 — Unprocessed gels and western blots. [file 41594_2026_1812_MOESM22_ESM.pdf]

ED Figure 5B

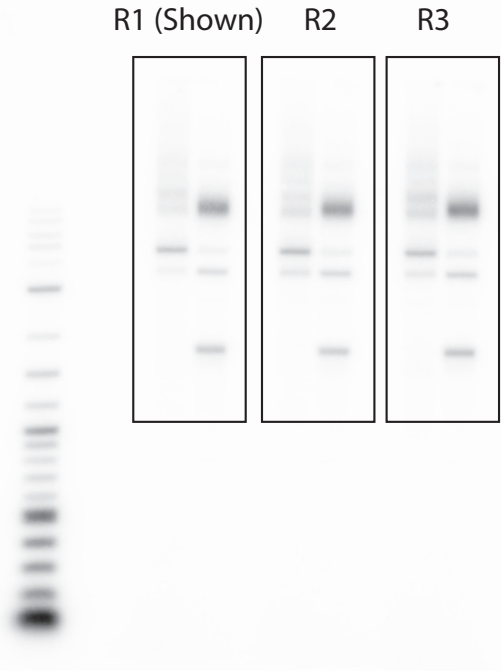

# ED Figure 5G

R1

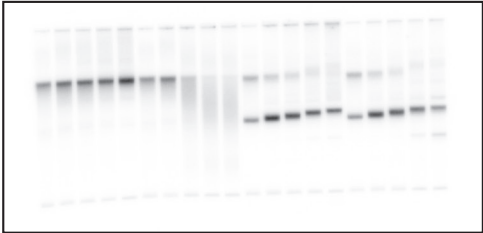

R2 (Shown)

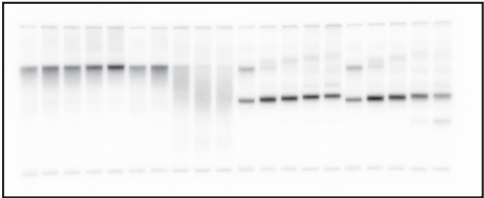

R3

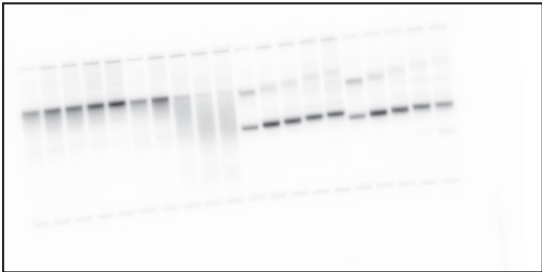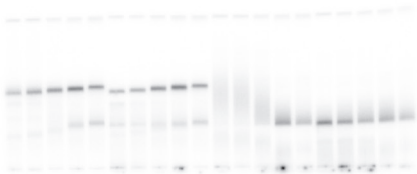

Supplement: Supplementary file 25 — Unprocessed gels and western blots. [file 41594_2026_1812_MOESM25_ESM.pdf]

# ED Figure 6D

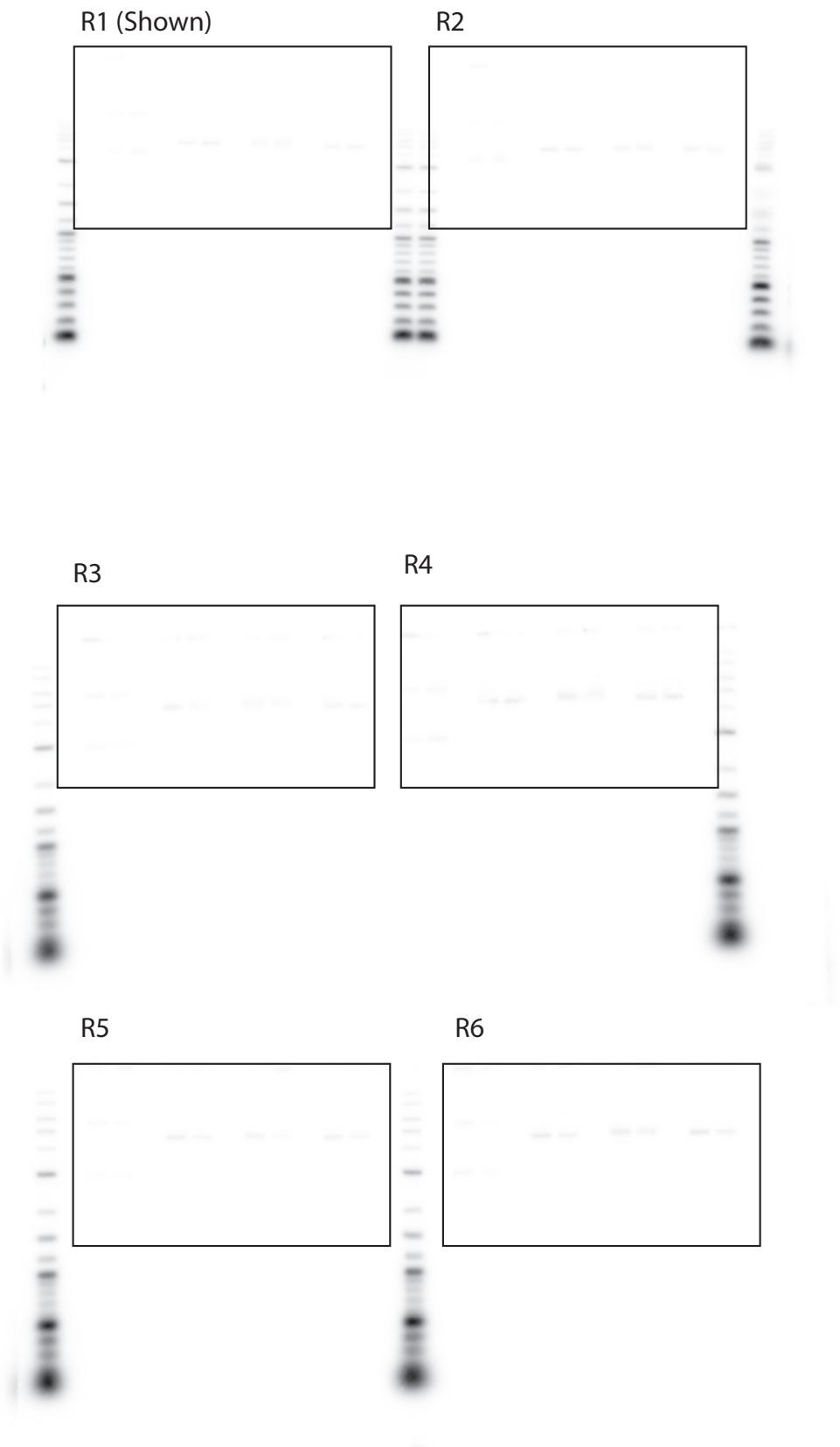

ED Figure 6E

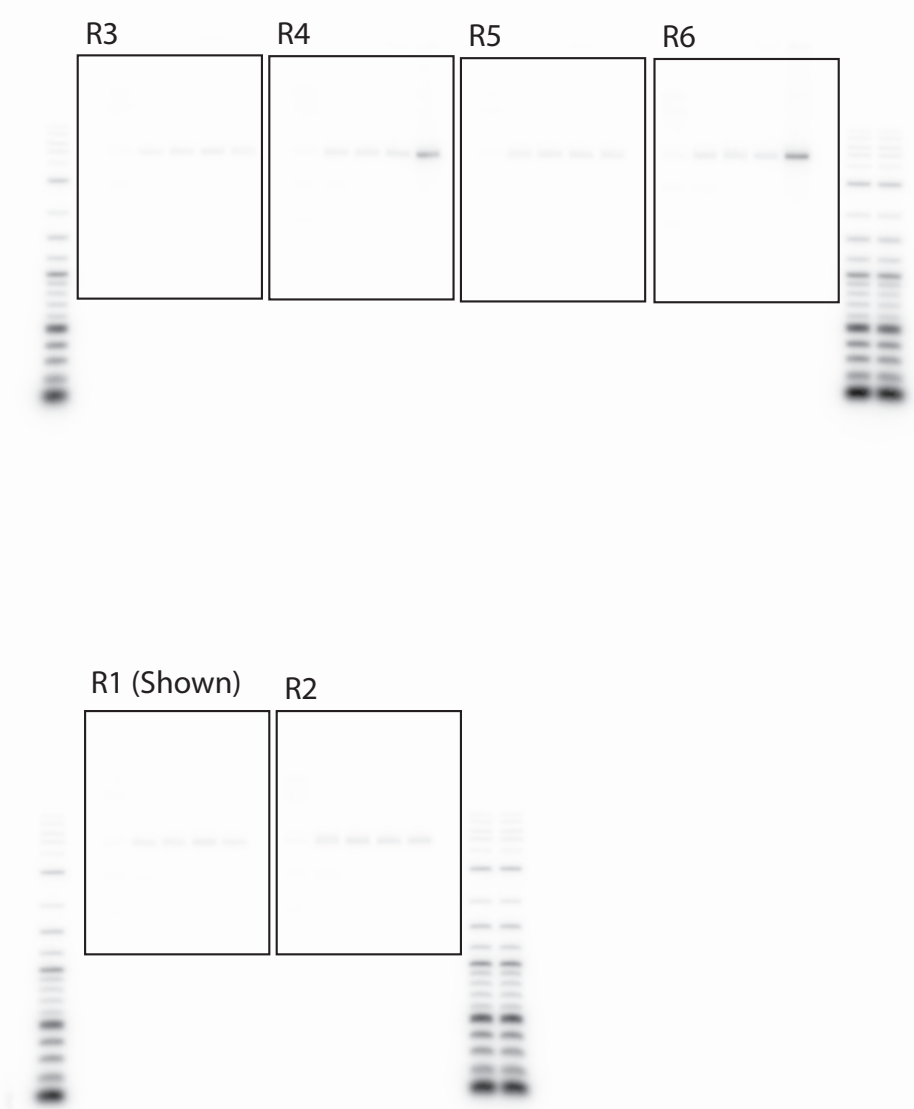

# ED Figure 6H

R3 (Shown)

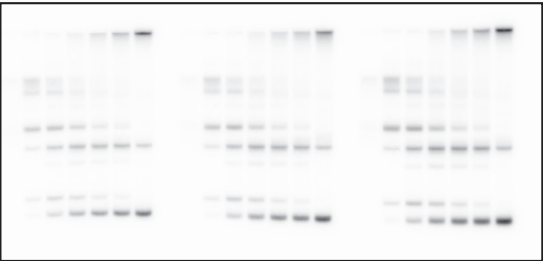

R2

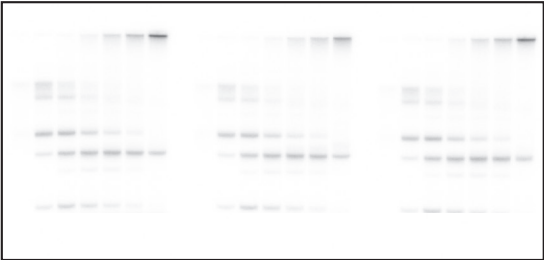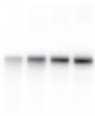

R1

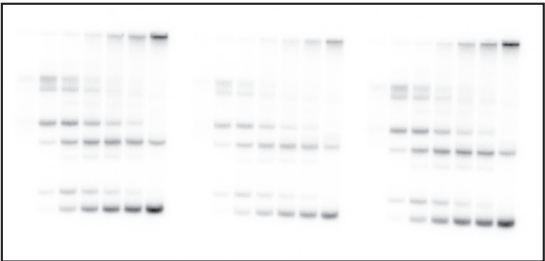

Supplement: Supplementary file 27 — Unprocessed gels and western blots. [file 41594_2026_1812_MOESM27_ESM.pdf]

ED Figure 7B

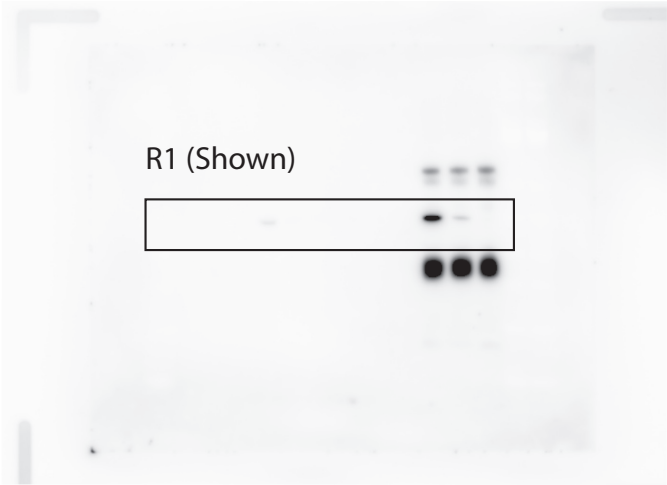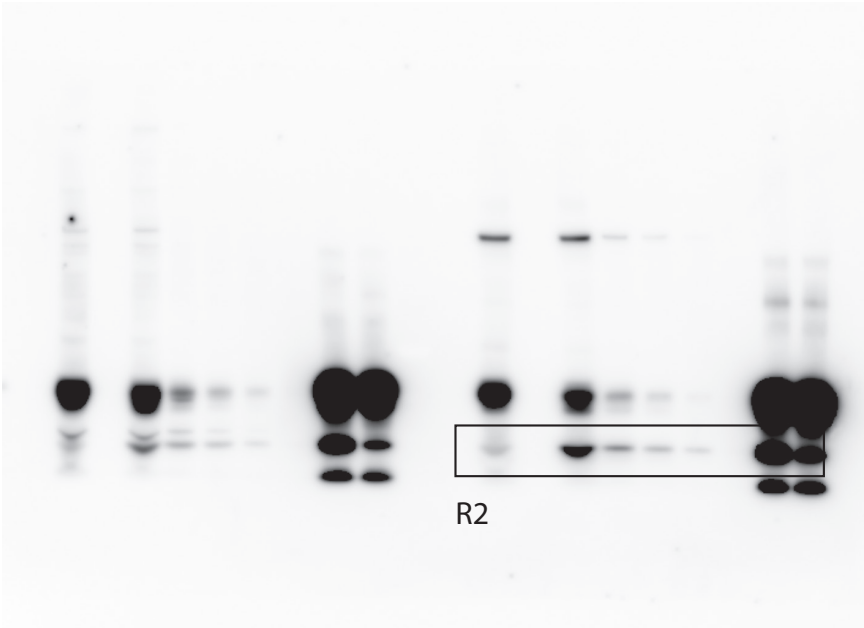

# ED Figure 7C

R3 (Shown)

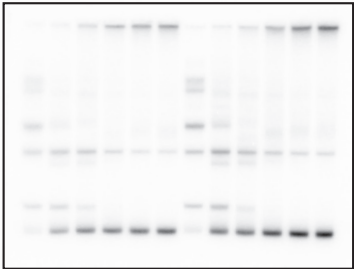

R2

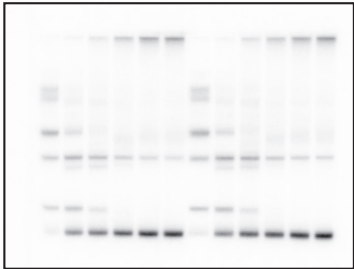

R1

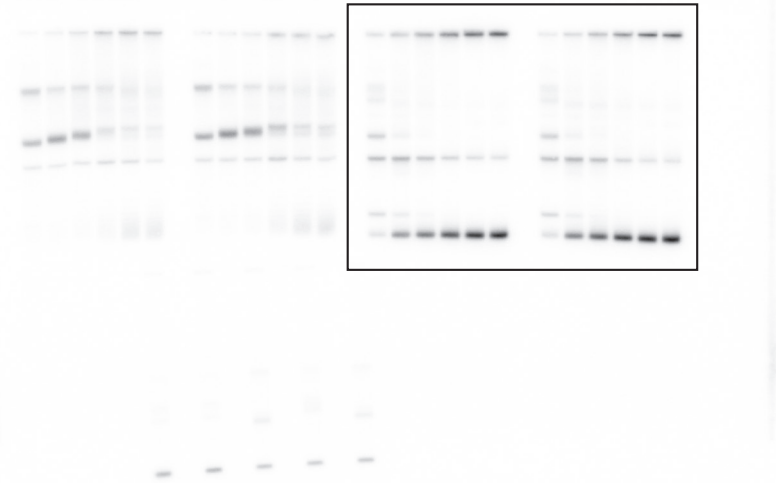

ED Figure 7G

R2 (Shown)

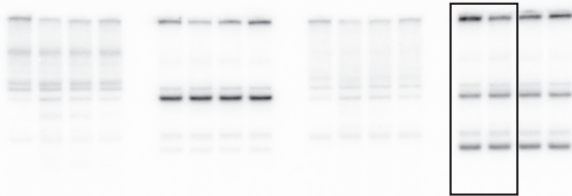

R3

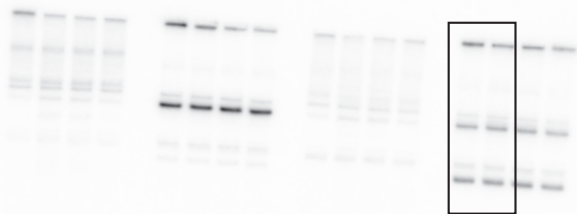

R1

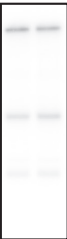

ED Figure 7H

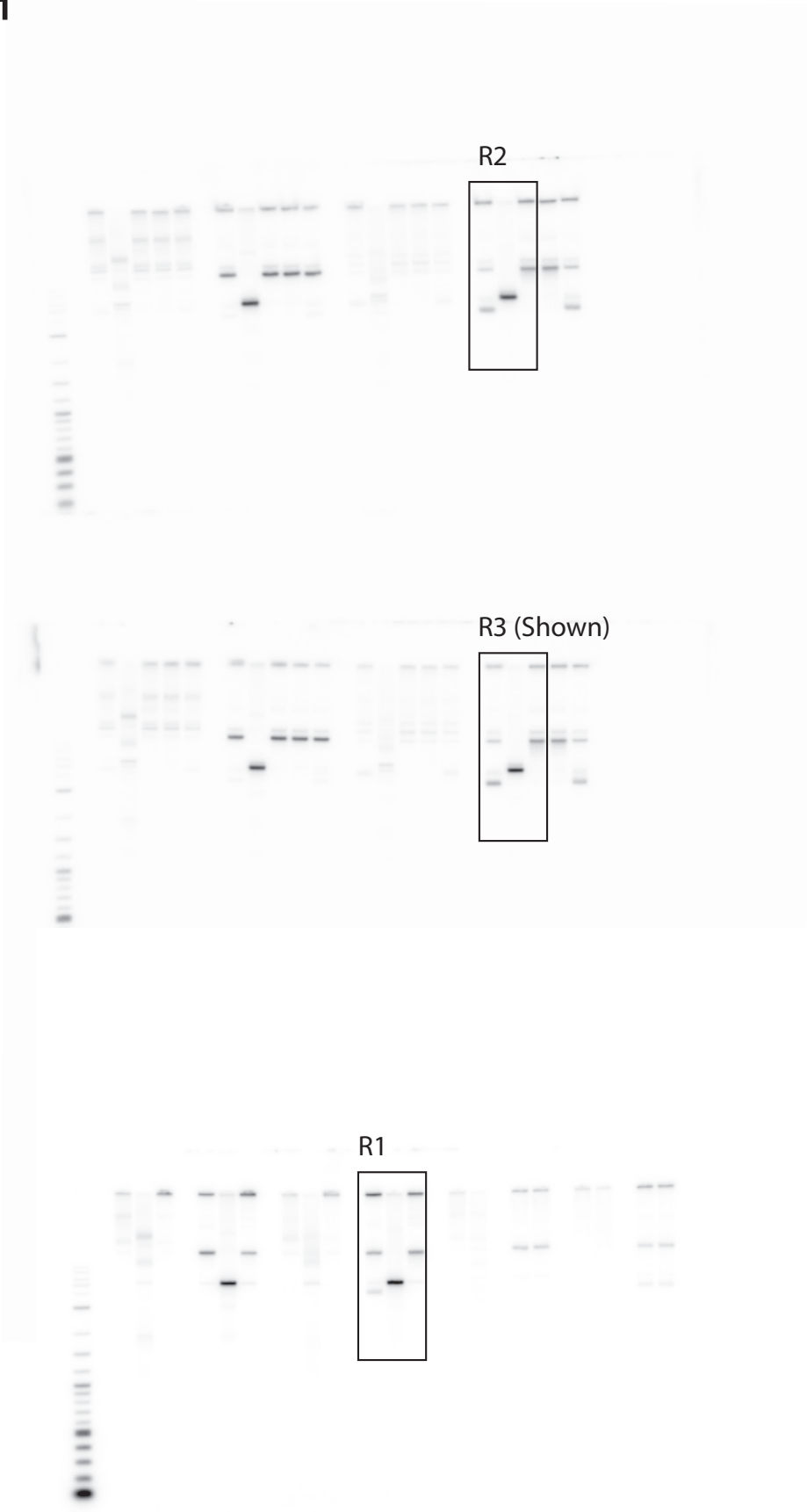

ED Figure 7J

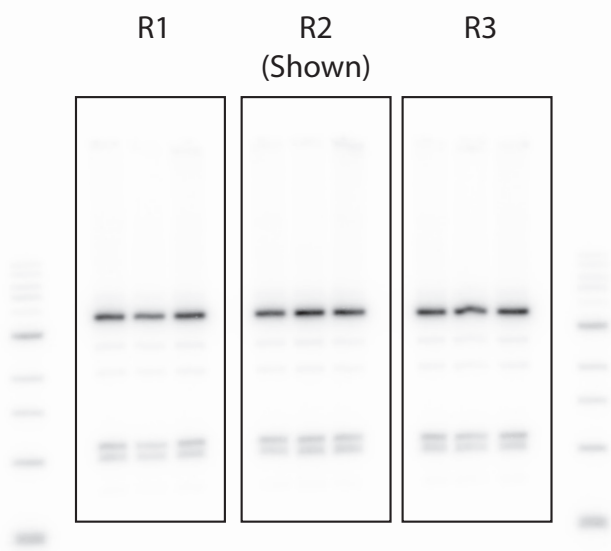

Supplement: Supplementary file 29 — Unprocessed gels and western blots. [file 41594_2026_1812_MOESM29_ESM.pdf]
