# Supplementary material for: Distinct repair outcomes from single and convergent replication fork collapse
Source: Nat Struct Mol Biol. 2026 May 27;33(6):939–52. doi: 10.1038/s41594-026-01812-9 (PMC13275508; doi:10.1038/s41594-026-01812-9)

ED Figure 8F

R1 (Shown)

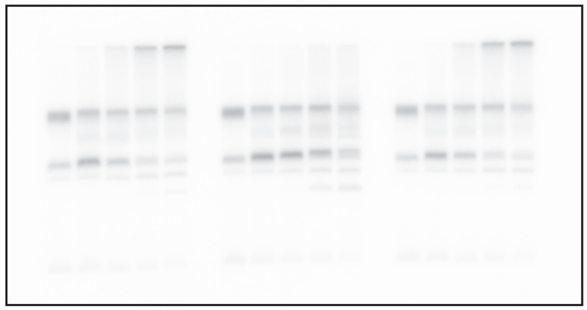

R3

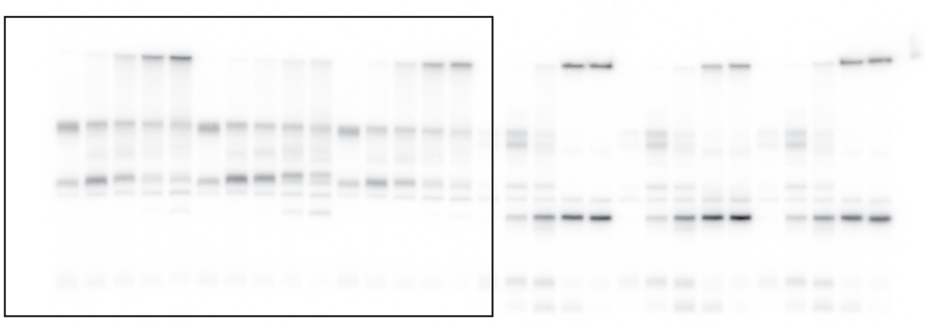

R2

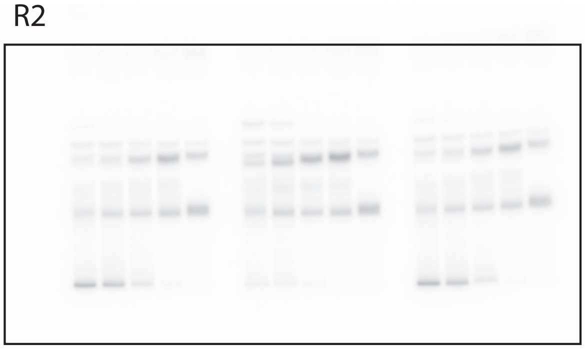

ED Figure 8l

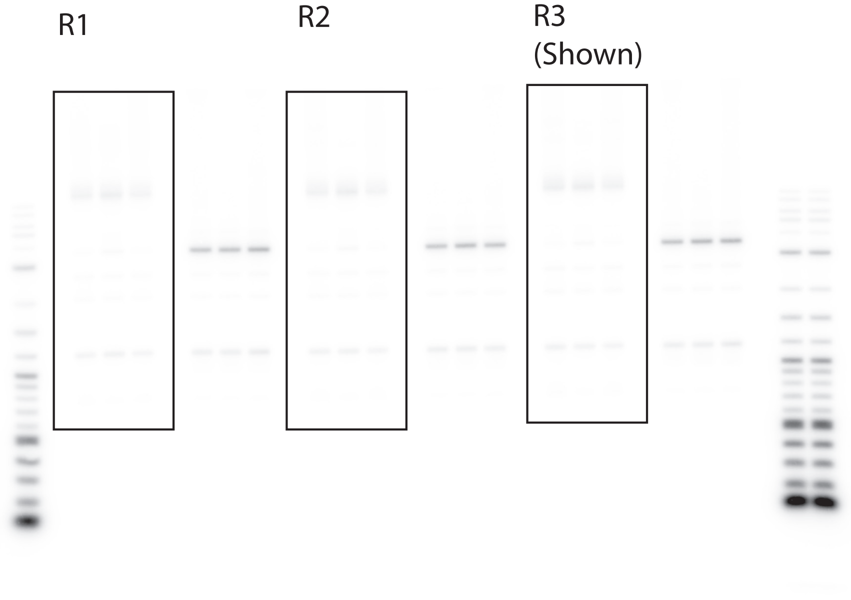

ED Figure 8K

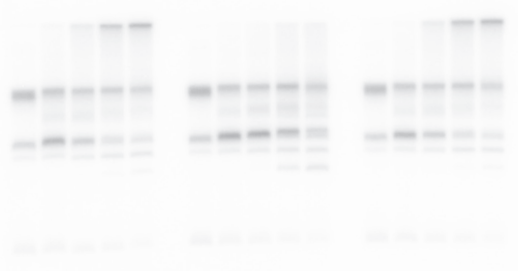

R3

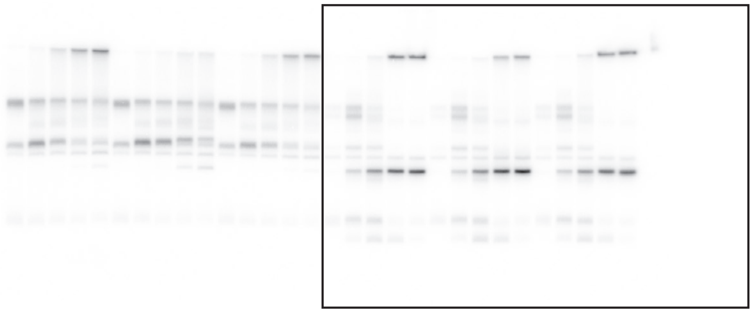

R1 (Shown)

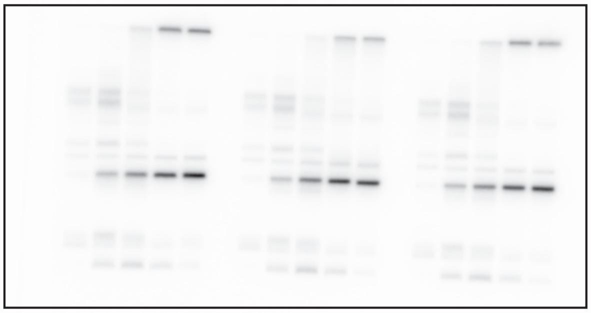

R2

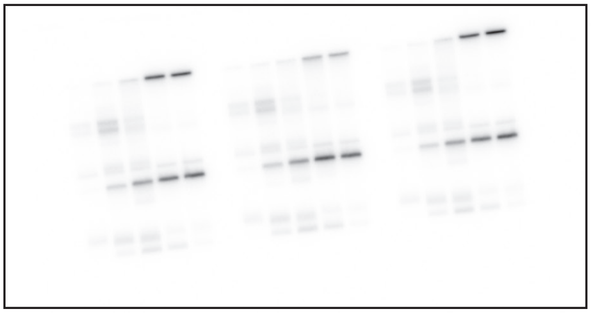

ED Figure 8N

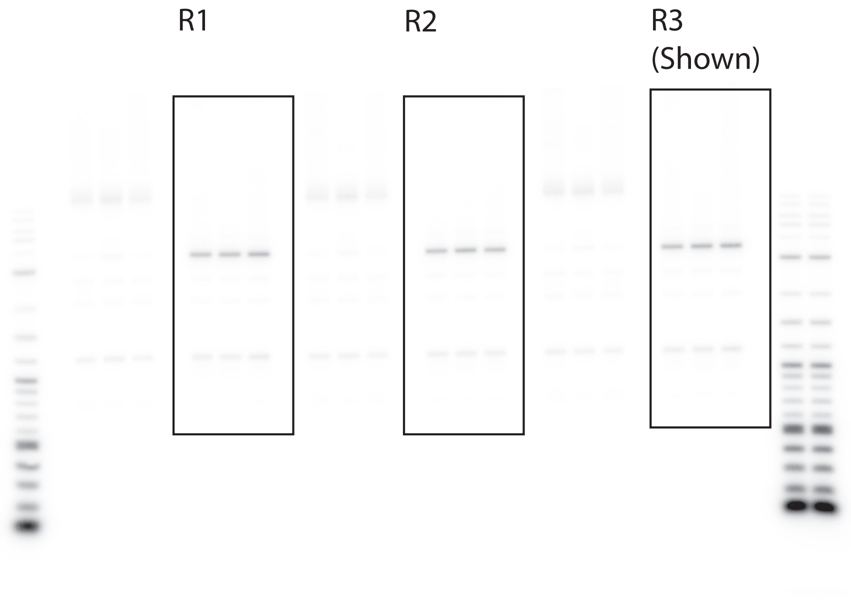

ED Figure 8P

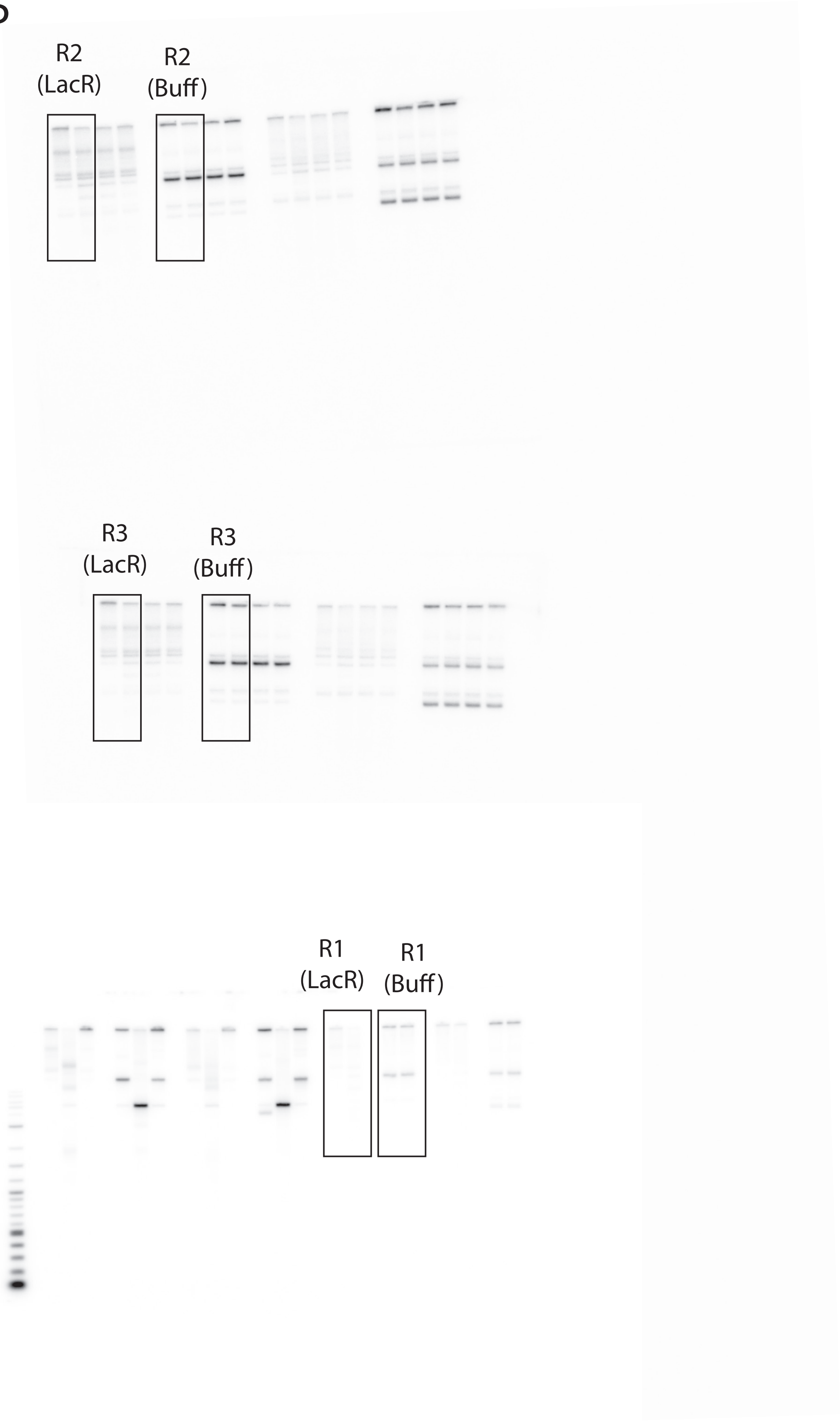

ED Figure 8Q

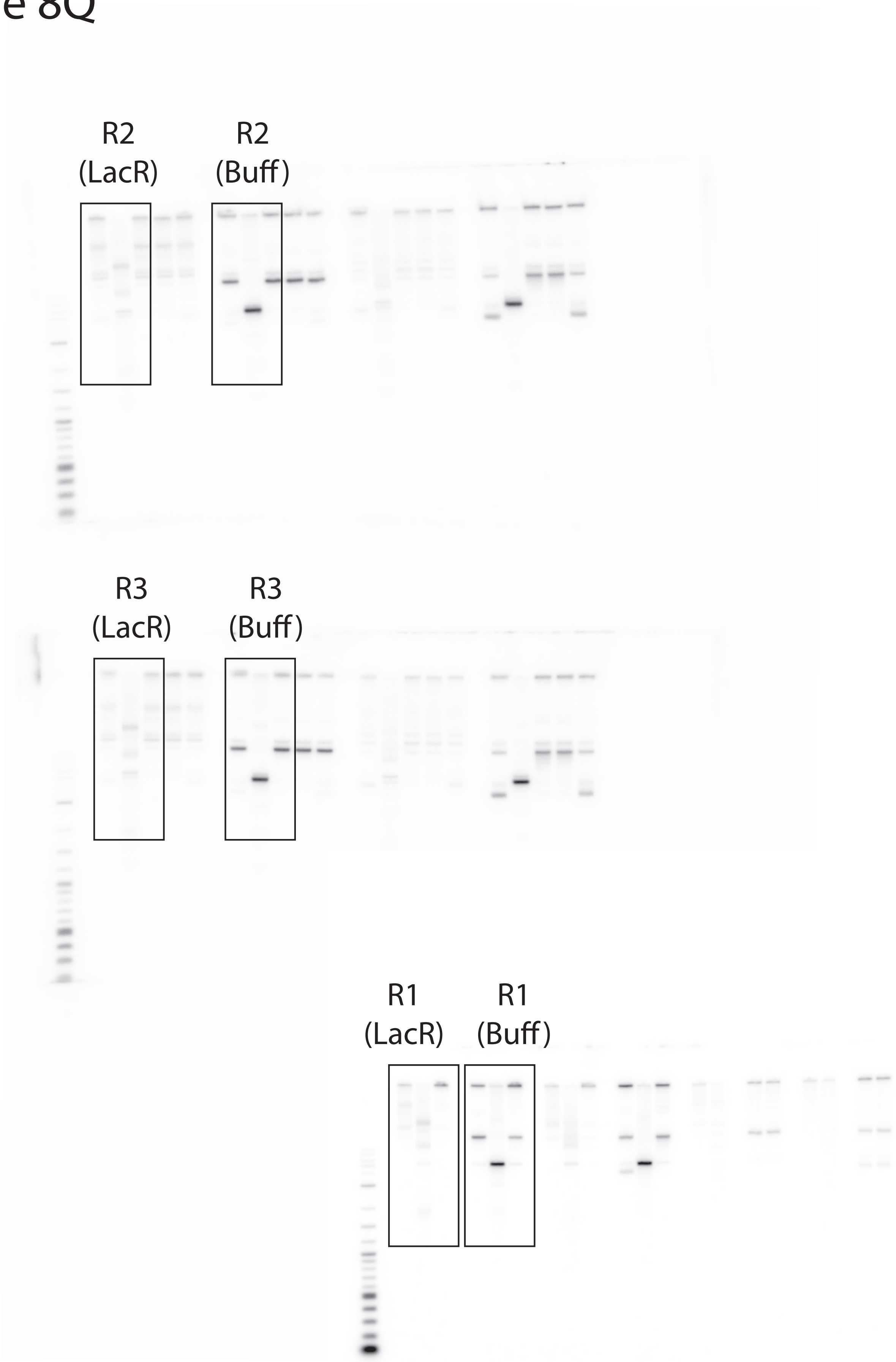

ED Figure 8S

R1 (Shown)

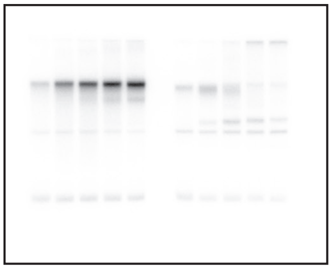

R3

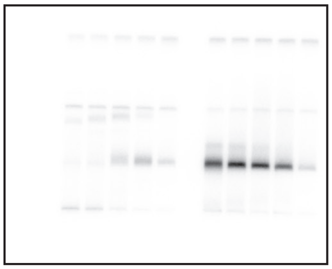

R2

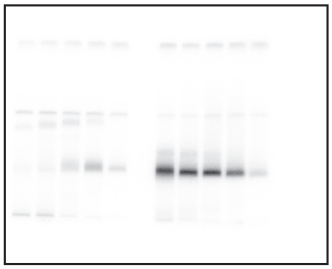

ED Figure 8T

R3

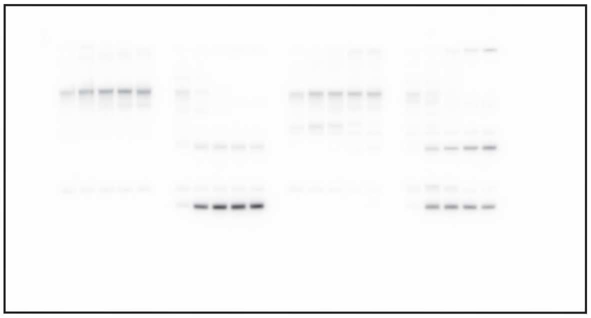

R2

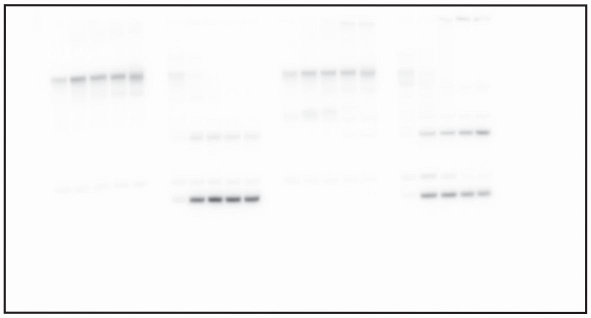

R1

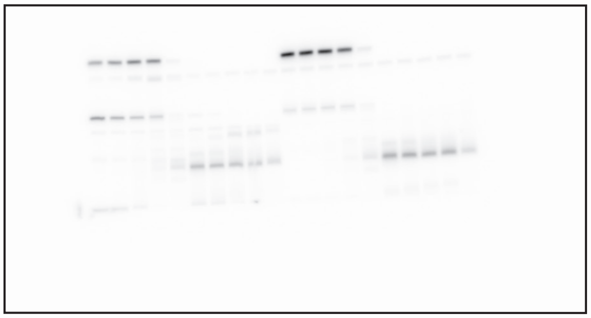

R4

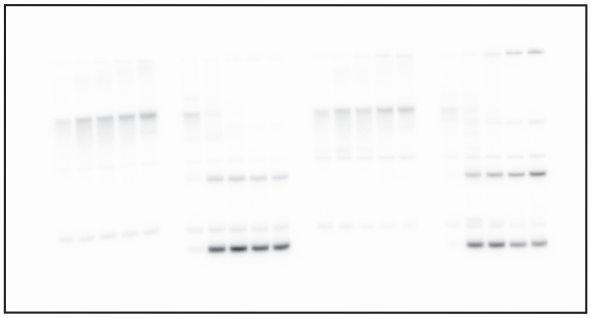

R5

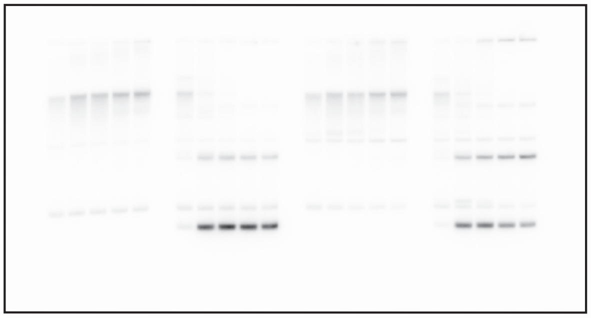

R6 (Shown)

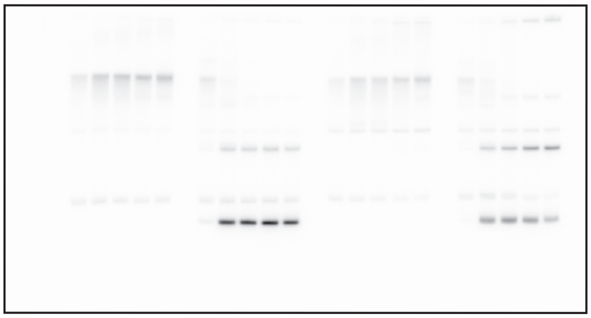

Supplement: Supplementary file 31 — Unprocessed gels and western blots. [file 41594_2026_1812_MOESM31_ESM.pdf]
