# Supplementary material for: Distinct repair outcomes from single and convergent replication fork collapse
Source: Nat Struct Mol Biol. 2026 May 27;33(6):939–52. doi: 10.1038/s41594-026-01812-9 (PMC13275508; doi:10.1038/s41594-026-01812-9)
Supplement: Supplementary file 33 — Unprocessed gels and western blots. [file 41594_2026_1812_MOESM33_ESM.pdf]

ED Figure 9A

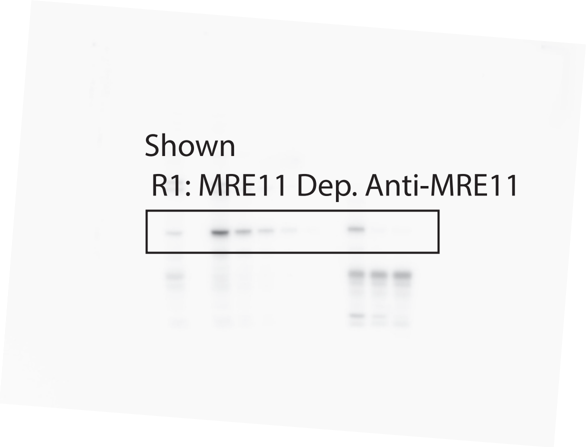
Shown
R1: MRE11 Dep. Anti-MRE11

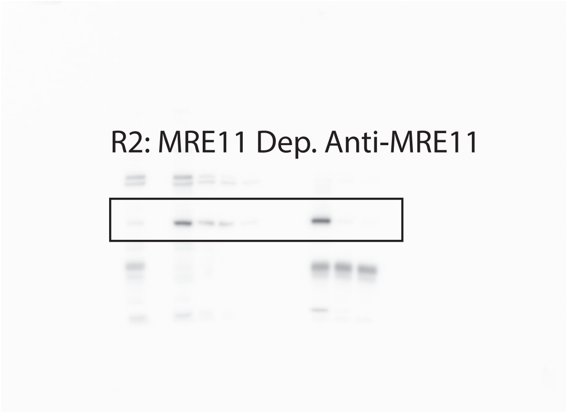
R2: MRE11 Dep. Anti-MRE11

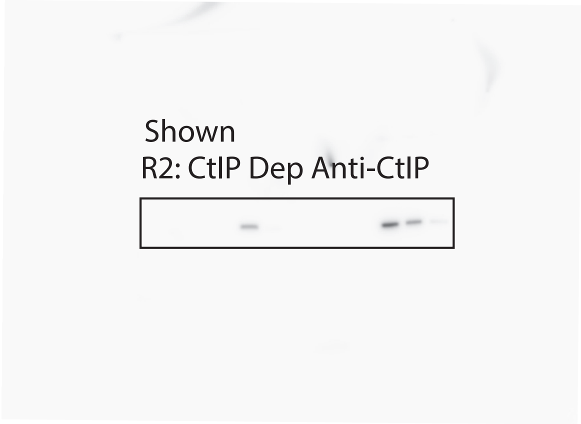
Shown
R2: CtIP Dep Anti-CtIP

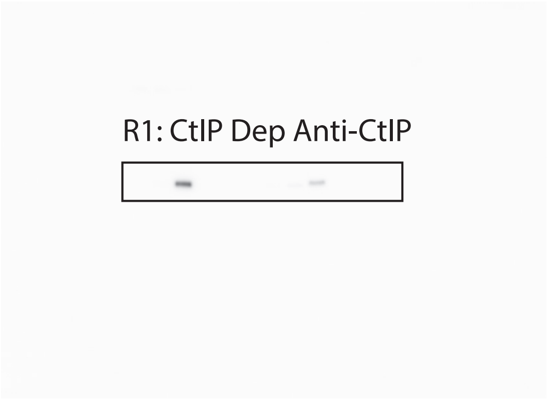
R1: CtIP Dep Anti-CtIP

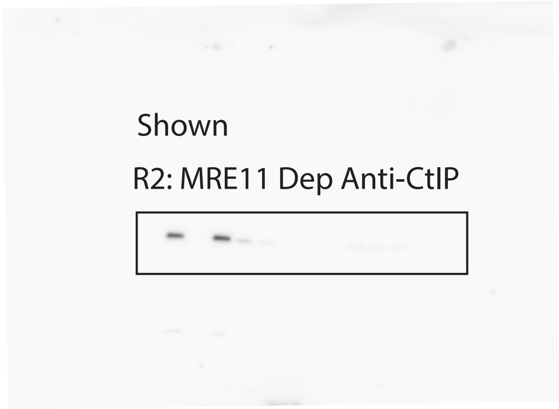
Shown
R2: MRE11 Dep Anti-CtIP

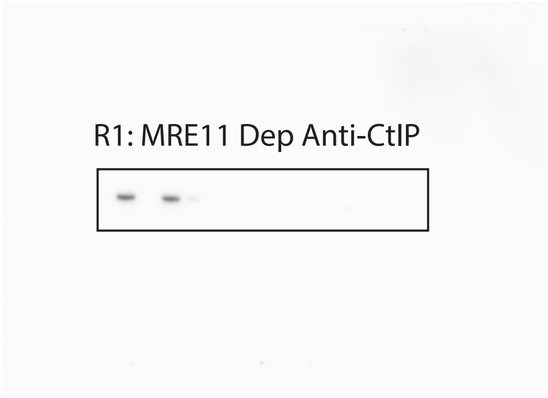
R1: MRE11 Dep Anti-CtIP

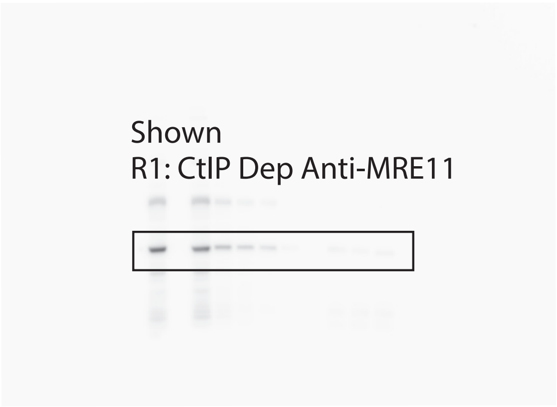
Shown
R1: CtIP Dep Anti-MRE11

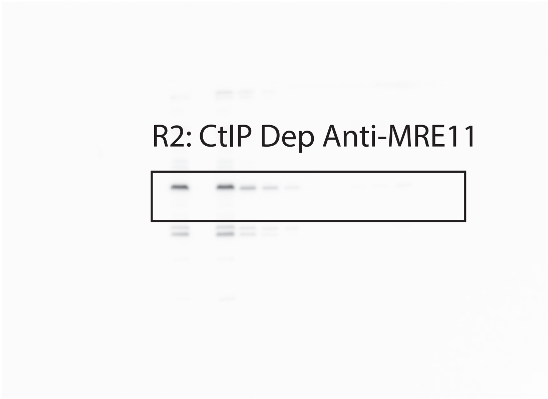
R2: CtIP Dep Anti-MRE11
