# Supplementary material for: Distinct repair outcomes from single and convergent replication fork collapse
Source: Nat Struct Mol Biol. 2026 May 27;33(6):939–52. doi: 10.1038/s41594-026-01812-9 (PMC13275508; doi:10.1038/s41594-026-01812-9)
Supplement: Supplementary file 3 — Peer Review File [file 41594_2026_1812_MOESM3_ESM.pdf]

nature portfolio

Peer Review File

Distinct repair outcomes from single and convergent replication fork collapse

Corresponding Author: Dr James Dewar

Version 0:

Decision Letter:

22nd May 2025

Dear Dr. Dewar,

Thank you again for submitting your manuscript "Single and convergent collapsed replication forks elicit distinct repair outcomes". I apologise for the delay in responding, which resulted from the difficulty in timely obtaining suitable referee reports. Nevertheless, we now have comments (below) from the 3 reviewers who evaluated your paper. In light of those reports, we remain interested in your study and would like to see your response to the comments of the referees, in the form of a revised manuscript.

You will see that all the experts appreciate the technical robustness of the work and its potential contribution to the field, they nevertheless raise fairly similar and very important concerns that must be convincingly addressed in a revised manuscript. More specifically, multiple reviewers 1) point out the lack of direct validation of the proposed EJ events and request the addition of relevant experiments, 2) note that the plasmids used may be introducing bias in the possible repair outcomes, including the absence of fork restart, 3) request further evidence for the proposed secondary collapse model (e.g. by demonstrating the generation of very long ssDNA stretches or actually reaching a convergin fork) and 4) raise doubts about how translatable these findings would be in organisms with long linear chromosomes (here presenting evidence for similar outcomes in sufficiently long and linear templates could prove to be useful). We editorially note we would expect the response to these concerns to predominantly be in the form of further experiments, both in accordance to the guidelines provided by all reviewers and additional experiments you may deem suitable to lend more evidence to the proposed models, and not simply discussing alternative scenaria or caveating conclusions.

Please be sure to address/respond to all concerns of the referees in full in a point-by-point response and highlight all changes in the revised manuscript text file. We are committed to providing a fair and constructive peer-review process. Do not hesitate to contact us if there are specific requests from the reviewers that you believe are technically impossible or unlikely to yield a meaningful outcome.

We appreciate the requested revisions are extensive. We thus expect to see your revised manuscript within 6 months. If you cannot send it within this time, please let us know. We will be happy to consider your revision as long as nothing similar has been accepted for publication at NSMB or published elsewhere. Should your manuscript be substantially delayed without notifying us in advance and your article is eventually published, the received date would be that of the revised, not the original, version.

As you already know, we put great emphasis on ensuring that the methods and statistics reported in our papers are correct and accurate. As such, if there are any changes that should be reported, please submit an updated version of the Reporting Summary along with your revision.

Reporting Summary:
https://www.nature.com/documents/nr-reporting-summary.pdf

Please note that the form is a dynamic ‘smart pdf’ and must therefore be downloaded and completed in Adobe Reader.

When submitting the revised version of your manuscript, please pay close attention to our
href="https://www.nature.com/nature-portfolio/editorial-policies/image-integrity">Digital Image Integrity Guidelines. and to the following points below:

-- that unprocessed scans are clearly labelled and match the gels and western blots presented in figures.

-- that control panels for gels and western blots are appropriately described as loading on sample processing controls
-- all images in the paper are checked for duplication of panels and for splicing of gel lanes.

EXTENDED DATA FIGURES

When re-submitting your manuscript, please ensure that any supplementary figures and tables that are crucial to the manuscript's conclusions are converted into Extended Data figures and tables to increase visibility of these data. Extended Data figures and tables are online-only (present in the online PDF and full-text HTML versions of the paper), peer-reviewed display items that provide essential background to the article but are not included in the main article due to space constraints. A maximum of ten Extended Data display items (figures and tables) is permitted.

Finally, please ensure that you retain unprocessed data and metadata files after publication, ideally archiving data in perpetuity, as these may be requested during the peer review and production process or after publication if any issues arise.

SOURCE DATA: we urge authors to provide, in tabular form, the data underlying the graphical representations used in figures. This is to further increase transparency in data reporting, as detailed in this editorial (http://www.nature.com/nsmb/journal/v22/n10/full/nsmb.3110.html). Spreadsheets can be submitted in excel format. Only one (1) file per figure is permitted; thus, for multi-paneled figures, the source data for each panel should be clearly labeled in the Excel file; alternately the data can be provided as multiple, clearly labeled sheets in an Excel file. When submitting files, the title field should indicate which figure the source data pertains to. We encourage our authors to provide source data at the revision stage, so that they are part of the peer-review process.

While we encourage the use of color in preparing figures, please note that this will incur a charge to partially defray the cost of printing. Information about color charges can be found at http://www.nature.com/nsmb/authors/submit/index.html#costs

We require deposition of coordinates (and, in the case of crystal structures, structure factors) into the Protein Data Bank with the designation of immediate release upon publication (HPUB). Electron microscopy-derived density maps and coordinate data must be deposited in EMDB and released upon publication. Deposition and immediate release of NMR chemical shift assignments are highly encouraged. Deposition of deep sequencing and microarray data is mandatory, and the datasets must be released prior to or upon publication. To avoid delays in publication, dataset accession numbers must be supplied with the final accepted manuscript and appropriate release dates must be indicated at the galley proof stage. Please find the complete NRG policies on data availability at http://www.nature.com/authors/policies/availability.html.

Nature Structural & Molecular Biology is committed to improving transparency in authorship. As part of our efforts in this direction, we are now requesting that all authors identified as 'corresponding author' on published papers create and link their Open Researcher and Contributor Identifier (ORCID) with their account on the Manuscript Tracking System (MTS), prior to acceptance. This applies to primary research papers only. ORCID helps the scientific community achieve unambiguous attribution of all scholarly contributions. You can create and link your ORCID from the home page of the MTS by clicking on 'Modify my Springer Nature account'. For more information please visit please visit www.springernature.com/orcid.

Please use the link below to submit your revised manuscript and related files:

Link Redacted

Note: This URL links to your confidential home page and associated information about manuscripts you may have submitted, or that you are reviewing for us. If you wish to forward this email to co-authors, please delete the link to your homepage.

We look forward to seeing the revised manuscript and thank you for the opportunity to review your work.

Sincerely,

Dimitris Typas
Senior Editor
Nature Structural & Molecular Biology
ORCID: 0000-0002-8737-1319

Reviewers' Comments:

Reviewer #1 (Remarks to the Author):

This work from Dewar and colleagues investigates the consequences of fork collapse at SSBs in a cell free system and describes how collapsed forks are resolved, depending on the nature of the SSB and on its location with respect to the replication fork. The authors take advantage of previously developed systems (nicks and nCas9 protected by TetR) to induce fork collapse at SSBs on plasmids in Xenopus laevis egg extracts (Vrtis, 2021), with some improvements to increase efficiency of fork collapse. In Vrtis et al., it was already described how the encounter between the replisome and a SSB leads to replisome disassembly by two distinct mechanisms depending on whether the SSB is located on the leading or the

lagging strand. In this previous publication the intermediates of fork collapse were already identified (seDSBs) and a model for their resolution involving D-loop formation was proposed.
In this work, Dewar and colleagues confirm the model and show dependency on RAD51 for D-loop formation and generation of high molecular well-products. They also further address the resolution of SSBs during “convergent collapse” and show that, in this case, SSBs first generate seDSBs, before being converted into deDSBs by fork convergence. They also show that high molecular weight products still form but are not dependent on RAD51. Finally, the authors investigate the contribution of PARP to this system, showing that PARP can contribute to the repair of SSBs flaked by a DNA binding protein.

This is a comprehensive and detailed analysis of the outcomes of fork collapse at SSBs. Although some of the outcomes of fork collapse at SSBs had been anticipated by previous studies, this analysis has been conducted rigorously and systematically.
On the other hand, a few conclusions are unexpected based on the literature. In particular, the complete absence of fork restart after collapse in this system and the claimed dependency of high molecular weight products on end-joining during convergent collapse are somewhat surprising and would need some further elucidation before I can recommend publication of the manuscript in NSMB.

Major points:
1) Replication fork restart mechanisms after collapse have been widely described in cell-based systems and rely on recombination-dependent mechanisms. In the presented SSB replication fork collapse model in egg extracts, fork restart is never observed. Because a LacR-LacO array is in close proximity to the SSB sites, is it possible that protein binding or topological constraints might inhibit fork restart? Could the authors place the LacR-LacO array further away from the SSBs and show that fork restart is still undetectable?
2) In Fig 4. the authors show that generation of scCM is dependent on MRE11 and propose a model by which these circular monomers are the result of extensive resection of the leading template containing the SSBs. This secondary collapse model predicts generation of very long stretches of ssDNA that should be coated by RPA and activate the checkpoint. Could the authors provide evidence of this to strengthen their model of resection past divergent lagging strands? This seems particularly important because MRE11 depletion also blocks formation of well products (D-loops), and it is therefore impossible to exclude that the very small proportion of observed scCM might originate from an alternative mechanism.
3) In Fig. 5 the authors show that during convergent collapse the HM well products are independent of RAD51 (D-loop) and they propose the action of an end-joining mechanisms. Il would be important to probe this hypothesis by showing decrease of well products after inhibition of NHEJ (for example with NU7441, DNA-PKcs inhibitor (Zhu,2016)) and/or MMEJ (for example by depleting pol Theta, (Mann, 2022)).

Minor points:
1) In fig 1F the nature of the “σ*” species should be explained.
2) In Fig 6B lane 16-20 the equilibrium between scCM and nCM is shifted. The authors should better explain why in the text.
3) Line 339 “collapse at collapsed forks” should probably be corrected to “collapse at converging forks”?

Reviewer #2 (Remarks to the Author):

In this study, Conwell and colleagues examine the generation and fate of collapsed replication forks using plasmids and Xenopus egg extracts. The authors investigate collapsed forks on leading or lagging strands generated by the collision of the replisome with “simple” nicks, Cas9 H840A-induced nicks, and abasic sites. The experiments are well designed, and the conclusions are supported by the results. The lack of DNA synthesis following seDSB invasion is a novel finding that expands current models of seDSB repair, a process that remains poorly understood. My main concern is that some of the observed events don not likely reflect physiological conditions, wherein chromatin context and regulated DNA end resection play critical roles. Therefore, I recommend that the authors temper some of their conclusions, particularly when extrapolating findings to cellular contexts. The paper is recommended for acceptance pending revisions addressing the following points:

-While the schematics in Figures S1M and 1E help illustrate the expected restriction digestion products, it remains unclear for this reader how DNA synthesis is measured.
While some descriptions are in the methods, these explanations are difficult to follow for even a specialist. Given that this measurement supports major conclusions of the study, it would be helpful if an example is included in the supplementary materials or clarified otherwise. This would significantly help readers understand the approach.

-Could the authors provide a zoomed-in view of the E-E products (with increased exposure) in Figure S2C to more clearly demonstrate the reduction of E-E products following RAD51 inhibition? This is a bit hard to see.

-Is the egg extract used in these experiments best characterized as a purely S-phase environment, or does it reflect a mixture of multiple cell cycle phases? Recent studies (PMID: 39631395; PMID: 39631396) suggest that non-homologous end joining (NHEJ) is not a predominant mechanism for repairing collapsed forks during S phase under physiological conditions. It would therefore be helpful for the authors to include a brief discussion on how the cell cycle context of the extract might influence the observed repair outcomes. While end joining could still occur in this system, it may be regulated or suppressed in the S-phase in vivo, and acknowledging this would strengthen the interpretation of the data.

-“Importantly, none of these data show evidence for replication restart at a single collapsed fork, despite efficient resolution of

seDSBs."
What is the meaning of efficient resolution of seDSB in this context? Is it D-loop formation? Please clarify.

-What is the σ* in Fig4F that appears in MRE11 deficient cells?

- The proposed "secondary collapse" model is a plausible explanation for the formation of full-length products at leading-strand collapse in the absence of DNA synthesis within this system. However, its applicability to eukaryotic cells is unclear. In vivo, the DNA ends generated at leading-strand breaks would likely require extensive resection to reach a converging replication fork, which may be unrealistic due to chromatin. Reported resection lengths in wild-type mammalian and yeast cells were reported to be ~5 kb or less (PMID: 30673606; PMID: 38900911), likely insufficient to reach the divergent replication fork.
The authors should discuss the relevance and potential limitations of this secondary collapse model in a cellular context and speculate when this could actually happen. Possible scenarios might be a stalled fork in front of a collapsed fork? Could this occur under conditions that cause replication stress?

- According to the lagging-strand collapse model, a secondary collapse does not occur because the template strand of the converging fork remains intact (Fig. 4I), allowing DNA polymerases to re-synthesize the degraded strand. Could this hypothesis be tested directly by inhibiting DNA synthesis - for example, using polymerase inhibitors such as aphidicolin after collapse? This would help determine whether the recovery of full-length products indeed depends on re-synthesis from the converging fork and further support the proposed mechanism.

- "Thus, seDSBs were resolved prior to deDSBs. During convergent collapse, seDSBs were ~2.5 fold lower than for leading and lagging collapse (~20%in Fig. 5D; ~50% in Fig. 1C, Fig. 3C), suggesting rapid conversion to deDSBs by the converging fork. deDSBs were also 3-fold less abundant than seDSBs (~6% in Fig. S5B; ~20% in Fig. 5D) suggesting that deDSBs were more readily resolved than seDSBs"

While it is possible that deDSBs are resolved more quickly than seDSBs, an alternative explanation is that some leading or lagging seDSBs may invade the template strand prior to the arrival of the converging fork and subsequently merge with it without generating a detectable deDSB. This scenario could also account for the higher percentage of detected seDSBs compared to deDSBs - perhaps not all seDSBs are converted into deDSBs if they engage in strand invasion before fork convergence.

-Although the observation that abasic sites generate collapsed forks in this system is interesting, the benefit of including these data sets in the manuscript is not entirely clear - particularly since PARP inhibition did not influence break formation or resolution. I would suggest removing this section which seemed like an unnecessary distraction in a manuscript that is already hard to follow.

Minor

-line 421: The longer duration between initial seDSB formation and fork convergence may have led to extensive degradation of the seDSB, which might preclude end joining.
The wording "extensive degradation" is referring to physiological resection. The way this is written makes it seem pathological. Please re-phrase it (ie. Extensive resection of the seDSB).

-line 87: "We observed similar outcomes at SSBs generated by Cas9 nickases."
The authors only investigated Cas9 nickase H840A, this should be pointed here instead of nickases.
-It would be helpful to have the origin pointed in the schematics, so the reader can better visualize bidirectional fork progression until it reaches the TetR site.
- please add nickase Cas9 H840A in the model in figure 8.

Reviewer #3 (Remarks to the Author):

This m/s uses an established system in frog egg extracts to study repair and replication consequences of replication fork collision with a leading or lagging ssDNA break (SSB). The principal finding is that the systems examined do not reveal evidence of recombination-mediated resumption of DNA synthesis at the site of the nick. Instead, seDSBs induced by either leading or lagging strand fork breakage (in this m/s termed "collapse") were shown to generate high MW products that were Rad51-dependent and these products appeared as sigma structures disappeared. Thus, Rad51-mediated repair of seDSBs involving a neighboring DNA molecule was a major repair product in this system. High MW products were resolved by RuvC, suggesting that they contain joint molecules/recombination intermediates. The inference that the HR structures generated are D-loops is plausible but not proven. In addition, end-to-end fusions can also form, by mechanisms that were not investigated in this study. Leading strand seDSBs differ from lagging seDSBs by the capacity of MRE11-mediated DNA end resection to effectively remove the free DNA end of the sigma structure, leading to formation of a product that resembles the original plasmid. This is explained by the resection process proceeding around the circular plasmid substrate, causing breakage/collapse of the opposing LacR-blocked fork. The same does not happen for lagging strand seDSBs, because of the continuity of the non-resected strand of the sigma structure (the parental lagging strand of the nick; the parental leading

strand of the opposing fork). This ‘secondary fork collapse’ occurs because the substrate is a small circular plasmid. A Cas9 nickase system generated similar results to those described above and addition of a PARP inhibitor accentuated the potency of a leading strand nick.

Why HR/BIR-mediated replication restart is not observed in this system is unclear. However, the experimental system generates numerous synchronous seDSBs from plasmids in close proximity, presenting an opportunity for repair by either HR or end joining with a neighboring molecule (as shown in the data), which might swamp any potential BIR response. The question of when/how BIR is initiated at a nick-collapsed fork could therefore relate to the abundance of competing substrates for repair. Alternatively, BIR in yeast typically arises after some delay in the processing of a DSB (PMID: 27075725). This delay is abolished in mph1 and sgs1 mutants, suggesting that BIR is actively suppressed by these genes in wild type cells. Perhaps BIR-type restart in the authors’ system is a last resort that is never activated, because of the host of competing alternative repair pathways available (HR- or end joining-mediated).

The data is clear and carefully presented, which is a strength of the m/s. Some weaknesses include the frog egg extract plasmid system itself. This experimental tool floods the system with near-synchronous seDSBs, which might limit the ability to study perhaps rare, late outcomes of fork collapse (potentially including fork restart processes). The use of a small circular plasmid revealed the ‘secondary fork collapse’ phenomenon, but the significance of this effect on a large linear vertebrate chromosome is unclear. The end joining mechanisms are not explored. Some of the text is very dense and some terms are puzzling, making the m/s sometimes difficult to follow. One term that this reviewer had difficulty with is ‘divergent’ fork (when it is in fact the convergent fork at the nick site, but accessed via resection around the entirety of the small circular plasmid).

Specific points

1. Fig 1b why do the sigma structures migrate more slowly over the time-course? Is this because of DNA end resection?

2. Fig 2a The formation of D-loops is inferred but not demonstrated. It might be better to use the term “joint molecules”, since Holliday junctions have not been excluded experimentally.

3. How does depletion of FANCM or BLM (MPH1 and SGS1 homologs) affect the pattern of recombination products? (Does their depletion reveal a fraction of restart events at the nick-collapsed fork?)

4. As noted above, the terms “divergent fork” and “Diverging lagging strands” were confusing.

5. Fig 5i BRC4 peptide doesn’t seem to inhibit very effectively. The authors interpret this effect as meaning that there is a limited requirement for HR at convergent collapsed forks. Alternatively, Rad51 loading might be more efficient in this setting than at a seDSB, exposing the limited ability of the BRC4 peptide to completely block Rad51 loading.

6. What model do the authors propose for the conversion of seDSBs to end-to-end fusions by RAD51?

7. Some typos/word choices make the reading more difficult e.g.: lines 339-340: “Nonetheless, these results support the conclusion that collapse at collapsed forks efficiently complete DNA synthesis while single collapsed forks do not, as for simple SSBs.” I couldn’t work out what this sentence means.

Version 1:

Decision Letter:

Our ref: NSMB-A50810A

26th Mar 2026

Dear Dr. Dewar,

Thank you for submitting your revised manuscript "Distinct repair outcomes from single and convergent replication fork collapse" (NSMB-A50810A). It has now been seen by the original referees and their comments are below. While Reviewers #1 and #2 support the publication of the manuscript, Reviewer #3 retains some of the original concerns, most notably on the potential physiological relevance of the findings in mammalian chromosomes. While we appreciate the systems used here could potentially overrpresent certain events, we editorially deem that it is very likelt that similar events will occur at linear chromosomes, though potentially at lower rates. As importantly as our editorial interpretation, when crossconsulting with Reviewer #2 on whether they agree with this editorial assessment they agreed provided that relevant textual caveats are provided. In that light, and with the support of these two Reviewers, we can now accept the manuscript in principle in Nature Structural & Molecular Biology, pending revisions to tone down and provide caveats where editorially requested to assuage concerns such as those of Reviewer #3, and to comply with our editorial and formatting guidelines.

We are now performing detailed checks on your paper and will send you a checklist detailing our editorial and formatting requirements in about a week. Please do not upload the final materials and make any revisions until you receive this additional information from us.

To facilitate our work at this stage, it is important that we have a copy of the main text as a word file. If you could please send

along a word version of this file as soon as possible, we would greatly appreciate it; please make sure to copy the NSMB account (cc'ed above).

Thank you again for your interest in Nature Structural & Molecular Biology Please do not hesitate to contact me if you have any questions.

Sincerely,

Dimitris Typas
Senior Editor
Nature Structural & Molecular Biology
ORCID: 0000-0002-8737-1319

Reviewer #1 (Remarks to the Author):

The revised manuscript by Dewar and co-workers thoroughly addresses the major and minor concerns raised during the first review. I therefore reccommend publication of this revised manuscript.

Reviewer #2 (Remarks to the Author):

The authors have thoroughly addressed the reviewers' comments, and I support its publication.

Reviewer #3 (Remarks to the Author):

The resubmission has clarified several aspects of the work. Restart is not detected even following depletion of FANCM or BLM. The end joining mechanisms remain undefined. The key problem of the physiological significance of the findings remains unresolved. It is not clear to this reviewer that a 10-fold dilution of the reactants would bring the system towards a physiological context, especially when it is compared with the status of a solitary broken fork on a mammalian chromosome. The physiological significance of secondary collapse of the sister fork remains a matter of speculation.

Version 2:

Decision Letter:

24th Apr 2026

Dear Dr. Dewar,

We are now happy to accept your revised paper "Distinct repair outcomes from single and convergent replication fork collapse" for publication as an Article in Nature Structural & Molecular Biology.

Acceptance is conditional on the manuscript's not being published elsewhere and on there being no announcement of this work to the newspapers, magazines, radio or television until the publication date in Nature Structural & Molecular Biology.

Over the next few weeks, your paper will be copyedited to ensure that it conforms to Nature Structural & Molecular Biology style. Once your paper is typeset, you will receive an email with a link to choose the appropriate publishing options for your paper and our Author Services team will be in touch regarding any additional information that may be required.

After the grant of rights is completed, you will receive a link to your electronic proof via email with a request to make any corrections within 48 hours. If, when you receive your proof, you cannot meet this deadline, please inform us at rjsproduction@springernature.com immediately.

You will not receive your proofs until the publishing agreement has been received through our system.

Due to the importance of these deadlines, we ask that you please let us know now whether you will be difficult to contact over the next month. If this is the case, we ask you provide us with the contact information (email, phone and fax) of someone who will be able to check the proofs on your behalf, and who will be available to address any last-minute problems.

To assist our authors in disseminating their research to the broader community, our SharedIt initiative provides all co-authors with the ability to generate a unique shareable link that will allow anyone (with or without a subscription) to read the published article. Recipients of the link with a subscription will also be able to download and print the PDF.

As soon as your article is published, you can generate your shareable link by entering the DOI of your article here: http://authors.springernature.com/share. Corresponding authors will also receive an automated email with the shareable link

Note the policy of the journal on data deposition: http://www.nature.com/authors/policies/availability.html.

Your paper will be published online soon after we receive proof corrections and will appear in print in the next available issue. You can find out your date of online publication by contacting the production team shortly after sending your proof corrections.

You may wish to make your media relations office aware of your accepted publication, in case they consider it appropriate to organize some internal or external publicity. Once your paper has been scheduled you will receive an email confirming the publication details. This is normally 3-4 working days in advance of publication. If you need additional notice of the date and time of publication, please let the production team know when you receive the proof of your article to ensure there is sufficient time to coordinate. Further information on our embargo policies can be found here: https://www.nature.com/authors/policies/embargo.html

You can now use a single sign-on for all your accounts, view the status of all your manuscript submissions and reviews, access usage statistics for your published articles and download a record of your refereeing activity for the Nature journals.

If you have not already done so, we strongly recommend that you upload the step-by-step protocols used in this manuscript to the Protocol Exchange. Protocol Exchange is an open online resource that allows researchers to share their detailed experimental know-how. All uploaded protocols are made freely available, assigned DOIs for ease of citation and fully searchable through nature.com. Protocols can be linked to any publications in which they are used and will be linked to from your article. You can also establish a dedicated page to collect all your lab Protocols. By uploading your Protocols to Protocol Exchange, you are enabling researchers to more readily reproduce or adapt the methodology you use, as well as increasing the visibility of your protocols and papers. Upload your Protocols at www.nature.com/protocolexchange/. Further information can be found at www.nature.com/protocolexchange/about.

An online order form for reprints of your paper is available at https://www.nature.com/reprints/author-reprints.html. Please let your coauthors and your institutions' public affairs office know that they are also welcome to order reprints by this method.

Authors may need to take specific actions to achieve compliance with funder and institutional open access mandates. If your research is supported by a funder that requires immediate open access (e.g. according to Plan S principles or the NIH public access policy) then you should select the gold OA route, and we will direct you to the compliant route where possible. Because authors warrant under our subscription licensing terms that they haven't committed to licensing any version of their article under a licence inconsistent with the terms of our agreement – including the applicable embargo period – publication under the subscription model isn't suitable for authors whose funders require no embargo.

In approximately 10 business days you will receive an email with a link to choose the appropriate publishing options for your paper and our Author Services team will be in touch regarding any additional information that may be required.

You will not receive your proofs until the publishing agreement has been received through our system.

If you have any questions about our publishing options, costs, Open Access requirements, or our legal forms, please contact ASJournals@springernature.com

Sincerely,

Dimitris Typas
Senior Editor
Nature Structural & Molecular Biology
ORCID: 0000-0002-8737-1319

Click here if you would like to recommend Nature Structural & Molecular Biology to your librarian: http://www.nature.com/subscriptions/recommend.html#forms

Open Access This Peer Review File is licensed under a Creative Commons Attribution 4.0 International License, which permits use, sharing, adaptation, distribution and reproduction in any medium or format, as long as you give appropriate credit to the original author(s) and the source, provide a link to the Creative Commons license, and indicate if changes were made.
In cases where reviewers are anonymous, credit should be given to 'Anonymous Referee' and the source.
The images or other third party material in this Peer Review File are included in the article's Creative Commons license, unless indicated otherwise in a credit line to the material. If material is not included in the article's Creative Commons license and your intended use is not permitted by statutory regulation or exceeds the permitted use, you will need to obtain permission directly from the copyright holder.
To view a copy of this license, visit https://creativecommons.org/licenses/by/4.0/

We thank the editor and reviewers for their detailed and constructive feedback on our manuscript. We are pleased that all three reviewers found our systematic analysis of fork collapse outcomes to be of considerable interest, and we are grateful for the many suggestions that have strengthened the work.

(1) All three reviewers raised questions about the secondary collapse model, including the mechanism, its dependence on resection, and its relevance to chromosomal contexts (responses #2, #13, #14, #22). We have performed two new experiments that provide additional support for this model. Addition of aphidicolin following collapse caused a substantial increase in nicked circular monomers (nCMs), consistent with DNA polymerase activity guarding against resection (new Extended Data Fig. 5F-I; response #2). nCMs were essentially eliminated when the sister fork was positioned further away (new Extended Data Fig. 2I-R; responses #2, #13), supporting the conclusion that secondary collapse arises from resection reaching the sister fork. We have also expanded the Discussion to address when secondary collapse could occur in a chromosomal context, including scenarios involving impaired homologous recombination (HR), stalled forks, or lagging collapse (responses #13, #22).

(2) All three reviewers requested characterization of the end-joining mechanism operating at convergent collapsed forks (responses #3, #10, #23, #29, #30). We have performed extensive new experiments establishing that convergent and single fork collapse produce distinct repair outcomes. Convergent collapse repair is independent of canonical NHEJ and MMEJ: immunodepletion of DNA-PKcs or POLQ had no effect on repair; small-molecule inhibitors of both pathways produced effects that persisted after immunodepletion, indicating off-target activity (Reviewer Figs. R2-R4; response #3). RAD52 activity was also dispensable (Reviewer Figs. R5-R6). In contrast, repair required resection activity, as immunodepletion of either MRE11 or CtIP inhibited the process (new Figs. 6A-H, 6I-P; response #3). Direct sequencing of repair products revealed that approximately half of sequenced products contained replication-dependent precise deletions within the tetO array, consistent with annealing-dependent DSB repair (new Fig. 7; response #3). To our knowledge, precise deletions arising from annealing-dependent DSB repair have not previously been observed following fork collapse. These error-prone products were not detected following single fork collapse, consistent with distinct repair outcomes at convergent versus single collapsed forks. Together, these data indicate that annealing-dependent DSB repair produces deletions specifically following convergent collapse, raising the possibility that this pathway could be harnessed for programmed deletions in a chromosomal context (lines 370-372).

(3) Reviewers 1 and 3 questioned the absence of replication restart following single fork collapse (responses #1, #21). We have performed two new control experiments. Moving the single-strand break (SSB) further from the lacO array revealed a low level of detectable repair that was negligible, consistent with a minority of forks converging on the nick rather than restart (new Extended Data Fig. 2I-R; response #1). Supplementation with a 10-fold excess of nucleoplasm did not promote detectable restart (Reviewer Fig. R7; response #21), arguing against seDSB density as a sufficient explanation for absent restart. Finally, we calculated the upper bound of restart that would be beyond the resolution of our experiments as ~10% (lines 327-330), consistent with stable D-loops being the dominant outcome.

(4) Reviewer 2 raised the possibility that seDSBs could be resolved prior to fork convergence during convergent collapse (response #15). We performed a fine time course of convergent collapse in the presence of BRC peptide (new Extended Data Fig. 6G-K) and found no detectable alteration of seDSB kinetics, arguing against HR-mediated resolution of seDSBs prior to fork convergence. MRE11 depletion also did not alter seDSB resolution (Fig. 6B-C). The ~1:1 ratio of linears to circular monomers under MRE11-depleted conditions (Extended Data Fig. 9C) is consistent with most breaks being converted to double-ended DSBs (deDSBs).

(5) Reviewers 2 and 3 raised concerns about the clarity of the writing (responses #16 and #24). In response we have removed the previous abasic site data to focus the manuscript (as suggested by reviewer 2, response #16). We have also endeavored to simplify the text as much as possible.

In total, we performed 18 new experiments addressing the reviewers' concerns, of which 9 are incorporated into the revised manuscript and 9 are provided as Reviewer Figures. We have also thoroughly re-written and simplified the entire manuscript. We believe the manuscript is substantially improved and is now suitable for publication.

Reviewer #1 (Remarks to the Author):

This work from Dewar and colleagues investigates the consequences of fork collapse at SSBs in a cell free system and describes how collapsed forks are resolved, depending on the nature of the SSB and on its location with respect to the replication fork. The authors take advantage of previously developed systems (nicks and nCas9 protected by TetR) to induce fork collapse at SSBs on plasmids in Xenopus laevis egg extracts (Vrtis, 2021), with some improvements to increase efficiency of fork collapse. In Vrtis et al., it was already described how the encounter between the replisome and a SSB leads to replisome disassembly by two distinct mechanisms depending on whether the SSB is located on the leading or the lagging strand. In this previous publication the intermediates of fork collapse were already identified (seDSBs) and a model for their resolution involving D-loop formation was proposed.
In this work, Dewar and colleagues confirm the model and show dependency on RAD51 for D-loop formation and generation of high molecular well-products. They also further address the resolution of SSBs during "convergent collapse" and show that, in this case, SSBs first generate seDSBs, before being converted into deDSBs by fork convergence. They also show that high molecular weight products still form but are not dependent on RAD51. Finally, the authors investigate the contribution of PARP to this system, showing that PARP can contribute to the repair of SSBs flaked by a DNA binding protein.

This is a comprehensive and detailed analysis of the outcomes of fork collapse at SSBs. Although some of the outcomes of fork collapse at SSBs had been anticipated by previous studies, this analysis has been conducted rigorously and systematically.
On the other hand, a few conclusions are unexpected based on the literature. In particular, the complete absence of fork restart after collapse in this system and the claimed dependency of high molecular weight products on end-joining during convergent collapse are somewhat surprising and would need some further elucidation before I can recommend publication of the manuscript in NSMB.

Major points:
1) Replication fork restart mechanisms after collapse have been widely described in cell-based systems and rely on recombination-dependent mechanisms. In the presented SSB replication fork collapse model in egg extracts, fork restart is never observed. Because a LacR-LacO array is in close proximity to the SSB sites, is it possible that protein binding or topological constraints might inhibit fork restart? Could the authors place the LacR-LacO array further away from the SSBs and show that fork restart is still undetectable?

#1 We thank the reviewer for raising this important point. We have now performed this experiment by moving the SSB further from the lacO array (new Extended Data Fig. 2I-R). Repair was detectable but negligible, consistent with a minority of forks converging on the nick rather than restart at the seDSB (lines 98-102). These data are consistent with our conclusion that we detect no evidence of replication restart at single collapsed forks.

2) In Fig 4. the authors show that generation of scCM is dependent on MRE11 and propose a model by which these circular monomers are the result of extensive resection of the leading template containing the SSBs. This secondary collapse model predicts generation of very long stretches of ssDNA that should be coated by RPA and activate the checkpoint. Could the authors provide evidence of this to strengthen their model of resection past divergent lagging strands? This seems particularly important because MRE11 depletion also blocks formation of well products (D-loops), and it is therefore impossible to exclude that the very small proportion of observed scCM might originate from an alternative mechanism.

#2 We thank the reviewer for this thoughtful suggestion. We apologize that the original manuscript did not clearly describe the distinct effects of RAD51 and MRE11 on these products. RAD51 inhibition blocked D-loop formation but increased circular monomer (CM) production (Fig. 2B; Extended Data Fig. 3B), whereas MRE11 depletion abolished both D-loops and CMs (Fig. 3E-H). We have clarified this distinction in the revised manuscript (lines 122-124, 163-166).

We performed the Chk1 analysis as the reviewer suggested. Chk1 phosphorylation was not substantially altered following collapse (Reviewer Fig. R1). This result neither supports nor contradicts

the model, because secondary collapse is a rare event that may not generate sufficient single-stranded DNA (ssDNA) to substantially alter Chk1 phosphorylation levels.

To test our hypothesis that DNA polymerase activity guards against resection, we added aphidicolin following collapse. This treatment caused a substantial increase in nCMs (Extended Data Fig. 5F-I), consistent with DNA polymerase activity guarding against resection. We also performed experiments using a larger backbone plasmid to move the sister fork further away. nCMs were essentially eliminated when the sister fork was positioned further away (new Fig. 3I-K), strongly supporting the conclusion that secondary collapse arises from resection reaching the sister fork. Together, these data provide additional support for the secondary collapse model.

3) In Fig. 5 the authors show that during convergent collapse the HM well products are independent of RAD51 (D-loop) and they propose the action of an end-joining mechanisms. Il would be important to probe this hypothesis by showing decrease of well products after inhibition of NHEJ (for example with NU7441, DNA-PKcs inhibitor (Zhu,2016)) and/or MMEJ (for example by depleting pol Theta, (Mann, 2022)).

#3 We thank the reviewer for this excellent suggestion. The experiments prompted by this comment led to a more complete characterization of the repair mechanism. We tested the roles of DNA-PKcs and POLQ as suggested, using both immunodepletion and the suggested small-molecule inhibitors (Reviewer Figs. R2-R4). Immunodepletion of either DNA-PKcs or POLQ had no effect on repair. Small-molecule inhibitors of both pathways produced effects that persisted after immunodepletion, indicating off-target activity (Reviewer Figs. R2-R4). We also tested whether RAD52 activity was required but found that it was not necessary following collapse at either tetO-protected nicks or nCas9 induced nicks (Reviewer Figs. R5-6). Thus, in this system, repair at convergent collapsed forks is independent of RAD51, RAD52, DNA-PKcs, and POLQ.

We next asked whether the mechanism required resection activity. Repair was dependent on resection, as immunodepletion of either MRE11 (new Fig. 6A-H) or CtIP (new Fig. 6I-P) inhibited the process. To characterize the repair products directly, we performed sequencing of the repair products (new Fig. 7). Sequencing revealed precise deletions within the tetO array (new Fig. 7B-C). The replication-dependent fraction of deletions was ~50% of total reads, corresponding to essentially all repair products, consistent with an efficient annealing-dependent DSB repair mechanism (new Fig. 7B-C). These deletion products were dependent on replication (new Fig. 7B-C) and diminished by MRE11 depletion (new Fig. 7H-I), as expected for products of repair. These products were absent from leading collapse (Fig. 7L-M), consistent with a distinct repair outcome at convergent versus single collapsed forks.

Together, these data indicate that annealing-dependent DSB repair produces deletions specifically following convergent collapse.

Minor points:
1) In fig 1F the nature of the “σ*” species should be explained.

#4 We now explain in the text that this is a topoisomer of the σ species (lines 478-479).

2) In Fig 6B lane 16-20 the equilibrium between scCM and nCM is shifted. The authors should better explain why in the text.

#5 We now explain in the text that persistent nicking by nCas9 prevents the compensatory supercoiling that otherwise accompanies nucleosome removal during deproteinization, which shifts the equilibrium toward nicked species (lines 230-232).

3) Line 339 “collapse at collapsed forks” should probably be corrected to “collapse at converging forks”?

#6 We thank the reviewer for identifying this typo. This is now corrected.

Reviewer #2 (Remarks to the Author):

In this study, Conwell and colleagues examine the generation and fate of collapsed replication forks using plasmids and Xenopus egg extracts. The authors investigate collapsed forks on leading or lagging strands generated by the collision of the replisome with "simple" nicks, Cas9 H840A-induced nicks, and abasic sites. The experiments are well designed, and the conclusions are supported by the results. The lack of DNA synthesis following seDSB invasion is a novel finding that expands current models of seDSB repair, a process that remains poorly understood. My main concern is that some of the observed events don not likely reflect physiological conditions, wherein chromatin context and regulated DNA end resection play critical roles. Therefore, I recommend that the authors temper some of their conclusions, particularly when extrapolating findings to cellular contexts. The paper is recommended for acceptance pending revisions addressing the following points:

#7 We thank the reviewer for their supportive appraisal of the manuscript. In the revised manuscript, we more carefully acknowledge potential differences between our in vitro system and cellular contexts. We now use "semi-synchronous" throughout to describe convergent collapse in our system, note that our extracts are proficient for HR, and discuss synchrony as a potential explanation for the predominance of HR-independent repair at convergent collapsed forks (lines 337-353).

-While the schematics in Figures S1M and 1E help illustrate the expected restriction digestion products, it remains unclear for this reader how DNA synthesis is measured.
While some descriptions are in the methods, these explanations are difficult to follow for even a specialist. Given that this measurement supports major conclusions of the study, it would be helpful if an example is included in the supplementary materials or clarified otherwise. This would significantly help readers understand the approach.

#8 We thank the reviewer for bringing this to our attention. In the revised manuscript we have included a table illustrating how the experimentally measured values are affected by different efficiencies of collapse and repair, which are the major variables influencing the underlying data. We also include a worked example in Supplementary Figure 2.

-Could the authors provide a zoomed-in view of the E-E products (with increased exposure) in Figure S2C to more clearly demonstrate the reduction of E-E products following RAD51 inhibition? This is a bit hard to see.

#9 We have now added a zoomed-in view with increased exposure (Extended Data Fig. 3C) and also included quantification of these species for additional clarity (Extended Data Fig. 3E).

-Is the egg extract used in these experiments best characterized as a purely S-phase environment, or does it reflect a mixture of multiple cell cycle phases? Recent studies (PMID: 39631395; PMID: 39631396) suggest that non-homologous end joining (NHEJ) is not a predominant mechanism for repairing collapsed forks during S phase under physiological conditions. It would therefore be helpful for the authors to include a brief discussion on how the cell cycle context of the extract might influence the observed repair outcomes. While end joining could still occur in this system, it may be regulated or suppressed in the S-phase in vivo, and acknowledging this would strengthen the interpretation of the data.

#10 We thank the reviewer for raising this important point. The extracts we use are derived from replicating nuclei and contain high CDK and DDK activity, mimicking S phase of the early embryonic cell cycle. We cannot formally define these as "S phase" because there is only interphase and mitosis during early Xenopus development, but these extracts are proficient for HR as demonstrated by robust RAD51-dependent repair at single collapsed forks (Figs. 1-2). Less concentrated versions of these extracts (G1-like extracts) favor NHEJ (PMID 26990988), and supplementation with mitotic CDK activates different repair pathways (PMID 30849395), indicating that cell cycle context modulates

pathway choice in this system. In the revised manuscript, we now discuss these points and note that end joining can act throughout S phase in human cells (PMID 22841003; lines 351-352). Our favored explanation for the HR independence of convergent collapse repair is that the synchrony with which the two DNA ends form favors HR-independent pathways, rather than cell cycle context per se (lines 342-353).

-"Importantly, none of these data show evidence for replication restart at a single collapsed fork, despite efficient resolution of seDSBs."
What is the meaning of efficient resolution of seDSB in this context? Is it D-loop formation? Please clarify.

#11 We apologize that this was not sufficiently clear. In the revised manuscript, we define "DSB resolution" broadly as removal of the free DNA end (line 40), which encompasses both repair events (D-loop formation, end joining) and the degradation events we observe (secondary collapse). In this context, "efficient resolution of seDSBs" refers to the conversion of seDSBs to joint molecules arising from D-loops and other products, without detectable restart of DNA synthesis.

-What is the σ* in Fig4F that appears in MRE11 deficient cells?

#12 These species correspond to topoisomers of σ species, as noted above (response #4) (lines 478-479).

- The proposed "secondary collapse" model is a plausible explanation for the formation of full-length products at leading-strand collapse in the absence of DNA synthesis within this system. However, its applicability to eukaryotic cells is unclear. In vivo, the DNA ends generated at leading-strand breaks would likely require extensive resection to reach a converging replication fork, which may be unrealistic due to chromatin. Reported resection lengths in wild-type mammalian and yeast cells were reported to be ~5 kb or less (PMID: 30673606; PMID: 38900911), likely insufficient to reach the divergent replication fork.
The authors should discuss the relevance and potential limitations of this secondary collapse model in a cellular context and speculate when this could actually happen. Possible scenarios might be a stalled fork in front of a collapsed fork? Could this occur under conditions that cause replication stress?

#13 We thank the reviewer for this thoughtful suggestion. In the revised manuscript, we directly tested the prediction that extensive resection is required for secondary collapse. Secondary collapse was essentially eliminated when the sister fork was positioned further away (new Fig. 3I-K), consistent with a requirement for extensive resection. Our favored model is that secondary collapse could occur when resolution of the broken end is impaired, for example due to deficient HR activity, given that RAD51 inhibition increases circular monomer production (Fig. 2B) and increased resection is observed in cells under these same conditions (Fig. 3 of PMID 30673606; and Fig. 2 of PMID 38900911). We appreciate the reviewer's suggestion that an adjacent stalled fork could also facilitate secondary collapse and have incorporated this scenario. We also note that secondary collapse could influence whether lagging collapse generates single- or double-ended DSBs. We discuss these models in the revised Discussion (lines 384-396) and incorporate them into our revised model (Fig. 8D-E).

- According to the lagging-strand collapse model, a secondary collapse does not occur because the template strand of the converging fork remains intact (Fig. 4I), allowing DNA polymerases to re-synthesize the degraded strand. Could this hypothesis be tested directly by inhibiting DNA synthesis - for example, using polymerase inhibitors such as aphidicolin after collapse? This would help determine whether the recovery of full-length products indeed depends on re-synthesis from the converging fork and further support the proposed mechanism.

#14 The reviewer raises an excellent point. We have clarified the mechanistic basis for this asymmetry in the revised Discussion. Secondary collapse is specific to leading collapse because for lagging

collapse, 5'-3' resection targets the nascent rather than parental strands (lines 387-388). Our model predicts that aphidicolin would enhance secondary collapse following leading collapse, where 5'-3' resection targets the parental DNA strand. This is indeed the case (Extended Data Fig. 5F-I), consistent with DNA polymerase activity guarding against resection.

- “Thus, seDSBs were resolved prior to deDSBs. During convergent collapse, seDSBs were ~2.5 fold lower than for leading and lagging collapse (~20%in Fig. 5D; ~50% in Fig. 1C, Fig. 3C), suggesting rapid conversion to deDSBs by the converging fork. deDSBs were also 3-fold less abundant than seDSBs (~6% in Fig. S5B; ~20% in Fig. 5D) suggesting that deDSBs were more readily resolved than seDSBs”

While it is possible that deDSBs are resolved more quickly than seDSBs, an alternative explanation is that some leading or lagging seDSBs may invade the template strand prior to the arrival of the converging fork and subsequently merge with it without generating a detectable deDSB. This scenario could also account for the higher percentage of detected seDSBs compared to deDSBs - perhaps not all seDSBs are converted into deDSBs if they engage in strand invasion before fork convergence.

#15 We thank the reviewer for raising this alternative interpretation. We agree that the original data did not rule out this possibility. In the revised manuscript, we provide new experiments to address this concern. We performed a fine time course of convergent collapse in the presence of BRC peptide to block resolution of seDSBs (new Extended Data Fig. 6G-K). BRC peptide did not detectably alter seDSB kinetics during convergent collapse, arguing against rapid HR-mediated resolution of seDSBs prior to fork convergence. MRE11 depletion also did not alter seDSB resolution (new Fig. 6B-C). The ~1:1 ratio of linears to circular monomers under MRE11-depleted conditions (new Extended Data Fig. 9C) is consistent with most breaks being converted to deDSBs rather than being resolved as seDSBs. Together, these data argue against seDSB resolution prior to fork convergence.

-Although the observation that abasic sites generate collapsed forks in this system is interesting, the benefit of including these data sets in the manuscript is not entirely clear - particularly since PARP inhibition did not influence break formation or resolution. I would suggest removing this section which seemed like an unnecessary distraction in a manuscript that is already hard to follow.

#16 We appreciate the reviewer's comment. Given the substantial data we have added with the revision, we have now removed this figure from the manuscript.

Minor

-line 421: The longer duration between initial seDSB formation and fork convergence may have led to extensive degradation of the seDSB, which might preclude end joining.
The wording “extensive degradation” is referring to physiological resection. The way this is written makes it seem pathological. Please re-phrase it (ie. Extensive resection of the seDSB).

#17 We now use "extensive resection" in place of "extensive degradation" throughout the revised manuscript.

-line 87: “We observed similar outcomes at SSBs generated by Cas9 nickases.”
The authors only investigated Cas9 nickase H840A, this should be pointed here instead of nickases.

#18 We have updated this to specify Cas9 nickase H840A (line 67).

-It would be helpful to have the origin pointed in the schematics, so the reader can better visualize bidirectional fork progression until it reaches the TetR site.

#19 We thank the reviewer for this suggestion. In this system, replication initiation is distributed throughout the plasmid rather than occurring at a defined site, so a single origin cannot be marked in the schematics. We now state this explicitly in the Fig. 1A legend: "Plasmids harbor multiple sequence non-specific origins distributed throughout the backbone" (lines 408-410). We have updated the schematics to indicate distributed origin firing rather than a single origin.

- please add nickase Cas9 H840A in the model in figure 8.

#20 We have removed the initial panel from the model figure because the abasic site figure (previously Fig. 7) was removed from the manuscript (see response #16). The revised model figure no longer explicitly references Cas9 nickase.

Reviewer #3 (Remarks to the Author):

This m/s uses an established system in frog egg extracts to study repair and replication consequences of replication fork collision with a leading or lagging ssDNA break (SSB). The principal finding is that the systems examined do not reveal evidence of recombination-mediated resumption of DNA synthesis at the site of the nick. Instead, seDSBs induced by either leading or lagging strand fork breakage (in this m/s termed "collapse") were shown to generate high MW products that were Rad51-dependent and these products appeared as sigma structures disappeared. Thus, Rad51-mediated repair of seDSBs involving a neighboring DNA molecule was a major repair product in this system. High MW products were resolved by RuvC, suggesting that they contain joint molecules/recombination intermediates. The inference that the HR structures generated are D-loops is plausible but not proven. In addition, end-to-end fusions can also form, by mechanisms that were not investigated in this study. Leading strand seDSBs differ from lagging seDSBs by the capacity of MRE11-mediated DNA end resection to effectively remove the free DNA end of the sigma structure, leading to formation of a product that resembles the original plasmid. This is explained by the resection process proceeding around the circular plasmid substrate, causing breakage/collapse of the opposing LacR-blocked fork. The same does not happen for lagging strand seDSBs, because of the continuity of the non-resected strand of the sigma structure (the parental lagging strand of the nick; the parental leading strand of the opposing fork). This 'secondary fork collapse' occurs because the substrate is a small circular plasmid. A Cas9 nickase system generated similar results to those described above and addition of a PARP inhibitor accentuated the potency of a leading strand nick.

Why HR/BIR-mediated replication restart is not observed in this system is unclear. However, the experimental system generates numerous synchronous seDSBs from plasmids in close proximity, presenting an opportunity for repair by either HR or end joining with a neighboring molecule (as shown in the data), which might swamp any potential BIR response. The question of when/how BIR is initiated at a nick-collapsed fork could therefore relate to the abundance of competing substrates for repair. Alternatively, BIR in yeast typically arises after some delay in the processing of a DSB (PMID: 27075725). This delay is abolished in mph1 and sgs1 mutants, suggesting that BIR is actively suppressed by these genes in wild type cells. Perhaps BIR-type restart in the authors' system is a last resort that is never activated, because of the host of competing alternative repair pathways available (HR- or end joining-mediated).

The data is clear and carefully presented, which is a strength of the m/s.

We thank the reviewer for this detailed and thoughtful assessment.

Some weaknesses include the frog egg extract plasmid system itself. This experimental tool floods the system with near-synchronous seDSBs, which might limit the ability to study perhaps rare, late outcomes of fork collapse (potentially including fork restart processes).

#21 In the revised manuscript, we address the concern that seDSB density could prevent detection of restart. We induced collapse and then supplemented with a 10-fold excess of nucleoplasm (Reviewer Fig. R7). This dilution reduced the ratio of DSBs per nucleoplasm to levels comparable to experiments in yeast where restart is observed (see legend for Reviewer Fig. R7), arguing against seDSB density as the explanation for absent restart. We also note that increasing seDSB density in human cells does not appear to alter the repair mechanism (PMID 38900911). In the Discussion (lines 330-335), we state that our inability to detect BIR is consistent with evidence that most fork collapse events involve deDSB formation and that BIR proteins do not appear to be major determinants of PARP inhibitor cytotoxicity, while acknowledging that we cannot rule out unique properties of our in vitro system.

The use of a small circular plasmid revealed the 'secondary fork collapse' phenomenon, but the significance of this effect on a large linear vertebrate chromosome is unclear.

#22 We thank the reviewer for raising the question of secondary collapse relevance in a chromosomal context. In the revised manuscript, we now discuss this point in the Discussion (lines 384-396). Secondary collapse was essentially eliminated when the sister fork was positioned further away (new Fig. 3I-K; see also response #13), consistent with a requirement for extensive resection. We propose

that secondary collapse could operate when HR is deficient or when an adjacent fork stalls after origin firing (Fig. 3, lines 390-392). We also note that secondary collapse could negatively regulate deDSB formation following lagging collapse (Fig. 8E, lines 392-395) and state that "Understanding the cellular contexts in which this mechanism occurs is an important future direction" (lines 395-396).

The end joining mechanisms are not explored.

#23 We thank the reviewer for this comment. In the revised manuscript, we have now extensively characterized the end-joining mechanism at deDSBs arising from convergent collapse. We show that this repair is independent of DNA-PKcs, POLQ, and RAD52, but is dependent on resection by MRE11 and CtIP. Direct sequencing reveals precise deletions consistent with annealing-dependent DSB repair (see response #3 to Reviewer 1 for details). We have also characterized the end-to-end fusions at seDSBs and show that these involve short stretches of microhomology, consistent with alternative end joining (response #30, below).

Some of the text is very dense and some terms are puzzling, making the m/s sometimes difficult to follow. One term that this reviewer had difficulty with is 'divergent' fork (when it is in fact the convergent fork at the nick site, but accessed via resection around the entirety of the small circular plasmid).

#24 We apologize that aspects of the writing were difficult to follow. We have endeavored to improve clarity throughout the revised manuscript, including removal of prior abasic site data (as suggested by Reviewer 2, response #16). We have updated the "divergent fork" terminology to the standard "sister fork" terminology throughout. We also specify in the legend for Figure 1A that a "1.6 kb lacO array bound by LacR blocks converging forks at a distance" (line 406).

Specific points

1. Fig 1b why do the sigma structures migrate more slowly over the time-course? Is this because of DNA end resection?

#25 We thank the reviewer for this question. The slower migration of sigma structures over time is due to resection. As stated in the Fig. 3 legend (lines 480-482): "sigma structures migrated more slowly over time in mock but not MRE11-immunodepleted conditions, indicating the slowed migration was due to resection, presumably because of secondary structures formed by exposed single-stranded DNA."

2. Fig 2a The formation of D-loops is inferred but not demonstrated. It might be better to use the term "joint molecules", since Holliday junctions have not been excluded experimentally.

#26 We agree that we have not formally ruled out Holliday junctions. In the revised manuscript, we refer to these species as "joint molecules arising from D-loops" (first used at lines 129-130) to account for the possibility that they could contain D-loops or Holliday junctions, which would form downstream of D-loops.

3. How does depletion of FANCM or BLM (MPH1 and SGS1 homologs) affect the pattern of recombination products? (Does their depletion reveal a fraction of restart events at the nick-collapsed fork?)

#27 We thank the reviewer for this suggestion. Depletion of either FANCM or BLM increased joint molecule formation (Reviewer Figs. R8-R9), consistent with these species containing D-loops that are negatively regulated by FANCM and BLM. However, we did not observe increased synthesis in the collapsed region and thus did not detect evidence of restart. We did not include these data in the revised manuscript, which is already very dense as the reviewer noted.

4. As noted above, the terms “divergent fork” and “Diverging lagging strands” were confusing.

#28 We have updated the language, as noted in response #24.

5. Fig 5i BRC4 peptide doesn’t seem to inhibit very effectively. The authors interpret this effect as meaning that there is a limited requirement for HR at convergent collapsed forks. Alternatively, Rad51 loading might be more efficient in this setting than at a seDSB, exposing the limited ability of the BRC4 peptide to completely block Rad51 loading.

#29 We thank the reviewer for raising this alternative interpretation. In the revised manuscript, we also immunodepleted RAD51 and observed the same limited effect on repair (new Extended Data Fig. 7A-F). We also note that BRC peptide is highly inhibitory at deDSBs in other contexts in the extract system we use (PMIDs 21719678 and 35641523). These results, together with the deletion sequencing data revealing a high level of precise deletions (responses #3, #23), support the conclusion that, in this setting, convergent collapse repair occurs largely independently of HR.

6. What model do the authors propose for the conversion of seDSBs to end-to-end fusions by RAD51?

#30 Sequencing reveals that these fusions occur at sites of microhomology, suggesting limited resection. We suggest that the dsDNA-binding activity of RAD51 may limit resection and promote these fusions. We now discuss this model in the manuscript (lines 374-382).

7. Some typos/word choices make the reading more difficult e.g.: lines 339-340: “Nonetheless, these results support the conclusion that collapse at collapsed forks efficiently complete DNA synthesis while single collapsed forks do not, as for simple SSBs.” I couldn’t work out what this sentence means.

#31 We have corrected these typos and endeavored to improve clarity throughout the revised manuscript, as noted in response #24.

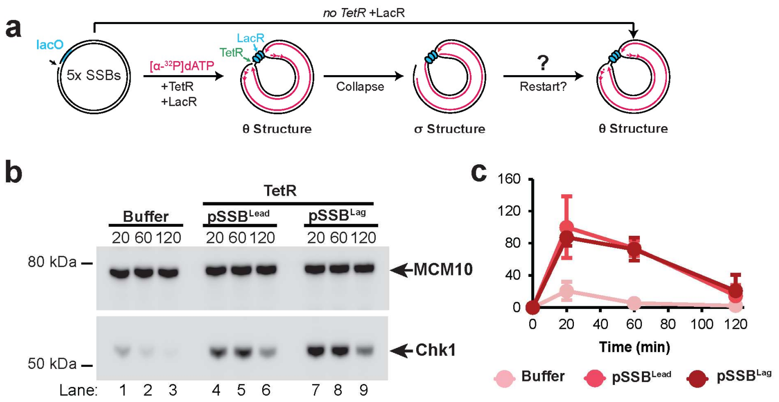

Reviewer Figure R1: Analysis of pCHK1 following leading and lagging collapse

(A) Schematic of the experimental design. $pSSB^{LEAD}$ or $pSSB^{LAG}$ was replicated in Xenopus egg extract in the presence of TetR and LacR to induce leading or lagging strand collapse, respectively. Nascent strands were radiolabeled by inclusion of [α-^{32}P]dATP. TetR was omitted in the buffer control (+Buffer). **(B)** Chromatin samples were collected at the indicated time points (20, 60, 120 min) under buffer, $pSSB^{LEAD}$ (+TetR), and $pSSB^{LAG}$ (+TetR) conditions and analyzed by western blotting for MCM10 (loading control, top) and Chk1 (bottom). Chk1 phosphorylation was assessed by mobility shift. **(C)** Quantification of Chk1 signal from (B) across the indicated time points and conditions. Chk1 phosphorylation was not substantially altered following either leading or lagging collapse relative to the buffer control. This result neither supports nor contradicts the secondary collapse model, because secondary collapse is a rare event that may not generate sufficient single-stranded DNA to substantially alter Chk1 phosphorylation levels. *n* = 3.

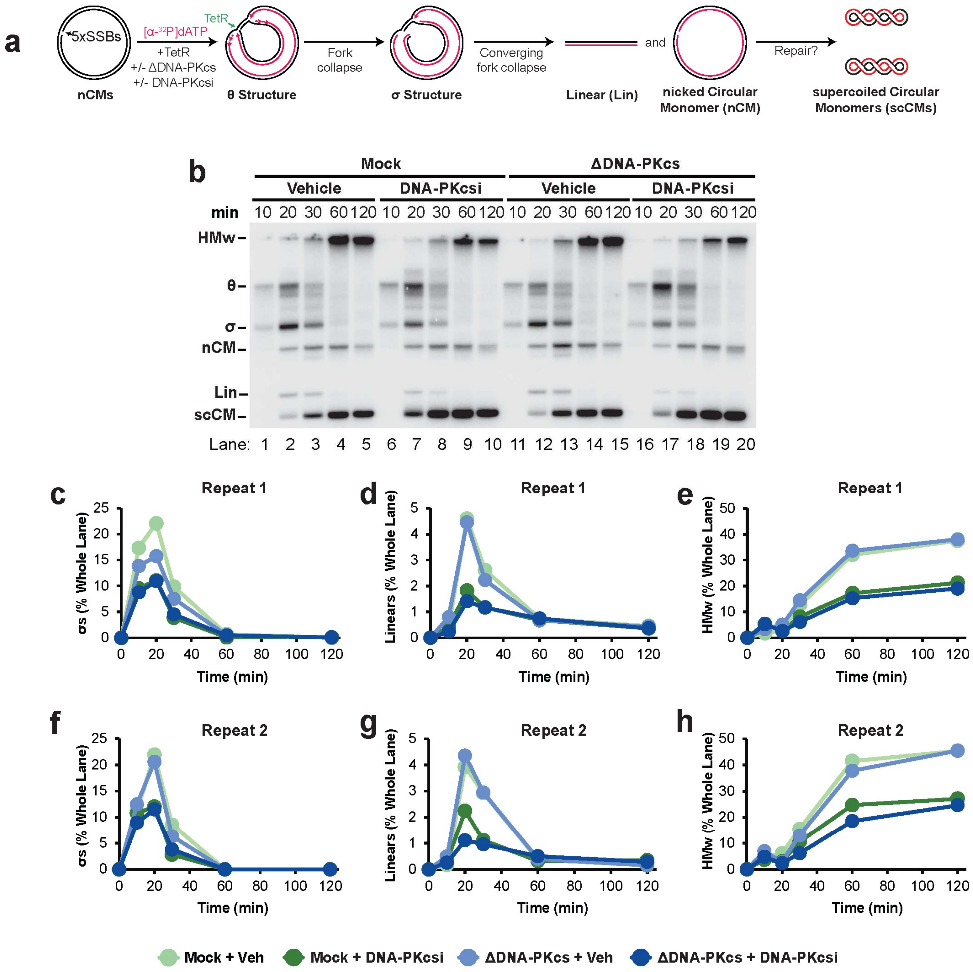

Reviewer Figure R2: Small molecule inhibition, but not depletion, of DNA-PKcs reduces HMw abundance **(A)** Schematic of the experimental design. Convergent collapse was induced by replication of pSSB in mock- or DNA-PKcs-immunodepleted extracts, each supplemented with either vehicle or DNA-PKcs inhibitor (DNA-PKcsi). Nascent strands were radiolabeled by inclusion of [α-^{32}P]dATP. **(B)** Samples from (A) were separated on an agarose gel and visualized by autoradiography. Key species are indicated: high molecular weight products (HMw), θ structures, σ structures, nicked circular monomers (nCM), linear molecules (Lin), and supercoiled circular monomers (scCM). **(C)** Quantification of σ structures from (B), Repeat 1. **(D)** Quantification of linear molecules from (B), Repeat 1. **(E)** Quantification of HMw products from (B), Repeat 1. **(F)** Quantification of σ structures from (B), Repeat 2. **(G)** Quantification of linear molecules from (B), Repeat 2. **(H)** Quantification of HMw products from (B), Repeat 2. DNA-PKcs immunodepletion did not reduce HMw abundance, indicating that DNA-PKcs is dispensable for repair of deDSBs following convergent collapse. DNA-PKcsi reduced HMw in both mock- and DNA-PKcs-immunodepleted extracts, indicating that the inhibitor effect persisted in the absence of DNA-PKcs and was therefore attributable to off-target activity. *n* = 2 independent experiments.

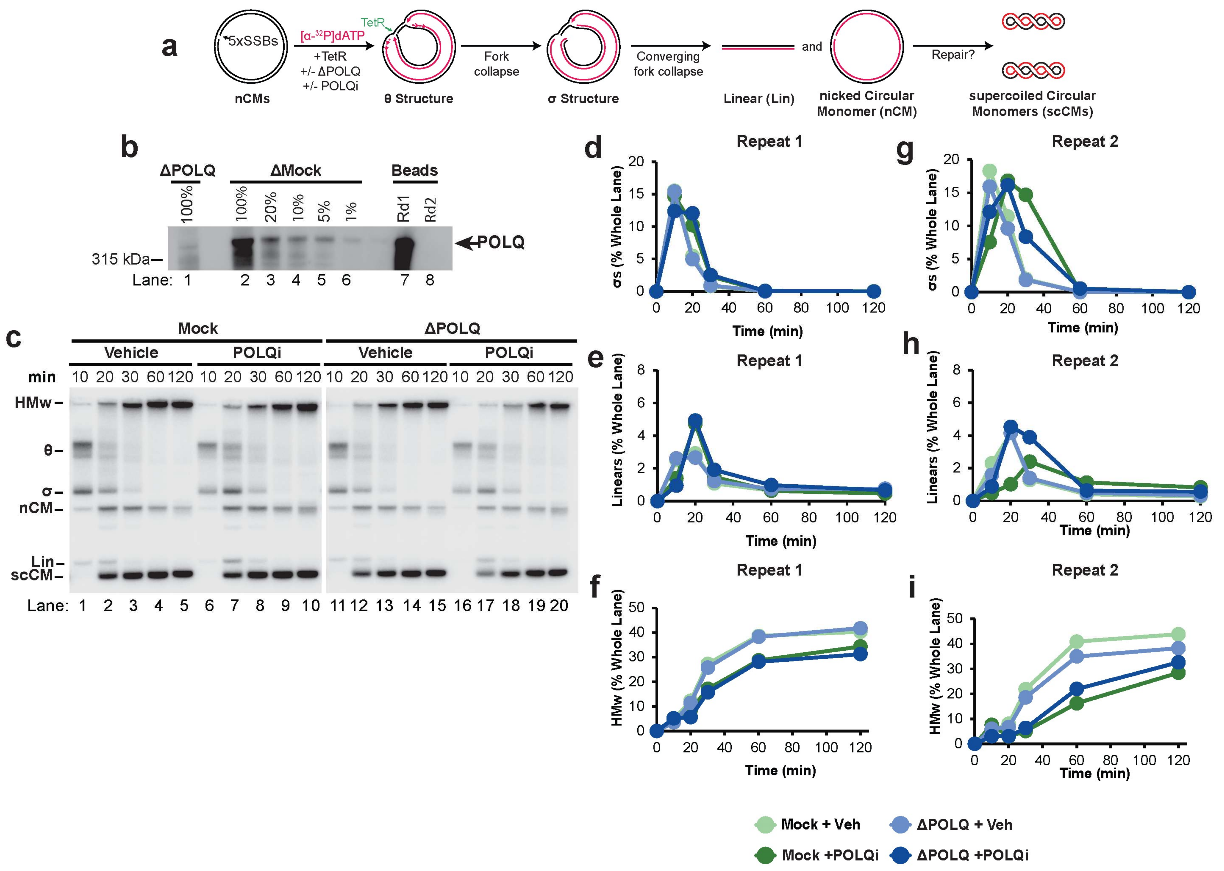

Reviewer Figure R3: Depletion and/or inhibition of POLQ does not impact resolution of collapsed ends **(A)** Schematic of the experimental design. Convergent collapse was induced by replication of pSSB in mock- or POLQ-immunodepleted extracts, each supplemented with either vehicle or POLQ inhibitor (POLQi). Nascent strands were radiolabeled by inclusion of [α-^{32}P]dATP. **(B)** Western blot confirming immunodepletion of POLQ from Xenopus egg extract. Serial dilutions of depleted (ΔPOLQ) and mock-depleted (ΔMock) extracts are shown alongside depletion beads (Rd1, Rd2). **(C)** Samples from (A) were separated on an agarose gel and visualized by autoradiography. Key species are indicated: HMw, θ, σ, nCM, Lin, and scCM. **(D)** Quantification of σ structures from (C), Repeat 1. **(E)** Quantification of linear molecules from (C), Repeat 1. **(F)** Quantification of HMw products from (C), Repeat 1. **(G)** Quantification of σ structures from (C), Repeat 2. **(H)** Quantification of linear molecules from (C), Repeat 2. **(I)** Quantification of HMw products from (C), Repeat 2. POLQ immunodepletion did not reduce HMw abundance, indicating that POLQ is dispensable for repair following convergent collapse. POLQi reduced HMw in both mock- and POLQ-immunodepleted extracts, indicating that the inhibitor effect persisted in the absence of POLQ and was therefore attributable to off-target activity. *n* = 2 independent experiments. See also Reviewer Fig. R4.

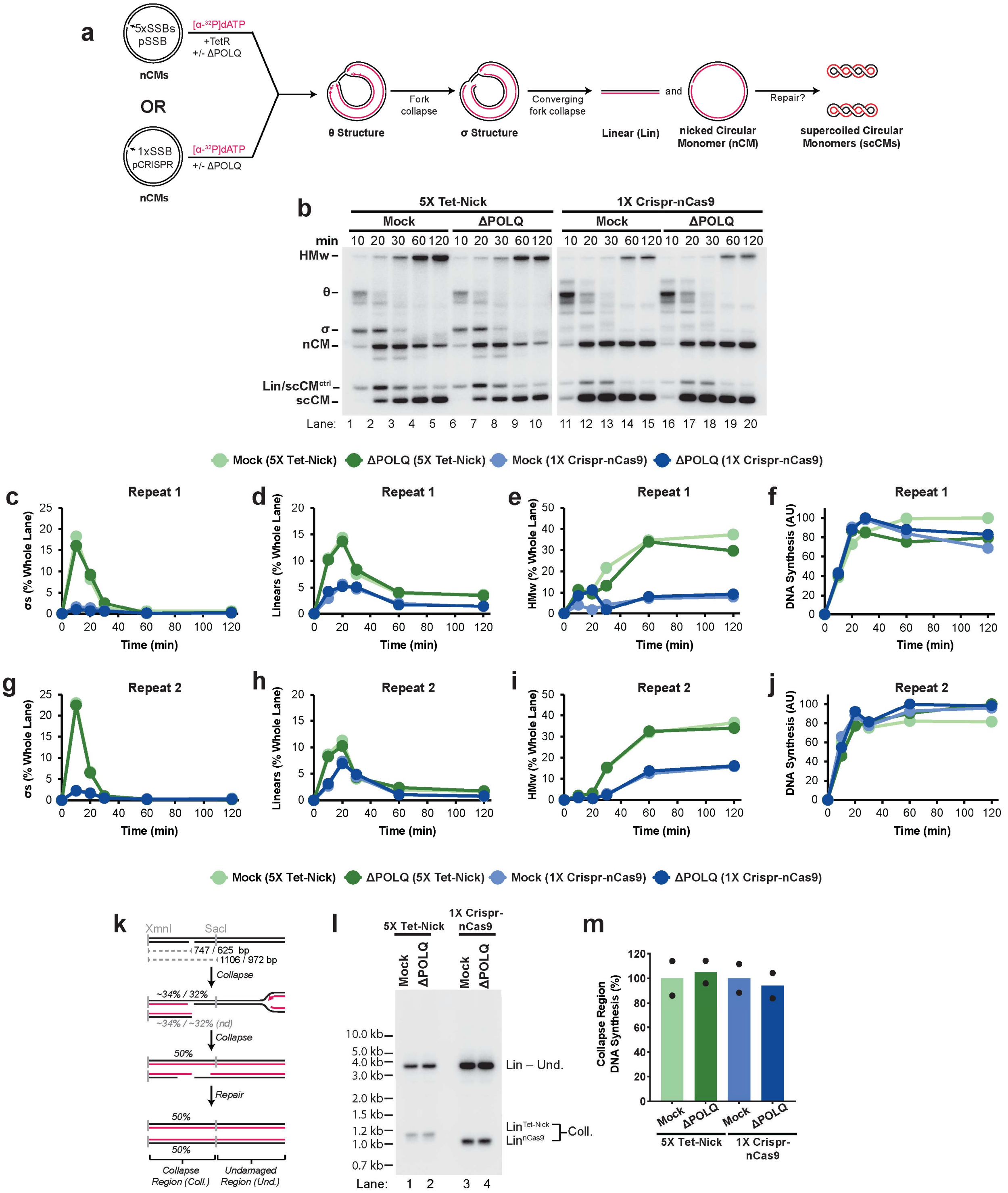

Reviewer Figure R4: Depletion of POLQ does not impact nascent DNA synthesis following collapse

(A) Schematic of the experimental design. Convergent collapse was induced by replication of pSSB (5X Tet-Nick) or pCRISPR (1X Crispr-nCas9) in mock- or POLQ-immunodepleted extracts. Nascent strands were radiolabeled by inclusion of [α-^{32}P]dATP. **(B)** Samples from (A) were separated on an agarose gel and visualized by autoradiography. Key species are indicated: HMw, θ, σ, nCM, Lin/scCM^{ctrl}, and scCM. Lanes 1-10 correspond to the 5X Tet-Nick substrate; lanes 11-20 correspond to the 1X Crispr-nCas9 substrate. **(C)** Quantification of σ structures from (B), Repeat 1. **(D)** Quantification of linear molecules from (B), Repeat 1. **(E)** Quantification of HMw products from (B), Repeat 1. **(F)** Quantification of DNA synthesis from (B), Repeat 1. **(G)** Quantification of σ structures from (B), Repeat 2. **(H)** Quantification of linear molecules from (B), Repeat 2. **(I)** Quantification of HMw products from (B), Repeat 2. **(J)** Quantification of DNA synthesis from (B), Repeat 2. **(K)** Schematic depicting the assay for collapse region DNA synthesis. Purified DNA samples were digested with XmnI and SacI to generate fragments corresponding to the collapse region (Coll.) and an undamaged region (Und.). Expected

signal values are indicated for each substrate. **(L)** Purified DNA samples from T=120 min in (B) were digested with XmnI and SacI, separated on an agarose gel, and visualized by autoradiography. Lanes 1-2, 5X Tet-Nick (Mock and ΔPOLQ); lanes 3-4, 1X Crispr-nCas9 (Mock and ΔPOLQ). **(M)** Quantification of collapse region DNA synthesis from (L). POLQ depletion did not alter collapse region DNA synthesis for either substrate, consistent with the conclusion that POLQ is dispensable for repair following convergent collapse. *n* = 2 independent experiments. See also Reviewer Fig. R3.

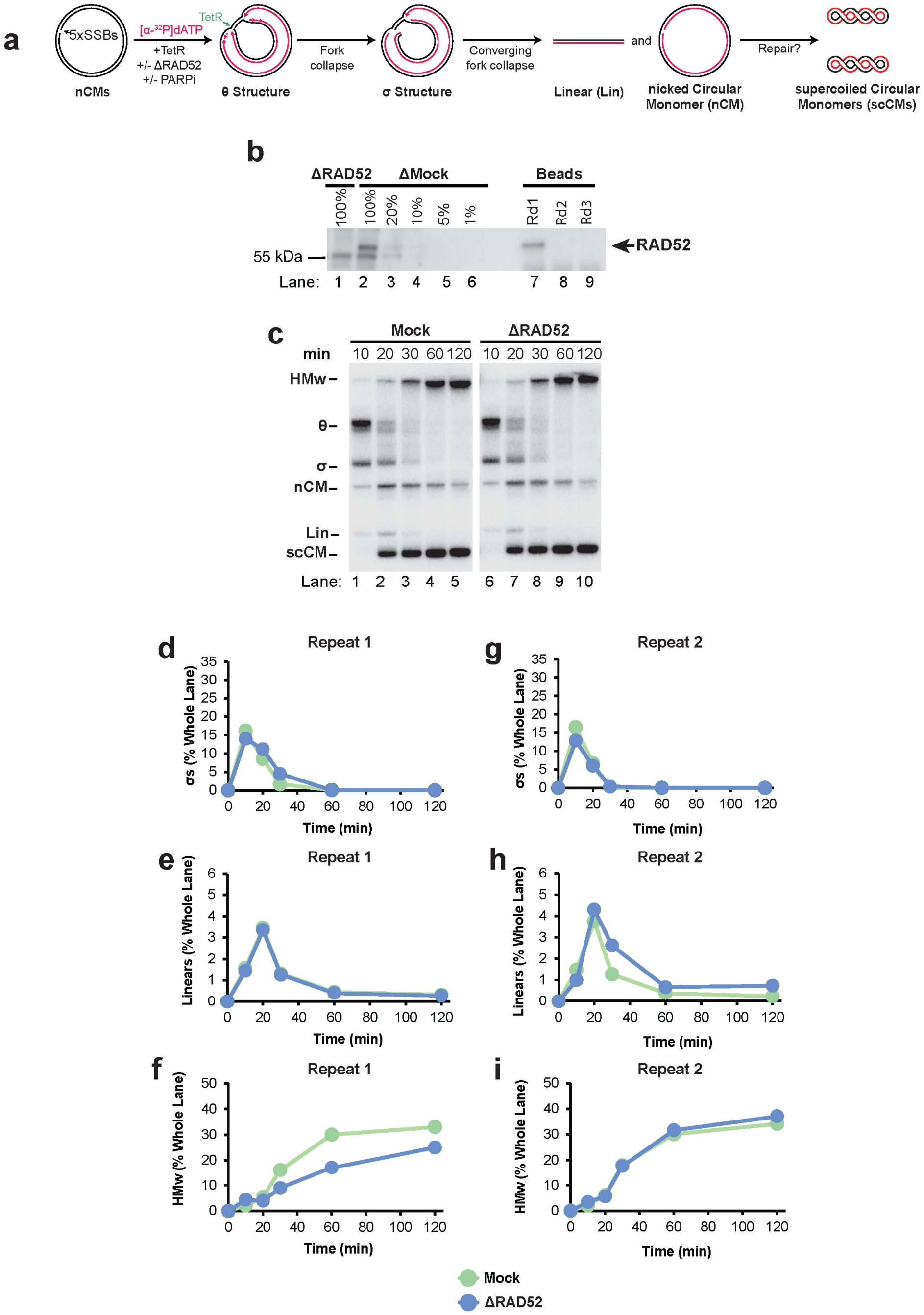

Reviewer Figure R5: Depletion of RAD52 does not impact resolution of collapsed ends

(A) Schematic of the experimental design. Convergent collapse was induced by replication of pSSB in mock- or RAD52-immunodepleted extracts. Nascent strands were radiolabeled by inclusion of [α-^{32}P]dATP. **(B)** Western blot confirming immunodepletion of RAD52 from Xenopus egg extract. Serial dilutions of depleted (ΔRAD52) and mock-depleted (ΔMock) extracts are shown alongside depletion beads (Rd1, Rd2, Rd3). **(C)** Samples from (A) were separated on an agarose gel and visualized by autoradiography. Key species are indicated: HMw, θ, σ, nCM, Lin, and scCM. **(D)** Quantification of σ structures from (C), Repeat 1. **(E)** Quantification of linear molecules from (C), Repeat 1. **(F)** Quantification of HMw products from (C), Repeat 1. **(G)** Quantification of σ structures from (C), Repeat 2. **(H)** Quantification of linear molecules from (C), Repeat 2. **(I)** Quantification of HMw products from (C), Repeat 2. RAD52 immunodepletion did not reduce HMw abundance, indicating that RAD52 is dispensable for repair of deDSBs following convergent collapse. *n* = 2 independent experiments. See also Reviewer Fig. R6.

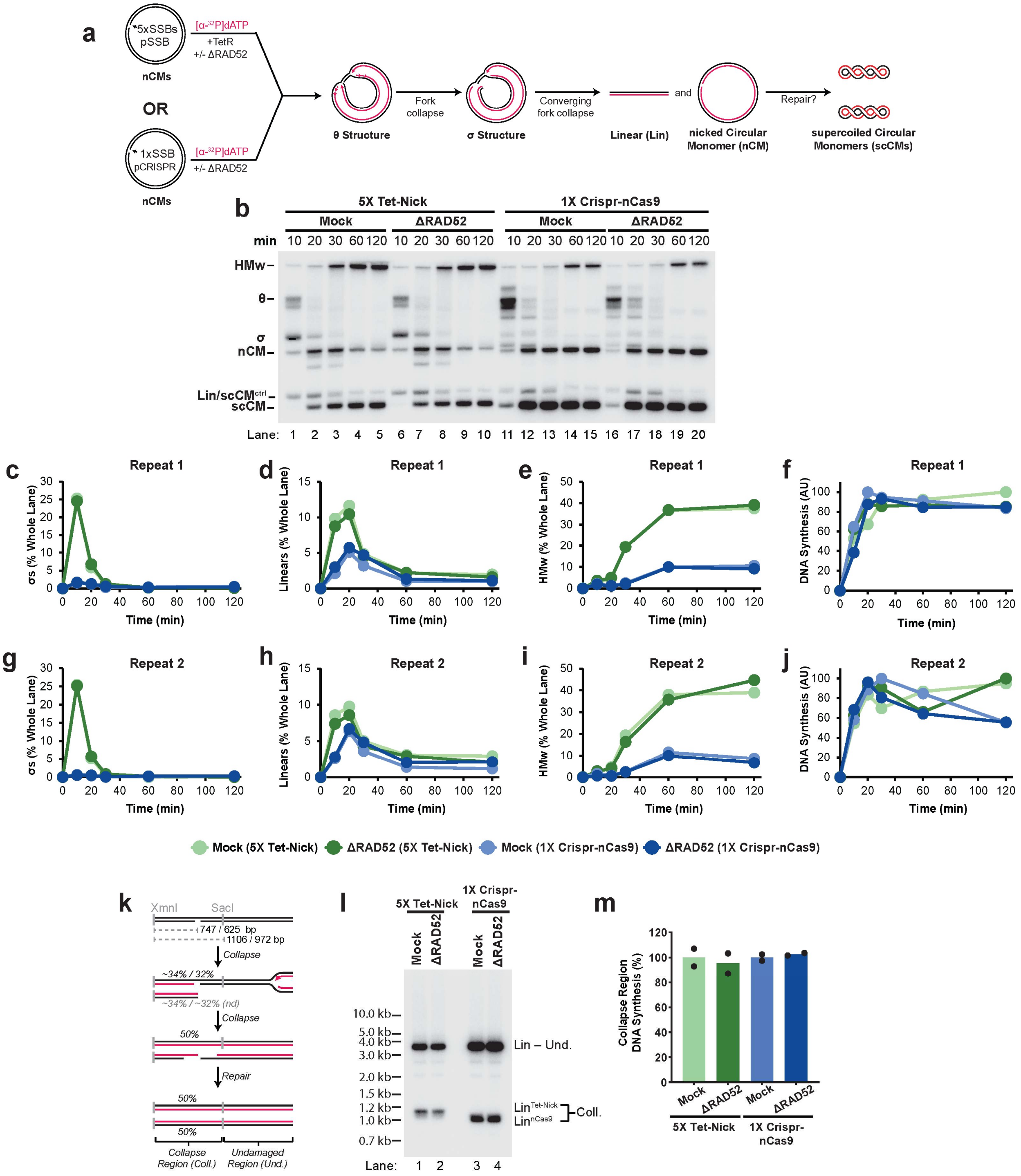

Reviewer Figure R6: Depletion of RAD52 does not impact nascent DNA synthesis following collapse

(A) Schematic of the experimental design. Convergent collapse was induced by replication of pSSB (5X Tet-Nick) or pCRISPR (1X Crispr-nCas9) in mock- or RAD52-immunodepleted extracts. Nascent strands were radiolabeled by inclusion of [α-^{32}P]dATP. **(B)** Samples from (A) were separated on an agarose gel and visualized by autoradiography. Key species are indicated: HMw, θ, σ, nCM, Lin/scCM^{ctrl}, and scCM. Lanes 1-10 correspond to the 5X Tet-Nick substrate; lanes 11-20 correspond to the 1X Crispr-nCas9 substrate. **(C)** Quantification of σ structures from (B), Repeat 1. **(D)** Quantification of linear molecules from (B), Repeat 1. **(E)** Quantification of HMw products from (B), Repeat 1. **(F)** Quantification of DNA synthesis from (B), Repeat 1. **(G)** Quantification of σ structures from (B), Repeat 2. **(H)** Quantification of linear molecules from (B), Repeat 2. **(I)** Quantification of HMw products from (B), Repeat 2. **(J)** Quantification of DNA synthesis from (B), Repeat 2. **(K)** Schematic depicting the assay for collapse region DNA synthesis, as in Reviewer Fig. R4K. **(L)** Purified DNA samples from T=120 min in (B) were digested with XmnI and SacI, separated on an agarose gel, and visualized by autoradiography. Lanes 1-2, 5X Tet-Nick (Mock and ΔRAD52); lanes 3-4, 1X Crispr-nCas9 (Mock and ΔRAD52).

(M) Quantification of collapse region DNA synthesis from (L). RAD52 depletion did not alter collapse region DNA synthesis for either substrate, consistent with the conclusion that RAD52 is dispensable for repair following convergent collapse. n = 2 independent experiments. See also Reviewer Fig. R5.

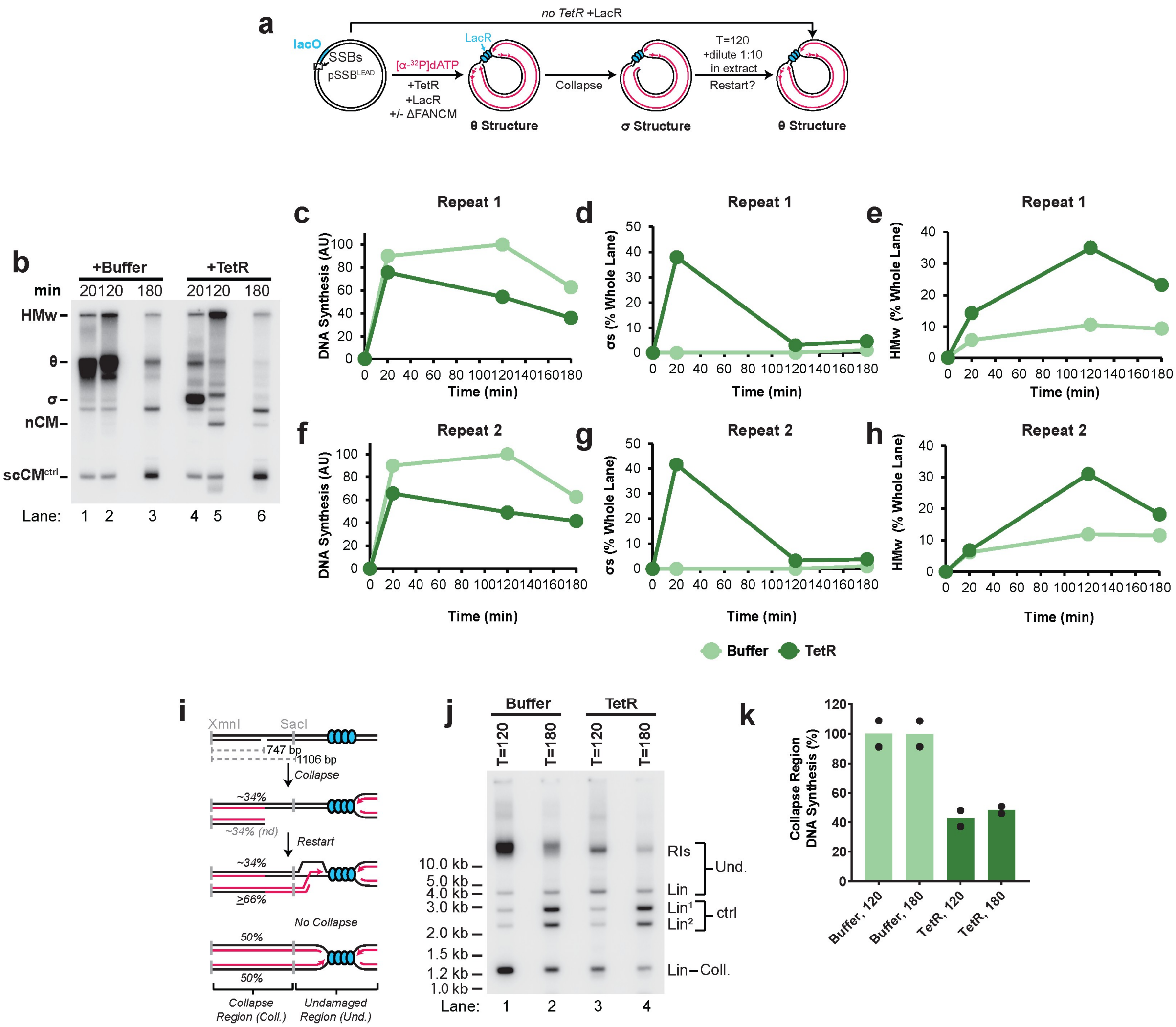

Reviewer Figure R7: Dilution of collapse DNA ends does not elicit fork restart

(A) Schematic of the experimental design. Leading collapse was induced by replication of $pSSB^{LEAD}$ in Xenopus egg extract in the presence of TetR and LacR. At T=120 min, collapsed reactions were diluted 1:10 into fresh nucleoplasmic extract (NPE) supplemented with [α-^{32}P]dATP to reduce the ratio of collapsed DNA ends to available repair factors. Reactions were then monitored for an additional 60 min (to T=180 min). Under standard reaction conditions, the density of DSBs per nucleoplasmic extract corresponds to approximately 42 DSBs per yeast genome equivalent of DNA (see calculations below). Following 1:10 dilution, this density was reduced to approximately 4 DSBs per yeast genome equivalent, which is comparable to the single DSB per genome at which break-induced replication operates efficiently in S. cerevisiae. **(B)** Samples from (A) were separated on an agarose gel and visualized by autoradiography. Key species are indicated: HMw, θ, σ, nCM, and $scCM^{ctrl}$. **(C)** Quantification of DNA synthesis from (B), Repeat 1. **(D)** Quantification of σ structures from (B), Repeat 1. **(E)** Quantification of HMw products from (B), Repeat 1. **(F)** Quantification of DNA synthesis from (B), Repeat 2. **(G)** Quantification of σ structures from (B), Repeat 2. **(H)** Quantification of HMw products from (B), Repeat 2. **(I)** Schematic depicting the assay for collapse region DNA synthesis. Purified DNA samples were digested with XmnI and SacI to generate fragments corresponding to the collapse region (Coll.) and an undamaged region (Und.). Expected signal values are indicated for no collapse, collapse without restart, and collapse with restart conditions. **(J)** Purified DNA samples from T=120 and T=180 min were digested with XmnI and SacI, separated on an agarose gel, and visualized by autoradiography. Lanes 1-2, +Buffer (T=120, T=180); lanes 3-4, +TetR (T=120, T=180). Replication intermediates (RIs), undamaged region (Und.), control fragment (ctrl), and collapse region (Coll.) fragments are indicated. **(K)** Quantification of collapse region DNA synthesis from (J). Dilution of collapsed DNA ends into fresh extract did not promote detectable restart of DNA synthesis, arguing against seDSB density as the explanation for the absence of restart. *n* = 2 independent experiments. DSB density calculation: each 1 ul reaction contains 0.33 ul NPE and 4.33 ng of a 4,662 bp plasmid (1 DSB per plasmid), yielding approximately 8.5 x 10^8 DSBs per 0.33 ul NPE. NPE is prepared from 1.19 x 10^8 sperm nuclei (haploid

genome, 3.1 Gb) concentrated into 500 ul, giving 2.38 x 10^5 nuclear equivalents per ul NPE. This yields approximately 1.08 x 10^4 DSBs per nuclear equivalent, or approximately 3.5 DSBs per Mb, equivalent to approximately 42 DSBs per *S. cerevisiae* genome equivalent (12.1 Mb). The 1:10 dilution in this experiment represents ~4.2 DSBs per *S. cerevisiae* genome equivalent and did not result in detectable restart.

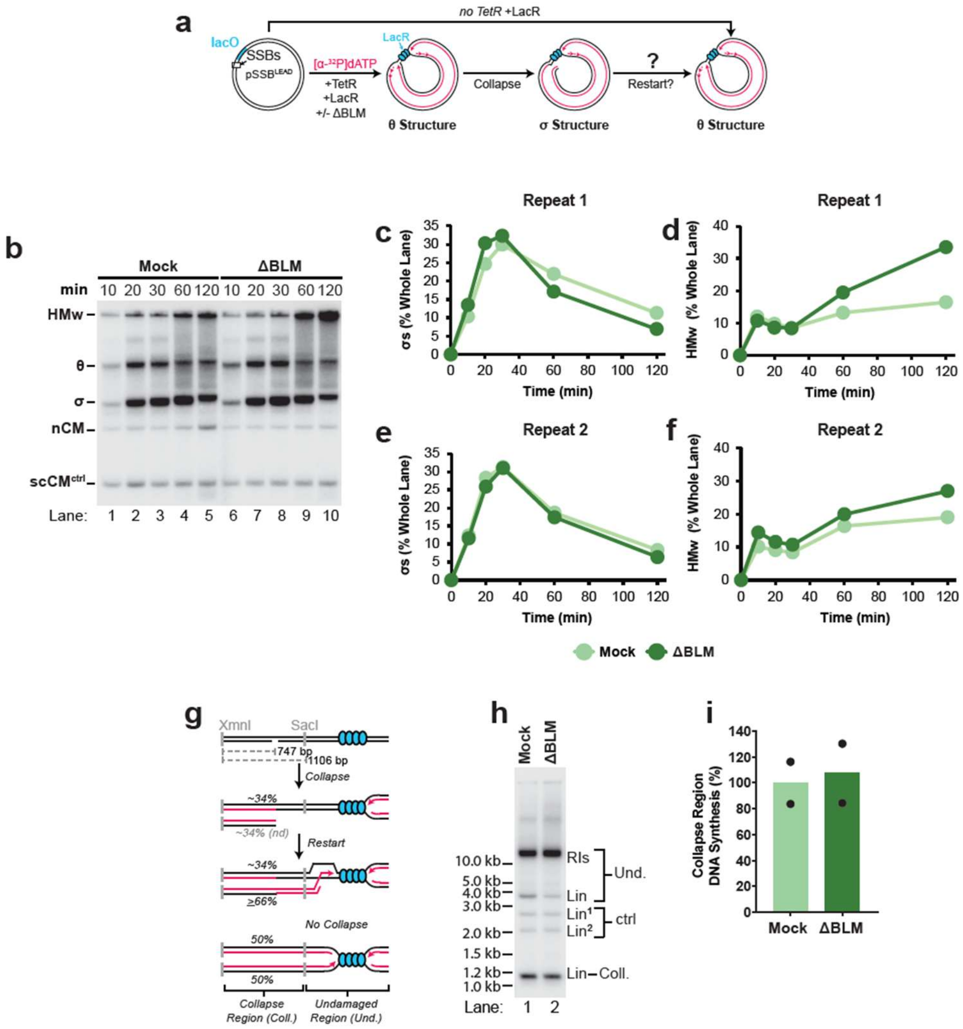

Reviewer Figure R8: Depletion of BLM does not elicit fork restart

(A) Schematic of the experimental design. Leading collapse was induced by replication of pSSB^{LEAD} in mock- or BLM-immunodepleted Xenopus egg extracts in the presence of TetR and LacR. Nascent strands were radiolabeled by inclusion of [α-^{32}P]dATP. **(B)** Samples from (A) were separated on an agarose gel and visualized by autoradiography. Key species are indicated: HMw, θ, σ, nCM, and scCM^{ctrl}. **(C-D)** Quantification of σ structures and HMw products from (B), Repeat 1. **(E-F)** Quantification of σ structures and HMw products from (B), Repeat 2. BLM depletion increased HMw abundance, consistent with accumulation of joint molecules that are normally dissociated by BLM. Lower panels: **(G)** Schematic depicting the assay for collapse region DNA synthesis, as in Fig. 1E. **(H)** Purified DNA samples from T=120 min in (B) were digested with XmnI and SacI, separated on an agarose gel, and visualized by autoradiography. Replication intermediates (RIs), undamaged region (Und.), control fragment (ctrl), and collapse region (Coll.) fragments are indicated. **(I)** Quantification of collapse region DNA synthesis from (F). BLM depletion did not reveal evidence of increased DNA synthesis in the collapse region, indicating that BLM depletion did not unmask restart at single collapsed forks. *n* = 2 independent experiments.

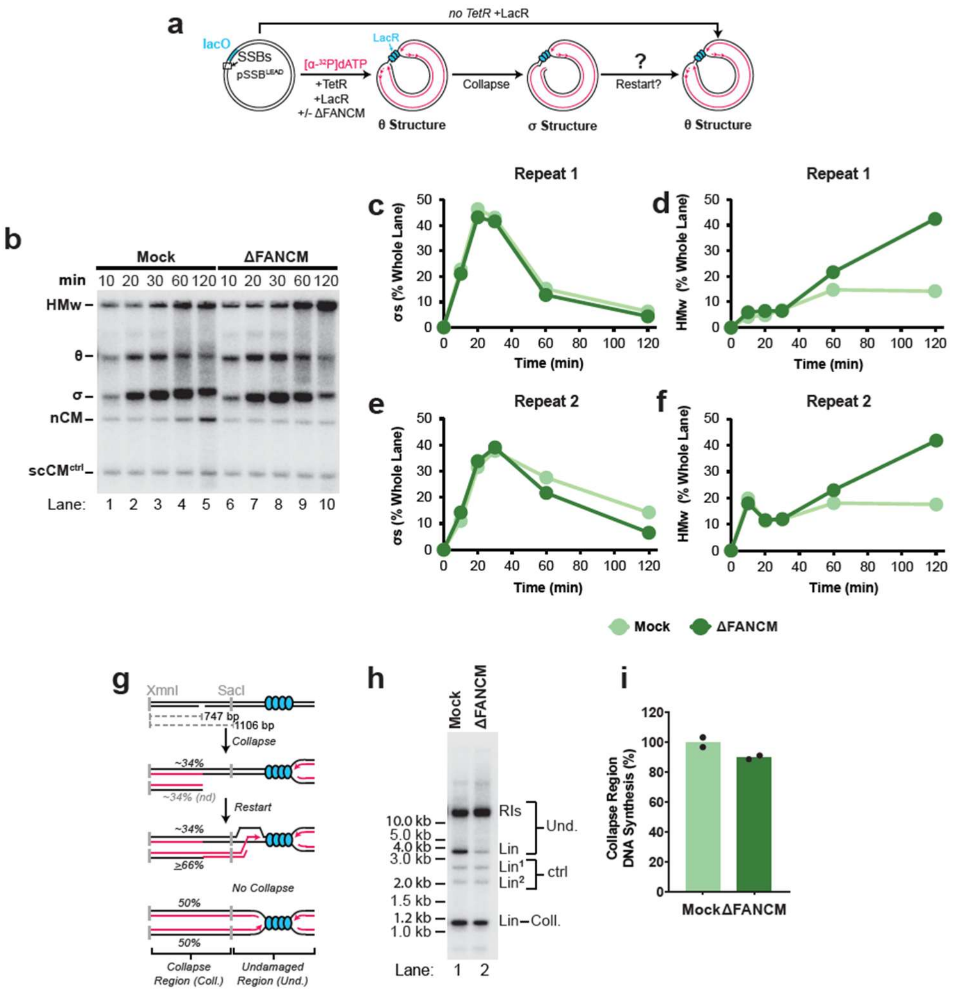

Reviewer Figure R9: Depletion of FANCM does not elicit fork restart

(A) Schematic of the experimental design. Leading collapse was induced by replication of pSSB^{LEAD} in mock- or FANCM-immunodepleted Xenopus egg extracts in the presence of TetR and LacR. Nascent strands were radiolabeled by inclusion of [α-^{32}P]dATP. **(B)** Samples from (A) were separated on an agarose gel and visualized by autoradiography. Key species are indicated: HMw, θ, σ, nCM, and scCM^{ctrl}. **(C-D)** Quantification of σ structures and HMw products from (B), Repeat 1. **(E-F)** Quantification of σ structures and HMw products from (B), Repeat 2. FANCM depletion increased HMw abundance, consistent with accumulation of joint molecules that are normally dissociated by FANCM. Lower panels: **(G)** Schematic depicting the assay for collapse region DNA synthesis, as in Fig. 1E. **(H)** Purified DNA samples from T=120 min in (B) were digested with XmnI and SacI, separated on an agarose gel, and visualized by autoradiography. Replication intermediates (RIs), undamaged region (Und.), control fragment (ctrl), and collapse region (Coll.) fragments are indicated. **(I)** Quantification of collapse region DNA synthesis from (F). FANCM depletion did not reveal evidence of increased DNA synthesis in the collapse region, indicating that FANCM depletion did not unmask restart at single collapsed forks. *n* = 2 independent experiments.

We thank the editor and reviewers for their detailed and constructive feedback on our manuscript. We are pleased that all three reviewers found our systematic analysis of fork collapse outcomes to be of considerable interest, and we are grateful for the many suggestions that have strengthened the work.

(1) All three reviewers raised questions about the secondary collapse model, including the mechanism, its dependence on resection, and its relevance to chromosomal contexts (responses #2, #13, #14, #22). We have performed two new experiments that provide additional support for this model. Addition of aphidicolin following collapse caused a substantial increase in nicked circular monomers (nCMs), consistent with DNA polymerase activity guarding against resection (new Extended Data Fig. 5F-I; response #2). nCMs were essentially eliminated when the sister fork was positioned further away (new Extended Data Fig. 2I-R; responses #2, #13), supporting the conclusion that secondary collapse arises from resection reaching the sister fork. We have also expanded the Discussion to address when secondary collapse could occur in a chromosomal context, including scenarios involving impaired homologous recombination (HR), stalled forks, or lagging collapse (responses #13, #22).

(2) All three reviewers requested characterization of the end-joining mechanism operating at convergent collapsed forks (responses #3, #10, #23, #29, #30). We have performed extensive new experiments establishing that convergent and single fork collapse produce distinct repair outcomes. Convergent collapse repair is independent of canonical NHEJ and MMEJ: immunodepletion of DNA-PKcs or POLQ had no effect on repair; small-molecule inhibitors of both pathways produced effects that persisted after immunodepletion, indicating off-target activity (Reviewer Figs. R2-R4; response #3). RAD52 activity was also dispensable (Reviewer Figs. R5-R6). In contrast, repair required resection activity, as immunodepletion of either MRE11 or CtIP inhibited the process (new Figs. 6A-H, 6I-P; response #3). Direct sequencing of repair products revealed that approximately half of sequenced products contained replication-dependent precise deletions within the tetO array, consistent with annealing-dependent DSB repair (new Fig. 7; response #3). To our knowledge, precise deletions arising from annealing-dependent DSB repair have not previously been observed following fork collapse. These error-prone products were not detected following single fork collapse, consistent with distinct repair outcomes at convergent versus single collapsed forks. Together, these data indicate that annealing-dependent DSB repair produces deletions specifically following convergent collapse, raising the possibility that this pathway could be harnessed for programmed deletions in a chromosomal context (lines 370-372).

(3) Reviewers 1 and 3 questioned the absence of replication restart following single fork collapse (responses #1, #21). We have performed two new control experiments. Moving the single-strand break (SSB) further from the lacO array revealed a low level of detectable repair that was negligible, consistent with a minority of forks converging on the nick rather than restart (new Extended Data Fig. 2I-R; response #1). Supplementation with a 10-fold excess of nucleoplasm did not promote detectable restart (Reviewer Fig. R7; response #21), arguing against seDSB density as a sufficient explanation for absent restart. Finally, we calculated the upper bound of restart that would be beyond the resolution of our experiments as ~10% (lines 327-330), consistent with stable D-loops being the dominant outcome.

(4) Reviewer 2 raised the possibility that seDSBs could be resolved prior to fork convergence during convergent collapse (response #15). We performed a fine time course of convergent collapse in the presence of BRC peptide (new Extended Data Fig. 6G-K) and found no detectable alteration of seDSB kinetics, arguing against HR-mediated resolution of seDSBs prior to fork convergence. MRE11 depletion also did not alter seDSB resolution (Fig. 6B-C). The ~1:1 ratio of linears to circular monomers under MRE11-depleted conditions (Extended Data Fig. 9C) is consistent with most breaks being converted to double-ended DSBs (deDSBs).

(5) Reviewers 2 and 3 raised concerns about the clarity of the writing (responses #16 and #24). In response we have removed the previous abasic site data to focus the manuscript (as suggested by reviewer 2, response #16). We have also endeavored to simplify the text as much as possible.

In total, we performed 18 new experiments addressing the reviewers' concerns, of which 9 are incorporated into the revised manuscript and 9 are provided as Reviewer Figures. We have also thoroughly re-written and simplified the entire manuscript. We believe the manuscript is substantially improved and is now suitable for publication.

Reviewer #1 (Remarks to the Author):

This work from Dewar and colleagues investigates the consequences of fork collapse at SSBs in a cell free system and describes how collapsed forks are resolved, depending on the nature of the SSB and on its location with respect to the replication fork. The authors take advantage of previously developed systems (nicks and nCas9 protected by TetR) to induce fork collapse at SSBs on plasmids in Xenopus laevis egg extracts (Vrtis, 2021), with some improvements to increase efficiency of fork collapse. In Vrtis et al., it was already described how the encounter between the replisome and a SSB leads to replisome disassembly by two distinct mechanisms depending on whether the SSB is located on the leading or the lagging strand. In this previous publication the intermediates of fork collapse were already identified (seDSBs) and a model for their resolution involving D-loop formation was proposed.
In this work, Dewar and colleagues confirm the model and show dependency on RAD51 for D-loop formation and generation of high molecular well-products. They also further address the resolution of SSBs during "convergent collapse" and show that, in this case, SSBs first generate seDSBs, before being converted into deDSBs by fork convergence. They also show that high molecular weight products still form but are not dependent on RAD51. Finally, the authors investigate the contribution of PARP to this system, showing that PARP can contribute to the repair of SSBs flaked by a DNA binding protein.

This is a comprehensive and detailed analysis of the outcomes of fork collapse at SSBs. Although some of the outcomes of fork collapse at SSBs had been anticipated by previous studies, this analysis has been conducted rigorously and systematically.
On the other hand, a few conclusions are unexpected based on the literature. In particular, the complete absence of fork restart after collapse in this system and the claimed dependency of high molecular weight products on end-joining during convergent collapse are somewhat surprising and would need some further elucidation before I can recommend publication of the manuscript in NSMB.

Major points:
1) Replication fork restart mechanisms after collapse have been widely described in cell-based systems and rely on recombination-dependent mechanisms. In the presented SSB replication fork collapse model in egg extracts, fork restart is never observed. Because a LacR-LacO array is in close proximity to the SSB sites, is it possible that protein binding or topological constraints might inhibit fork restart? Could the authors place the LacR-LacO array further away from the SSBs and show that fork restart is still undetectable?

#1 We thank the reviewer for raising this important point. We have now performed this experiment by moving the SSB further from the lacO array (new Extended Data Fig. 2I-R). Repair was detectable but negligible, consistent with a minority of forks converging on the nick rather than restart at the seDSB (lines 98-102). These data are consistent with our conclusion that we detect no evidence of replication restart at single collapsed forks.

2) In Fig 4. the authors show that generation of scCM is dependent on MRE11 and propose a model by which these circular monomers are the result of extensive resection of the leading template containing the SSBs. This secondary collapse model predicts generation of very long stretches of ssDNA that should be coated by RPA and activate the checkpoint. Could the authors provide evidence of this to strengthen their model of resection past divergent lagging strands? This seems particularly important because MRE11 depletion also blocks formation of well products (D-loops), and it is therefore impossible to exclude that the very small proportion of observed scCM might originate from an alternative mechanism.

#2 We thank the reviewer for this thoughtful suggestion. We apologize that the original manuscript did not clearly describe the distinct effects of RAD51 and MRE11 on these products. RAD51 inhibition blocked D-loop formation but increased circular monomer (CM) production (Fig. 2B; Extended Data Fig. 3B), whereas MRE11 depletion abolished both D-loops and CMs (Fig. 3E-H). We have clarified this distinction in the revised manuscript (lines 122-124, 163-166).

We performed the Chk1 analysis as the reviewer suggested. Chk1 phosphorylation was not substantially altered following collapse (Reviewer Fig. R1). This result neither supports nor contradicts

the model, because secondary collapse is a rare event that may not generate sufficient single-stranded DNA (ssDNA) to substantially alter Chk1 phosphorylation levels.

To test our hypothesis that DNA polymerase activity guards against resection, we added aphidicolin following collapse. This treatment caused a substantial increase in nCMs (Extended Data Fig. 5F-I), consistent with DNA polymerase activity guarding against resection. We also performed experiments using a larger backbone plasmid to move the sister fork further away. nCMs were essentially eliminated when the sister fork was positioned further away (new Fig. 3I-K), strongly supporting the conclusion that secondary collapse arises from resection reaching the sister fork. Together, these data provide additional support for the secondary collapse model.

3) In Fig. 5 the authors show that during convergent collapse the HM well products are independent of RAD51 (D-loop) and they propose the action of an end-joining mechanisms. Il would be important to probe this hypothesis by showing decrease of well products after inhibition of NHEJ (for example with NU7441, DNA-PKcs inhibitor (Zhu,2016)) and/or MMEJ (for example by depleting pol Theta, (Mann, 2022)).

#3 We thank the reviewer for this excellent suggestion. The experiments prompted by this comment led to a more complete characterization of the repair mechanism. We tested the roles of DNA-PKcs and POLQ as suggested, using both immunodepletion and the suggested small-molecule inhibitors (Reviewer Figs. R2-R4). Immunodepletion of either DNA-PKcs or POLQ had no effect on repair. Small-molecule inhibitors of both pathways produced effects that persisted after immunodepletion, indicating off-target activity (Reviewer Figs. R2-R4). We also tested whether RAD52 activity was required but found that it was not necessary following collapse at either tetO-protected nicks or nCas9 induced nicks (Reviewer Figs. R5-6). Thus, in this system, repair at convergent collapsed forks is independent of RAD51, RAD52, DNA-PKcs, and POLQ.

We next asked whether the mechanism required resection activity. Repair was dependent on resection, as immunodepletion of either MRE11 (new Fig. 6A-H) or CtIP (new Fig. 6I-P) inhibited the process. To characterize the repair products directly, we performed sequencing of the repair products (new Fig. 7). Sequencing revealed precise deletions within the tetO array (new Fig. 7B-C). The replication-dependent fraction of deletions was ~50% of total reads, corresponding to essentially all repair products, consistent with an efficient annealing-dependent DSB repair mechanism (new Fig. 7B-C). These deletion products were dependent on replication (new Fig. 7B-C) and diminished by MRE11 depletion (new Fig. 7H-I), as expected for products of repair. These products were absent from leading collapse (Fig. 7L-M), consistent with a distinct repair outcome at convergent versus single collapsed forks.

Together, these data indicate that annealing-dependent DSB repair produces deletions specifically following convergent collapse.

Minor points:
1) In fig 1F the nature of the “σ*” species should be explained.

#4 We now explain in the text that this is a topoisomer of the σ species (lines 478-479).

2) In Fig 6B lane 16-20 the equilibrium between scCM and nCM is shifted. The authors should better explain why in the text.

#5 We now explain in the text that persistent nicking by nCas9 prevents the compensatory supercoiling that otherwise accompanies nucleosome removal during deproteinization, which shifts the equilibrium toward nicked species (lines 230-232).

3) Line 339 “collapse at collapsed forks” should probably be corrected to “collapse at converging forks”?

#6 We thank the reviewer for identifying this typo. This is now corrected.

Reviewer #2 (Remarks to the Author):

In this study, Conwell and colleagues examine the generation and fate of collapsed replication forks using plasmids and Xenopus egg extracts. The authors investigate collapsed forks on leading or lagging strands generated by the collision of the replisome with “simple” nicks, Cas9 H840A-induced nicks, and abasic sites. The experiments are well designed, and the conclusions are supported by the results. The lack of DNA synthesis following seDSB invasion is a novel finding that expands current models of seDSB repair, a process that remains poorly understood. My main concern is that some of the observed events don not likely reflect physiological conditions, wherein chromatin context and regulated DNA end resection play critical roles. Therefore, I recommend that the authors temper some of their conclusions, particularly when extrapolating findings to cellular contexts. The paper is recommended for acceptance pending revisions addressing the following points:

#7 We thank the reviewer for their supportive appraisal of the manuscript. In the revised manuscript, we more carefully acknowledge potential differences between our in vitro system and cellular contexts. We now use "semi-synchronous" throughout to describe convergent collapse in our system, note that our extracts are proficient for HR, and discuss synchrony as a potential explanation for the predominance of HR-independent repair at convergent collapsed forks (lines 337-353).

-While the schematics in Figures S1M and 1E help illustrate the expected restriction digestion products, it remains unclear for this reader how DNA synthesis is measured.
While some descriptions are in the methods, these explanations are difficult to follow for even a specialist. Given that this measurement supports major conclusions of the study, it would be helpful if an example is included in the supplementary materials or clarified otherwise. This would significantly help readers understand the approach.

#8 We thank the reviewer for bringing this to our attention. In the revised manuscript we have included a table illustrating how the experimentally measured values are affected by different efficiencies of collapse and repair, which are the major variables influencing the underlying data. We also include a worked example in Supplementary Figure 2.

-Could the authors provide a zoomed-in view of the E-E products (with increased exposure) in Figure S2C to more clearly demonstrate the reduction of E-E products following RAD51 inhibition? This is a bit hard to see.

#9 We have now added a zoomed-in view with increased exposure (Extended Data Fig. 3C) and also included quantification of these species for additional clarity (Extended Data Fig. 3E).

-Is the egg extract used in these experiments best characterized as a purely S-phase environment, or does it reflect a mixture of multiple cell cycle phases? Recent studies (PMID: 39631395; PMID: 39631396) suggest that non-homologous end joining (NHEJ) is not a predominant mechanism for repairing collapsed forks during S phase under physiological conditions. It would therefore be helpful for the authors to include a brief discussion on how the cell cycle context of the extract might influence the observed repair outcomes. While end joining could still occur in this system, it may be regulated or suppressed in the S-phase in vivo, and acknowledging this would strengthen the interpretation of the data.

#10 We thank the reviewer for raising this important point. The extracts we use are derived from replicating nuclei and contain high CDK and DDK activity, mimicking S phase of the early embryonic cell cycle. We cannot formally define these as "S phase" because there is only interphase and mitosis during early Xenopus development, but these extracts are proficient for HR as demonstrated by robust RAD51-dependent repair at single collapsed forks (Figs. 1-2). Less concentrated versions of these extracts (G1-like extracts) favor NHEJ (PMID 26990988), and supplementation with mitotic CDK activates different repair pathways (PMID 30849395), indicating that cell cycle context modulates

pathway choice in this system. In the revised manuscript, we now discuss these points and note that end joining can act throughout S phase in human cells (PMID 22841003; lines 351-352). Our favored explanation for the HR independence of convergent collapse repair is that the synchrony with which the two DNA ends form favors HR-independent pathways, rather than cell cycle context per se (lines 342-353).

-“Importantly, none of these data show evidence for replication restart at a single collapsed fork, despite efficient resolution of seDSBs.”
What is the meaning of efficient resolution of seDSB in this context? Is it D-loop formation? Please clarify.

#11 We apologize that this was not sufficiently clear. In the revised manuscript, we define "DSB resolution" broadly as removal of the free DNA end (line 40), which encompasses both repair events (D-loop formation, end joining) and the degradation events we observe (secondary collapse). In this context, "efficient resolution of seDSBs" refers to the conversion of seDSBs to joint molecules arising from D-loops and other products, without detectable restart of DNA synthesis.

-What is the σ* in Fig4F that appears in MRE11 deficient cells?

#12 These species correspond to topoisomers of σ species, as noted above (response #4) (lines 478-479).

- The proposed “secondary collapse” model is a plausible explanation for the formation of full-length products at leading-strand collapse in the absence of DNA synthesis within this system. However, its applicability to eukaryotic cells is unclear. In vivo, the DNA ends generated at leading-strand breaks would likely require extensive resection to reach a converging replication fork, which may be unrealistic due to chromatin. Reported resection lengths in wild-type mammalian and yeast cells were reported to be ~5 kb or less (PMID: 30673606; PMID: 38900911), likely insufficient to reach the divergent replication fork.
The authors should discuss the relevance and potential limitations of this secondary collapse model in a cellular context and speculate when this could actually happen. Possible scenarios might be a stalled fork in front of a collapsed fork? Could this occur under conditions that cause replication stress?

#13 We thank the reviewer for this thoughtful suggestion. In the revised manuscript, we directly tested the prediction that extensive resection is required for secondary collapse. Secondary collapse was essentially eliminated when the sister fork was positioned further away (new Fig. 3I-K), consistent with a requirement for extensive resection. Our favored model is that secondary collapse could occur when resolution of the broken end is impaired, for example due to deficient HR activity, given that RAD51 inhibition increases circular monomer production (Fig. 2B) and increased resection is observed in cells under these same conditions (Fig. 3 of PMID 30673606; and Fig. 2 of PMID 38900911). We appreciate the reviewer's suggestion that an adjacent stalled fork could also facilitate secondary collapse and have incorporated this scenario. We also note that secondary collapse could influence whether lagging collapse generates single- or double-ended DSBs. We discuss these models in the revised Discussion (lines 384-396) and incorporate them into our revised model (Fig. 8D-E).

- According to the lagging-strand collapse model, a secondary collapse does not occur because the template strand of the converging fork remains intact (Fig. 4I), allowing DNA polymerases to re-synthesize the degraded strand. Could this hypothesis be tested directly by inhibiting DNA synthesis - for example, using polymerase inhibitors such as aphidicolin after collapse? This would help determine whether the recovery of full-length products indeed depends on re-synthesis from the converging fork and further support the proposed mechanism.

#14 The reviewer raises an excellent point. We have clarified the mechanistic basis for this asymmetry in the revised Discussion. Secondary collapse is specific to leading collapse because for lagging

collapse, 5'-3' resection targets the nascent rather than parental strands (lines 387-388). Our model predicts that aphidicolin would enhance secondary collapse following leading collapse, where 5'-3' resection targets the parental DNA strand. This is indeed the case (Extended Data Fig. 5F-I), consistent with DNA polymerase activity guarding against resection.

- “Thus, seDSBs were resolved prior to deDSBs. During convergent collapse, seDSBs were ~2.5 fold lower than for leading and lagging collapse (~20%in Fig. 5D; ~50% in Fig. 1C, Fig. 3C), suggesting rapid conversion to deDSBs by the converging fork. deDSBs were also 3-fold less abundant than seDSBs (~6% in Fig. S5B; ~20% in Fig. 5D) suggesting that deDSBs were more readily resolved than seDSBs”

While it is possible that deDSBs are resolved more quickly than seDSBs, an alternative explanation is that some leading or lagging seDSBs may invade the template strand prior to the arrival of the converging fork and subsequently merge with it without generating a detectable deDSB. This scenario could also account for the higher percentage of detected seDSBs compared to deDSBs - perhaps not all seDSBs are converted into deDSBs if they engage in strand invasion before fork convergence.

#15 We thank the reviewer for raising this alternative interpretation. We agree that the original data did not rule out this possibility. In the revised manuscript, we provide new experiments to address this concern. We performed a fine time course of convergent collapse in the presence of BRC peptide to block resolution of seDSBs (new Extended Data Fig. 6G-K). BRC peptide did not detectably alter seDSB kinetics during convergent collapse, arguing against rapid HR-mediated resolution of seDSBs prior to fork convergence. MRE11 depletion also did not alter seDSB resolution (new Fig. 6B-C). The ~1:1 ratio of linears to circular monomers under MRE11-depleted conditions (new Extended Data Fig. 9C) is consistent with most breaks being converted to deDSBs rather than being resolved as seDSBs. Together, these data argue against seDSB resolution prior to fork convergence.

-Although the observation that abasic sites generate collapsed forks in this system is interesting, the benefit of including these data sets in the manuscript is not entirely clear - particularly since PARP inhibition did not influence break formation or resolution. I would suggest removing this section which seemed like an unnecessary distraction in a manuscript that is already hard to follow.

#16 We appreciate the reviewer's comment. Given the substantial data we have added with the revision, we have now removed this figure from the manuscript.

Minor

-line 421: The longer duration between initial seDSB formation and fork convergence may have led to extensive degradation of the seDSB, which might preclude end joining.
The wording “extensive degradation” is referring to physiological resection. The way this is written makes it seem pathological. Please re-phrase it (ie. Extensive resection of the seDSB).

#17 We now use "extensive resection" in place of "extensive degradation" throughout the revised manuscript.

-line 87: “We observed similar outcomes at SSBs generated by Cas9 nickases.”
The authors only investigated Cas9 nickase H840A, this should be pointed here instead of nickases.

#18 We have updated this to specify Cas9 nickase H840A (line 67).

-It would be helpful to have the origin pointed in the schematics, so the reader can better visualize bidirectional fork progression until it reaches the TetR site.

#19 We thank the reviewer for this suggestion. In this system, replication initiation is distributed throughout the plasmid rather than occurring at a defined site, so a single origin cannot be marked in the schematics. We now state this explicitly in the Fig. 1A legend: "Plasmids harbor multiple sequence non-specific origins distributed throughout the backbone" (lines 408-410). We have updated the schematics to indicate distributed origin firing rather than a single origin.

- please add nickase Cas9 H840A in the model in figure 8.

#20 We have removed the initial panel from the model figure because the abasic site figure (previously Fig. 7) was removed from the manuscript (see response #16). The revised model figure no longer explicitly references Cas9 nickase.

Reviewer #3 (Remarks to the Author):

This m/s uses an established system in frog egg extracts to study repair and replication consequences of replication fork collision with a leading or lagging ssDNA break (SSB). The principal finding is that the systems examined do not reveal evidence of recombination-mediated resumption of DNA synthesis at the site of the nick. Instead, seDSBs induced by either leading or lagging strand fork breakage (in this m/s termed “collapse”) were shown to generate high MW products that were Rad51-dependent and these products appeared as sigma structures disappeared. Thus, Rad51-mediated repair of seDSBs involving a neighboring DNA molecule was a major repair product in this system. High MW products were resolved by RuvC, suggesting that they contain joint molecules/recombination intermediates. The inference that the HR structures generated are D-loops is plausible but not proven. In addition, end-to-end fusions can also form, by mechanisms that were not investigated in this study. Leading strand seDSBs differ from lagging seDSBs by the capacity of MRE11-mediated DNA end resection to effectively remove the free DNA end of the sigma structure, leading to formation of a product that resembles the original plasmid. This is explained by the resection process proceeding around the circular plasmid substrate, causing breakage/collapse of the opposing LacR-blocked fork. The same does not happen for lagging strand seDSBs, because of the continuity of the non-resected strand of the sigma structure (the parental lagging strand of the nick; the parental leading strand of the opposing fork). This ‘secondary fork collapse’ occurs because the substrate is a small circular plasmid. A Cas9 nickase system generated similar results to those described above and addition of a PARP inhibitor accentuated the potency of a leading strand nick.

Why HR/BIR-mediated replication restart is not observed in this system is unclear. However, the experimental system generates numerous synchronous seDSBs from plasmids in close proximity, presenting an opportunity for repair by either HR or end joining with a neighboring molecule (as shown in the data), which might swamp any potential BIR response. The question of when/how BIR is initiated at a nick-collapsed fork could therefore relate to the abundance of competing substrates for repair. Alternatively, BIR in yeast typically arises after some delay in the processing of a DSB (PMID: 27075725). This delay is abolished in mph1 and sgs1 mutants, suggesting that BIR is actively suppressed by these genes in wild type cells. Perhaps BIR-type restart in the authors’ system is a last resort that is never activated, because of the host of competing alternative repair pathways available (HR- or end joining-mediated).

The data is clear and carefully presented, which is a strength of the m/s.

We thank the reviewer for this detailed and thoughtful assessment.

Some weaknesses include the frog egg extract plasmid system itself. This experimental tool floods the system with near-synchronous seDSBs, which might limit the ability to study perhaps rare, late outcomes of fork collapse (potentially including fork restart processes).

#21 In the revised manuscript, we address the concern that seDSB density could prevent detection of restart. We induced collapse and then supplemented with a 10-fold excess of nucleoplasm (Reviewer Fig. R7). This dilution reduced the ratio of DSBs per nucleoplasm to levels comparable to experiments in yeast where restart is observed (see legend for Reviewer Fig. R7), arguing against seDSB density as the explanation for absent restart. We also note that increasing seDSB density in human cells does not appear to alter the repair mechanism (PMID 38900911). In the Discussion (lines 330-335), we state that our inability to detect BIR is consistent with evidence that most fork collapse events involve deDSB formation and that BIR proteins do not appear to be major determinants of PARP inhibitor cytotoxicity, while acknowledging that we cannot rule out unique properties of our in vitro system.

The use of a small circular plasmid revealed the ‘secondary fork collapse’ phenomenon, but the significance of this effect on a large linear vertebrate chromosome is unclear.

#22 We thank the reviewer for raising the question of secondary collapse relevance in a chromosomal context. In the revised manuscript, we now discuss this point in the Discussion (lines 384-396). Secondary collapse was essentially eliminated when the sister fork was positioned further away (new Fig. 3I-K; see also response #13), consistent with a requirement for extensive resection. We propose

that secondary collapse could operate when HR is deficient or when an adjacent fork stalls after origin firing (Fig. 3, lines 390-392). We also note that secondary collapse could negatively regulate deDSB formation following lagging collapse (Fig. 8E, lines 392-395) and state that "Understanding the cellular contexts in which this mechanism occurs is an important future direction" (lines 395-396).

The end joining mechanisms are not explored.

#23 We thank the reviewer for this comment. In the revised manuscript, we have now extensively characterized the end-joining mechanism at deDSBs arising from convergent collapse. We show that this repair is independent of DNA-PKcs, POLQ, and RAD52, but is dependent on resection by MRE11 and CtIP. Direct sequencing reveals precise deletions consistent with annealing-dependent DSB repair (see response #3 to Reviewer 1 for details). We have also characterized the end-to-end fusions at seDSBs and show that these involve short stretches of microhomology, consistent with alternative end joining (response #30, below).

Some of the text is very dense and some terms are puzzling, making the m/s sometimes difficult to follow. One term that this reviewer had difficulty with is 'divergent' fork (when it is in fact the convergent fork at the nick site, but accessed via resection around the entirety of the small circular plasmid).

#24 We apologize that aspects of the writing were difficult to follow. We have endeavored to improve clarity throughout the revised manuscript, including removal of prior abasic site data (as suggested by Reviewer 2, response #16). We have updated the "divergent fork" terminology to the standard "sister fork" terminology throughout. We also specify in the legend for Figure 1A that a "1.6 kb lacO array bound by LacR blocks converging forks at a distance" (line 406).

Specific points

1. Fig 1b why do the sigma structures migrate more slowly over the time-course? Is this because of DNA end resection?

#25 We thank the reviewer for this question. The slower migration of sigma structures over time is due to resection. As stated in the Fig. 3 legend (lines 480-482): "sigma structures migrated more slowly over time in mock but not MRE11-immunodepleted conditions, indicating the slowed migration was due to resection, presumably because of secondary structures formed by exposed single-stranded DNA."

2. Fig 2a The formation of D-loops is inferred but not demonstrated. It might be better to use the term "joint molecules", since Holliday junctions have not been excluded experimentally.

#26 We agree that we have not formally ruled out Holliday junctions. In the revised manuscript, we refer to these species as "joint molecules arising from D-loops" (first used at lines 129-130) to account for the possibility that they could contain D-loops or Holliday junctions, which would form downstream of D-loops.

3. How does depletion of FANCM or BLM (MPH1 and SGS1 homologs) affect the pattern of recombination products? (Does their depletion reveal a fraction of restart events at the nick-collapsed fork?)

#27 We thank the reviewer for this suggestion. Depletion of either FANCM or BLM increased joint molecule formation (Reviewer Figs. R8-R9), consistent with these species containing D-loops that are negatively regulated by FANCM and BLM. However, we did not observe increased synthesis in the collapsed region and thus did not detect evidence of restart. We did not include these data in the revised manuscript, which is already very dense as the reviewer noted.

4. As noted above, the terms “divergent fork” and “Diverging lagging strands” were confusing.

#28 We have updated the language, as noted in response #24.

5. Fig 5i BRC4 peptide doesn’t seem to inhibit very effectively. The authors interpret this effect as meaning that there is a limited requirement for HR at convergent collapsed forks. Alternatively, Rad51 loading might be more efficient in this setting than at a seDSB, exposing the limited ability of the BRC4 peptide to completely block Rad51 loading.

#29 We thank the reviewer for raising this alternative interpretation. In the revised manuscript, we also immunodepleted RAD51 and observed the same limited effect on repair (new Extended Data Fig. 7A-F). We also note that BRC peptide is highly inhibitory at deDSBs in other contexts in the extract system we use (PMIDs 21719678 and 35641523). These results, together with the deletion sequencing data revealing a high level of precise deletions (responses #3, #23), support the conclusion that, in this setting, convergent collapse repair occurs largely independently of HR.

6. What model do the authors propose for the conversion of seDSBs to end-to-end fusions by RAD51?

#30 Sequencing reveals that these fusions occur at sites of microhomology, suggesting limited resection. We suggest that the dsDNA-binding activity of RAD51 may limit resection and promote these fusions. We now discuss this model in the manuscript (lines 374-382).

7. Some typos/word choices make the reading more difficult e.g.: lines 339-340: “Nonetheless, these results support the conclusion that collapse at collapsed forks efficiently complete DNA synthesis while single collapsed forks do not, as for simple SSBs.” I couldn’t work out what this sentence means.

#31 We have corrected these typos and endeavored to improve clarity throughout the revised manuscript, as noted in response #24.

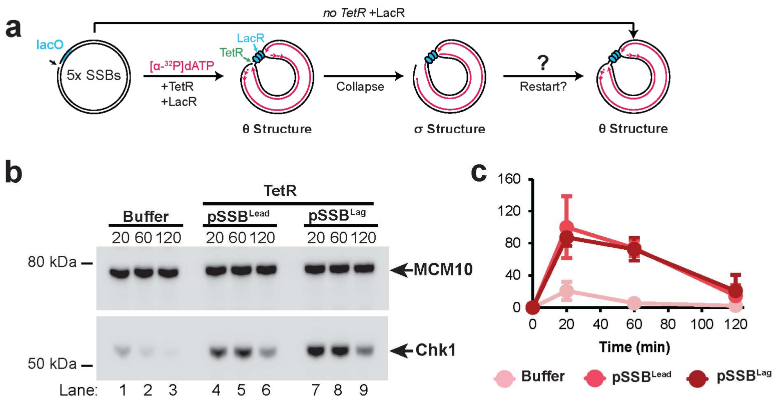

Reviewer Figure R1: Analysis of pCHK1 following leading and lagging collapse

(A) Schematic of the experimental design. pSSB^{LEAD} or pSSB^{LAG} was replicated in Xenopus egg extract in the presence of TetR and LacR to induce leading or lagging strand collapse, respectively. Nascent strands were radiolabeled by inclusion of [α-^{32}P]dATP. TetR was omitted in the buffer control (+Buffer). **(B)** Chromatin samples were collected at the indicated time points (20, 60, 120 min) under buffer, pSSB^{LEAD} (+TetR), and pSSB^{LAG} (+TetR) conditions and analyzed by western blotting for MCM10 (loading control, top) and Chk1 (bottom). Chk1 phosphorylation was assessed by mobility shift. **(C)** Quantification of Chk1 signal from (B) across the indicated time points and conditions. Chk1 phosphorylation was not substantially altered following either leading or lagging collapse relative to the buffer control. This result neither supports nor contradicts the secondary collapse model, because secondary collapse is a rare event that may not generate sufficient single-stranded DNA to substantially alter Chk1 phosphorylation levels. *n* = 3.

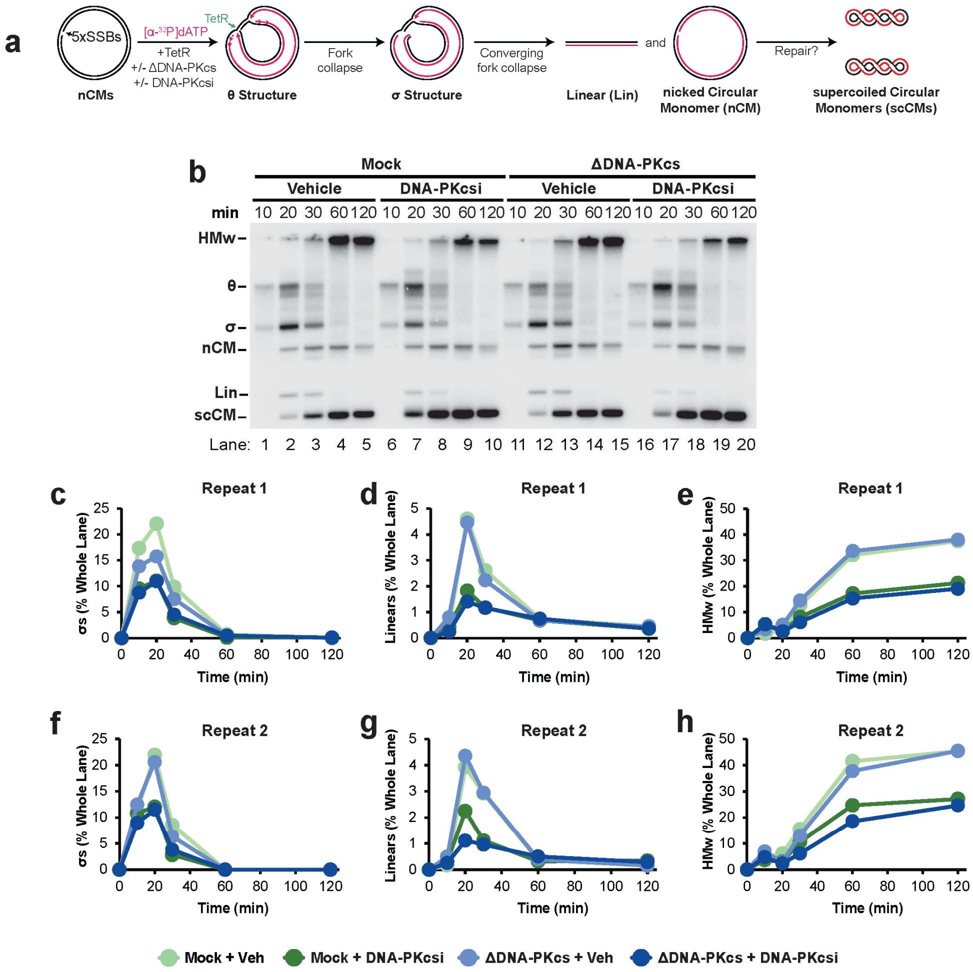

Reviewer Figure R2: Small molecule inhibition, but not depletion, of DNA-PKcs reduces HMw abundance (A) Schematic of the experimental design. Convergent collapse was induced by replication of pSSB in mock- or DNA-PKcs-immunodepleted extracts, each supplemented with either vehicle or DNA-PKcs inhibitor (DNA-PKcsi). Nascent strands were radiolabeled by inclusion of [α-^{32}P]dATP. **(B)** Samples from (A) were separated on an agarose gel and visualized by autoradiography. Key species are indicated: high molecular weight products (HMw), θ structures, σ structures, nicked circular monomers (nCM), linear molecules (Lin), and supercoiled circular monomers (scCM). **(C)** Quantification of σ structures from (B), Repeat 1. **(D)** Quantification of linear molecules from (B), Repeat 1. **(E)** Quantification of HMw products from (B), Repeat 1. **(F)** Quantification of σ structures from (B), Repeat 2. **(G)** Quantification of linear molecules from (B), Repeat 2. **(H)** Quantification of HMw products from (B), Repeat 2. DNA-PKcs immunodepletion did not reduce HMw abundance, indicating that DNA-PKcs is dispensable for repair of deDSBs following convergent collapse. DNA-PKcsi reduced HMw in both mock- and DNA-PKcs-immunodepleted extracts, indicating that the inhibitor effect persisted in the absence of DNA-PKcs and was therefore attributable to off-target activity. *n* = 2 independent experiments.

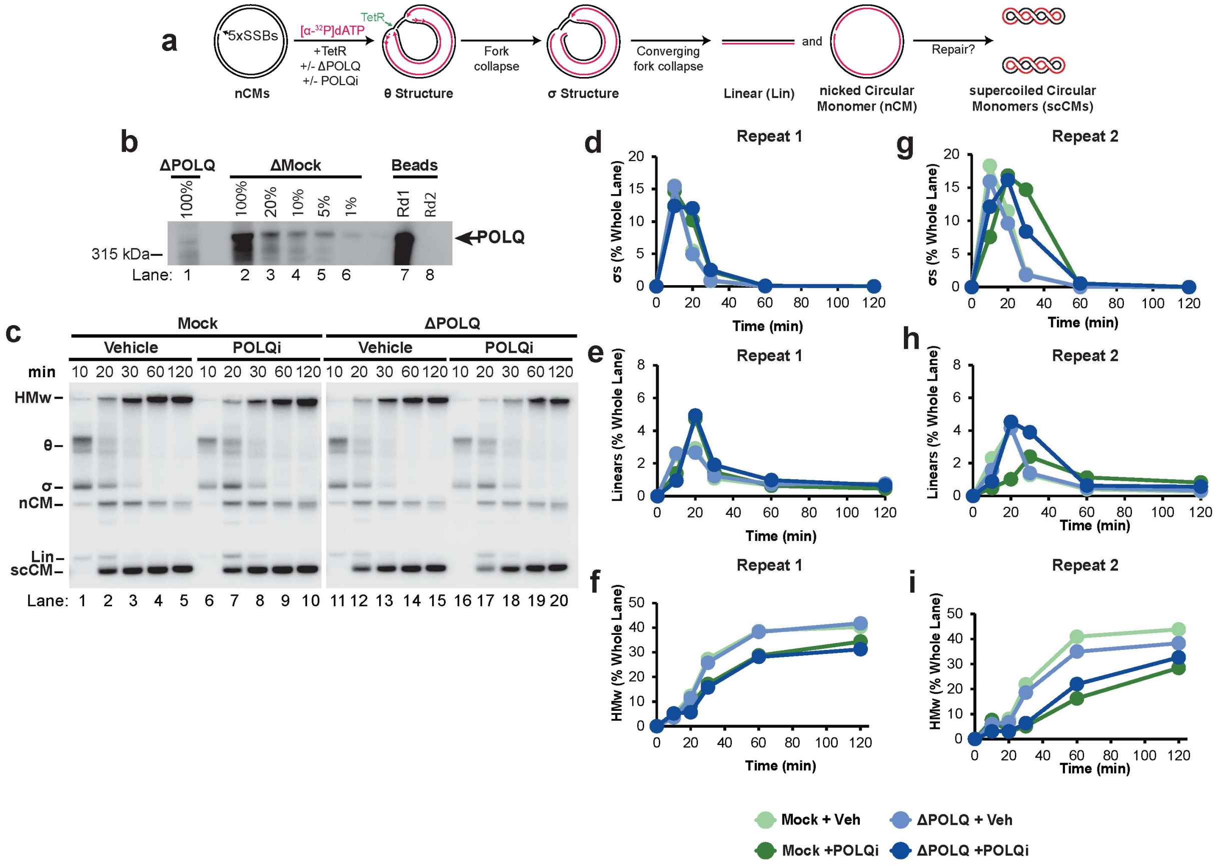

Reviewer Figure R3: Depletion and/or inhibition of POLQ does not impact resolution of collapsed ends
(A) Schematic of the experimental design. Convergent collapse was induced by replication of pSSB in mock- or POLQ-immunodepleted extracts, each supplemented with either vehicle or POLQ inhibitor (POLQi). Nascent strands were radiolabeled by inclusion of [α-^{32}P]dATP. **(B)** Western blot confirming immunodepletion of POLQ from Xenopus egg extract. Serial dilutions of depleted (ΔPOLQ) and mock-depleted (ΔMock) extracts are shown alongside depletion beads (Rd1, Rd2). **(C)** Samples from (A) were separated on an agarose gel and visualized by autoradiography. Key species are indicated: HMw, θ, σ, nCM, Lin, and scCM. **(D)** Quantification of σ structures from (C), Repeat 1. **(E)** Quantification of linear molecules from (C), Repeat 1. **(F)** Quantification of HMw products from (C), Repeat 1. **(G)** Quantification of σ structures from (C), Repeat 2. **(H)** Quantification of linear molecules from (C), Repeat 2. **(I)** Quantification of HMw products from (C), Repeat 2. POLQ immunodepletion did not reduce HMw abundance, indicating that POLQ is dispensable for repair following convergent collapse. POLQi reduced HMw in both mock- and POLQ-immunodepleted extracts, indicating that the inhibitor effect persisted in the absence of POLQ and was therefore attributable to off-target activity. *n* = 2 independent experiments. See also Reviewer Fig. R4.

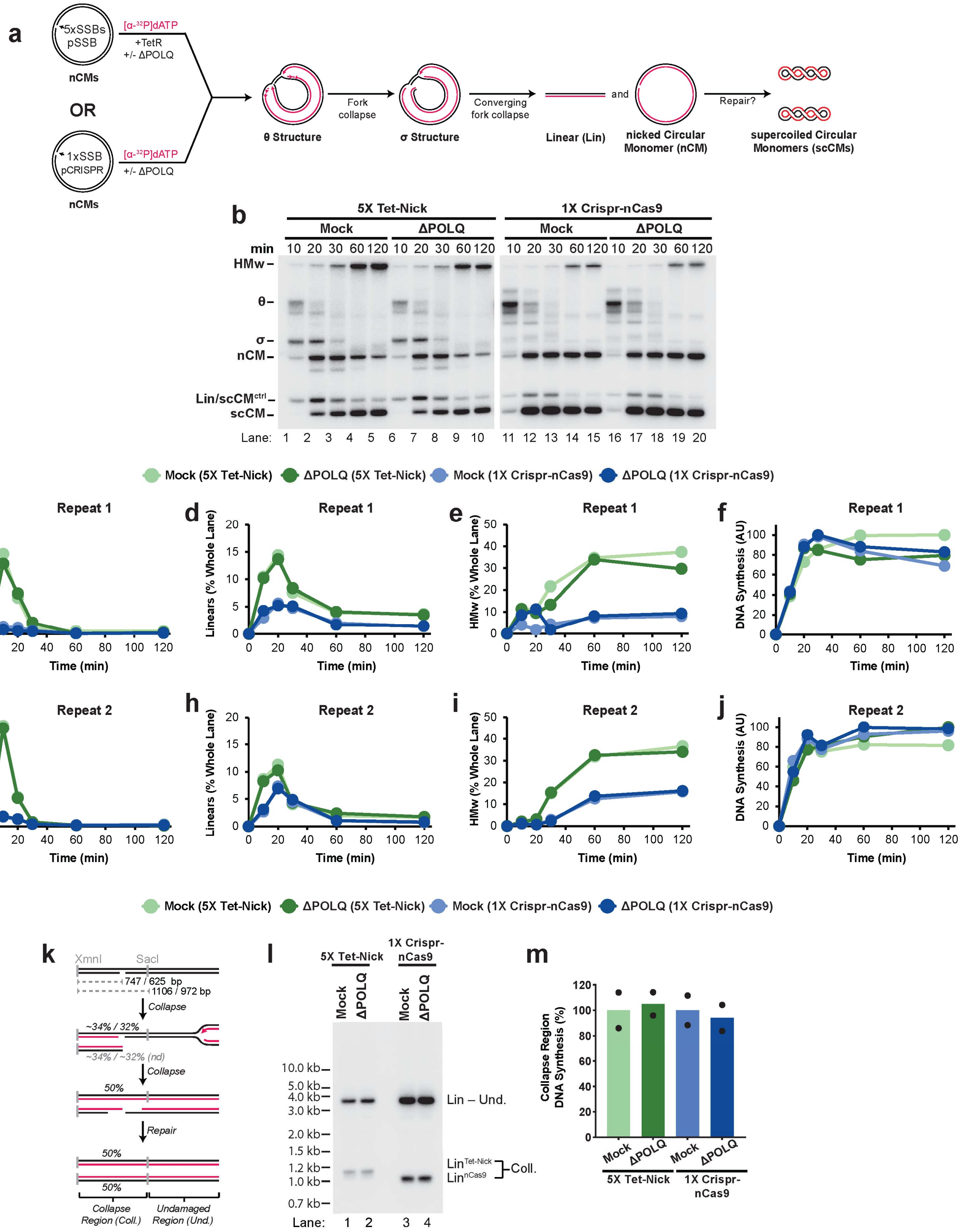

Reviewer Figure R4: Depletion of POLQ does not impact nascent DNA synthesis following collapse
(A) Schematic of the experimental design. Convergent collapse was induced by replication of pSSB (5X Tet-Nick) or pCRISPR (1X Crispr-nCas9) in mock- or POLQ-immunodepleted extracts. Nascent strands were radiolabeled by inclusion of [α-^{32}P]dATP. **(B)** Samples from (A) were separated on an agarose gel and visualized by autoradiography. Key species are indicated: HMw, θ, σ, nCM, Lin/scCM^{ctrl}, and scCM. Lanes 1-10 correspond to the 5X Tet-Nick substrate; lanes 11-20 correspond to the 1X Crispr-nCas9 substrate. **(C)** Quantification of σ structures from (B), Repeat 1. **(D)** Quantification of linear molecules from (B), Repeat 1. **(E)** Quantification of HMw products from (B), Repeat 1. **(F)** Quantification of DNA synthesis from (B), Repeat 1. **(G)** Quantification of σ structures from (B), Repeat 2. **(H)** Quantification of linear molecules from (B), Repeat 2. **(I)** Quantification of HMw products from (B), Repeat 2. **(J)** Quantification of DNA synthesis from (B), Repeat 2. **(K)** Schematic depicting the assay for collapse region DNA synthesis. Purified DNA samples were digested with XmnI and SacI to generate fragments corresponding to the collapse region (Coll.) and an undamaged region (Und.). Expected

signal values are indicated for each substrate. **(L)** Purified DNA samples from T=120 min in (B) were digested with XmnI and SacI, separated on an agarose gel, and visualized by autoradiography. Lanes 1-2, 5X Tet-Nick (Mock and ΔPOLQ); lanes 3-4, 1X Crispr-nCas9 (Mock and ΔPOLQ). **(M)** Quantification of collapse region DNA synthesis from (L). POLQ depletion did not alter collapse region DNA synthesis for either substrate, consistent with the conclusion that POLQ is dispensable for repair following convergent collapse. *n* = 2 independent experiments. See also Reviewer Fig. R3.

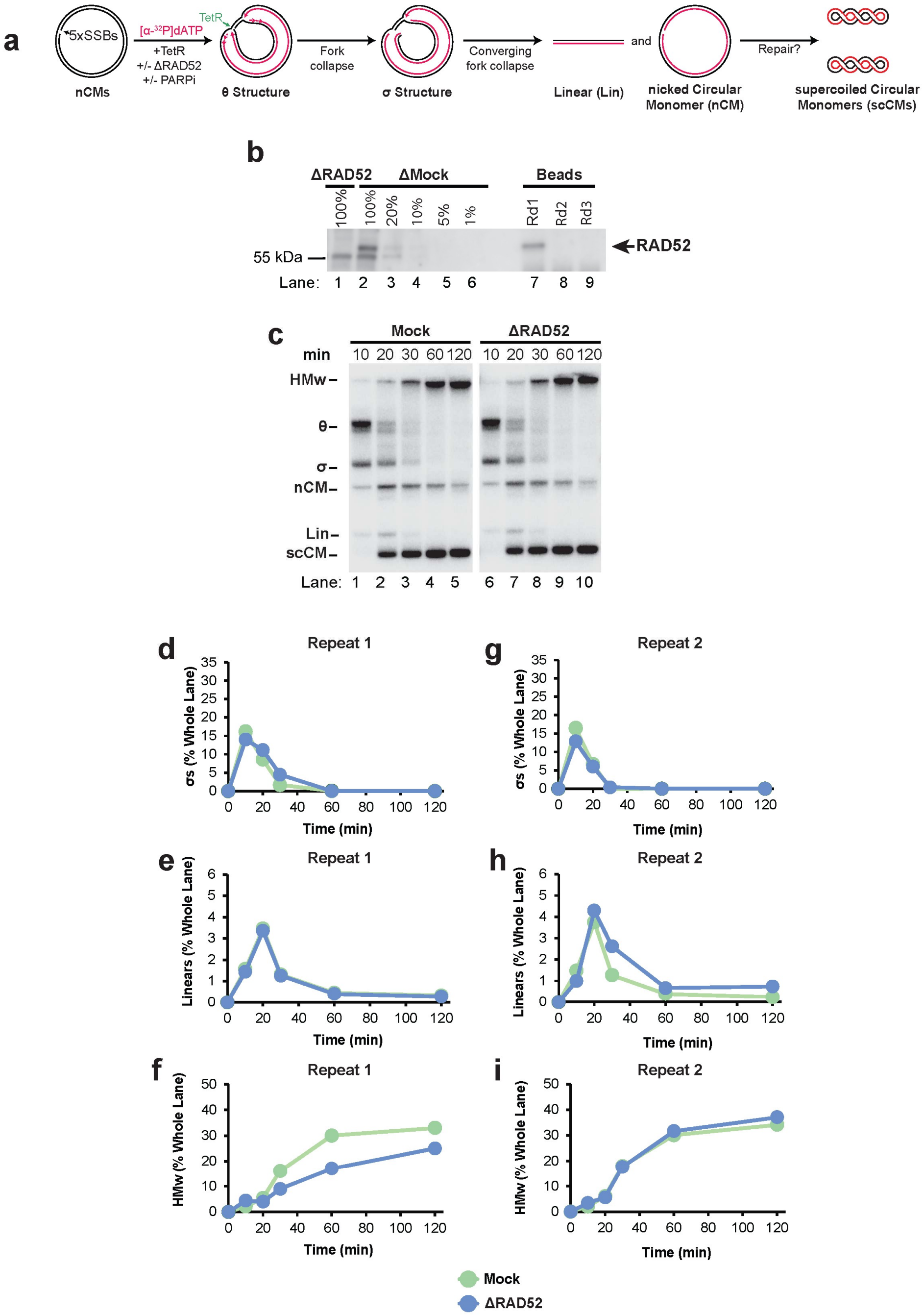

Reviewer Figure R5: Depletion of RAD52 does not impact resolution of collapsed ends

(A) Schematic of the experimental design. Convergent collapse was induced by replication of pSSB in mock- or RAD52-immunodepleted extracts. Nascent strands were radiolabeled by inclusion of [α-^{32}P]dATP. **(B)** Western blot confirming immunodepletion of RAD52 from Xenopus egg extract. Serial dilutions of depleted (ΔRAD52) and mock-depleted (ΔMock) extracts are shown alongside depletion beads (Rd1, Rd2, Rd3). **(C)** Samples from (A) were separated on an agarose gel and visualized by autoradiography. Key species are indicated: HMw, θ, σ, nCM, Lin, and scCM. **(D)** Quantification of σ structures from (C), Repeat 1. **(E)** Quantification of linear molecules from (C), Repeat 1. **(F)** Quantification of HMw products from (C), Repeat 1. **(G)** Quantification of σ structures from (C), Repeat 2. **(H)** Quantification of linear molecules from (C), Repeat 2. **(I)** Quantification of HMw products from (C), Repeat 2. RAD52 immunodepletion did not reduce HMw abundance, indicating that RAD52 is dispensable for repair of deDSBs following convergent collapse. *n* = 2 independent experiments. See also Reviewer Fig. R6.

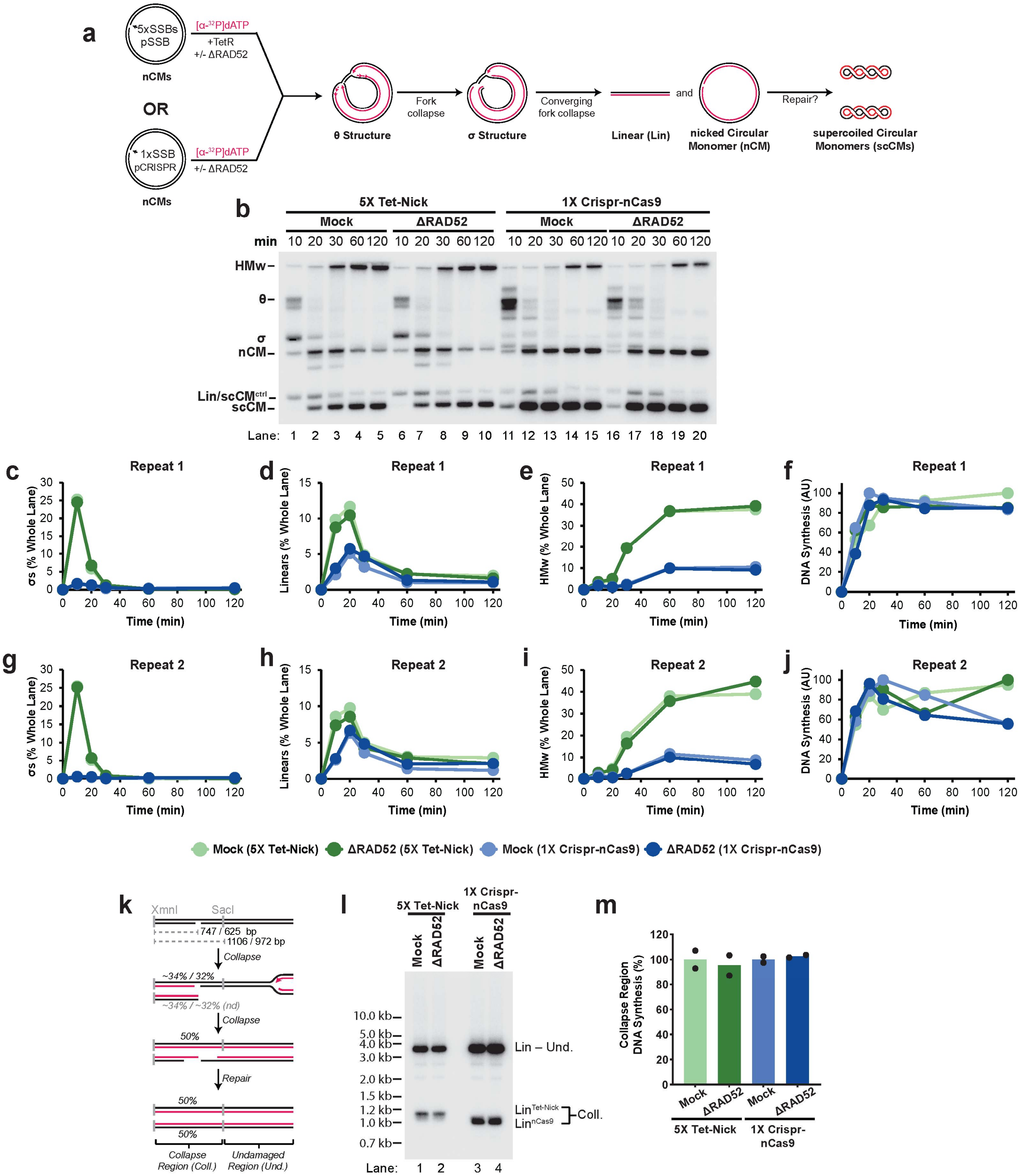

Reviewer Figure R6: Depletion of RAD52 does not impact nascent DNA synthesis following collapse
(A) Schematic of the experimental design. Convergent collapse was induced by replication of pSSB (5X Tet-Nick) or pCRISPR (1X Crispr-nCas9) in mock- or RAD52-immunodepleted extracts. Nascent strands were radiolabeled by inclusion of [α-^{32}P]dATP. **(B)** Samples from (A) were separated on an agarose gel and visualized by autoradiography. Key species are indicated: HMw, θ, σ, nCM, Lin/scCM^{ctrl}, and scCM. Lanes 1-10 correspond to the 5X Tet-Nick substrate; lanes 11-20 correspond to the 1X Crispr-nCas9 substrate. **(C)** Quantification of σ structures from (B), Repeat 1. **(D)** Quantification of linear molecules from (B), Repeat 1. **(E)** Quantification of HMw products from (B), Repeat 1. **(F)** Quantification of DNA synthesis from (B), Repeat 1. **(G)** Quantification of σ structures from (B), Repeat 2. **(H)** Quantification of linear molecules from (B), Repeat 2. **(I)** Quantification of HMw products from (B), Repeat 2. **(J)** Quantification of DNA synthesis from (B), Repeat 2. **(K)** Schematic depicting the assay for collapse region DNA synthesis, as in Reviewer Fig. R4K. **(L)** Purified DNA samples from T=120 min in (B) were digested with XmnI and SacI, separated on an agarose gel, and visualized by autoradiography. Lanes 1-2, 5X Tet-Nick (Mock and ΔRAD52); lanes 3-4, 1X Crispr-nCas9 (Mock and ΔRAD52).

(M) Quantification of collapse region DNA synthesis from (L). RAD52 depletion did not alter collapse region DNA synthesis for either substrate, consistent with the conclusion that RAD52 is dispensable for repair following convergent collapse. *n* = 2 independent experiments. See also Reviewer Fig. R5.

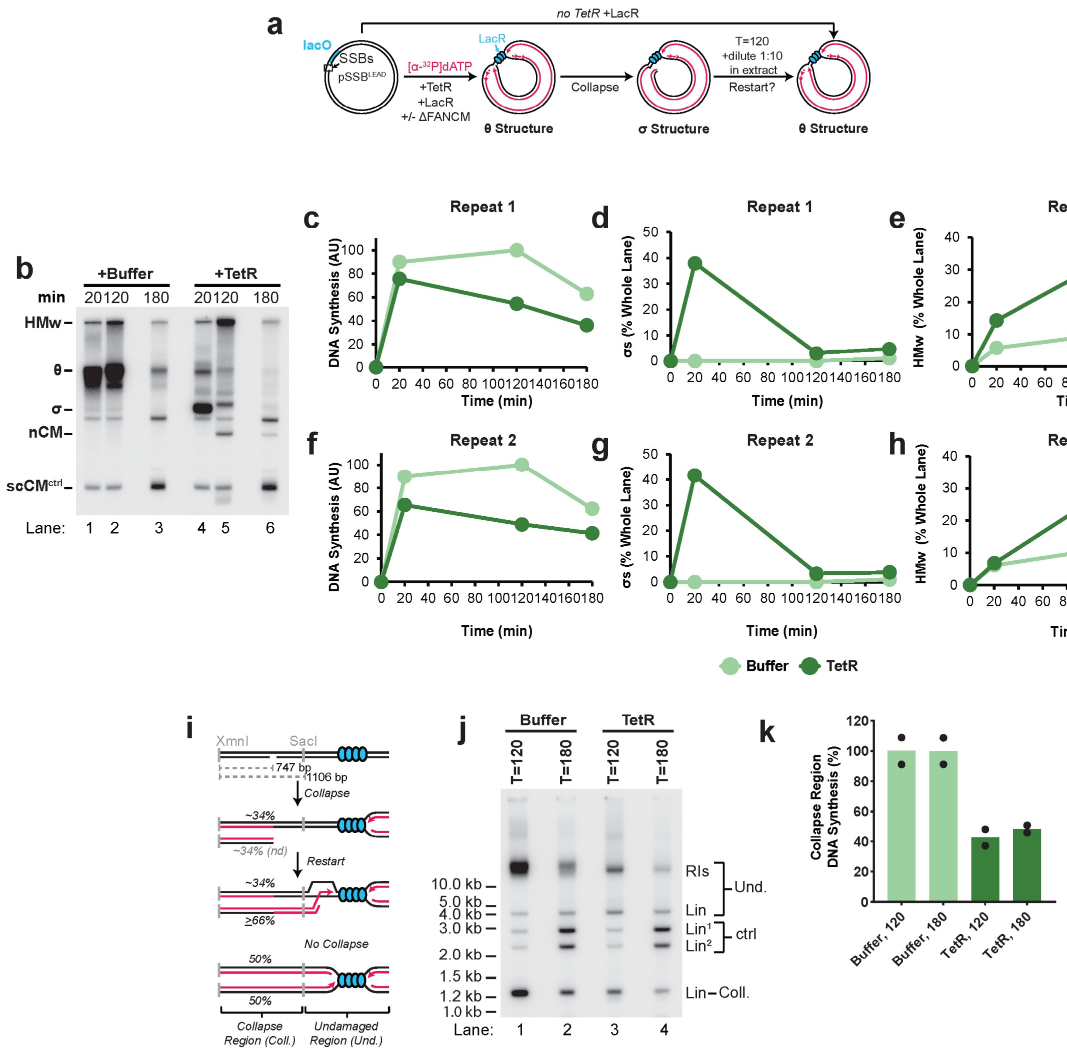

Reviewer Figure R7: Dilution of collapse DNA ends does not elicit fork restart

(A) Schematic of the experimental design. Leading collapse was induced by replication of pSSB^LEAD in Xenopus egg extract in the presence of TetR and LacR. At T=120 min, collapsed reactions were diluted 1:10 into fresh nucleoplasmic extract (NPE) supplemented with [α-^{32}P]dATP to reduce the ratio of collapsed DNA ends to available repair factors. Reactions were then monitored for an additional 60 min (to T=180 min). Under standard reaction conditions, the density of DSBs per nucleoplasmic extract corresponds to approximately 42 DSBs per yeast genome equivalent of DNA (see calculations below). Following 1:10 dilution, this density was reduced to approximately 4 DSBs per yeast genome equivalent, which is comparable to the single DSB per genome at which break-induced replication operates efficiently in S. cerevisiae. **(B)** Samples from (A) were separated on an agarose gel and visualized by autoradiography. Key species are indicated: HMw, θ, σ, nCM, and scCM^{ctrl}. **(C)** Quantification of DNA synthesis from (B), Repeat 1. **(D)** Quantification of σ structures from (B), Repeat 1. **(E)** Quantification of HMw products from (B), Repeat 1. **(F)** Quantification of DNA synthesis from (B), Repeat 2. **(G)** Quantification of σ structures from (B), Repeat 2. **(H)** Quantification of HMw products from (B), Repeat 2. **(I)** Schematic depicting the assay for collapse region DNA synthesis. Purified DNA samples were digested with XmnI and SacI to generate fragments corresponding to the collapse region (Coll.) and an undamaged region (Und.). Expected signal values are indicated for no collapse, collapse without restart, and collapse with restart conditions. **(J)** Purified DNA samples from T=120 and T=180 min were digested with XmnI and SacI, separated on an agarose gel, and visualized by autoradiography. Lanes 1-2, +Buffer (T=120, T=180); lanes 3-4, +TetR (T=120, T=180). Replication intermediates (RIs), undamaged region (Und.), control fragment (ctrl), and collapse region (Coll.) fragments are indicated. **(K)** Quantification of collapse region DNA synthesis from (J). Dilution of collapsed DNA ends into fresh extract did not promote detectable restart of DNA synthesis, arguing against seDSB density as the explanation for the absence of restart. *n* = 2 independent experiments. DSB density calculation: each 1 ul reaction contains 0.33 ul NPE and 4.33 ng of a 4,662 bp plasmid (1 DSB per plasmid), yielding approximately 8.5 x 10^8 DSBs per 0.33 ul NPE. NPE is prepared from 1.19 x 10^8 sperm nuclei (haploid

genome, 3.1 Gb) concentrated into 500 ul, giving 2.38 x 10^5 nuclear equivalents per ul NPE. This yields approximately 1.08 x 10^4 DSBs per nuclear equivalent, or approximately 3.5 DSBs per Mb, equivalent to approximately 42 DSBs per *S. cerevisiae* genome equivalent (12.1 Mb). The 1:10 dilution in this experiment represents ~4.2 DSBs per *S. cerevisiae* genome equivalent and did not result in detectable restart.

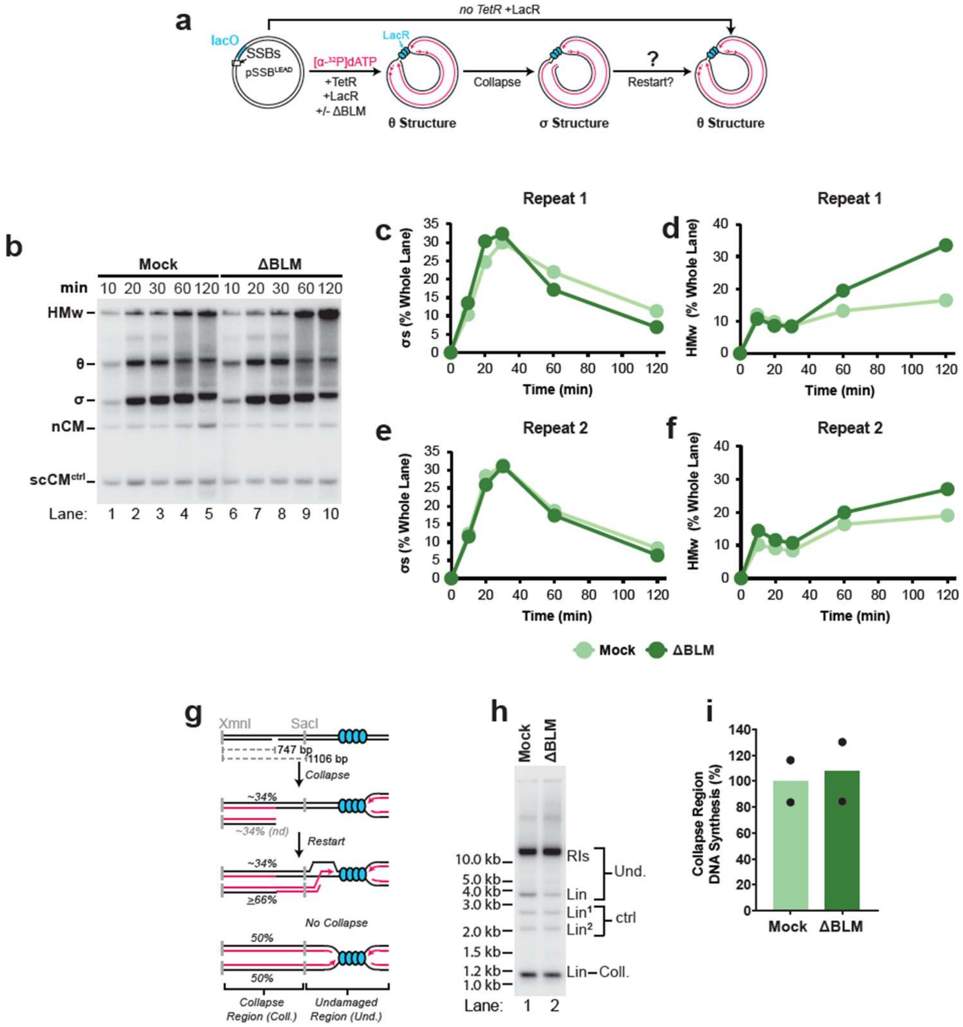

Reviewer Figure R8: Depletion of BLM does not elicit fork restart

(A) Schematic of the experimental design. Leading collapse was induced by replication of pSSB^{LEAD} in mock- or BLM-immunodepleted Xenopus egg extracts in the presence of TetR and LacR. Nascent strands were radiolabeled by inclusion of [α-^{32}P]dATP. **(B)** Samples from (A) were separated on an agarose gel and visualized by autoradiography. Key species are indicated: HMw, θ, σ, nCM, and scCM^{ctrl}. **(C-D)** Quantification of σ structures and HMw products from (B), Repeat 1. **(E-F)** Quantification of σ structures and HMw products from (B), Repeat 2. BLM depletion increased HMw abundance, consistent with accumulation of joint molecules that are normally dissociated by BLM. Lower panels: **(G)** Schematic depicting the assay for collapse region DNA synthesis, as in Fig. 1E. **(H)** Purified DNA samples from T=120 min in (B) were digested with XmnI and SacI, separated on an agarose gel, and visualized by autoradiography. Replication intermediates (RIs), undamaged region (Und.), control fragment (ctrl), and collapse region (Coll.) fragments are indicated. **(I)** Quantification of collapse region DNA synthesis from (F). BLM depletion did not reveal evidence of increased DNA synthesis in the collapse region, indicating that BLM depletion did not unmask restart at single collapsed forks. *n* = 2 independent experiments.

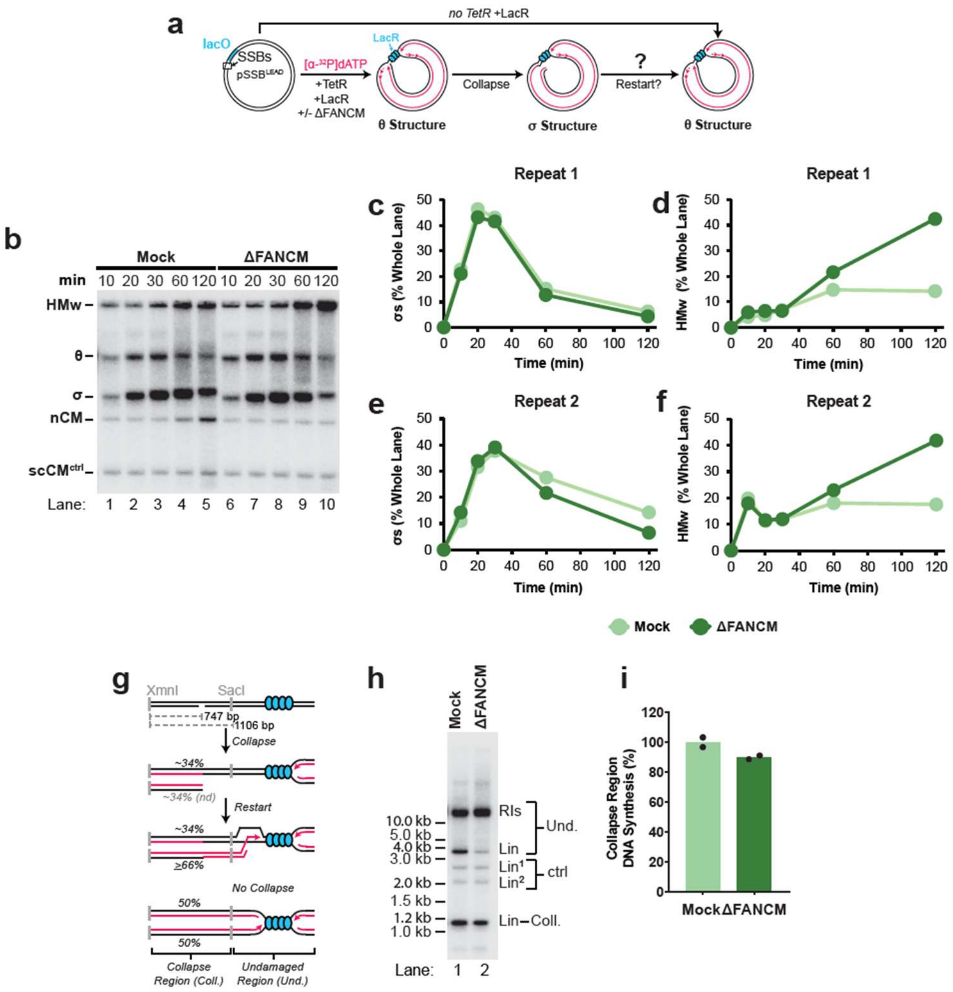

Reviewer Figure R9: Depletion of FANCM does not elicit fork restart

(A) Schematic of the experimental design. Leading collapse was induced by replication of pSSB^{LEAD} in mock- or FANCM-immunodepleted Xenopus egg extracts in the presence of TetR and LacR. Nascent strands were radiolabeled by inclusion of [α-^{32}P]dATP. **(B)** Samples from (A) were separated on an agarose gel and visualized by autoradiography. Key species are indicated: HMw, θ, σ, nCM, and scCM^{ctrl}. **(C-D)** Quantification of σ structures and HMw products from (B), Repeat 1. **(E-F)** Quantification of σ structures and HMw products from (B), Repeat 2. FANCM depletion increased HMw abundance, consistent with accumulation of joint molecules that are normally dissociated by FANCM. Lower panels: **(G)** Schematic depicting the assay for collapse region DNA synthesis, as in Fig. 1E. **(H)** Purified DNA samples from T=120 min in (B) were digested with XmnI and SacI, separated on an agarose gel, and visualized by autoradiography. Replication intermediates (RIs), undamaged region (Und.), control fragment (ctrl), and collapse region (Coll.) fragments are indicated. **(I)** Quantification of collapse region DNA synthesis from (F). FANCM depletion did not reveal evidence of increased DNA synthesis in the collapse region, indicating that FANCM depletion did not unmask restart at single collapsed forks. *n* = 2 independent experiments.
